# Association between substituting macronutrients and all-cause mortality: a network meta-analysis of prospective observational studies

**DOI:** 10.1016/j.eclinm.2024.102807

**Published:** 2024-09-05

**Authors:** Sabina Wallerer, Theodoros Papakonstantinou, Jakub Morze, Julia Stadelmaier, Eva Kiesswetter, Lea Gorenflo, Janett Barbaresko, Edyta Szczerba, Manuela Neuenschwander, William Bell, Tilman Kühn, Szimonetta Lohner, Marta Guasch-Ferré, Georg Hoffmann, Joerg J. Meerpohl, Sabrina Schlesinger, Adriani Nikolakopoulou, Lukas Schwingshackl

**Affiliations:** a Institute for Evidence in Medicine, Faculty of Medicine and Medical Center - University of Freiburg, Freiburg, Germany; b Institute of Medical Biometry and Statistics, Faculty of Medicine and Medical Center - University of Freiburg, Freiburg, Germany; c Department of Life Sciences, Chalmers University of Technology, Gothenburg, Sweden; d College of Medical Sciences, SGMK Copernicus University, Warsaw, Poland; e Cochrane Germany, Cochrane Germany Foundation, Freiburg, Germany; f Institute for Biometrics and Epidemiology, German Diabetes Center, Leibniz Center for Diabetes Research at the Heinrich Heine University Düsseldorf, Germany; g German Center for Diabetes Research (DZD), Partner Düsseldorf, München-Neuherberg, Germany; h The Institute for Global Food Security, School of Biological Sciences, Queen’s University Belfast, Belfast, United Kingdom; i Department of Nutritional Sciences, University of Vienna, Vienna, Austria; j Center for Public Health, Medical University of Vienna, Vienna, Austria; k Heidelberg Institute of Global Health (HIGH), Faculty of Medicine and University Hospital, Heidelberg, Germany; l Cochrane Hungary, Medical School, University of Pécs, Pécs, Hungary; m Department of Public Health Medicine, Medical School, University of Pécs, Pécs, Hungary; n Department of Public Health and Novo Nordisk Center for Basic Metabolic Research, University of Copenhagen, Denmark; o Department of Nutrition, Harvard TH Chan School of Public Health, Boston, MA, USA

**Keywords:** Network meta-analysis, Replacement, Substitution, Mortality, Macronutrients

## Abstract

**Background:**

Suboptimal diet quality is a key risk factor for premature death. Assuming relatively stable energy intake among individuals, changes in nutrient intakes occur by exchanging different nutrients. Therefore we aimed to examine the association of isocaloric substitution of dietary (macro)nutrients with all-cause mortality using network meta-analysis (NMA).

**Methods:**

For this systematic review and NMA of prospective observational studies MEDLINE, Embase, and Scopus were searched from inception to February 13th, 2024. Eligible studies reported substitution analyses for quantity and/or quality of macronutrients, including carbohydrates, proteins, and fatty acids on all-cause mortality. Random-effects NMA were used in order to evaluate the pooled hazard ratios (HR) and 95% confidence intervals (CI) of substituting each included nutrient with another. We assessed risk of bias with the ROBINS-E tool, and the certainty of evidence (CoE) using the Grading of Recommendations Assessment, Development and Evaluations (GRADE) approach. This study is registered with PROSPERO (CRD42023450706).

**Findings:**

Thirty-nine studies with 1,737,644 participants, 395,491 deaths, 297 direct comparisons, and seven nutrient-specific networks were included. Moderate CoE was found for an association with lower mortality risk when replacing 5% of energy intake from carbohydrates with polyunsaturated fatty acids (PUFA; HR: 0.90; 95%CI: 0.84, 0.95), n-6 PUFA (0.85; 0.77, 0.94), n-3 PUFA (0.72; 0.59, 0.86), and plant monounsaturated fatty acids (MUFA; 0.90; 0.85, 0.95), and when replacing 5% of energy from saturated fatty acids (SFA) and trans-fatty acids (TFA), with PUFA, MUFA, and plant-MUFA (HR_range_: 0.75 to 0.91). A lower mortality risk was additionally found when 5% of animal-MUFA was replaced with plant-MUFA, and when replacing animal protein, and SFA with plant protein (HR_range_: 0.81 to 0.87, moderate CoE).

**Interpretation:**

Our results provide practical knowledge for public health professionals and can inform upcoming dietary guidelines. The beneficial association of increasing PUFA (both n-3 and n-6) and (plant-) MUFA intake while reducing carbohydrates, SFA and TFA, along with replacing animal protein and animal-MUFA with plant-based sources of protein and fat (MUFA) on the all-cause mortality risk, underscores the importance of plant-based dietary recommendations.

**Funding:**

None.

**Research in context**

**Evidence before this study**

Suboptimal diet quality is recognized as one of the key risk factors for premature death and chronic diseases. Assuming relatively stable energy intake among individuals, changes in nutrient intakes occur by exchanging different nutrients. Substitution analyses have been widely implemented to assess the associations of replacing nutrients in prospective observational studies. We searched Medline (via Ovid), Embase, and Scopus from inception to February 13th, 2024 for eligible studies, with no restriction on language or outcome. Eligible studies were prospective observational studies that reported the association of macronutrient substitutions and all-cause mortality. Twenty-five out of 39 included cohort studies were judged with “some concerns” in the risk of bias assessment. We assessed the certainty of evidence (CoE) for each comparison using the Grading of Recommendations Assessment, Development and Evaluations (GRADE) approach.

**Added value of this study**

Several systematic reviews of cohort studies have investigated the direct association of different macronutrients and disease or mortality risk, yet not considering isocaloric substitutions, and to the best of our knowledge, no such network meta-analysis (NMA) has been conducted to date. We were able to analyse data from 39 studies with 1,737,644 participants, 395,491 mortality events. Therefore, 297 direct comparisons and seven nutrient-specific networks were examined. Increasing the intake of n-3 polyunsaturated fatty acids (PUFA), n-6 PUFA and (plant-) monounsaturated fatty acids (MUFA) while simultaneously reducing carbohydrates and saturated fatty acids (SFA), as well as replacing animal protein and animal-MUFA with plant-based sources of protein and fat (MUFA) is beneficially associated with lower risk of all-cause mortality, based on moderate CoE.

**Implications of all the available evidence**

The favourable impact on mortality risk of higher fat intake, especially PUFA (both n-3 and n-6) and (plant-) MUFA at the expenses of carbohydrates and SFA, and replacing animal protein and animal-MUFA with plant-based sources of protein and fat (MUFA) reinforces the importance of plant-based dietary guidelines. In addition, the data may diverge from current WHO recommendations on a healthy diet, particularly regarding the limitation of fat intake to no more than 30% of total caloric intake.

## Introduction

Suboptimal diet quality is recognized as one of the key risk factors for premature death and chronic diseases, including cardiovascular diseases (CVD), type 2 diabetes, and cancer.^1^ From a historical perspective, recommendations on macronutrient intake have always been an integral part of dietary guidelines.^2^ For example, in 1980, the Dietary Guidelines for Americans recommended limiting dietary fat to <30% of total energy intake, which was then revised in 2005, to a range from 20 to 35%.^3^ Moreover, the World Health Organization (WHO) recommended recently to limit the daily intake of calories from saturated fatty acids (SFA) to <10% and replacing them with unsaturated fatty acids, and limiting the intake of trans-fatty acids (TFA) to <1%.^4^ The Recommended Dietary Allowance of protein is usually between 10 and 15% of total energy intake (0.8 g protein per kg body weight per day),^5^ whereas the recommended carbohydrate allowance ranges from 45% to 65%.^6^

Assuming relatively stable energy intake levels among individuals, changes in dietary habits are mainly represented by substitutions of different nutrients. In this approach, increased consumption of specific nutrients typically occurs at the expense of other nutrients.^7^ Randomized controlled trials (RCTs) can evaluate health effects of nutrient substitution by comparing arms with distinct dietary interventions. However, due to the scarcity of RCTs with clinically relevant outcomes, including all-cause mortality, and the difficulty of achieving long-term adherence in an RCT, the applicability of findings generated by them is limited. Alternatively, substitution models have been widely implemented to assess the theoretical effect of replacing nutrients in prospective observational studies.^8^ In nutrition research, well-designed prospective cohort studies are the main source of evidence to address the health impact of decade long exposures of populations on various clinical endpoints.^9^ The interpretation of substitution analyses results might be challenging, as usually a small set of comparators is evaluated. Network meta-analysis (NMA) represents a valuable tool to synthesize evidence from nutritional epidemiological studies encompassing substitution analyses in order to compare two or more macronutrient substitutions simultaneously (e.g. ↑ fat vs. ↓ carbohydrates and ↑ fat vs. ↓ protein), and gain knowledge between the clinical endpoints of interest. A NMA allows the computation of indirect estimates from the available direct comparisons. For example, the indirect estimate for the substitution of carbohydrates with fat is derived from direct evidence on the substitution of protein with carbohydrates and on the substitution of protein with fat. Therefore, data from indirect comparisons enables the examination of substitution pairs not explicitly considered in cohort studies.^10^ Depending on the proportion of direct evidence, the indirect evidence contributes to a greater or lesser extent to the network estimate. By combining direct and indirect evidence to form the network estimate more precise results can be obtained. Additionally, NMA facilitates a ranking over all available nutrient substitutions from best to worst.

Several systematic reviews of cohort studies have investigated the direct association of different macronutrients and disease or mortality risk,^11^, ^12^, ^13^ yet not considering isocaloric substitutions, and to the best of our knowledge, no such NMA has been conducted to date.

Therefore, this systematic review with NMA aimed to investigate the isocaloric substitution of macronutrients, as well as types of carbohydrates, proteins, and fatty acids on the risk of all-cause mortality in the general healthy adult population.

## Methods

We report this systematic review with NMA according to the PRISMA Extension for Network Meta-analyses (PRISMA-NMA) checklist^14^ and the PRISMA Statement for Reporting Literature Searches in Systematic Reviews (PRISMA-S).^15^ The protocol of this work was pre-defined and registered on the International Prospective Register of Systematic Reviews (PROSPERO; registration number CRD42023450706).

### Data sources and searches

We conducted a comprehensive systematic literature search in three electronic databases including MEDLINE (via OVID), Embase, and Scopus from inception to February 13th, 2024. No language filter was applied and no restrictions were set on outcomes. The detailed search strategies can be found in Supplemental Appendix 1.

In addition, we conducted backward citation tracking on systematic and narrative reviews, identified by our searches and on all included studies.

### Eligibility criteria

We included studies fulfilling the following eligibility criteria.

#### Population

Adults (aged ≥ 18 years); generally healthy: >2/3 of the study population without a particular condition, i.e., stable coronary heart disease, chronic kidney disease, diabetes, cancer. We excluded studies involving exclusively infants, children, adolescents, or pregnant women.

#### Exposure and comparison

Studies reporting substitutions of different macronutrients, or substitutions of different types of carbohydrates, proteins, or fatty acids, with one another were eligible to be included. Studies reporting estimates for substitution analyses conducted according to established methodology (i.e., statistical approach of leave-one-out method or partition method^8^^,^^16^) were included.

#### Outcome

All-cause mortality. Publications that did not provide any information on all-cause mortality (e.g., studies on CVD, type 2 diabetes, or cancer), were excluded from the present review.

#### Study design

We considered prospective observational studies (e.g., cohort, case-cohort, nested case–control).

Detailed eligibility criteria are displayed in Supplemental Table S1.

### Study selection

After deduplication of search hits using Systematic Review Accelerator,^17^ two reviewers from a group of 10 (EK, ES, JB, JS, JM, LS, MN, SS, SW, WB) independently screened each title/abstract and full text for potentially eligible publications. On the full text level, reasons for exclusion were recorded. Any disagreements were resolved by discussion or with the help of a third reviewer (JM, LS, SS) if no agreement could be reached. The screening process was implemented using Covidence systematic review software.^18^

If multiple publications investigated the same cohort, the one with larger number of cases followed by the one with longer follow-up was included. Conference abstracts with adequate information on methods and results were also considered eligible.

### Data extraction

After identification of eligible publications, two reviewers (SW, LS) extracted the data independently in a piloted data extraction form (Microsoft Excel). Conflicts were solved by discussion with a third reviewer (SS or JM) if no agreement could be reached. We extracted the following data: first author’s name, cohort name, year of publication, location, study design, age, sex, body mass index, number of participants, length of follow-up, outcome assessment, number of cases, exposure assessment (type and number of assessments (i.e., at baseline or repeated)), types of nutrient substituted, unit of substitution (% energy, kcal/d, g/d), covariate adjustment set, risk estimate with 95% confidence interval (CI). If a cohort study presents several risk estimates, the one with maximal adjustment was chosen.

If studies reported the relevant data only in figures, we used the ‘Web plot digitizer’^19^ for extraction.

If a study did not report estimates for all network connections, we contacted study authors to provide them or accessed individual-level data to extract them.

### Risk of bias assessment

Two reviewers out of a group of four (EK, LG, JS, SW) assessed the risk of bias (RoB) of each included study independently and any disagreements were resolved by consensus. We used the Risk Of Bias In Non-randomized Studies - of Exposures (ROBINS-E) tool^20^ to evaluate the RoB and visualized it using the robvis tool.^21^ The RoB assessment includes seven domains of bias: 1. Confounding, 2. measurement of exposure, 3. selection of participants into the study (or into the analysis), 4. post exposure interventions, 5. missing data, 6. measurement of the outcome, and 7. selection of the reported results. As recommended by Higgins et al.,^20^ we used a triage approach if a study did not adjust for all pre-specified confounders (age, sex, smoking, alcohol consumption, education/socioeconomic status, and physical activity). An inadequate adjustment leads to a (very) high risk of bias in the first domain (“confounding”). By using the triage approach, other domains are not assessed if a (very) high risk of bias was assigned to the first domain since the overall judgement will not be influenced any further. In our analyses a very high risk of bias was not reasonable in any domain due to the adjustments made, and the prospective observational nature of the included studies for which differential misclassification is not expected,^22^ post-exposure variables that influence the selection of participants are unlikely, and there was also no major concern regarding selective reporting.

We judged each domain as well as the overall RoB as low, some concerns, and high RoB. Details of the ROBINS-E assessment are provided in Supplemental Appendix 2.

### Statistical analysis

Study estimates for the outcome all-cause mortality (hazard ratio [HR]) were used as effect size in NMA.

All substitution effect sizes were converted to 5% of total energy exchange, to ensure comparability and to mitigate the risk of violating the transitivity assumption.^10^^,^^23^ If isocaloric substitutions were reported per kcal/d or g/d, we calculated the percent exchange as described in Supplemental Appendix 3. If effect sizes were presented per quantiles/unit of intake/exchange, we estimated the linear estimate (5% of total energy exchange) using Greenland and Longnecker method.^24^

If estimates were presented separately within the same publication for men and women or different age-ranges, they were pooled with the fixed-effect model before inclusion into the main analysis. We did not combine estimates for separate cohort studies reported in the same publication. A detailed description of handling of multiple publications reporting on the same study, to ensure we did not break the principle of independence of studies, can be found in Supplemental Appendix 4.

For the NMA, we analysed the data using the following node definitions:

- Network 1: Overall macronutrient network: fat, carbohydrates, protein;
- Network 2: Fatty acids expanded network: SFA, monounsaturated fatty acids (MUFA), polyunsaturated fatty acids (PUFA), TFA, carbohydrates, protein;
- Network 3: MUFA-origin network: animal-MUFA, plant-MUFA, SFA, PUFA, carbohydrates, protein;
- Network 4: PUFA-origin network: n-3 PUFA, n-6 PUFA, SFA, MUFA, TFA, carbohydrates, protein;
- Network 5: Fat-origin subnetwork: animal fat, plant fat, carbohydrates, protein;
- Network 6: Protein-origin subnetwork: animal protein, plant protein, SFA, MUFA, PUFA, TFA, carbohydrates;
- Network 7: Carbohydrate-origin subnetwork: high-quality carbohydrates/polysaccharides, low-quality carbohydrates/mono-/disaccharides, SFA, MUFA, PUFA, TFA, protein.

In case a study did not report higher-tier contrasts (e.g., fat vs. carbohydrates, fat vs. protein), but did specify lower-tier origin-specific contrasts (e.g., SFA vs. carbohydrates, MUFA vs. carbohydrates, etc), we approximated higher-tier contrast by pooling lower tier contrasts. This way we combined networks 2–7 or 3–7 with network 1 or 2 (original nodes → higher-tier node, i.e., SFA, MUFA, PUFA, TFA → fat).

Random-effects pairwise meta-analysis and NMA were used in order to evaluate the pooled relative effect of substituting each included nutrient with another. For pairwise meta-analysis, the heterogeneity variance was estimated with the restricted maximum likelihood estimator (REML). The NMA model was fitted in a frequentist framework using a graph-theoretical approach.^25^ A single heterogeneity parameter was assumed across all treatment comparisons and was estimated via a generalised methods of moments estimate.^26^ The results of the NMA are presented as summary effect estimates with 95% CIs in league tables.

Due to secondary nature of substitution analyses in nutritional epidemiological publications, estimates for all possible comparisons were not always reported. Moreover, some cohort studies did report estimates, showing high relative residual effects for some network connections, and a detailed description on handling of these risk estimates and missing or inconsistent variances can be found in Supplemental Appendix 5.

Analyses were conducted in R 4.2.0 software (R Foundation for Statistical Computing, Vienna, Austria) with netmeta package.^27^

A ranking of different substitutions was conducted by calculating the P-score, a frequentist analogue of the surface under the cumulative ranking curve. P-scores are based on summary effects from NMA and their standard errors. They capture the extent of certainty that one treatment is better than another, averaged over all competing treatments.^28^

Transitivity assumption was evaluated by comparing baseline characteristic (age, sex, geographical location, and body mass index) across all comparisons. Global and local approaches were used to assess incoherence. Specifically, a design-by-treatment interaction model and node-splitting approach were performed.^29^^,^^30^

We used funnel plots and Egger’s linear regression test for funnel plot asymmetry to evaluate dissemination bias and small study effects for each pairwise comparison with at least 10 comparisons.^31^

Pre-specified subgroup analyses (if at least 10 cohort studies for a network were available) were performed for: sex, geographical location, and dietary assessment. To examine the robustness of our findings, sensitivity analyses were conducted by excluding studies with high RoB and by excluding studies with high relative residual effects.

### Certainty of evidence

Finally, for each comparison, two investigators (LS, SW) evaluated the direct, indirect, and network estimates certainty of evidence by using the Grading of Recommendations Assessment, Development and Evaluations (GRADE) approach^32^ for NMA.^33^ By using the ROBINS-E tool, the initial certainty of evidence level is “high” for observational studies.^34^ However, the certainty of evidence can be downgraded (up to three levels) for the GRADE domain.

Direct evidence was rated based on RoB, inconsistency, indirectness, and publication bias (if at least 10 cohort studies were available). If the certainty of direct evidence was high and its contribution was at least as much as that of the indirect evidence, we did not rate the indirect evidence.^33^ If the rating of indirect evidence was necessary, we used the certainty of direct estimates to inform indirect estimates considering the lowest of the ratings of the two direct comparisons forming the most dominant first-order loop. In the presence of serious intransitivity, we rated down the certainty of the indirect estimate. To establish the certainty of network estimates, we compared the ratings for direct and indirect estimates. The estimate with the higher certainty was chosen and rated down if incoherence and/or imprecision were detected.^33^ In the presence of a convincing dose–response gradient or large effect, NMA estimates were rated up.^35^^,^^36^ A detailed description of the GRADEing procedure can be found in Supplemental Appendix 6.

Evidence profiles were created to summarize the evidence in a transparent and informative format.^37^ These contain information on the type of comparison, the number of included studies, the effect estimates and corresponding 95% CI of the direct, indirect and network evidence, the overall rating and the domain-specific judgements with explanations for down- or upgrading as informative footnotes.

The certainty of evidence is classified as high, moderate, low or very low.^38^

### Ethics statement

An ethical approval was not required since this is an NMA of publicly available data.

### Role of funding source

There was no funding source for this study.

## Results

**Fig. 1:**
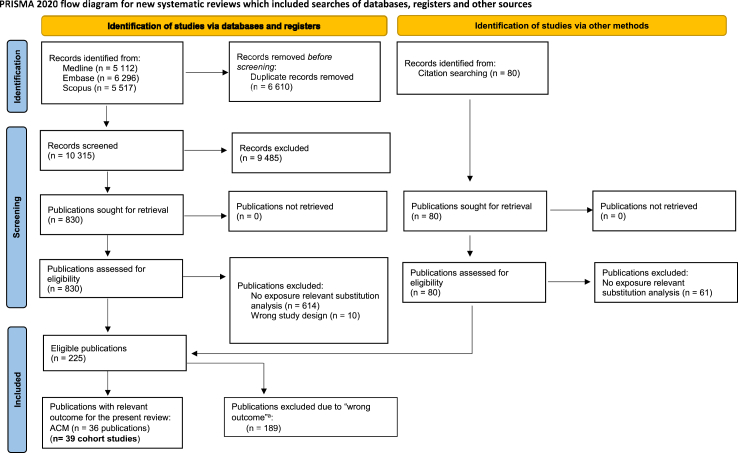
Flow chart of the process for study selection. ACM all-cause mortality.

**Fig. 2:**
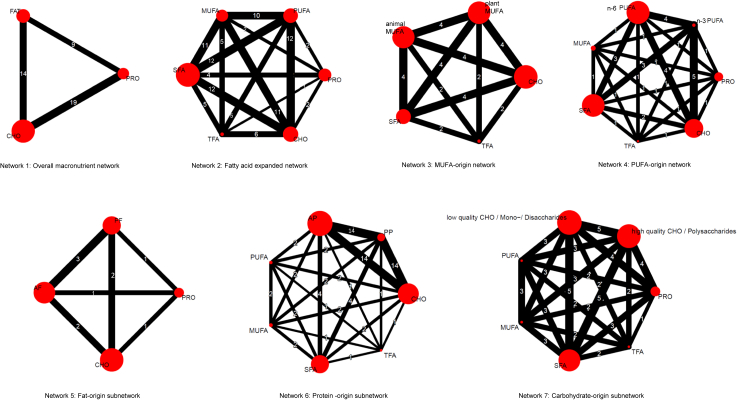
Network plots for all seven networks. The size of the red nodes corresponds to the inverse variance, and the lines correspond to the number of comparisons between arms. AF animal fat; AP animal protein; CHO carbohydrates; MUFA monounsaturated fatty acids; n-3 PUFA omega-3 polyunsaturated fatty acids; n-6 PUFA omega-6 polyunsaturated fatty acids; PF plant fat; PP plant protein; PRO protein; PUFA polyunsaturated fatty acids; SFA saturated fatty acids; TFA trans-fatty acids.

The database searches resulted in 16,925 references. After deduplication, we screened eligibility of 10,315 titles/abstracts and in a subsequent step 830 full texts. Reasons for exclusion of full texts are given in Supplemental Appendix 7. Additionally, 19 relevant full texts were identified via hand search. The flow diagram of the search and screening process is depicted in Fig. 1. Finally, we included 39 studies (36 publications) with 1,737,644 participants, 395,491 mortality events, 297 direct comparisons, and analysed seven nutrient-specific networks in the systematic review^12^^,^^39^, ^40^, ^41^, ^42^, ^43^, ^44^, ^45^, ^46^, ^47^, ^48^, ^49^, ^50^, ^51^, ^52^, ^53^, ^54^, ^55^, ^56^, ^57^, ^58^, ^59^, ^60^, ^61^, ^62^, ^63^, ^64^, ^65^, ^66^, ^67^, ^68^, ^69^, ^70^, ^71^, ^72^, ^73^ (Fig. 2).

For one cohort study, we received unpublished data from the authors,^48^ and for another we conducted additional isocaloric substitution analyses due to data access^50^ (Supplemental Appendix 8). One study was not included in the NMA, since authors did not respond to our queries.^39^ There were minor deviations from the registered protocol, which can be found in Supplemental Appendix 9.

### Study characteristics

Detailed information of the study characteristics can be found in Supplemental Tables S2–S4.

Thirteen publications including 16 cohort studies were conducted in the US,^48^^,^^51^^,^^52^^,^^56^^,^^57^^,^^61^, ^62^, ^63^, ^64^^,^^67^^,^^69^^,^^70^^,^^72^ 12 cohort studies in Europe,^12^^,^^40^^,^^44^, ^45^, ^46^, ^47^^,^^49^^,^^50^^,^^54^^,^^55^^,^^59^^,^^65^ 9 cohort studies in Asia,^39^^,^^41^^,^^53^^,^^58^^,^^60^^,^^66^^,^^68^^,^^71^^,^^73^ one cohort study in Australia,^42^ and one publication includes a cohort with participants from 18 countries and five continents.^43^ In all cohort studies except 10 (using consecutive 24-h recalls^50^^,^^56^, ^57^, ^58^^,^^61^^,^^62^^,^^68^^,^^69^^,^^71^^,^^73^ or dietary records^46^^,^^65^), diet was assessed using validated food-frequency questionnaires. In 15 cohort studies, diet was assessed at multiple time points and averages (or measures) of intake were used for the analysis.^47^, ^48^, ^49^^,^^54^^,^^55^^,^^58^^,^^59^^,^^63^^,^^67^^,^^68^^,^^71^^,^^73^ Five cohort studies used repeated dietary measurements for some, but not all participants.^50^^,^^57^^,^^61^^,^^62^^,^^69^ The mean follow-up duration was 14 years (range: 4–27), and most included both men and women (72% of cohort studies).

We evaluated the RoB in 38 cohort studies (three publications included two cohorts respectively and were evaluated separately). In one cohort study the RoB assessment was not possible, since findings were published as conference abstract only.^62^ However, this cohort study was based on NHANES, which was judged as high RoB due to an insufficient exposure assessment. According to our evaluation, 25 cohort studies had some concerns in the overall RoB,^12^^,^^41^^,^^42^^,^^45^, ^46^, ^47^, ^48^^,^^51^, ^52^, ^53^^,^^58^^,^^60^^,^^63^, ^64^, ^65^, ^66^, ^67^, ^68^^,^^70^, ^71^, ^72^, ^73^ and 13 were judged as high RoB.^39^^,^^40^^,^^43^^,^^44^^,^^49^^,^^50^^,^^54^, ^55^, ^56^, ^57^^,^^59^^,^^61^^,^^69^ Eight cohort studies were rated as high RoB due to insufficient adjustment of confounders,^39^^,^^40^^,^^43^^,^^44^^,^^49^^,^^54^, ^55^, ^56^ four cohort studies due to inadequate exposure assessment,^50^^,^^57^^,^^61^^,^^69^ and one cohort study due to a very high proportion of missing data^59^ (Supplemental Fig. S1).

### Overall macronutrient network

We found moderate certainty of evidence for a small association with lower risk of all-cause mortality, when replacing 5% of energy from carbohydrates with total fat (HR: 0.97; 95% CI: 0.96, 1.00), whereas no association for replacing protein with carbohydrates or fat was observed (low certainty) (Fig. 2, Supplemental Fig. S2 and Tables S5 and S6).

### Fat-specific networks

We found moderate certainty of evidence for an association with lower risk of all-cause mortality when replacing 5% of energy from SFA and TFA, with PUFA (HR: 0.86; 95% CI: 0.81, 0.91; HR: 0.75; 95% CI: 0.67, 0.84), MUFA (HR: 0.91; 95% CI: 0.86, 0.97; HR: 0.80; 95% CI: 0.72, 0.89) or plant-MUFA (HR: 0.85; 95% CI: 0.80, 0.90; HR: 0.79; 95% CI: 0.67, 0.94). Moderate certainty of evidence was also found for an association for all-cause mortality when TFA was exchanged with SFA (HR: 0.87; 95% CI: 0.78, 0.97), and carbohydrates with TFA (HR: 1.20; 95% CI: 1.08, 1.33) (Fig. 2, Table 1, Supplemental Fig. S3 and Tables S7 and S8). Replacing SFA with n-6 or n-3 PUFA was also inversely related to mortality risk (HR: 0.82; 95% CI: 0.74, 0.92; HR: 0.69; 95% CI: 0.57, 0.83; both moderate certainty), whereas no association of higher intakes of SFA at the expenses of carbohydrates or protein, and between higher MUFA/PUFA intake at the expenses of protein was observed (low or very low certainty) (Fig. 2, Table 1, Supplemental Fig. S3 and Tables S7–S9).

**Table 1:** GRADE assessment of direct, indirect and network estimates for the main fatty acid networks (5% isocaloric energy substitution).

Network 2 fatty acid expanded network (n = 1,102,268 participants; n = 224,319 mortality events)								
		Direct evidence			Indirect evidence		Network Meta-Analysis	
Comparison	N studies	Proportion direct evidence	HR (95% CI)	Certainty of evidence	HR (95% CI)	Certainty of evidence	HR (95% CI)	Certainty of evidence
↑ PUFA								
↓ MUFA	10	88	0.92 [0.86, 0.99]	⨁⨁⨁◯^a^	1.11 [0.92, 1.35]	⨁⨁◯◯	0.94 [0.88, 1.01]	⨁⨁◯◯^d^
↓ SFA	12	90	0.86 [0.80, 0.92]	⨁⨁⨁◯^a^	0.84 [0.69; 1.03]	⨁⨁◯◯	0.86 [0.81, 0.91]	⨁⨁⨁◯
↓ TFA	5	68	0.64 [0.56, 0.73]	⨁⨁⨁◯^a^	1.07 [0.88, 1.30]	⨁⨁◯◯	0.75 [0.67, 0.84]	⨁⨁⨁◯
↓ CHO	12	88	0.90 [0.84, 0.96]	⨁⨁◯◯^a^^,^^c^	0.88 [0.74, 1.05]	⨁⨁⨁◯	0.90 [0.84, 0.95]	⨁⨁⨁◯
↓ PRO	2	54	0.99 [0.86, 1.14]	⨁⨁◯◯^b^	0.82 [0.70; 0.95]	⨁⨁◯◯	0.91 [0.82, 1.01]	⨁◯◯◯^d^
↑ MUFA								
↓ SFA	11	88	0.91 [0.86, 0.97]	⨁⨁⨁◯^a^	0.90 [0.75; 1.08]	⨁⨁⨁◯	0.91 [0.86, 0.97]	⨁⨁⨁◯
↓ TFA	5	70	0.74 [0.65, 0.84]	⨁⨁⨁◯^a^	0.95 [0.78, 1.16]	⨁⨁◯◯	0.80 [0.72, 0.89]	⨁⨁⨁◯
↓ CHO	11	86	0.94 [0.88, 1.01]	⨁⨁⨁◯^a^	1.01 [0.86, 1.19]	⨁⨁⨁◯	0.95 [0.90, 1.01]	⨁⨁◯◯^d^
↓ PRO	2	51	0.95 [0.82, 1.09]	⨁◯◯◯^b^^,^^c^	0.98 [0.85; 1.14]	⨁⨁◯◯	0.96 [0.87, 1.07]	⨁◯◯◯^d^
↑ SFA								
↓ TFA	5	68	0.85 [0.75, 0.97]	⨁⨁⨁◯^a^	0.92 [0.76, 1.12]	⨁⨁◯◯	0.87 [0.78, 0.97]	⨁⨁⨁◯
↓ CHO	12	86	1.06 [1.00, 1.13]	⨁⨁⨁◯^a^	0.95 [0.82; 1.11]	⨁⨁◯◯	1.04 [0.99, 1.11]	⨁⨁◯◯^d^
↓ PRO	4	77	1.01 [0.91, 1.13]	⨁⨁◯◯^a^^,^^c^	1.23 [1.01, 1.51]	⨁⨁◯◯	1.06 [0.96, 1.16]	⨁◯◯◯^d^
↑ TFA								
↓ CHO	6	86	1.18 [1.05, 1.32]	⨁⨁◯◯^a^^,^^c^	1.30 [0.98, 1.72]	⨁⨁⨁◯	1.20 [1.08, 1.33]	⨁⨁⨁◯
↓ PRO	1	6	1.04 [0.60, 1.82]	⨁⨁◯◯^b^	1.22 [1.06; 1.41]	⨁⨁◯◯	1.21 [1.05, 1.39]	⨁⨁◯◯

Network 3: MUFA-origin network (n = 628,803 participants; n = 151,006 mortality events)								
		Direct evidence			Indirect evidence		Network Meta-Analysis	
Comparison	N studies	Proportion direct evidence	HR (95% CI)	Certainty of evidence	HR (95% CI)	Certainty of evidence	HR (95% CI)	Certainty of evidence
↑ Plant-MUFA								
↓ Animal-MUFA	4	100	0.81 [0.76, 0.85]	⨁⨁⨁◯^a^	NA	NA	0.81 [0.76, 0.85]	⨁⨁⨁◯
↓ SFA	4	97	0.85 [0.80, 0.90]	⨁⨁⨁◯^a^	0.88 [0.64, 1.22]	⨁⨁⨁◯	0.85 [0.80, 0.90]	⨁⨁⨁◯
↓ TFA	2	96	0.78 [0.66, 0.92]	⨁⨁⨁◯^a^	1.25 [0.57, 2.74]	⨁⨁⨁◯	0.79 [0.67, 0.94]	⨁⨁⨁◯
↓ CHO	4	99	0.90 [0.85, 0.95]	⨁⨁⨁◯^a^	1.11 [0.53, 2.34]	⨁⨁⨁◯	0.90 [0.85, 0.95]	⨁⨁⨁◯
↑ Animal-MUFA								
↓ SFA	4	96	1.05 [0.98, 1.12]	⨁⨁⨁◯^a^	1.20 [0.86, 1.68]	⨁⨁⨁◯	1.05 [0.99, 1.12]	⨁⨁◯◯^d^
↓ TFA	2	92	1.01 [0.85, 1.20]	⨁⨁⨁◯^a^	0.75 [0.42, 1.34]	⨁⨁⨁◯	0.99 [0.84, 1.16]	⨁⨁◯◯^d^
↓ CHO	4	99	1.12 [1.05, 1.19]	⨁⨁⨁◯^a^	1.07 [0.55, 2.12]	⨁⨁⨁◯	1.12 [1.05, 1.18]	⨁⨁⨁◯

Network 4: PUFA-origin network (n = 884,003 participants; n = 179,859 mortality events)								
		Direct evidence			Indirect evidence		Network Meta-Analysis	
Comparison	N studies	Proportion direct evidence	HR (95% CI)	Certainty of evidence	HR (95% CI)	Certainty of evidence	HR (95% CI)	Certainty of evidence
↑ n-3 PUFA								
↓ n-6 PUFA	4	79	0.92 [0.74, 1.13]	⨁⨁⨁◯^a^	0.60 [0.40, 0.90]	⨁⨁⨁◯	0.84 [0.70, 1.01]	⨁⨁◯◯^d^
↓ MUFA	1	52	0.81 [0.57, 1.15]	⨁⨁◯◯^b^	0.70 [0.49, 1.01]	⨁⨁◯◯	0.75 [0.58, 0.97]	⨁⨁◯◯
↓ SFA	3	88	0.70 [0.58, 0.86]	⨁⨁⨁◯^a^	0.59 [0.35, 0.99]	⨁⨁⨁◯	0.69 [0.57, 0.83]	⨁⨁⨁◯
↓ TFA	1	86	0.83 [0.43, 1.58]	⨁⨁◯◯^b^	0.51 [0.10, 2.51]	⨁⨁◯◯	0.77 [0.42, 1.41]	⨁◯◯◯^e^
↓ CHO	5	83	0.82 [0.66, 1.01]	⨁⨁⨁◯^a^	0.38 [0.24, 0.59]	⨁⨁⨁◯	0.72 [0.59, 0.86]	⨁⨁⨁◯
↓ PRO	1	48	0.83 [0.59, 1.17]	⨁⨁◯◯^b^	0.73 [0.53, 1.01]	⨁⨁◯◯	0.78 [0.61, 0.98]	⨁⨁◯◯
↑ n-6 PUFA								
↓ MUFA	1	78	0.96 [0.76, 1.21]	⨁⨁◯◯^b^	0.70 [0.45, 1.08]	⨁⨁◯◯	0.90 [0.73, 1.10]	⨁◯◯◯^d^
↓ SFA	3	90	0.86 [0.77, 0.97]	⨁⨁⨁◯^a^	0.55 [0.39, 0.78]	⨁⨁⨁◯	0.82 [0.74, 0.92]	⨁⨁⨁◯
↓ TFA	1	97	0.98 [0.54, 1.78]	⨁⨁◯◯^b^	0.12 [0.005, 2.95]	⨁⨁◯◯	0.92 [0.51, 1.64]	⨁◯◯◯^e^
↓ CHO	4	95	0.85 [0.77, 0.94]	⨁⨁⨁◯^a^	0.89 [0.55, 1.42]	⨁⨁⨁◯	0.85 [0.77, 0.94]	⨁⨁⨁◯
↓ PRO	1	74	0.99 [0.81, 1.22]	⨁⨁◯◯^b^	0.76 [0.54, 1.08]	⨁⨁◯◯	0.93 [0.77, 1.11]	⨁◯◯◯^d^

⨁⨁⨁⨁ High; ⨁⨁⨁◯ Moderate; ⨁⨁◯◯ Low; ⨁◯◯◯ Very low.

95% CI 95% confidence interval; animal-MUFA monounsaturated fatty acids of animal origin; CHO carbohydrates; GRADE Grading of Recommendations Assessment, Development and Evaluations; HR hazard ratio; MUFA monounsaturated fatty acids; n-3 PUFA n-3 polyunsaturated fatty acids; n-6 PUFA n-6 polyunsaturated fatty acids; NA not applicable (the proportion of evidence was 100% for the direct estimate); plant-MUFA monounsaturated fatty acids of plant origin; PRO protein, RoB risk of bias, SFA saturated fatty acids; TFA trans-fatty acids.

a Downgraded by 1 level for RoB: less than 2/3 of the studies (and their contributing weight) were rated with a low RoB, and less than 2/3 of the studies were rated with a high RoB, OR more than 2/3 of the studies (and their contributing weight) were rated with a high RoB, but the effect estimate in the subgroup analysis, excluding studies with a high RoB, was robust.

b Downgraded by 2 levels for RoB: More than 2/3 of the studies (and their contributing weight) were rated with a high RoB. No subgroup analysis for RoB could be conducted to test to robustness of the effect estimates.

c Downgraded by 1 level for inconsistency: The point estimates differ substantially between primary studies, and the corresponding 95% CI overlap only minimally or not at all. We found no clinical or methodological explanation for this inconsistency.

d Downgraded by 1 level for imprecision: The 95% CI includes a RR/HR of 1 and the 95% CI is not narrow (maximal width of 0.05).

e Downgraded by 2 levels for imprecision: The 95% CI includes a RR/HR of 1 and the ratio of the upper to the lower CI bound is > 3.

Additionally, replacing 5% of energy from carbohydrates with plant fat (HR: 0.95, 95% CI: 0.93, 0.98; low certainty), PUFA (HR: 0.90; 95% CI: 0.84, 0.95; moderate certainty), plant-MUFA (HR: 0.90; 95% CI: 0.85, 0.95; moderate certainty), n-6 PUFA (HR: 0.85; 95% CI: 0.77, 0.94; moderate certainty), and n-3 PUFA (HR: 0.72; 95% CI: 0.59, 0.86; moderate certainty), showed an association with a lower all-cause mortality risk (Fig. 2, Table 1, Supplemental Fig. S3 and Tables S7–S11).

Replacing 5% of animal-MUFA, with plant-MUFA was inversely related to mortality risk (HR: 0.81; 95% CI: 0.76, 0.85; moderate certainty), whereas replacing carbohydrates with animal-MUFA was associated with increased risk (HR: 1.12; 95% CI: 1.05, 1.18; moderate certainty). No association between replacement of SFA or TFA with animal-MUFA was observed (low certainty) (Fig. 2, Table 1 and Supplemental Table S8).

### Protein-specific networks

We found moderate certainty of evidence for an association with lower risk of all-cause mortality, when replacing 5% of energy from animal protein, SFA, and carbohydrates with plant protein (HR: 0.87; 95% CI: 0.84, 0.91; HR: 0.86; 95% CI: 0.82, 0.91; HR: 0.88; 95% CI: 0.84, 0.91), whereas the certainty of evidence for the favourable association of replacing PUFA with plant protein was rated as low. No association between higher intakes of animal protein at the expenses of fatty acids was observed based on low or very low certainty of evidence (Fig. 2, Table 2, Supplemental Fig. S4 and Table S12).

**Table 2:** GRADE assessment of direct, indirect and network estimates for the protein-origin subnetwork (5% isocaloric energy substitution): number of participants (n = 1,050,971) and number of mortality events (n = 228,500).

Comparison	N studies	Direct evidence			Indirect evidence		Network Meta-Analysis	
		Proportion direct evidence	HR (95% CI)	Certainty of evidence	HR (95% CI)	Certainty of evidence	HR (95% CI)	Certainty of evidence
↑ PP								
↓ AP	14	79	0.87 [0.83, 0.91]	⨁⨁⨁◯^a^	0.88 [0.81, 0.97]	⨁⨁◯◯	0.87 [0.84, 0.91]	⨁⨁⨁◯
↓ PUFA	2	50	0.98 [0.88, 1.10]	⨁⨁◯◯^b^	0.76 [0.68, 0.85]	⨁⨁◯◯	0.86 [0.80, 0.94]	⨁⨁◯◯
↓ MUFA	2	55	1.02 [0.91, 1.15]	⨁◯◯◯^b^^,^^c^	0.80 [0.71, 0.91]	⨁◯◯◯	0.92 [0.84, 1.00]	⨁◯◯◯^d^^,^^e^
↓ SFA	4	59	0.90 [0.84, 0.97]	⨁⨁⨁◯^a^	0.81 [0.75, 0.89]	⨁⨁⨁◯	0.86 [0.82, 0.91]	⨁⨁⨁◯
↓ TFA	1	97	0.93 [0.53, 1.64]	⨁⨁◯◯^b^	0.11 [0.005, 2.31]	⨁⨁◯◯	0.87 [0.50, 1.51]	⨁◯◯◯^f^
↓ CHO	14	89	0.88 [0.85, 0.92]	⨁⨁⨁◯^a^	0.82 [0.73, 0.93]	⨁⨁◯◯	0.88 [0.84, 0.91]	⨁⨁⨁◯
↑ AP								
↓ PUFA	2	76	1.01 [0.92, 1.10]	⨁⨁◯◯^b^	0.92 [0.79, 1.08]	⨁⨁◯◯	0.99 [0.92, 1.07]	⨁◯◯◯^e^
↓ MUFA	2	81	1.04 [0.95, 1.14]	⨁◯◯◯^b^^,^^c^	1.06 [0.88, 1.28]	⨁◯◯◯	1.05 [0.96, 1.14]	⨁◯◯◯^e^
↓ SFA	4	70	0.99 [0.93, 1.05]	⨁⨁⨁◯^a^	0.99 [0.90, 1.09]	⨁⨁⨁◯	0.99 [0.94, 1.04]	⨁⨁◯◯^e^
↓ TFA	1	99	0.95 [0.55, 1.66]	⨁⨁◯◯^b^	87 [0.31, 24,508]	⨁⨁◯◯	0.99 [0.57, 1.73]	⨁◯◯◯^f^
↓ CHO	14	95	1.00 [0.97, 1.03]	⨁⨁◯◯^a^^,^^c^	1.01 [0.88, 1.17]	⨁⨁⨁◯	1.00 [0.97, 1.03]	⨁⨁◯◯^e^

⨁⨁⨁⨁ High; ⨁⨁⨁◯ Moderate; ⨁⨁◯◯ Low; ⨁◯◯◯ Very low.

95% CI 95% confidence interval; AP animal protein; CHO carbohydrates; GRADE Grading of Recommendations Assessment, Development and Evaluations; HR hazard ratio; MUFA monounsaturated fatty acids; PP plant protein; PRO protein, PUFA polyunsaturated fatty acids; RoB risk of bias; SFA saturated fatty acids; TFA trans-fatty acids.

a Downgraded by 1 level for RoB: less than 2/3 of the studies (and their contributing weight) were rated with a low RoB, and less than 2/3 of the studies were rated with a high RoB, OR more than 2/3 of the studies (and their contributing weight) were rated with a high RoB, but the effect estimate in the subgroup analysis, excluding studies with a high RoB, was robust.

b Downgraded by 2 levels for RoB: More than 2/3 of the studies (and their contributing weight) were rated with a high RoB. No subgroup analysis for RoB could be conducted to test to robustness of the effect estimates.

c Downgraded by 1 level for inconsistency: The point estimates differ substantially between primary studies, and the corresponding 95% CI overlap only minimally or not at all. We found no clinical or methodological explanation for this inconsistency.

d Downgraded by 1 level for incoherence: The direct and indirect estimates differ beyond chance and this difference cannot be explained. Although the proportion of the indirect estimate was low, its value had an impact on the network estimate. The p-value for the comparison of the indirect and direct evidence is statistically significant.

e Downgraded by 1 level for imprecision: The 95% CI includes a RR/HR of 1 and the 95% CI is not narrow (maximal width of 0.05).

f Downgraded by 2 levels for imprecision: The 95% CI includes a RR/HR of 1 and the ratio of the upper to the lower CI bound is >3.

### Carbohydrate-specific networks

The evidence is very uncertain about the association of replacing 5% of energy from low-with high-quality carbohydrates on all-cause mortality (HR: 0.97; 95% CI: 0.92, 1.02). Moreover, neither the replacement of PUFA, MUFA, SFA or protein with low- or high-quality carbohydrates was associated with mortality risk (very low certainty) (Fig. 2, Supplemental Tables S13 and S14).

### Heterogeneity

The heterogeneity standard deviation (tau, τ) was estimated at τ: 0.03 for network 1, τ: 0.09 for network 2, τ: 0.04 for network 3, τ: 0.1 for network 4, τ: 0.01 for network 5, τ: 0.05 for network 6, and τ: 0.05 for network 7. We investigated any differences using predefined study characteristics, although the heterogeneity was low overall.

### Incoherence

The side-splitting approach showed no substantial indication for statistical inconsistency in any of the networks, except for some comparisons in network 2 (PUFA vs. MUFA, PUFA vs. TFA, PUFA vs. protein, MUFA vs. TFA, SFA vs. PRO), network 4 (n-3 PUFA vs. n-6 PUFA, n-3 PUFA vs. carbohydrate, n-6 PUFA vs. SFA), network 6 (plant protein vs. PUFA, plant protein vs. MUFA, plant protein vs. SFA), and network 7 (high-quality carbohydrates vs. SFA) (Supplemental Tables S15–S21).

The design by treatment test showed no indication for inconsistency for network 1, 3, and 5; but for network 2, 4, 6, and 7 (p < 0.05) (Supplemental Table S22).

The detected statistical inconsistency in these networks is largely driven by the standardization to 5% energy substitution, especially for TFA in network 2 and n-3 PUFA in network 4. This procedure is however essential to hold the transitivity assumption. Since the direct evidence in these networks was always superior (median: 86% contribution of evidence), we did not downgrade for incoherence in any comparison (except for plant protein vs. MUFA) of the seven networks and assume that this statistical inconsistency is not of clinical relevance.

### P-scores

Fat (P-score: 0.95) had the highest P-score in network 1, PUFA (P-score: 0.99) followed by MUFA (P-score: 0.75) for network 2, plant-MUFA (P-score: 1.00) for network 3, n-3 PUFA (P-score: 0.96) followed by n-6 PUFA (P-score: 0.72) for network 4, protein (P-score: 0.97) followed by plant fat (P-score: 0.68) for network 5, plant protein (P-score: 0.94) for network 6, and MUFA (P-score: 0.81) followed by high-quality carbohydrates (P-score: 0.68) in network 7 (Supplemental Tables S23–S29).

### Subgroup and sensitivity analyses

Due to the minimum requirement of 10 cohort studies, subgroup and sensitivity analysis could be conducted only in few networks, and the subgroup analysis for sex was not possible.

The subgroup analyses for geographical location was conducted for network 1, 2, 4, and was in agreement with the main analysis (Supplemental Tables S30–S32). This also applies for the subgroup analysis including studies with a single dietary assessment, which was performed for network 1 and 4 (Supplemental Tables S33 and S34). All main findings were confirmed in the sensitivity analyses excluding high RoB studies and when excluding cohort studies that had high relative residual effects (Supplemental Tables S35–S40).

## Discussion

This is the first systematic review and NMA that summarized the associations between the substitution of a wide range of (macro)nutrients and all-cause mortality by including 1,737,644 participants with 395,491 mortality events. Our results suggest that replacing 5% of total energy from carbohydrates with PUFA, n-6 PUFA, n-3 PUFA or plant-MUFA, is inversely associated with mortality risk based on moderate certainty of evidence. Moreover, replacing 5% of energy from SFA and TFA, with PUFA, MUFA or plant-MUFA, and higher intakes of n-6 or n-3 PUFA at the expenses of SFA was associated with lower mortality risk. Moderate certainty of evidence for lower mortality risk was also found when 5% of animal-MUFA was replaced with plant-MUFA, and when replacing animal protein, and SFA with plant protein. The certainty of evidence for the replacement of low-with high-quality carbohydrates was rated as very low.

Our results on macronutrient intake suggest a slight benefit of a 5% replacement of carbohydrates with fat, whereas no association was observed for exchanging carbohydrates with protein, and fat with protein. Due to the lack of a systematic review on substitution effects, our results cannot be directly compared with other studies. However, recent findings from systematic reviews show that a 5% higher intake of carbohydrate and fat and a 3% higher protein intake (without specifying the type of replaced macronutrient) was not associated with all-cause mortality,^13^^,^^74^, ^75^, ^76^ which is not fully supported by our results.

With regard to fat quality, our results are mainly in agreement with a WHO evidence-report of pairwise meta-analyses on the health-impact of SFA and TFA, indicating that replacement of 5% SFA by PUFA, MUFA, and carbohydrates was inversely related to mortality risk, although we did not observe a lower mortality risk when SFA was replaced by carbohydrates. On the contrary, in the WHO report, no association was observed when replacing TFA by SFA,^77^ whereas our NMA suggests a lower mortality risk by 13%. Moreover, we observed that replacement of 5% TFA by PUFA, MUFA, carbohydrates and protein was associated with lower mortality risk, associations that were not investigated in the WHO report. Similarly, replacement of carbohydrates by MUFA and PUFA has so far only been investigated in our NMA, and both, MUFA and PUFA performed better than carbohydrates, favouring PUFA.

The beneficial effects of MUFA compared to SFA, TFA and carbohydrates were attributed to plant-MUFAs, whereas replacing plant-MUFA and carbohydrates with animal-MUFAs was associated with a higher mortality risk. No systematic review so far has investigated the association between animal- or plant-based MUFAs and mortality.^78^ Predominant food sources of plant-MUFAs include olive oil and nuts.^79^ A systematic review of 30 RCTs has shown beneficial effects of virgin olive oil on inflammatory markers such as C-reactive protein and interleukin-6, as well as endothelial function.^80^ The detrimental association of animal-MUFAs on all-cause mortality, may be attributed to the high content of SFA in animal-MUFA rich foods like processed and unprocessed red meat and dairy products.^48^

Compared to carbohydrates, both n-6 and n-3 PUFA performed better. The beneficial associations of n-6 and n-3 PUFA were also shown in comparison with SFA. Our findings are in line with a systematic review of cohort studies, which has shown that a 5% increase in MUFA and PUFA was inversely associated with mortality, whereas SFA and TFA intake was associated with higher mortality risk.^74^ The risk of all-cause mortality was significantly increased up to 11% of the energy from SFA intake, with a tendency to plateau at higher percentage. Moreover, in systematic reviews of observational studies higher intakes of EPA/DHA were inversely associated with mortality risk.^81^

The available data from RCTs on mortality as an outcome is scare. A Cochrane review by Hooper and colleagues^82^ found no effect of reducing SFA on all-cause mortality, and a further evidence synthesis did not show any association between higher PUFA intake and risk of all-cause mortality,^81^ although protective effects on CVD were detected. The evidence on intermediate risk factors (such as atherogenic lipoproteins, glycaemia measures, and blood pressure) from RCTs is abundant and aligns with our findings. A systematic review of RCTs showed that isocaloric substitution of carbohydrates by SFA raises total cholesterol, low density lipoprotein cholesterol (LDL-C), high density lipoprotein (HDL-C) and reduces triglycerides, whereas substitution of carbohydrates by MUFA and PUFA reduces total cholesterol, LDL-C, triglycerides, and increases HDL-C.^83^ As compared with an isoenergetic intake of MUFA and PUFA, the consumption of industrial TFA raises levels of total cholesterol, LDL-C, triglycerides, reduces levels of HDL-C, whereas for ruminant TFAs, the number of studies is still low.^84^ Replacement of industrial TFA by SFA led to increased levels of total, LDL-C and HDL-C.^85^ The concentration of LDL particles represents a valuable indicator for the development of arteriosclerosis.^86^ Considering the potential mechanisms of action, reducing SFA (e.g., via replacement with PUFA/MUFA) increased the expression of LDL receptor. This may contribute to an improved clearance of LDL particles by the liver. Some studies have shown that this effect of fats can be mediated by influencing the transcription factor sterol-response element binding protein 2.^87^^,^^88^ The detrimental effects of TFA might be explained either by compromising the LDL-Apo B metabolism^89^ or via activation of plasma lipid transfer protein.^90^ Replacing carbohydrates or SFA with MUFAs or PUFAs also improves biomarkers of glucose metabolism such as HbA1c and HOMA-IR.^91^ In turn, elevated biomarkers of lipid and glucose metabolism are associated with greater mortality risk according to recent Mendelian Randomization studies, thus supporting that our NMA shows causal associations.^92^^,^^93^

Regarding protein quality, our findings align with a systematic review of nine prospective cohort studies found an inverse association for all-cause mortality, when replacing animal protein with plant protein, in four out of five studies.^94^ An umbrella review by Lv and colleagues^75^ reported no association between a 3% increase in animal protein and all-cause mortality, whereas plant protein was inversely related to overall mortality.^75^ We report for the first time a beneficial association of plant protein compared to carbohydrates and SFA. Among the most important food groups for the intake of plant-based proteins are legumes and nuts. Both are characterized by a high content in fibre, unsaturated fatty acids, folic acid, secondary plant metabolites and other antioxidants and are associated with an improved serum lipid profile and a reduction in cardiovascular risk.^95^, ^96^, ^97^, ^98^ Potential disadvantages of plant protein sources are e.g., a lower bioavailability and an unfavourable amino acid profile.^99^ However, a focus on plant-based protein sources does not imply complete avoidance of animal-based proteins, e.g., from fish and seafood in order to obtain a high protein quality with sufficient amounts of essential amino acids.

Although it is well established that carbohydrate quality is more important than quantity, in our NMA, we did not observe an association when replacing low-quality with high-quality carbohydrates on mortality. Similar to our findings, a dose–response meta-analysis found no association between glycaemic index/load and mortality.^100^ However, findings from prospective studies and RCTs associated with relatively high intakes of dietary fibre and whole grains were complementary, and dose–response evidence indicates that the relationships with several non-communicable diseases could be causal.^101^ As no substitution data on carbohydrate types such a glucose, fructose, etc were available, it was not possible to conduct the additional a-priori planned NMA.

The Acceptable Macronutrient Distribution Range for carbohydrates is set at 45–65% of total energy, at 10–35% for protein intake, and at 20–35% for total fat intake.^6^ A recent scoping review of guidelines on dietary fat commissioned by the International Union of Nutritional Sciences,^102^ highlighted the range of intake recommendations for MUFA 10–25%, for PUFA 6–11%, for SFA 7–11%, and for industrial TFA 0–2%.

Our findings confirm recommendations by the WHO to replace TFA and SFA with plant-MUFAs and PUFAs, but somewhat question the recommendation to limit total fat intake to ≤30%, and aim for a carbohydrate intake between 40 and 70%, whereas, our findings indicate that an increased carbohydrate recommendation should be seen with caution, as we showed an association between a 5% substitution of carbohydrates with fat, especially vegetable fats such as plant-MUFAs, PUFAs (n-6 and n-3), and plant protein and lower mortality. The intake of animal-MUFA should be limited, as compared to carbohydrates and plant-MUFA as it seems to be associated with an increased mortality risk. Although, we did not observe an association between low-quality compared to high-quality carbohydrates, carbohydrates from foods containing naturally occurring dietary fibre (e.g., whole grains, vegetables, fruits, pulses) are associated with additional health benefits based on other systematic reviews.^101^ Moreover, our findings are in line with the current U.S. Department of Agriculture dietary guidelines, that replacing animal with plant protein is deemed beneficial. This is particularly important as protein intake, according to the Acceptable Range of Macronutrient Distribution, should be between 10% and 35% of energy, which is a considerable amount.

Our systematic review and NMA enclosing 39 studies and 297 (macro)nutrient comparisons has several strengths, including its novelty and impact, the stringent methodology such as the a-priori published study protocol, extensive search and the screening, data extraction, RoB assessment carried out by two independent authors, the methodology of NMA first time used in this setting for advances in statistical analyses, and GRADE certainty of evidence assessment. The usage of substitution analyses is an additional strength, due to the better health-interpretation of (macro)nutrients. Furthermore, if effect sizes were only available per quantiles/unit of intake/exchange for a primary study, we estimated the linear association per 5% of total energy exchange, this allowed us to derive additional data on substitution analyses. By combining direct and indirect evidence to form the network estimates, we were able to increase the precision of our results. Furthermore, using NMA allowed us to rank all nutrient substitutions within their respective networks from best to worst. However, our work also has several limitations. First of all, we are not able to assume causality for the observed associations considering the observational nature of the included cohort studies. Residual and unmeasured confounding cannot be excluded despite the adjustment for important personal (e.g., age, sex) and lifestyle factors (e.g., physical activity, smoking) by 30 cohort studies.^12^^,^^41^^,^^42^^,^^45^, ^46^, ^47^, ^48^^,^^50^, ^51^, ^52^, ^53^^,^^57^, ^58^, ^59^, ^60^, ^61^^,^^63^, ^64^, ^65^, ^66^, ^67^, ^68^, ^69^, ^70^, ^71^, ^72^, ^73^ Nonetheless, the estimates provided in our analyses are conservative, since we analysed the most-adjusted risk estimates available from each cohort study, many of which were adjusted for known mediators for all-cause mortality (e.g., hypertension, hypercholesterolemia). It may be that more judicious adjustments of confounders rather than mediators in future cohort studies would better reflect the relationship between nutrients and health outcomes. Second, the different tools to assess dietary intakes in the included studies are based on self-reporting and therefore subject to measurement error.^103^ The relative paucity of data available precluded higher certainty of evidence relating to (macro)nutrients and mortality. Third, the findings on TFA need to be interpreted with caution, since several cohort studies with dietary assessment in early 2000 were included, and since then TFA levels of intake have decreased substantially.^104^ Nevertheless, it seems possible, that risk estimates associated with TFA may represent an underestimate of the true effect of sustained intakes, since in the countries from which most data were derived regulatory measures have resulted in a reduction of TFA intake, to the extent that baseline measurements from cohorts do not reflect intakes over the period of observation. Moreover, due to the limited availability of data, we were not able to differentiate between industrial and ruminant TFA. Detailed information on ruminant TFA was available in one cohort study only.^54^ Fourth, the carbohydrate network needs to be interpreted with caution, since we used carbohydrates categorized as “complex”, “high-quality” to form the node “high-quality carbohydrates/polysaccharides”. However, “starch” could also include carbohydrates of lower quality. Sixth, all analyses were based on a linear assumption. Although it was shown in previous systematic reviews that non-linearity was seldom for macronutrients,^74^^,^^105^ it cannot be ruled out.

In conclusion, our results are of practical importance for many professionals working in the field of public health, and can inform upcoming dietary guidelines. Our data showed a beneficial association on mortality risk by increasing PUFA (both n-3 and n-6) and (plant-) MUFA at the expenses of carbohydrates, SFA and TFA, and when replacing animal protein and animal-MUFA with plant-based sources of protein and fat (MUFA). These findings reinforce the importance of plant-based dietary guidelines. However, it is worth noting that these conclusions may diverge from current WHO recommendations on a healthy diet, particularly regarding the limitation of fat intake to no more than 30% of total caloric intake. In future studies, specific fatty acids, amino acids types, and a more differentiated approach for different carbohydrates could also be investigated in addition to non-communicable disease outcomes.

## Contributors

LS, AN, TP, SW designed the research. EK, ES, JB, JS, JM, LS, MN, SS, SW, and WB conducted the literature search and literature screening. LS and SW extracted the data. EK, LG, JS, and SW assessed the risk of bias of the included publications. LS and SW evaluated the certainty of evidence. AN, TP, SW, LS analysed the data and wrote the first draft of the paper. All authors (AN, EK, ES, GH, JB, JM, JJM, JS, LG, MG, LS, MN, SL, SS, SW, TK, TP, and WB) interpreted the data, read the manuscript, and approved the final version. LS and SW are the guarantors. The corresponding author attests that all listed authors meet authorship criteria and that no others meeting the criteria were omitted. Transparency: The lead authors (the manuscript’s guarantors) affirm that the manuscript is an honest, accurate, and transparent account of the study being reported; that no important aspects of the study have been omitted; and that any discrepancies from the study as planned have been explained.

All authors had full access to the study data and had final responsibility for the decision to submit for publication. LS and SW had direct access to and verified the underlying data reported in the manuscript.

## Data sharing statement

Data were extracted from published prospective observational studies. Results of unpublished data can be found in the Supplemental. All data, code for statistical analysis and output can be found under the following link: https://zenodo.org/doi/10.5281/zenodo.10978842.

## Declaration of interests

All authors have completed the ICMJE uniform disclosure form at www.icmje.org/disclosure-of-interest/ and declare to have no conflict of interest.

## References

[1] GBD 2017 Diet Collaborators (2019). Health effects of dietary risks in 195 countries, 1990-2017: a systematic analysis for the Global Burden of Disease Study 2017. Lancet.

[2] U.S. Department of Health and Human Services (1980).

[3] U.S. Department of Health and Human Services, U.S. Department of Agriculture (2005).

[4] (2023). Saturated fatty acid and trans-fatty acid intake for adults and children: WHO guideline.

[5] Wu G. (2016). Dietary protein intake and human health. Food Funct.

[6] Trumbo P., Schlicker S., Yates A.A., Poos M. (2002). Dietary reference intakes for energy, carbohydrate, fiber, fat, fatty acids, cholesterol, protein and amino acids.(Commentary). J Am Diet Assoc.

[7] Schulze M.B., Martínez-González M.A., Fung T.T., Lichtenstein A.H., Forouhi N.G. (2018). Food based dietary patterns and chronic disease prevention. BMJ.

[8] Song M., Giovannucci E. (2018). Substitution analysis in nutritional epidemiology: proceed with caution. Eur J Epidemiol.

[9] Schwingshackl L., Schünemann H.J., Meerpohl J.J. (2021). Improving the trustworthiness of findings from nutrition evidence syntheses: assessing risk of bias and rating the certainty of evidence. Eur J Nutr.

[10] Schwingshackl L., Schwarzer G., Rücker G., Meerpohl J.J. (2019). Perspective: network meta-analysis reaches nutrition research: current status, scientific concepts, and future directions. Adv Nutr.

[11] de Souza R.J., Mente A., Maroleanu A. (2015). Intake of saturated and trans unsaturated fatty acids and risk of all cause mortality, cardiovascular disease, and type 2 diabetes: systematic review and meta-analysis of observational studies. BMJ.

[12] Chen Z., Glisic M., Song M. (2020). Dietary protein intake and all-cause and cause-specific mortality: results from the Rotterdam Study and a meta-analysis of prospective cohort studies. Eur J Epidemiol.

[13] Qin P., Huang C., Jiang B. (2023). Dietary carbohydrate quantity and quality and risk of cardiovascular disease, all-cause, cardiovascular and cancer mortality: a systematic review and meta-analysis. Clin Nutr.

[14] Hutton B., Salanti G., Caldwell D.M. (2015). The PRISMA extension statement for reporting of systematic reviews incorporating network meta-analyses of health care interventions: checklist and explanations. Ann Intern Med.

[15] Rethlefsen M.L., Kirtley S., Waffenschmidt S. (2021). PRISMA-S: an extension to the PRISMA statement for reporting literature searches in systematic reviews. Syst Rev.

[16] Willett W.C. (2012). Dietary fats and coronary heart disease. J Intern Med.

[17] Clark J., Glasziou P., Del Mar C., Bannach-Brown A., Stehlik P., Scott A. (2020). A full systematic review was completed in 2 weeks using automation tools: a case study. J Clin Epidemiol.

[18] Innovation V.H. Covidence systematic review software Melbourne, Australia. http://www.covidence.org.

[19] Rohatki A. (2022). https://automeris.io/WebPlotDigitizer.

[20] Higgins J.P.T., Morgan R.L., Rooney A.A. (2024). A tool to assess risk of bias in non-randomized follow-up studies of exposure effects (ROBINS-E). Environ Int.

[21] McGuinness L.A., Higgins J.P.T. (2020). Risk-of-bias VISualization (robvis): an R package and Shiny web app for visualizing risk-of-bias assessments. Res Synth Methods.

[22] Freedman L.S., Schatzkin A., Midthune D., Kipnis V. (2011). Dealing with dietary measurement error in nutritional cohort studies. J Natl Cancer Inst.

[23] Salanti G. (2012). Indirect and mixed-treatment comparison, network, or multiple-treatments meta-analysis: many names, many benefits, many concerns for the next generation evidence synthesis tool. Res Synth Methods.

[24] Greenland S., Longnecker M.P. (1992). Methods for trend estimation from summarized dose-response data, with applications to meta-analysis. Am J Epidemiol.

[25] Rücker G. (2012). Network meta-analysis, electrical networks and graph theory. Res Synth Methods.

[26] Jackson D., White I.R., Riley R.D. (2012). Quantifying the impact of between-study heterogeneity in multivariate meta-analyses. Stat Med.

[27] Balduzzi S., Rücker G., Nikolakopoulou A. (2023). netmeta: an R package for network meta-analysis using frequentist methods. J Stat Softw.

[28] Rücker G., Schwarzer G. (2015). Ranking treatments in frequentist network meta-analysis works without resampling methods. BMC Med Res Methodol.

[29] Jackson D., Barrett J.K., Rice S., White I.R., Higgins J.P. (2014). A design-by-treatment interaction model for network meta-analysis with random inconsistency effects. Stat Med.

[30] Dias S., Welton N.J., Caldwell D.M., Ades A.E. (2010). Checking consistency in mixed treatment comparison meta-analysis. Stat Med.

[31] Egger M., Davey Smith G., Schneider M., Minder C. (1997). Bias in meta-analysis detected by a simple, graphical test. BMJ.

[32] GDT G (2024).

[33] Brignardello-Petersen R., Bonner A., Alexander P.E. (2018). Advances in the GRADE approach to rate the certainty in estimates from a network meta-analysis. J Clin Epidemiol.

[34] Schünemann H.J., Cuello C., Akl E.A. (2019). GRADE guidelines: 18. How ROBINS-I and other tools to assess risk of bias in nonrandomized studies should be used to rate the certainty of a body of evidence. J Clin Epidemiol.

[35] Murad M.H., Verbeek J., Schwingshackl L. (2023). GRADE guidance 38: updated guidance for rating up certainty of evidence due to a dose-response gradient. J Clin Epidemiol.

[36] Guyatt G.H., Oxman A.D., Sultan S. (2011). GRADE guidelines: 9. Rating up the quality of evidence. J Clin Epidemiol.

[37] Yepes-Nuñez J.J., Li S.A., Guyatt G. (2019). Development of the summary of findings table for network meta-analysis. J Clin Epidemiol.

[38] Balshem H., Helfand M., Schünemann H.J. (2011). GRADE guidelines: 3. Rating the quality of evidence. J Clin Epidemiol.

[39] Argos M., Melkonian S., Parvez F. (2013). A population-based prospective study of energy-providing nutrients in relation to all-cause cancer mortality and cancers of digestive organs mortality. Int J Cancer.

[40] Bajracharya R., Katzke V., Mukama T., Kaaks R. (2023). Effect of iso-caloric substitution of animal protein for other macro nutrients on risk of overall, cardiovascular and cancer mortality: prospective evaluation in EPIC-heidelberg cohort and systematic review. Nutrients.

[41] Budhathoki S., Sawada N., Iwasaki M. (2019). Association of animal and plant protein intake with all-cause and cause-specific mortality in a Japanese cohort. JAMA Intern Med.

[42] Das A., Cumming R., Naganathan V. (2022). Associations between dietary intake of total protein and sources of protein (plant vs. animal) and risk of all-cause and cause-specific mortality in older Australian men: the Concord Health and Ageing in Men Project. J Hum Nutr Diet.

[43] Dehghan M., Mente A., Zhang X. (2017). Associations of fats and carbohydrate intake with cardiovascular disease and mortality in 18 countries from five continents (PURE): a prospective cohort study. Lancet.

[44] Dominguez L.J., Bes-Rastrollo M., Basterra-Gortari F.J., Gea A., Barbagallo M., Martínez-González M.A. (2018). Should we recommend reductions in saturated fat intake or in red/processed meat consumption? The SUN prospective cohort study. Clin Nutr.

[45] Fontana L., Sieri S., Ricceri F. (2021). Dietary intake of animal and plant proteins and risk of all cause and cause-specific mortality: the Epic-Italy cohort. Nutr Healthy Aging.

[46] Fridén M., Olsson E., Lind L., Rosqvist F., Risérus U. (2023). Substitution analyses of foods with varying fat quality and the associations with all-cause mortality and impact of the FADS-1 genotype in elderly men. Eur J Nutr.

[47] Guasch-Ferré M., Babio N., Martínez-González M.A. (2015). Dietary fat intake and risk of cardiovascular disease and all-cause mortality in a population at high risk of cardiovascular disease. Am J Clin Nutr.

[48] Guasch-Ferré M., Zong G., Willett W.C. (2019). Associations of monounsaturated fatty acids from plant and animal sources with total and cause-specific mortality in two US prospective cohort studies. Circ Res.

[49] Hernández-Alonso P., Salas-Salvadó J., Ruiz-Canela M. (2016). High dietary protein intake is associated with an increased body weight and total death risk. Clin Nutr.

[50] Ho F.K., Gray S.R., Welsh P. (2020). Associations of fat and carbohydrate intake with cardiovascular disease and mortality: prospective cohort study of UK Biobank participants. BMJ.

[51] Huang J., Liao L.M., Weinstein S.J., Sinha R., Graubard B.I., Albanes D. (2020). Association between plant and animal protein intake and overall and cause-specific mortality. JAMA Intern Med.

[52] Kelemen L.E., Kushi L.H., Jacobs D.R., Cerhan J.R. (2005). Associations of dietary protein with disease and mortality in a prospective study of postmenopausal women. Am J Epidemiol.

[53] Kwon Y.-J., Hyun D.S., Koh S.B., Lee J.W. (2021). Differential relationship between dietary fat and cholesterol on total mortality in Korean population cohorts. J Intern Med.

[54] Laake I., Pedersen J.I., Selmer R. (2012). A prospective study of intake of trans-fatty acids from ruminant fat, partially hydrogenated vegetable oils, and marine oils and mortality from CVD. Br J Nutr.

[55] Laguna J.C., Alegret M., Cofán M. (2021). Simple sugar intake and cancer incidence, cancer mortality and all-cause mortality: a cohort study from the PREDIMED trial. Clin Nutr.

[56] Levine M.E., Suarez J.A., Brandhorst S. (2014). Low protein intake is associated with a major reduction in IGF-1, cancer, and overall mortality in the 65 and younger but not older population. Cell Metab.

[57] Li L., Shan Z., Wan Z. (2022). Associations of lower-carbohydrate and lower-fat diets with mortality among people with prediabetes. Am J Clin Nutr.

[58] Mao L., Zhang Y., Wang W., Zhuang P., Wu F., Jiao J. (2020). Plant-sourced and animal-sourced monounsaturated fatty acid intakes in relation to mortality: a prospective nationwide cohort study. Eur J Nutr.

[59] Meroño T., Zamora-Ros R., Hidalgo-Liberona N. (2022). Animal protein intake is inversely associated with mortality in older adults: the InCHIANTI study. J Gerontol a Biol Sci Med Sci.

[60] Nagata C., Nakamura K., Wada K. (2012). Total fat intake is associated with decreased mortality in Japanese men but not in women. J Nutr.

[61] Ricci C., Baumgartner J., Zec M., Kruger H.S., Smuts C.M. (2018). Type of dietary fat intakes in relation to all-cause and cause-specific mortality in US adults: an iso-energetic substitution analysis from the American National Health and Nutrition Examination Survey linked to the US mortality registry. Br J Nutr.

[62] Shan Z., Haslam D.E., Rehm C.D. (2020). Abstract P510: association of animal and plant protein intake with mortality among us adults: a prospective cohort study. Circulation.

[63] Song M., Fung T.T., Hu F.B. (2016). Association of animal and plant protein intake with all-cause and cause-specific mortality. JAMA Intern Med.

[64] Sun Y., Liu B., Snetselaar L.G. (2021). Association of major dietary protein sources with all-cause and cause-specific mortality: prospective cohort study. J Am Heart Assoc.

[65] Virtanen H.E.K., Voutilainen S., Koskinen T.T. (2019). Dietary proteins and protein sources and risk of death: the kuopio ischaemic heart disease risk factor study. Am J Clin Nutr.

[66] Wakai K., Naito M., Date C., Iso H., Tamakoshi A. (2014). Dietary intakes of fat and total mortality among Japanese populations with a low fat intake: the Japan Collaborative Cohort (JACC) Study. Nutr Metab.

[67] Wang D.D., Li Y., Chiuve S.E. (2016). Association of specific dietary fats with total and cause-specific mortality. JAMA Intern Med.

[68] Wu F., Mao L., Zhuang P., Chen X., Jiao J., Zhang Y. (2020). Plant-sourced cooking oil consumption is associated with lower total mortality in a longitudinal nationwide cohort study. Clin Nutr.

[69] Zeng X., Li X., Zhang Z. (2022). A prospective study of carbohydrate intake and risk of all-cause and specific-cause mortality. Eur J Nutr.

[70] Zhao Y., Li Y., Wang W. (2023). Low-carbohydrate diets, low-fat diets, and mortality in middle-aged and older people: a prospective cohort study. J Intern Med.

[71] Zhou C., Yang S., Zhang Y. (2022). Relations of variety and quantity of dietary proteins intake from different sources with mortality risk: a nationwide population-based cohort. J Nutr Health Aging.

[72] Zhuang P., Zhang Y., He W. (2019). Dietary fats in relation to total and cause-specific mortality in a prospective cohort of 521 120 individuals with 16 Years of follow-up. Circ Res.

[73] Zhuang P., Cheng L., Wang J., Zhang Y., Jiao J. (2019). Saturated fatty acid intake is associated with total mortality in a nationwide cohort study. J Nutr.

[74] Kim Y., Je Y., Giovannucci E.L. (2021). Association between dietary fat intake and mortality from all-causes, cardiovascular disease, and cancer: a systematic review and meta-analysis of prospective cohort studies. Clin Nutr ESPEN.

[75] Lv J.L., Wu Q.J., Li X.Y. (2022). Dietary protein and multiple health outcomes: an umbrella review of systematic reviews and meta-analyses of observational studies. Clin Nutr.

[76] Liu Y.S., Wu Q.J., Lv J.L. (2021). Dietary carbohydrate and diverse health outcomes: umbrella review of 30 systematic reviews and meta-analyses of 281 observational studies. Front Nutr.

[77] Reynolds A.N., Hodson L., De Souza R., Tran Diep Pham H., Vlietstra L., Mann J. (2022).

[78] Lotfi K., Salari-Moghaddam A., Yousefinia M., Larijani B., Esmaillzadeh A. (2021). Dietary intakes of monounsaturated fatty acids and risk of mortality from all causes, cardiovascular disease and cancer: a systematic review and dose-response meta-analysis of prospective cohort studies. Ageing Res Rev.

[79] Linseisen J., Welch A.A., Ocké M. (2009). Dietary fat intake in the European Prospective Investigation into Cancer and Nutrition: results from the 24-h dietary recalls. Eur J Clin Nutr.

[80] Schwingshackl L., Christoph M., Hoffmann G. (2015). Effects of olive oil on markers of inflammation and endothelial function—a systematic review and meta-analysis. Nutrients.

[81] Schwingshackl L., Zähringer J., Beyerbach J. (2021). Total dietary fat intake, fat quality, and health outcomes: a scoping review of systematic reviews of prospective studies. Ann Nutr Metab.

[82] Hooper L., Martin N., Jimoh O.F., Kirk C., Foster E., Abdelhamid A.S. (2020). Reduction in saturated fat intake for cardiovascular disease. Cochrane Database Syst Rev.

[83] Mensink R. (2016).

[84] Mozaffarian D., Katan M.B., Ascherio A., Stampfer M.J., Willett W.C. (2006). Trans fatty acids and cardiovascular disease. N Engl J Med.

[85] WHO Brouwer IA. (2016).

[86] Borén J., Chapman M.J., Krauss R.M. (2020). Low-density lipoproteins cause atherosclerotic cardiovascular disease: pathophysiological, genetic, and therapeutic insights: a consensus statement from the European Atherosclerosis Society Consensus Panel. Eur Heart J.

[87] Fernandez M.L., West K.L. (2005). Mechanisms by which dietary fatty acids modulate plasma lipids. J Nutr.

[88] Horton J.D., Goldstein J.L., Brown M.S. (2002). SREBPs: activators of the complete program of cholesterol and fatty acid synthesis in the liver. J Clin Invest.

[89] Matthan N.R., Welty F.K., Barrett P.H. (2004). Dietary hydrogenated fat increases high-density lipoprotein apoA-I catabolism and decreases low-density lipoprotein apoB-100 catabolism in hypercholesterolemic women. Arterioscler Thromb Vasc Biol.

[90] van Tol A., Zock P.L., van Gent T., Scheek L.M., Katan M.B. (1995). Dietary trans fatty acids increase serum cholesterylester transfer protein activity in man. Atherosclerosis.

[91] Imamura F., Micha R., Wu J.H.Y. (2016). Effects of saturated fat, polyunsaturated fat, monounsaturated fat, and carbohydrate on glucose-insulin homeostasis: a systematic review and meta-analysis of randomised controlled feeding trials. PLoS Med.

[92] Yang G., Mason A.M., Wood A.M., Schooling C.M., Burgess S. (2024). Dose-response associations of lipid traits with coronary artery disease and mortality. JAMA Netw Open.

[93] Yang G., Au Yeung S.L., Schooling C.M. (2022). Sex differences in the association of fasting glucose with HbA1c, and their consequences for mortality: a Mendelian randomization study. EBioMedicine.

[94] Zheng J., Zhu T., Yang G. (2022). The isocaloric substitution of plant-based and animal-based protein in relation to aging-related health outcomes: a systematic review. Nutrients.

[95] Afshin A., Micha R., Khatibzadeh S., Mozaffarian D. (2014). Consumption of nuts and legumes and risk of incident ischemic heart disease, stroke, and diabetes: a systematic review and meta-analysis. Am J Clin Nutr.

[96] Anderson J.W., Major A.W. (2002). Pulses and lipaemia, short- and long-term effect: potential in the prevention of cardiovascular disease. Br J Nutr.

[97] Mudryj A.N., Yu N., Aukema H.M. (2014). Nutritional and health benefits of pulses. Appl Physiol Nutr Metab.

[98] Visioli F., Poli A. (2020). Fatty acids and cardiovascular risk. Evidence, lack of evidence, and diligence. Nutrients.

[99] Berrazaga I., Micard V., Gueugneau M., Walrand S. (2019). The role of the anabolic properties of plant- versus animal-based protein sources in supporting muscle mass maintenance: a critical review. Nutrients.

[100] Shahdadian F., Saneei P., Milajerdi A., Esmaillzadeh A. (2019). Dietary glycemic index, glycemic load, and risk of mortality from all causes and cardiovascular diseases: a systematic review and dose-response meta-analysis of prospective cohort studies. Am J Clin Nutr.

[101] Reynolds A., Mann J., Cummings J., Winter N., Mete E., Te Morenga L. (2019). Carbohydrate quality and human health: a series of systematic reviews and meta-analyses. Lancet.

[102] Schwingshackl L., Zähringer J., Beyerbach J. (2021). A scoping review of current guidelines on dietary fat and fat quality. Ann Nutr Metab.

[103] Subar A.F., Freedman L.S., Tooze J.A. (2015). Addressing current criticism regarding the value of self-report dietary data. J Nutr.

[104] Wang S., Tian W., Liu Y. (2021). Temporal trend of circulating trans-fatty acids and risk of long-term mortality in general population. Clin Nutr.

[105] Ma Y., Zheng Z., Zhuang L. (2024). Dietary macronutrient intake and cardiovascular disease risk and mortality: a systematic review and dose-response meta-analysis of prospective cohort studies. Nutrients.

