# Supplementary material for: Association between substituting macronutrients and all-cause mortality: a network meta-analysis of prospective observational studies
Source: eClinicalMedicine. 2024 Sep 5;75:102807. doi: 10.1016/j.eclinm.2024.102807 (PMC11408053; doi:10.1016/j.eclinm.2024.102807)
Supplement: Supplemental Appendix 1–9, Figs. S1–S4, and Tables S1–S40 [file mmc1.pdf]

Substituting macronutrients and all-cause mortality: a network meta-analysis of prospective observational studies

Data supplement

Table of Contents

Supplemental figure 1: Risk of bias of each study for each domain and all-cause mortality ... 5

Supplemental figure 2: Funnel plots for the overall macronutrient network (5% isocaloric energy substitution) for the outcome all-cause mortality[a] ... 7

Supplemental figure 3: Funnel plots for the fatty acid expanded network (5% isocaloric energy substitution) for the outcome all-cause mortality[a] ... 8

Supplemental figure 4: Funnel plots for the protein-origin subnetwork (5% isocaloric energy substitution) for the outcome all-cause mortality[a] ... 10

Supplemental table 1: Eligibility criteria by the PICOS statement ... 11

Supplemental table 2: General characteristics of the included prospective observational studies for the outcome all-cause mortality ... 12

Supplemental table 3: (Macro)nutrient replacement including unit, effect estimates with 95% CI (as reported by authors), and adjustment factors of the included studies for the outcome all-cause mortality ... 21

Supplemental table 4: Reasons for exclusion of specific comparisons from the network meta-analysis ... 35

Supplemental table 5: League table for the overall macronutrient network (5% isocaloric energy substitution) for the outcome all-cause mortality ... 37

Supplemental table 6: GRADE assessment of all-cause mortality for the overall macronutrient network (5% isocaloric energy substitution); ... 38

Supplemental table 7: League table for the fatty acids expanded network (5% isocaloric energy substitution) for the outcome all-cause mortality ... 39

Supplemental table 8: League table for the MUFA-origin network (5% isocaloric energy substitution) for the outcome all-cause mortality ... 40

Supplemental table 9: League table for the PUFA-origin network (5% isocaloric energy substitution) for the outcome all-cause mortality ... 41

Supplemental table 10: League table for the fat-origin subnetwork (5% isocaloric energy substitution) for the outcome all-cause mortality ... 42

Supplemental table 11: GRADE assessment of all-cause mortality for the fat-origin subnetwork (5% isocaloric energy substitution); Number of participants (n= 258 291) and number of mortality events (n= 17 471) ... 43

Supplemental table 12: League table for the protein-origin subnetwork (5% isocaloric energy substitution) for the outcome all-cause mortality ... 44

Supplemental table 13: League table for the carbohydrate-origin subnetwork (5% isocaloric energy substitution) for the outcome all-cause mortality ... 45

Supplemental table 14: GRADE assessment of all-cause mortality for the carbohydrate-origin subnetwork (5% isocaloric energy substitution); ... 46

Supplemental table 15: SIDE splitting approach comparing direct and indirect evidence for the overall macronutrient network (5% isocaloric energy substitution) for the outcome all-cause mortality ... 48

Supplemental table 16: SIDE splitting approach comparing direct and indirect evidence for the fatty acids expanded network (5% isocaloric energy substitution) for the outcome all-cause mortality 49

Supplemental table 17: SIDE splitting approach comparing direct and indirect evidence for the MUFA-origin network (5% isocaloric energy substitution) for the outcome all-cause mortality........ 50

Supplemental table 18: SIDE splitting approach comparing direct and indirect evidence for the PUFA-origin network (5% isocaloric energy substitution) for the outcome all-cause mortality........ 51

Supplemental table 19: SIDE splitting approach comparing direct and indirect evidence for the fat-origin subnetwork (5% isocaloric energy substitution) for the outcome all-cause mortality........ 52

Supplemental table 20: SIDE splitting approach comparing direct and indirect evidence for the protein-origin subnetwork (5% isocaloric energy substitution) for the outcome all-cause mortality........ 53

Supplemental table 21: SIDE splitting approach comparing direct and indirect evidence for the carbohydrate-origin subnetwork (5% isocaloric energy substitution) for the outcome all-cause mortality 54

Supplemental table 22: Results of the design by treatment test (global test for statistical inconsistency in the network meta-analysis for the outcome all-cause mortality) 55

Supplemental table 23: P-scores for the overall macronutrient network (5% isocaloric energy substitution) for the outcome all-cause mortality 56

Supplemental table 24: P-scores for the fatty acids expanded network (5% isocaloric energy substitution) for the outcome all-cause mortality 56

Supplemental table 25: P-scores for the MUFA-origin network (5% isocaloric energy substitution) for the outcome all-cause mortality 56

Supplemental table 26: P-scores for the PUFA-origin network (5% isocaloric energy substitution) for the outcome all-cause mortality 57

Supplemental table 27: P-scores for the fat-origin subnetwork (5% isocaloric energy substitution) for the outcome all-cause mortality 57

Supplemental table 28: P-scores for the protein-origin subnetwork (5% isocaloric energy substitution) for the outcome all-cause mortality 57

Supplemental table 29: P-scores for the carbohydrate-origin subnetwork (5% isocaloric energy substitution) for the outcome all-cause mortality 58

Supplemental table 30: Subgroup analysis for the overall macronutrient network, including American cohort studies (5% isocaloric energy substitution); 59

Supplemental table 31: Subgroup analysis for the fatty acids expanded network, including American cohort studies (5% isocaloric energy substitution); 60

Supplemental table 32: Subgroup analysis for the protein-origin subnetwork, including American cohort studies (5% isocaloric energy substitution); 61

Supplemental table 33: Subgroup analysis for the overall macronutrient network, including studies with a single dietary assessment (5% isocaloric energy substitution); 61

Supplemental table 34: Subgroup analysis for the protein-origin subnetwork, including studies with a single dietary assessment (5% isocaloric energy substitution); 62
Supplemental table 35: Sensitivity analysis for the overall macronutrient network, excluding studies with a high risk of bias (5% isocaloric energy substitution); 63
Supplemental table 36: Sensitivity analysis for the fatty acids expanded network, excluding studies with a high risk of bias (5% isocaloric energy substitution); 64
Supplemental table 37: Sensitivity analysis for the protein-origin subnetwork, excluding studies with a high risk of bias (5% isocaloric energy substitution); 65
Supplemental table 38: Sensitivity analysis for the overall macronutrient network, excluding studies with high relative residual effects (5% isocaloric energy substitution); 65
Supplemental table 39: Sensitivity analysis for the fatty acids expanded network, excluding studies with high relative residual effects (5% isocaloric energy substitution); 66
Supplemental table 40: Sensitivity analysis for the protein origin network, excluding studies with high relative residual effects (5% isocaloric energy substitution); 67
Supplemental appendix 1: Search terms 68
Supplemental appendix 2: Detailed description and decision criteria for each domain in ROBINS-E assessment 71
Supplemental appendix 3: Handling of substitution analyses reported per kcal/d or g/d 78
Supplemental appendix 4: Handling of multiple publications on the same cohort: 79
Supplemental appendix 5: Handling of missing or inconsistent risk estimates and variances: 80
Supplemental appendix 6: Detailed description and decision criteria for each domain in the GRADE assessment 81
Supplemental appendix 7: List of excluded studies 85
Supplemental appendix 8: Analytical procedure for UK Biobank study 86
Supplemental appendix 9: Deviations from protocol 86
Supplemental References 87

Supplemental figure 1: Risk of bias of each study for each domain and all-cause mortality

	Risk of bias domains							
Study	D1	D2	D3	D4	D5	D6	D7	Overall
Argos 2013	X							X
Bajracharya 2023	X							X
Budhathoki 2019	-	-	+	+	-	+	+	-
Chen 2020	-	-	+	+	-	+	+	-
Das 2022	-	-	+	+	-	+	-	-
Dehghan 2017	X							X
Dominguez 2018	X							X
Fontana 2021	-	-	+	+	-	+	+	-
Friden 2023	-	-	+	+	+	+	+	-
Guasch-Ferre 2015	-	-	+	+	-	+	+	-
Guasch-Ferre 2019 (NHS)	-	-	+	+	-	+	+	-
Guasch-Ferre 2019 (HPFS)	-	-	+	+	-	+	+	-
Hernandez-Alonso 2016	X							X
Ho 2020	-	X	+	+	-	+	+	X
Huang 2020	-	-	+	+	+	+	-	-
Kelemen 2005	-	-	+	+	-	+	-	-
Kwon 2021	-	-	+	+	+	+	+	-
Laake 2012	X							X
Laguna 2021	X							X
Levine 2014	X							X

Supplemental figure 1 continued

	D1	D2	D3	D4	D5	D6	D7	Overall
Li 2022b	-	X	+	+	+	+	+	X
Mao 2020	-	-	+	+	-	+	+	-
Merono 2022	-	-	+	+	X	+	+	X
Nagata 2012	-	-	+	+	-	+	+	-
Ricci 2018	-	X	+	+	-	+	+	X
Song 2016 (NHS)	-	-	+	+	-	+	+	-
Song 2016 (HPFS)	-	-	+	+	-	+	+	-
Sun 2021	-	-	+	+	+	+	+	-
Virtanen 2019	-	-	+	+	+	+	+	-
Wang 2016 (NHS)	-	-	+	+	-	+	+	-
Wang 2016 (HPFS)	-	-	+	+	-	+	+	-
Wu 2020	-	-	+	+	-	+	+	-
Zeng 2022	-	X	+	+	-	+	+	X
Zhao 2023	-	-	+	+	+	+	+	-
Zhou 2022a	-	-	+	+	-	+	+	-
Zhuang 2019a	-	-	+	+	-	+	+	-
Zhuang 2019b	-	-	+	+	-	+	+	-
Wakai 2014	-	-	+	+	-	+	+	-

Domains:
Domain 1: Risk of bias due to confounding
Domain 2: Risk of bias arising from measurement of the exposures
Domain 3: Risk of bias in selection of participants into the study / into the analysis
Domain 4: Risk of bias due to post-exposure interventions
Domain 5: Risk of bias due to missing data
Domain 6: Risk of bias arising from measurement of the outcomes
Domain 7: Risk of bias in selection of the reported results

Judgement:

- + Low risk of bias
- - Some concerns
- X High risk of bias
- No judgement due to triage in first domain

D1-7 domain 1-7; HPFS Health Professional Follow up Study; NHS Nurses' Health Study

Supplemental figure 2: Funnel plots for the overall macronutrient network (5% isocaloric energy substitution) for the outcome all-cause mortality[a]

A

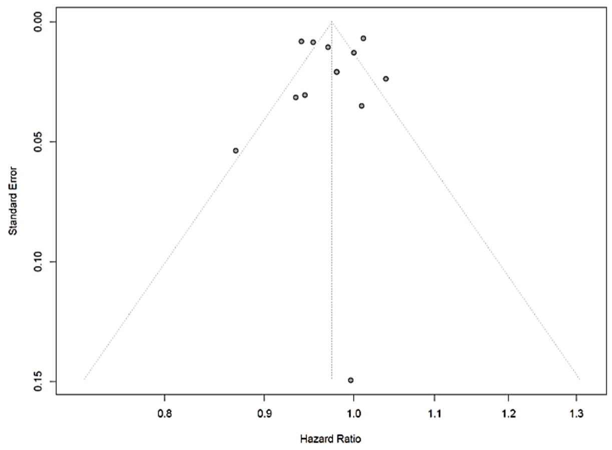

B

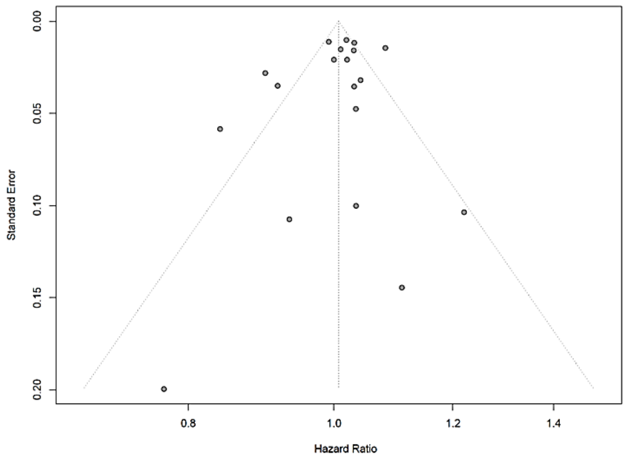

[a] At least 10 studies are required to conduct a funnel plot.

A: Funnel plot showing standard error against the hazard ratio for the substitution of carbohydrates with fat. P-value for Eggers linear regression test: p-value = 0.82

B: Funnel plot showing standard error against the hazard ratio for the substitution of protein with carbohydrates. P-value for Eggers linear regression test: p-value = 0.34

Results of Egger's regression test indicated no evidence of publication bias for all-cause mortality for any of the substitutions presented.

Supplemental figure 3: Funnel plots for the fatty acid expanded network (5% isocaloric energy substitution) for the outcome all-cause mortality[a]

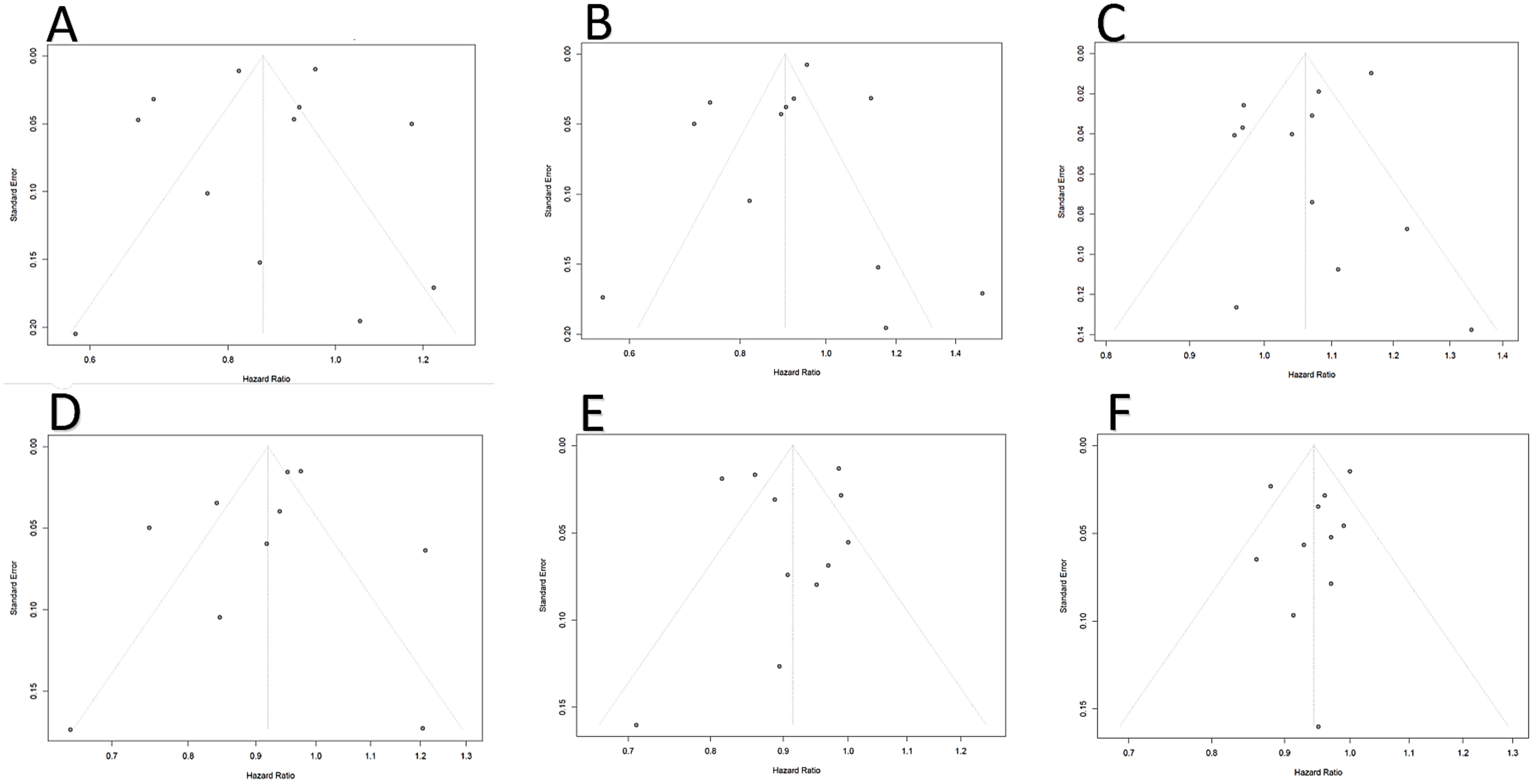

CHO carbohydrates; MUFA monounsaturated fatty acids; PUFA polyunsaturated fatty acids; SFA saturated fatty acids; TFA trans-fatty acids

[a] At least 10 studies are required to conduct a funnel plot.

A: Funnel plot showing standard error against the hazard ratio for the substitution of SFA with PUFA. P-value for Eggers linear regression test: p-value = 0.67

B: Funnel plot showing standard error against the hazard ratio for the substitution of CHO with PUFA. P-value for Eggers linear regression test: p-value = 0.46
C: Funnel plot showing standard error against the hazard ratio for the substitution of CHO with SFA. P-value for Eggers linear regression test: p-value = 0.11
D: Funnel plot showing standard error against the hazard ratio for the substitution of MUFA with PUFA. P-value for Eggers linear regression test: p-value = 0.44
E: Funnel plot showing standard error against the hazard ratio for the substitution of SFA with MUFA. P-value for Eggers linear regression test: p-value = 0.88
F: Funnel plot showing standard error against the hazard ratio for the substitution of CHO with MUFA. P-value for Eggers linear regression test: p-value = 0.33
Results of Egger's regression test indicated no evidence of publication bias for all-cause mortality for any of the substitutions presented.

Supplemental figure 4: Funnel plots for the protein-origin subnetwork (5% isocaloric energy substitution) for the outcome all-cause mortality[a]

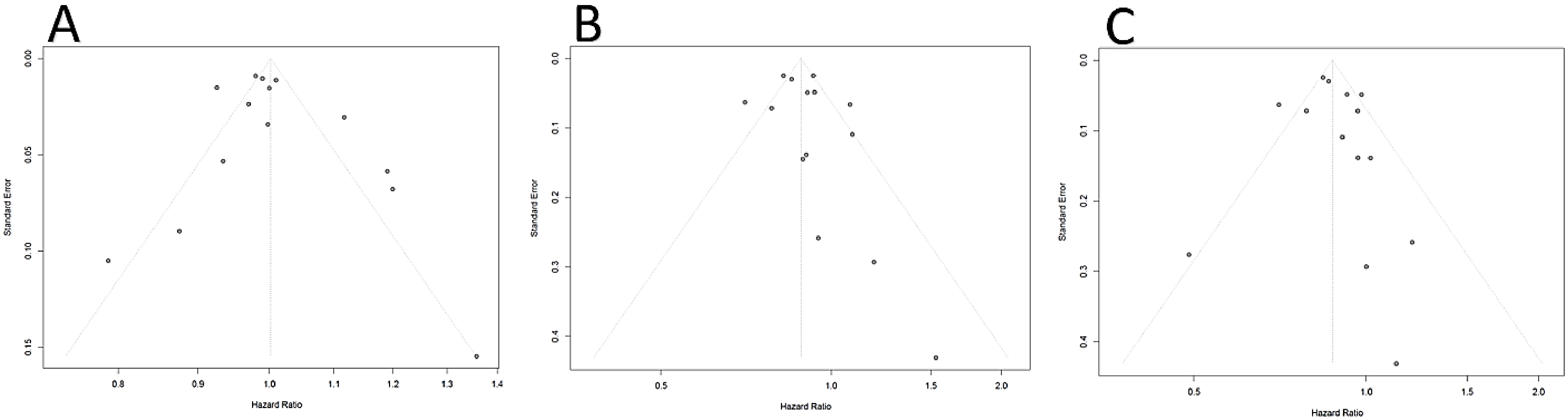

AP animal protein; CHO carbohydrates; PP plant protein

[a] At least 10 studies are required to conduct a funnel plot.

A: Funnel plot showing standard error against the hazard ratio for the substitution of CHO with AP. P-value for Eggers linear regression test: p-value = 0.42

B: Funnel plot showing standard error against the hazard ratio for the substitution of CHO with PP. P-value for Eggers linear regression test: p-value = 0.38

C: Funnel plot showing standard error against the hazard ratio for the substitution of AP with PP. P-value for Eggers linear regression test: p-value = 0.54

Results of Egger's regression test indicated no evidence of publication bias for all-cause mortality for any of the substitutions presented.

Supplemental table 1: Eligibility criteria by the PICOS statement

	**Inclusion criteria**	**Exclusion criteria**
**P** (population)	Adults (aged ≥18 years), generally healthy population: >2/3 of the study population without a particular condition i.e., stable coronary heart disease, chronic kidney disease, diabetes, cancer	Studies involving exclusively infants, children, adolescents, or pregnant women
**I** (intervention/exposure)<br><br>**C** (comparison)	Substitution analyses:<br>Network 1: Overall macronutrient network: FAT, PRO, CHO;<br>Network 2: Fatty acids expanded network: SFA, MUFA, PUFA, TFA, CHO, PRO;<br>Network 3: MUFA-origin network: plant MUFA, animal MUFA, SFA, PUFA, CHO, PRO;<br>Network 4: PUFA-origin network: n-3 PUFA, n-6 PUFA, SFA, MUFA, TFA, CHO, PRO;<br>Network 5: Fat-origin subnetwork: AF, PF, CHO, PRO;<br>Network 6: Protein-origin subnetwork: AP, PP, SFA, MUFA, PUFA, TFA, CHO;<br>Network 7: Carbohydrate-origin subnetwork[a]: high-quality carbohydrates / Polysaccharides, low-quality carbohydrates / Mono-/Disaccharides, SFA, MUFA, PUFA, TFA, PRO;	a. Supplements or only one particular bioactive plant compound<br><br>b. Substitution of food groups or foods<br><br>b. No substitution analysis
**O** (outcome)	- All-cause mortality **(present publication)**<br>- Cardiovascular disease, coronary heart disease, stroke (incidence and/or mortality)<br>- Type 2 Diabetes<br>- Cancer (incidence and/or mortality)<br>- Adiposity (obesity, overweight, changes in body weight/ waist circumference)<br>- Age related outcomes (dementia, cognitive decline, frailty, sarcopenia)<br>- Other outcomes (hypertension, atrial fibrillation, heart failure, chronic kidney disease)	Biomarkers of cardiometabolic risk (e.g., fasting glucose, blood lipids, etc)
**S** (study design)	Prospective observational studies (e.g., prospective observational study, nested case-control study, case-cohort study, follow up of RCTs)	In vitro/animal experiments, cross-sectional and retrospective case-control studies

AF animal fat; animal MUFA monounsaturated fatty acids of animal origin; AP animal protein; CHO carbohydrates; MUFA monounsaturated fatty acids; n-3 PUFA n-3 polyunsaturated fatty acids; n-6 PUFA n-6 polyunsaturated fatty acids; PF plant fat; PICOS Population, Intervention, Comparison, Outcome Study design; plant MUFA monounsaturated fatty acids of plant origin; PP plant protein; PRO protein; PUFA polyunsaturated fatty acids; SFA saturated fatty acids; TFA trans-fatty acids;

[a] originally planned network: glucose, fructose, sucrose, starch, fat, protein; Modified in line with the WHO report (1).

Supplemental table 2: General characteristics of the included prospective observational studies for the outcome all-cause mortality

Author, year	Cohort name, country	Disease status	Follow up (years)	Sex	Mean age (years)	Mean BMI (kg/m²)	Number of participants	Number of cases	Outcome assessment	Exposure assessment	Multiple dietary assessment
Argos, 2013 (2)	HEALS, Bangladesh	General healthy	9	M/W	36.9	19.7	17 244	818	Report of close relatives or neighbors of deceased participants, verbal autopsy questionnaire; verbal autopsies were reviewed by a panel of local expert physicians	Validated (through food diaries) FFQ at baseline	No
Bajracharya, 2023 (3)	EPIC-Heidelberg, Germany	General healthy	22.7	M/W	51.4	25.8	24 106	4 029	Record linkages with registries for vital status	Validated (through 24h recalls) FFQ at baseline	No
Budhathoki, 2019 (4)	JPHC, Japan	General healthy	18	M/W	55.7	23.5	70 696	12 381	Residential registry	Validated (through 14- or 28-day dietary records) FFQ, repeated every 5 years	Yes, but only single FFQ was used

Chen, 2020 (5)	Rotterdam, Netherlands	General healthy	13	M/W	63.7	26.6	7 786	3 589	Clinical follow-up data collection, municipal records, information from medical records at general practitioner's offices, hospitals and nursing homes	Validated (through 24 h food records and 24-h urinary urea excretion samples) FFQ at baseline	No
Das, 2022 (6)	CHAMP, Australia	General healthy, older population	3.7	M	81.1	27.2	794	162	Data from three population databases held by the Centre for Health Record Linkage, all-cause mortality was assessed as a result of cancer and CVD	Validated (through 4-day weighed food record), standardized DHQ, administered by research dietitians at baseline	No
Dehghan, 2017 (7)	PURE, Multiple Countries	General healthy	7.4	M/W	50.3	NR	135 335	5 796	Standardized case-report forms were used to record data on mortality	Validated (through 24h recalls), country-specific (or region-specific in India) FFQ at baseline	No

Dominguez, 2018 (8)	SUN, Spain	General healthy	9.5	M/W	53.9	25.3	18 540	255	Uninterrupted and dynamic follow-up, every six months National Death Index data were checked	Validated (through 4-day records) FFQ at baseline	Yes, every two years, but only baseline FFQ was used for the current analysis
Fontana, 2021 (9)	EPIC-Italy, Italy	General healthy	15.2	M/W	50.7	26.0	45 009	2 449	Municipal registries, date of death was obtained from the official mortality indices	Validated (through 24h recall), center-specific FFQ at baseline	No
Fridén, 2023 (10)	ULSAM, Sweden	General healthy, older population	13.7	M	71.0	NR	1 133	774	Cause of death registry	Validated (through biomarkers) 7-day dietary record at baseline, with the use of a validated (through 7-day weighed food record) pre-coded menu book from the Swedish National Food Agency	No

Guasch-Ferré, 2015 (11)	PREDIMED, Spain	High CVD risk, 50% T2D	6	M/W	67.0	29.9	7 038	414	Yearly questionnaires and examinations for all participants, family physicians, yearly review of medical records, yearly consultation of the National Death Index	Validated (3-day diet records) FFQ, done by face to face interview with a registered dietitian, annually	Yes, annually; updated intakes were used in models
Guasch-Ferré, 2019 (published and unpublished data) (12)	NHS, USA	General healthy	22	W	67.8	26.5	63 412	12 774	Linkage with vital records of states and of the National Death Index, reports from next of kin and postal authorities	Validated (through dietary records) FFQ, at baseline and every 4 years	Yes, every four years
	HPFS, USA			M	67.7	23.9	29 966	7 898			
Hernández-Alonso, 2016 (13)	PREDIMED, Spain	High CVD risk, 50% T2D	4.8	M/W	67.0	30.0	7 216	323	Yearly questionnaires and examinations for all participants, family physicians, yearly review of medical records, yearly consultation of the National Death Index	Validated (3-day diet records) FFQ, done by face to face interview with a registered dietitian, annually	Yes, annually
Ho, 2020 (published and unpublished data) (14)	UKB, United Kingdom	General healthy	13.2	M/W	56.0	25.1	208 294	12 611	Death certificates held within NHS Information Centre and NHS Central Register Scotland	Validated (through 24h hour recall administered by interviewer) Oxford WebQ web based 24h recall	Yes, up to four times

Huang, 2020 (15)	NIH-AARP, USA	General healthy	15.5	M/W	62.0	27.0	416 104	77 614	Linkage with Social Security Administration Death Master File	Validated (by 24h recall), self-administered FFQ at baseline	No
Kelemen, 2005 (16)	IWHS, USA	General healthy	15	W	75.8	25.8	29 017	3 978	Linkage with National Death Index	Validated (through 24h recalls) FFQ at baseline	No
Kwon, 2021 (17)	KoGES, Korea	General healthy	8.15	M/W	53.8	24.0	194 295	3 866	Publicly accessible files in the KoGES linked National Death Index	Validated (through dietary records) FFQ at baseline	No
Laake, 2012 (18)	NCS, Norway	General healthy	25.8	M/W	41.0	24.6	71 464	11 890	Linkage with Statistics Norway using the unique identification number	Validated (through 24h recall) FFQ	Yes, three times, at first screening only 59% of participants received a FFQ
Laguna, 2021 (19)	PREDIMED, Spain	High CVD risk, 50% T2D	6	M/W	67.0	30.0	7 056	409	Yearly questionnaires and examinations for all participants, family physicians, yearly review of medical records, yearly consultation of the National Death Index	Validated (3-day diet records) FFQ, done by face to face interview with a registered dietitian, annually	Yes, annually

Levine, 2014 (20)	NHANES III, USA	General healthy	18	M/W	65.0	27.2	6 381	2 578	Linkage with the National Death Index	24h recall, via automated, microcomputer-based coding system	No
Li, 2022 (21)	NHANES, USA	Prediabetes	7.4	M/W	55.3	29.8	9 793	1 352	Linkage with national death index	≥one 24-h recall conducted in person in the NHANES Mobile Examination Center or via telephone	Mixed (no: NHANES 1999 to 2002; yes: 2003 to 2014)
Mao, 2020 (22)	CHNS, China	General healthy	14	M/W	41.2	22.4	14 305	1 006	Based on report of household members in each survey and denoted in CHNS database	3-day 24h dietary recalls at individual level in combination with weighing inventory at household level	Yes
Merono, 2022 (23)	InCHIANTI, Italy	General healthy, older population	12	M/W	75.0	27.0	1 139	811	Mortality General Registry from the Tuscany Region, as well as death certificates	Validated (through 24h recalls) Italian FFQ, assessed by trained interviewers	Yes (three to four times)
Nagata, 2012 (24)	Takahama, Japan	General healthy	16	M/W	54.1	22.3	28 356	4 616	National Vital Statistics	Validated (3-day dietary records and 24h recalls) FFQ at baseline	No
Ricci, 2018 (25)	NHANES, USA	General healthy	6.1	M/W	46.2	27.4	18 372	1 118	Linkage with national death index	≥one 24-h recall conducted in person in the NHANES Mobile Examination Center or via telephone	Mixed (no: NHANES 1999 to 2002; yes: 2003 to 2014)

Shan, 2020 (26)	NHANES, USA	General healthy	8	M/W	NR	NR	37 233	4 866	Linkage with national death index	≥one 24-h recall conducted in person in the NHANES Mobile Examination Center or via telephone	Mixed (no: NHANES 1999 to 2002; yes: 2003 to 2014)
Song, 2016 (27)	NHS, USA	General healthy	27	W	49.0	25.9	131 342	36 115	Linkage with vital records of states and of the National Death Index, reports from next of kin and postal authorities	Validated (through dietary records) FFQ at baseline and every 4 years	Yes, every four years
	HPFS, USA			M							
Sun, 2021 (28)	WHI, USA	General healthy	18.1	W	62.8	27.7	102 521	25 976	Death certificates, medical records, autopsy reports, or linkage to the National Death Index	Validated (through 24h dietary recalls and a 4-day food record), self-administered FFQ at baseline	No
Virtanen, 2019 (29)	KIHD, Finland	General healthy	22.3	M	53.1	26.9	2 641	1 225	Linkage to national causes of death register, with social security number	Instructed food record of 4 days at baseline	No
Wakai, 2014 (30)	JACC, Japan	General healthy	19.3	M/W	56.1	22.8	58 672	11 656	Population registries from involved municipalities	Validated (through 3-day weighed diet records) FFQ at baseline	No
Wang, 2016 (31)	NHS, USA	General Healthy	27.3	W	46.5	24.2	126 233	33 304	Linkage with vital records of states and of the	Validated (through dietary records)	Yes, every four years
	HPFS,			M	53.2	25.4					

	USA								National Death Index, reports from next of kin and postal authorities	FFQ at baseline and every 4 years	
Wu, 2020 (32)	CHNS, China	General healthy	14	M/W	40.8	22.1	14 305	1 006	Based on report of household members in each survey and denoted in CHNS database	3-day 24h dietary recalls at individual level in combination with weighing inventory at household level	Yes
Zeng, 2022 (33)	NHANES, USA	General healthy	7.1	M/W	46.0	28.7	35 692	3 854	Linkage with national death index	≥one 24-h recall conducted in person in the NHANES Mobile Examination Center or via telephone	Mixed (no: NHANES 1999 to 2002; yes: 2003 to 2014)
Zhao, 2023 (34)	NIH-AARP, USA	General healthy	23.5	M/W	61.2	26.7	371 159	165 698	Linkage with Social Security Administration Death Master File	Validated (through 24h recall), self-administered FFQ at baseline	No
Zhou, 2022 (35)	CHNS, China	General healthy	9	M/W	44.0	22.8	17 310	1 324	Based on report of household members in each survey and denoted in CHNS database	3-day 24h dietary recalls at individual level in combination with weighing inventory at household level	Yes
Zhuang, 2019a (36)	NIH-AARP, USA	General healthy	16	M/W	62.8	26.3	521 120	129 328	Linkage with Social Security Administration Death Master File	Validated (through 24h recall), self-administered FFQ at baseline	No
Zhuang, 2019b (37)	CHNS, China	General healthy	14	M/W	41.3	22.5	14 383	1 011	Based on report of household members in each	3-day 24h dietary recalls at individual level in	Yes

									survey and denoted in CHNS database	combination with weighing inventory at household level	

AF animal fat; animal MUFA monounsaturated fatty acids of animal origin; A-oil animal oil; AP animal protein; BMI body mass index; CHAMP Concord Health Ageing in Men Project; CHNS Chinese Health and Nutrition Survey; CHO carbohydrates; CI confidence interval; d day; EPIC European Prospective Investigation into Cancer and Nutrition; FFQ food frequency questionnaire; g/d grams/day; HEALS Health Effects of Arsenic Longitudinal Study; HPFS Health Professionals Follow-Up Study; HR hazard ratio; HRT hormone replacement therapy; InCHIANTI Invecchiare in Chianti aging in the Chianti area; IWHS Iowa Women's Health Study; JACC Japan Collaborative Cohort; JPHC Japan Public Health Center–based Prospective Cohort; kcal kilocalories; KIHD Kuopio Ischaemic Heart Disease Risk Factor Study; KoGES Korean Genome and Epidemiology Study; M men; MMSE Mini-Mental State Examination; MUFA monounsaturated fatty acids; n-3 PUFA n-3 polyunsaturated fatty acids; n-6 PUFA n-6 polyunsaturated fatty acids; NCS Norwegian Counties Study; NHANES National Health and Nutrition Examination Survey; NHANES III National Health and Nutrition Examination Survey III; NHS Nurses Health Study; NIH-AARP National Institutes of Health - American Association of Retired Persons Diet and Health Study; NR not reported; PHFO partially hydrogenated fish oil; PHVO partially hydrogenated vegetable oil; plant MUFA monounsaturated fatty acids of plant origin; P-oil oil from plant origin; PP plant protein; PREDIMED PREvención con DIeta MEDiterránea; PRO protein; PUFA polyunsaturated fatty acids; PURE Prospective Urban Rural Epidemiology; rTFA ruminant trans-fatty acids; SD standard deviation; SFA saturated fatty acids; SUN Seguimiento Universidad de Navarra; T2D type 2 diabetes; TFA trans-fatty acids; UKB United Kingdom Biobank; ULSAM Uppsala Longitudinal Study of Adult Men; W women; WHI Women's Health Initiative; WHR waist-to-hip ratio; WHS The Women's Health Study;

Supplemental table 3: (Macro)nutrient replacement including unit, effect estimates with 95% CI (as reported by authors), and adjustment factors of the included studies for the outcome all-cause mortality

Author, year	Cohort, country	↓ Nutrient[a]	↑ Replacement[a]	Unit	Hazard ratio (95% CI)	Adjustment factors[b]
Argos, 2013 (2)	HEALS, Bangladesh	CHO	PRO	10.2%	1.00 (reference)	Age, sex, energy, BMI, smoking, formal education, years of education and cohort, height, water arsenic concentration
				12.1%	0.96 (0.79, 1.17)	
				26.0%	1.09 (0.88, 1.34)	
		FAT	PRO	10.2%	1.00 (reference)	
				12.1%	0.96 (0.79, 1.16)	
				26.0%	1.07 (0.85, 1.35)	
		CHO	FAT	4.8%	1.00 (reference)	
				8.6%	1.11 (0.92, 1.35)	
				23.4%	1.22 (0.98, 1.51)	
Bajracharya, 2023 (3)	EPIC-Heidelberg, Germany	AP	PP	3%	0.97 (0.90, 1.06)	Age, sex, energy, BMI, smoking, alcohol, fiber intake
		AP	SFA	3%	0.91 (0.86, 0.96)	
		AP	MUFA	3%	0.89 (0.82, 0.97)	
		AP	PUFA	3%	1.01 (0.94, 1.08)	
		AP	Mono-/Disaccharides	3%	0.93 (0.90, 0.97)	
		AP	Other CHO	3%	0.94 (0.91, 0.98)	
		PP	SFA	3%	0.93 (0.85, 1.01)	
		PP	MUFA	3%	0.91 (0.82, 1.02)	
		PP	PUFA	3%	1.03 (0.93, 1.14)	
		PP	Mono-/Disaccharides	3%	0.95 (0.88, 1.03)	
		PP	Other CHO	3%	0.96 (0.88, 1.05)	
		SFA	MUFA	3%	0.98 (0.88, 1.09)	
		SFA	PUFA	3%	1.10 (1.04, 1.17)	
		SFA	Mono-/Disaccharides	3%	1.02 (0.97, 1.07)	
		SFA	Other CHO	3%	1.03 (0.98, 1.09)	
		MUFA	PUFA	3%	1.12 (1.01, 1.26)	

		MUFA	Mono-/Disaccharides	3%	1.04 (0.97, 1.11)	
		MUFA	Other CHO	3%	1.05 (0.98, 1.13)	
		PUFA	Mono-/Disaccharides	3%	0.92 (0.87, 0.98)	
		PUFA	Other CHO	3%	0.93 (0.88, 0.99)	
		Mono-/Disaccharides	Other CHO	3%	1.01 (0.99, 1.03)	
Budhathoki, 2019 (4)	JPHC, Japan	CHO	PRO	11.3%	1.00 (reference)	Age, sex, energy, BMI, smoking, alcohol, occupation status, intake of green tea, coffee, physical activity
				13.0%	0.95 (0.89, 1.01)	
				14.3%	0.93 (0.86, 1.00)	
				15.6%	0.92 (0.84, 0.99)	
				17.6%	0.99 (0.90, 1.09)	
		CHO	AP	4.3%	1.00 (reference)	
				6.1%	0.91 (0.85, 0.97)	
				7.5%	0.95 (0.88, 1.02)	
				8.9%	0.97 (0.89, 1.05)	
				11.2%	0.98 (0.88, 1.08)	
		CHO	PP	5.0%	1.00 (reference)	
				6.0%	0.89 (0.83, 0.95)	
				6.6%	0.88 (0.82, 0.95)	
				7.3%	0.84 (0.77, 0.92)	
				8.4%	0.87 (0.78, 0.96)	
Chen, 2020 (5)	Rotterdam, Netherlands	CHO	PRO	5%	1.09 (1.02, 1.17)	Age, sex, energy, BMI, smoking, alcohol, education, fiber, overall diet quality score, physical activity, cohort (RS-I, -II, and -III)
		CHO	AP	5%	1.20 (1.05, 1.37)	
		CHO	PP	5%	1.09 (0.88, 1.35)	
		FAT	PRO	5%	1.08 (1.01, 1.15)	
		FAT	AP	5%	1.16 (1.02, 1.33)	
		FAT	PP	5%	1.04 (0.85, 1.27)	
Das, 2022 (6)	CHAMP, Australia	CHO	PRO	72 g/d	1.00 (reference)	Age, energy, BMI, smoking, alcohol, income, marital status, living arrangement, physical activity, number of comorbidities, self-rated health, MMSE, polypharmacy
				86 g/d	0.41 (0.19, 0.87)	
				100 g/d	0.41 (0.19, 0.90)	
				116 g/d	0.75 (0.37, 1.51)	
				133 g/d	0.69 (0.34, 1.39)	
		FAT	PRO	72 g/d	1.00 (reference)	Age, energy, BMI, smoking, alcohol, income, marital status, living arrangement, fiber, vitamin A, C, E, folate, sodium, potassium, calcium,
				86 g/d	0.42 (0.20, 0.88)	

				100 g/d	0.40 (0.18, 0.90)	magnesium, iron, zinc, MMSE, polypharmacy, self-rated health, number of comorbidities
				116 g/d	0.75 (0.36, 1.55)	
				133 g/d	0.70 (0.34, 1.45)	
Dehghan, 2017 (7)	PURE, Multiple Countries	CHO	SFA	5%	0.97 (0.90, 1.04)	Age, sex, energy, waist-to-hip ratio, smoking, education, physical activity, diabetes, urban or rural location, center was random effect and frailty models
		CHO	MUFA	5%	0.97 (0.88, 1.08)	
		CHO	PUFA	5%	0.89 (0.82, 0.97)	
		CHO	PRO	5%	0.96 (0.90, 1.02)	
Dominguez, 2018 (8)	SUN, Spain	MUFA	SFA	5%	1.41 (1.03, 1.93)	Age, sex, energy, BMI, smoking, university education, prescription of special diets at baseline, snacking between meals, physical activity, baseline hypercholesterolemia, baseline hypertension, history of depression, history of cardiovascular disease, history of cancer, history of diabetes, year of entering the cohort, hours per day spent watching television
		PUFA	SFA	5%	1.17 (0.87, 1.58)	
		CHO	SFA	5%	1.34 (1.02, 1.75)	
Fontana, 2021 (9)	EPIC-Italy, Italy	CHO	PRO	3%	0.98 (0.93, 1.04)	Age (stratified), sex (stratified), energy, BMI, WHR, smoking, alcohol, education, fiber, physical activity, center (stratified)
		CHO	AP	3%	0.96 (0.90, 1.02)	
		CHO	PP	3%	0.94 (0.79, 1.21)	
		AP	PP	3%	0.98 (0.83, 1.15)	
Fridén, 2023 (10)	ULSAM, Sweden	SFA	PUFA	100 kcal	1.27 (0.86, 1.88)	Age, energy, smoking, education, physical activity, family history of CVD, family history of type-2 diabetes, stress, sleep
		SFA	CHO	100 kcal	0.79 (0.65, 0.97)	
Guasch-Ferré, 2015 (11)	PREDIMED, Spain	CHO	TFA	0.05%	1.00 (reference)	Age, sex, energy, BMI, smoking, alcohol, education, fiber, dietary cholesterol, physical activity, baseline diabetes, hypertension, hypercholesterolemia, family history of coronary heart disease, antihypertensive medication, oral antidiabetic agents, lipid-lowering drugs, intervention group
				0.10%	1.11 (0.80, 1.54)	
				0.16%	0.86 (0.59, 1.24)	
				0.23%	1.13 (0.78, 1.64)	
				0.36%	1.29 (0.87, 1.90)	
		CHO	MUFA	5%	0.86 (0.76, 0.98)	
		CHO	PUFA	5%	0.56 (0.40, 0.79)	
		CHO	FAT	5%	0.87 (0.80, 0.95)	
		SFA	CHO	5%	1.04 (0.81, 1.33)	
		SFA	MUFA	5%	0.91 (0.65, 1.26)	
		SFA	PUFA	5%	0.61 (0.39, 0.97)	
		TFA	MUFA	1%	0.99 (0.92, 1.06)	
		TFA	PUFA	1%	0.92 (0.83, 1.00)	
Guasch-Ferré, 2019 (published and	NHS, USA	SFA	Plant MUFA	5%	0.86 (0.77, 0.95)	Age, energy, BMI, smoking, alcohol, fruits and vegetables, coffee intake, physical activity, baseline hypertension, baseline hypercholesterolemia, family history of myocardial infarction, family history of diabetes mellitus,
		Refined CHO	Plant MUFA	5%	0.86 (0.80, 0.92)	
		TFA	Plant MUFA	2%	0.89 (0.83, 0.97)	
		Animal MUFA	Plant MUFA	5%	0.77 (0.70, 0.84)	

unpublished data) (12)						family history of cancer, menopausal status, postmenopausal hormone use, current aspirin use, multivitamin use, ethnicity
	HPFS, USA	SFA	Plant MUFA	5%	0.81 (0.70, 0.95)	Age, energy, BMI, smoking, alcohol, fruits and vegetables, coffee intake, physical activity, baseline hypertension, baseline hypercholesterolemia, family history of myocardial infarction, family history of diabetes mellitus, family history of cancer, current aspirin use, multivitamin use, ethnicity
		Refined CHO	Plant MUFA	5%	0.88 (0.80, 0.96)	
		TFA	Plant MUFA	2%	0.93 (0.84, 1.04)	
		Animal MUFA	Plant MUFA	5%	0.77 (0.69, 0.86)	
Hernández-Alonso, 2016 (13)	PREDIMED, Spain	CHO	PRO	13.9%	1.22 (0.84, 1.77)	Age, sex, energy, BMI, smoking, alcohol, fiber, GI, physical activity, prevalence of diabetes, hypertension, hypercholesterolemia, family history of coronary heart disease, use of aspirin, antihypertensive medication, oral antidiabetic medication, insulin medication and hypocholesterolemic medication, intervention group, node
				15.4%	0.88 (0.60, 1.28)	
				16.5%	1.00 (reference)	
				17.6%	0.93 (0.63, 1.39)	
				19.5%	1.59 (1.08, 2.35)	
		FAT	PRO	13.9%	1.17 (0.80, 1.70)	
				15.4%	0.86 (0.59, 1.25)	
				16.5%	1.00 (reference)	
				17.6%	0.95 (0.64, 1.42)	
				19.5%	1.66 (1.13, 2.43)	
		CHO	AP	8.3%	1.27 (0.87, 1.84)	
				9.8%	0.88 (0.60, 1.29)	
				11.0%	1.00 (reference)	
				12.1%	1.10 (0.74, 1.63)	
				13.9%	1.86 (1.27, 2.73)	
		FAT	AP	8.3%	1.24 (0.86, 1.81)	
				9.8%	0.88 (0.60, 1.29)	
				11.0%	1.00 (reference)	
				12.1%	1.12 (0.76, 1.65)	
				13.9%	1.92 (1.31, 2.82)	
		CHO	PP	4.5%	1.04 (0.72, 1.52)	
				5.1%	0.86 (0.59, 1.25)	
				5.5%	1.00 (reference)	
				5.9%	1.01 (0.70, 1.48)	
				6.6%	1.28 (0.84, 1.94)	
		FAT	PP	4.5%	1.02 (0.70, 1.49)	
				5.1%	0.85 (0.58, 1.24)	
				5.5%	1.00 (reference)	
				5.9%	1.01 (0.70, 1.48)	
				6.6%	1.32 (0.88, 2.00)	

Ho, 2020 (published and unpublished data) (14)	UKB, United Kingdom	CHO	FAT	5%	0.95 (0.94, 0.97)	Age, sex, energy, BMI, smoking, alcohol, fiber, physical activity, systolic blood pressure, baseline diabetes, mental health disorders, deprivation index, ethnicity, height
		CHO	PRO	5%	0.92 (0.90, 0.95)	
		FAT	PRO	5%	0.97 (0.94, 1.00)	
		CHO	SFA	5%	0.97 (0.92, 1.02)	
		CHO	MUFA	5%	0.96 (0.91, 1.02)	
		CHO	PUFA	5%	0.90 (0.83, 0.97)	
		CHO	TFA	5%	0.97 (0.56, 1.65)	
		SFA	MUFA	5%	0.99 (0.90, 1.08)	
		SFA	PUFA	5%	0.93 (0.86, 1.00)	
		SFA	TFA	5%	0.99 (0.56, 1.77)	
		SFA	PRO	5%	0.95 (0.90, 1.00)	
		MUFA	PUFA	5%	0.94 (0.83, 1.06)	
		MUFA	TFA	5%	1.01 (0.59, 1.71)	
		MUFA	PRO	5%	0.96 (0.90, 1.03)	
		PUFA	TFA	5%	1.07 (0.62, 1.86)	
		PUFA	PRO	5%	1.03 (0.95, 1.11)	
		TFA	PRO	5%	0.96 (0.55, 1.66)	
		CHO	AP	5%	0.93 (0.90, 0.95)	
		CHO	PP	5%	0.91 (0.82, 1.01)	
		SFA	AP	5%	0.95 (0.91, 1.01)	
		SFA	PP	5%	0.94 (0.84, 1.04)	
		MUFA	AP	5%	0.96 (0.90, 1.03)	
		MUFA	PP	5%	0.95 (0.84, 1.06)	
		PUFA	AP	5%	1.02 (0.94, 1.11)	
		PUFA	PP	5%	1.00 (0.87, 1.15)	
		TFA	AP	5%	0.95 (0.55, 1.65)	
		TFA	PP	5%	0.93 (0.53, 1.64)	
		AP	PP	5%	0.98 (0.89, 1.08)	
		CHO	AF	5%	0.96 (0.94, 0.98)	
		CHO	PF	5%	0.95 (0.93, 0.97)	
		AF	PF	5%	0.99 (0.97, 1.01)	
		AF	PRO	5%	0.96 (0.92, 0.99)	
		PF	PRO	5%	0.97 (0.94, 1.00)	
		CHO	n-3 PUFA	5%	0.77 (0.58, 1.02)	
		CHO	n-6 PUFA	5%	0.92 (0.85, 1.01)	
		SFA	n-3 PUFA	5%	0.79 (0.60, 1.06)	

		SFA	n-6 PUFA	5%	0.95 (0.87, 1.03)	
		MUFA	n-3 PUFA	5%	0.81 (0.60, 1.08)	
		MUFA	n-6 PUFA	5%	0.96 (0.84, 1.10)	
		n-3 PUFA	n-6 PUFA	5%	1.19 (0.87, 1.63)	
		n-3 PUFA	TFA	5%	1.21 (0.67, 2.20)	
		n-3 PUFA	PRO	5%	1.20 (0.90, 1.60)	
		n-6 PUFA	TFA	5%	1.02 (0.58, 1.78)	
		n-6 PUFA	PRO	5%	1.01 (0.92, 1.10)	
		Sugar	Starch	5%	0.97 (0.95, 0.98)	
		Sugar	SFA	5%	0.95 (0.90, 1.00)	
		Sugar	MUFA	5%	0.95 (0.90, 1.01)	
		Sugar	PUFA	5%	0.89 (0.83, 0.96)	
		Sugar	TFA	5%	1.02 (0.59, 1.74)	
		Sugar	PRO	5%	0.91 (0.89, 0.94)	
		Starch	SFA	5%	0.98 (0.93, 1.03)	
		Starch	MUFA	5%	0.99 (0.93, 1.05)	
		Starch	PUFA	5%	0.92 (0.86, 1.00)	
		Starch	TFA	5%	1.05 (0.61, 1.81)	
		Starch	PRO	5%	0.95 (0.92, 0.98)	
Huang, 2020 (15) (men)[c]	NIH-AARP, USA	AP	PP	3%	0.90 (0.88, 0.93)	Age, energy, BMI, smoking, alcohol, education, fiber, vegetables, fruits, physical activity, diabetes, race or ethnic group, marital status, health status, vitamin supplement use
		CHO	PP	1-SD	0.95 (0.94, 0.97)	Additionally adjusted for median household income
		CHO	AP	1-SD	0.99 (0.98, 1.00)	Additionally adjusted for median household income
Huang, 2020 (15) (women)[c]		AP	PP	3%	0.90 (0.87, 0.93)	Age, energy, BMI, smoking, alcohol, education, fiber, vegetables, fruits, physical activity, diabetes, race or ethnic group, marital status, health status, vitamin supplement use, HRT
		CHO	PP	1-SD	0.95 (0.93, 0.97)	Additionally adjusted for median household income
		CHO	AP	1-SD	0.98 (0.97, 1.00)	Additionally adjusted for median household income
Kelemen, 2005 (16)[d]	IWHS, USA	CHO	PRO	14.1%	1.00 (reference)	Age, energy, BMI, smoking, alcohol, education, fiber, dietary cholesterol, dietary methionine, physical activity, history of hypertension, postmenopausal hormone use, multivitamin use, vitamin E supplement use, family history of cancer
				16.3%	0.95 (0.68, 1.32)	
				17.8%	0.81 (0.58, 1.13)	
				19.4%	0.84 (0.60, 1.17)	
				22.0%	0.99 (0.71, 1.38)	
		CHO	AP	8.9%	1.00 (reference)	
				11.3%	0.93 (0.67, 1.28)	

				12.9%	0.83 (0.60, 1.14)	
				14.7%	0.79 (0.57, 1.09)	
				17.5%	0.82 (0.59, 1.13)	
		CHO	PP	3.7%	1.00 (reference)	
				4.3%	0.90 (0.78, 1.04)	
				4.8%	0.95 (0.82, 1.10)	
				5.3%	0.93 (0.80, 1.08)	
				6.1%	0.95 (0.82, 1.10)	
		AP	PP	3.7%	1.00 (reference)	
				4.3%	0.93 (0.81, 1.07)	
				4.8%	0.98 (0.85, 1.13)	
				5.3%	0.98 (0.85, 1.13)	
				6.1%	0.99 (0.86, 1.14)	
Kwon, 2021 (17)	KoGES, Korea	CHO	FAT	7.4%	1.00 (reference)	Age, sex, energy, BMI, smoking, alcohol, fiber, physical activity, hypertension, diabetes, dyslipidemia
				10.2%	0.91 (0.81, 0.99)	
				12.9%	0.84 (0.75, 0.94)	
				16.0%	0.85 (0.74, 0.97)	
				19.5%	0.89 (0.74, 1.05)	
Laake, 2012 (18) (men and women)[e]	NCS, Norway	CHO	TFA from PHVO	0.08%	1.00 (reference)	Age, sex, energy, BMI, smoking, education, cholesterol, systolic blood pressure
				0.40%	1.01 (0.96, 1.07)	
				0.90%	1.04 (0.98, 1.11)	
				1.40%	0.95 (0.89, 1.02)	
				1.90%	0.96 (0.88, 1.05)	
		CHO	TFA from PHFO	0.60%	1.00 (reference)	
				1.10%	1.01 (0.95, 1.06)	
				1.60%	0.99 (0.93, 1.06)	
				2.10%	0.94 (0.87, 1.02)	
				2.60%	1.03 (0.94, 1.14)	
Laake, 2012 (18) (men)[e]		CHO	rTFA	0.32%	1.00 (reference)	Age, energy, BMI, smoking, education, cholesterol, systolic blood pressure
				0.47%	1.00 (0.93, 1.07)	
				0.62%	1.03 (0.94, 1.13)	
				0.77%	1.03 (0.91, 1.16)	
				0.92%	0.98 (0.83, 1.16)	
Laake, 2012 (18) (women)[e]		CHO	rTFA	0.32%	1.00 (reference)	Age, energy, BMI, smoking, education, cholesterol, systolic blood pressure
				0.47%	0.95 (0.87, 1.03)	
				0.62%	1.02 (0.92, 1.13)	

				0.77%	0.99 (0.86, 1.13)	
				0.92%	1.10 (0.93, 1.31)	
Laguna, 2021 (19)	PREDIMED, Spain	Liquid sugars	SFA	5%	1.00 (0.67, 1.49)	Age, sex, energy, BMI, smoking, alcohol, salt intake, red meat, processed meat, adherence to Mediterranean diet score, physical activity, history of diabetes, history of hypertension, history of dyslipidemia, recruitment center (stratified), aspirin intake, vitamin supplementation, family history of cancer, intervention group, and additionally adjusted for robust variance estimators to account for small deviations from individual randomization.
		Liquid sugars	TFA	1%	1.23 (0.54, 2.79)	
		Liquid sugars	MUFA	5%	0.73 (0.54, 1.00)	
		Liquid sugars	PUFA	5%	0.66 (0.46, 0.94)	
		Liquid sugars	Total solid sugars	5%	0.77 (0.57, 1.04)	
		Liquid sugars	Complex CHO	5%	0.83 (0.63, 1.10)	
Levine, 2014 (20) (aged: 50-65y)[f]	NHANES III, USA	CHO	PRO	1%	0.99 (0.98, 1.01)	Age, sex, energy, waist circumference, smoking, education, chronic conditions (diabetes, cancer, myocardial infarction), trying to lose weight in the last year, diet changed in the last year, reported intake representative of typical diet, race/ethnicity
		FAT	PRO	1%	1.00 (0.99, 1.01)	
Levine, 2014 (20) (aged: >66y)[f]		CHO	PRO	1%	1.00 (0.99, 1.01)	Age, sex, energy, waist circumference, smoking, education, chronic conditions (diabetes, cancer, myocardial infarction), trying to lose weight in the last year, diet changed in the last year, reported intake representative of typical diet, race/ethnicity
		FAT	PRO	1%	1.00 (0.99, 1.00)	
Li, 2022 (21)	NHANES, USA	Low-quality CHO	High-quality CHO	5%	0.85 (0.73, 0.99)	Age, sex, energy, BMI, smoking, alcohol, education, physical activity, cholesterol intake, self-reported hypertension, hypercholesterolemia, cardiovascular disease, cancer, race/ethnicity, marital status, family income : poverty ratio
		Low-quality CHO	PP	3%	0.70 (0.46, 1.07)	
		Low-quality CHO	AP	3%	0.81 (0.61, 1.07)	
		Low-quality CHO	UFA	3%	0.86 (0.77, 0.97)	
		Low-quality CHO	SFA	3%	1.12 (0.92, 1.36)	
		SFA	High-quality CHO	5%	0.71 (0.51, 0.99)	
		SFA	Low-quality CHO	5%	0.83 (0.60, 1.15)	
		SFA	PP	3%	0.63 (0.40, 0.98)	
		SFA	AP	3%	0.72 (0.50, 1.04)	
Mao, 2020 (22)	CHNS, China	SFA	MUFA	5%	0.95 (0.84, 1.07)	Age, sex, energy, BMI, smoking, alcohol, education, physical activity, history of hypertension, history of diabetes, marital status, residence
		SFA	Plant MUFA	5%	0.85 (0.75, 0.95)	
		SFA	Animal MUFA	5%	1.11 (0.99, 1.26)	
		CHO	MUFA	7.8%	1.00 (reference)	
				11.1%	0.89 (0.71, 1.13)	
				14.6%	0.94 (0.70, 1.24)	

				18.4%	0.79 (0.57, 1.09)	
		CHO	Plant MUFA	3.4%	1.00 (reference)	
				5.1%	0.67 (0.55, 0.81)	
				7.1%	0.71 (0.58, 0.86)	
				9.4%	0.72 (0.58, 0.89)	
		CHO	Animal MUFA	2.1%	1.00 (reference)	
				4.8%	0.96 (0.77, 1.21)	
				7.5%	1.12 (0.84, 1.49)	
				10.3%	1.38 (0.97, 1.95)	
Merono, 2022 (23)	InCHIANTI, Italy	CHO	PRO	13.3%	1.00 (reference)	Age, sex, energy, BMI, smoking, alcohol, education, economic situation, Mediterranean diet adherence score, physical activity, impaired renal function, diabetes, ischemic heart disease, cerebrovascular disease, peripheral artery disease, congestive heart failure, chronic obstructive pulmonary disease, cancer, dementia, cognitive impairment, Parkinson's disease, ADL disability
				14.7%	0.92 (0.74, 1.14)	
				15.7%	0.95 (0.77, 1.18)	
				16.9%	0.88 (0.70, 1.10)	
				18.7%	0.79 (0.62, 1.00)	
		CHO	AP	7.2%	1.00 (reference)	
				8.8%	0.91 (0.74, 1.13)	
				9.9%	0.86 (0.67, 1.09)	
				11.2%	0.76 (0.59, 0.97)	
				13.3%	0.77 (0.59, 1.01)	
		CHO	PP	4.4%	1.00 (reference)	
				5.3%	1.05 (0.83, 1.33)	
				5.7%	1.04 (0.82, 1.32)	
				6.3%	0.98 (0.76, 1.27)	
				7.1%	0.99 (0.74, 1.34)	
Nagata, 2012 (24) (men)[c]	Takahama, Japan	CHO	FAT	16.3%	1.00 (reference)	Age, energy, BMI, smoking, alcohol, education, fruit, vegetables, dietary fiber, physical activity, histories of diabetes and hypertension, marital status, height
				20.1%	0.88 (0.77, 1.00)	
				22.7%	0.92 (0.80, 1.05)	
				25.5%	0.85 (0.73, 0.99)	
				29.6%	0.83 (0.70, 0.99)	
		CHO	SFA	4.2%	1.00 (reference)	
				5.4%	0.95 (0.83, 1.08)	
				6.2%	0.93 (0.80, 1.07)	
				7.1%	0.99 (0.85, 1.16)	
				8.7%	0.89 (0.74, 1.08)	
		CHO	MUFA	5.3%	1.00 (reference)	

				6.7%	0.97 (0.85, 1.11)	
				7.7%	0.94 (0.80, 1.09)	
				8.8%	0.87 (0.72, 1.05)	
				10.4%	1.02 (0.81, 1.28)	
		CHO	PUFA	4.2%	1.00 (reference)	
				5.1%	0.84 (0.73, 0.97)	
				5.8%	0.82 (0.71, 0.96)	
				6.6%	0.85 (0.72, 1.01)	
				7.7%	0.77 (0.62, 0.95)	
		CHO	Long-chain n-3 PUFA	0.15%	1.00 (reference)	
				0.22%	1.00 (0.88, 1.14)	
				0.28%	0.97 (0.84, 1.11)	
				0.37%	0.95 (0.82, 1.11)	
				0.56%	1.02 (0.85, 1.22)	
Nagata, 2012 (24) (women)[c]		CHO	FAT	16.9%	1.00 (reference)	Age, energy, BMI, smoking, alcohol, education, fruit, vegetables, dietary fiber, physical activity, histories of diabetes and hypertension, marital status, height
				21.0%	1.02 (0.89, 1.16)	
				23.7%	1.05 (0.91, 1.21)	
				26.2%	1.10 (0.94, 1.30)	
				29.6%	1.10 (0.91, 1.34)	
		CHO	SFA	4.4%	1.00 (reference)	
				5.7%	0.95 (0.83, 1.10)	
				6.6%	1.04 (0.89, 1.21)	
				7.4%	1.09 (0.93, 1.29)	
				8.8%	1.22 (0.99, 1.49)	
		CHO	MUFA	5.4%	1.00 (reference)	
				6.9%	0.92 (0.80, 1.06)	
				7.9%	1.02 (0.87, 1.21)	
				8.9%	0.95 (0.77, 1.16)	
				10.4%	1.00 (0.78, 1.29)	
		CHO	PUFA	4.4%	1.00 (reference)	
				5.4%	0.92 (0.80, 1.06)	
				6.0%	0.99 (0.84, 1.17)	
				6.7%	0.98 (0.81, 1.18)	
				7.8%	0.96 (0.76, 1.20)	
		CHO	Long-chain n-3 PUFA	0.13%	1.00 (reference)	
				0.20%	1.02 (0.89, 1.17)	

				0.25%	1.02 (0.88, 1.17)	
				0.32%	0.91 (0.78, 1.07)	
				0.47%	0.98 (0.82, 1.18)	
Ricci, 2018 (25)	NHANES, USA	SFA	MUFA	10%	0.97 (0.93, 1.00)	Age, sex, energy, BMI, smoking, alcohol, education, fiber, sedentariness (subjects who declared having more than 8 h/d of sedentary activity or who do not declared moderate or vigorous physical activity), blood pressure (systolic and diastolic blood pressure as continuous data), ethnicity
		SFA	PUFA	10%	0.92 (0.90, 0.95)	
		MUFA	PUFA	10%	0.96 (0.92, 1.00)	
Shan, 2020 (26)	NHANES, USA	AP	PP	5%	0.49 (0.32, 0.74)	NR
Song, 2016 (27)	NHS, USA	CHO	AP	10%	1.02 (0.98, 1.07)	Age, sex, energy, BMI, smoking, alcohol, glycemic index, whole grains, fiber, fruits, and vegetables, physical activity, history of hypertension diagnosis, multivitamin use, calendar time
		CHO	PP	3%	0.96 (0.91, 1.02)	
	HPFS, USA	CHO	AP	10%	1.00 (0.94, 1.06)	
		CHO	PP	3%	0.81 (0.75, 0.87)	
Sun, 2021 (28)	WHI, USA	CHO	AP	5%	0.99 (0.97, 1.01)	Age, energy, BMI, smoking, alcohol, education, income, fiber, glycemic load, physical activity, baseline diabetes mellitus status, baseline high blood cholesterol status, family history of heart attack/stroke, race/ethnicity, Observational Study/Clinical Trials, unopposed estrogen use, estrogen + progesterone use
		CHO	PP	5%	0.86 (0.80, 0.93)	
		AP	PP	5%	0.86 (0.81, 0.91)	
Virtanen, 2019 (29)	KIHD, Finland	CHO	PRO	5 g/d	1.03 (1.01, 1.05)	Age, energy, BMI, smoking, alcohol, education, income, fiber, physical activity, diagnosis of type 2 diabetes, cardiovascular disease, cancer, hypertension or use of cardiac, hypercholesterolemia, hypertension, or diabetes medications, examination year, marital status
		CHO	AP	5 g/d	1.03 (1.01, 1.05)	
		CHO	PP	5 g/d	1.03 (0.93, 1.13)	
Wakai, 2014 (30) (men)[c]	JACC, Japan	CHO	FAT	10.8%	1.00 (reference)	Age, energy, BMI, smoking, alcohol, education, vegetable intake, fruit intake, daily walking habits, geographic area, sleep duration
				13.9%	1.01 (0.92, 1.11)	
				16.3%	0.99 (0.89, 1.09)	
				18.8%	0.94 (0.84, 1.06)	
				23.3%	1.02 (0.89, 1.17)	
		PRO	FAT	10.8%	1.00 (reference)	
				13.9%	1.02 (0.93, 1.11)	
				16.3%	0.99 (0.91, 1.09)	
				18.8%	0.95 (0.86, 1.05)	
				23.3%	1.02 (0.90, 1.16)	
Wakai, 2014 (30) (women)[c]		CHO	FAT	13.7%	1.00 (reference)	Age, energy, BMI, smoking, alcohol, education, vegetable intake, fruit intake, daily walking habits, geographic area, sleep duration
				17.6%	1.04 (0.95, 1.14)	
				20.1%	1.03 (0.92, 1.14)	
				22.6%	0.91 (0.81, 1.02)	

				26.8%	0.98 (0.86, 1.12)	
		PRO	FAT	13.7%	1.00 (reference)	
				17.6%	1.02 (0.93, 1.13)	
				20.1%	1.00 (0.89, 1.13)	
				22.6%	0.88 (0.76, 1.02)	
				26.8%	0.94 (0.78, 1.14)	
Wang, 2016 (31)	NHS/HPFS USA	SFA	MUFA	5%	0.87 (0.82, 0.93)	Age, energy, BMI, smoking, alcohol, dietary cholesterol, physical activity, history of hypertension, history of hypercholesterolemia, white race, marital status, multivitamin use (yes vs no), vitamin E supplement use, current aspirin use, family history of myocardial infarction, family history of diabetes, family history of cancer, menopausal status and hormone use in women
		SFA	PUFA	5%	0.73 (0.70, 0.77)	
		SFA	TFA	2%	1.16 (1.09, 1.24)	
		SFA	n-6 PUFA	2%	0.93 (0.91, 0.96)	
		SFA	n-3 PUFA	0.3%	0.95 (0.93, 0.96)	
	NHS, USA	CHO	FAT	5%	0.94 (0.93, 0.96)	
		CHO	SFA	5%	1.08 (1.04, 1.12)	
		CHO	PUFA	5%	0.74 (0.69, 0.79)	
		CHO	MUFA	5%	0.88 (0.84, 0.92)	
		CHO	TFA	2%	1.08 (1.00, 1.17)	
		CHO	n-6 PUFA	2%	0.92 (0.89, 0.95)	
		CHO	n-3 PUFA	0.3%	0.96 (0.93, 1.00)	
	HPFS, USA	CHO	FAT	5%	0.97 (0.95, 0.99)	
		CHO	SFA	5%	1.07 (1.01, 1.14)	
		CHO	PUFA	5%	0.71 (0.65, 0.79)	
		CHO	MUFA	5%	0.95 (0.89, 1.02)	
		CHO	TFA	2%	1.34 (1.20, 1.50)	
		CHO	n-6 PUFA	2%	0.88 (0.84, 0.92)	
		CHO	n-3 PUFA	0.3%	0.97 (0.94, 1.01)	
Wu, 2020 (32)	CHNS, China	A-oil	P-oil	8 g/2000 kcal	0.96 (0.92, 1.00)	Age, sex, energy, BMI, smoking, alcohol, education, income, red meat, white meat, vegetables, and fruit, physical activity, history of hypertension, history of diabetes, marital status, geographical location, geographical site
Zeng, 2022 (33)	NHANES, USA	CHO	FAT	5%	1.00 (0.97, 1.02)	Age, sex, energy, BMI, smoking, alcohol, education, family income to poverty ratio, physical activity, diabetes, race/ethnicity, marital status
		CHO	PF	5%	0.97 (0.93, 1.00)	
		CHO	AF	5%	0.99 (0.96, 1.03)	
		CHO	SFA	5%	1.04 (0.98, 1.11)	
		CHO	MUFA	5%	0.99 (0.92, 1.06)	
		CHO	PUFA	5%	0.92 (0.87, 0.96)	
		CHO	PRO	5%	0.97 (0.94, 1.00)	
		CHO	PP	5%	0.93 (0.90, 0.97)	

		CHO	AP	5%	0.97 (0.94, 1.01)	
Zhao, 2023 (34)	NIH-AARP, USA	Low-quality CHO	High-quality CHO	3%	0.992 (0.989, 0.994)	Age, sex, energy, BMI, smoking, alcohol, education, physical activity, race/ethnicity, marital status
		Low-quality CHO	UFA	3%	0.988 (0.984, 0.992)	
		Low-quality CHO	SFA	3%	1.036 (1.029, 1.043)	
		Low-quality CHO	AP	3%	0.989 (0.984, 0.994)	
		Low-quality CHO	PP	3%	0.901 (0.889, 0.913)	
		SFA	Low-quality CHO	3%	0.967 (0.964, 0.970)	
		SFA	High-quality CHO	3%	0.965 (0.962, 0.968)	
		SFA	AP	3%	0.965 (0.960, 0.970)	
		SFA	PP	3%	0.897 (0.886, 0.909)	
Zhou, 2022 (35)	CHNS, China	CHO	PRO	10.1%	1.32 (1.10, 1.59)	Age, sex, energy, BMI, smoking, alcohol, SBP, education levels, urban or rural residents, regions, occupations, physical activity, insoluble fiber intake, sodium intake, potassium intake
				11.1%	1.00 (reference)	
				12.1%	1.19 (0.98, 1.44)	
				13.3%	1.19 (0.97, 1.47)	
				14.7%	1.37 (1.07, 1.77)	
Zhuang, 2019a (36)	NIH-AARP	SFA	TFA	2%	1.03 (1.01, 1.05)	Age, sex, energy, BMI, smoking, alcohol, education, marital status, household income, physical activity, aspirin use, history of hypertension, history of hypercholesterolemia, perceived health condition, history of heart disease, stroke, diabetes, cancer at baseline, hormones use for women, multi-vitamin use, race
		SFA	MUFA	5%	0.84 (0.80, 0.87)	
		SFA	Animal MUFA	5%	0.92 (0.87, 0.98)	
		SFA	Plant MUFA	5%	0.85 (0.82, 0.89)	
		SFA	PUFA	5%	0.82 (0.81, 0.84)	
		SFA	n-3 PUFA	0.3%	0.99 (0.96, 1.01)	
		SFA	n-6 PUFA	2%	0.92 (0.91, 0.93)	
		CHO	SFA	1-SD	1.09 (1.08, 1.10)	
		CHO	TFA	1-SD	1.01 (1.00, 1.02)	
		CHO	MUFA	1-SD	1.00 (0.99, 1.02)	
		CHO	Animal MUFA	1-SD	1.05 (1.04, 1.07)	

		CHO	Plant MUFA	1-SD	0.98 (0.97, 0.99)	
		CHO	PUFA	1-SD	0.98 (0.97, 0.98)	
		CHO	n-3 PUFA	1-SD	1.00 (0.99, 1.01)	
		CHO	n-6 PUFA	1-SD	0.97 (0.96, 0.98)	
Zhuang, 2019b (37) (men)[c]	CHNS, China	CHO	SFA	2.6%	1.00 (reference)	Age, energy, BMI, smoking, alcohol, education, income, physical activity, baseline hypertension, baseline diabetes, marital status, residence, location
				5.1%	0.91 (0.70, 1.17)	
				7.3%	0.93 (0.68, 1.28)	
				10.9%	0.95 (0.62, 1.47)	
		PUFA	SFA	1%	1.03 (0.94, 1.13)	
		MUFA	SFA	1%	1.30 (1.14, 1.48)	
Zhuang, 2019b (37) (women)[c]		CHO	SFA	2.5%	1.00 (reference)	Age, energy, BMI, smoking, alcohol, education, income, physical activity, baseline hypertension, baseline diabetes, marital status, residence, location
				4.9%	1.14 (0.86, 1.52)	
				7.1%	1.20 (0.84, 1.70)	
				10.8%	1.65 (1.03, 2.62)	
		PUFA	SFA	1%	0.95 (0.86, 1.04)	
		MUFA	SFA	1%	1.07 (0.91, 1.27)	

AF animal fat; animal MUFA monounsaturated fatty acids of animal origin; A-oil animal oil; AP animal protein; BMI body mass index; CHAMP Concord Health Ageing in Men Project; CHNS Chinese Health and Nutrition Survey; CHO carbohydrates; CI confidence interval; d day; EPIC European Prospective Investigation into Cancer and Nutrition; FFQ food frequency questionnaire; g/d grams/day; HEALS Health Effects of Arsenic Longitudinal Study; HPFS Health Professionals Follow-Up Study; HR hazard ratio; HRT hormone replacement therapy; InCHIANTI Invecchiare in Chianti aging in the Chianti area; IWHS Iowa Women's Health Study; JACC Japan Collaborative Cohort; JPHC Japan Public Health Center–based Prospective Cohort; kcal kilocalories; KIHD Kuopio Ischaemic Heart Disease Risk Factor Study; KoGES Korean Genome and Epidemiology Study; M men; MMSE Mini-Mental State Examination; MUFA monounsaturated fatty acids; n-3 PUFA n-3 polyunsaturated fatty acids; n-6 PUFA n-6 polyunsaturated fatty acids; NCS Norwegian Counties Study; NHANES National Health and Nutrition Examination Survey; NHANES III National Health and Nutrition Examination Survey III; NHS Nurses Health Study; NIH-AARP National Institutes of Health - American Association of Retired Persons Diet and Health Study; NR not reported; PHFO partially hydrogenated fish oil; PHVO partially hydrogenated vegetable oil; plant MUFA monounsaturated fatty acids of plant origin; P-oil oil from plant origin; PP plant protein; PREDIMED PREvención con DIeta MEDiterránea; PRO protein; PUFA polyunsaturated fatty acid; PURE Prospective Urban Rural Epidemiology; rTFA ruminant trans- fatty acids; SD standard deviation; SFA saturated fatty acid; SUN Seguimiento Universidad de Navarra; T2D type 2 diabetes; TFA trans- fatty acids; UFA unsaturated fatty acids; UKB United Kingdom Biobank; ULSAM Uppsala Longitudinal Study of Adult Men; W women; WHI Women's Health Initiative; WHR waist-to-hip ratio;

[a] Mono-/Disaccharides, refined CHO, sugar, liquid sugars, low-quality CHO were used to form the node “low-quality CHO / Mono-/ Disaccharides” for the carbohydrate-origin subnetwork; other CHO, complex CHO, starch, high-quality CHO were used to form the node “high-quality CHO / Polysaccharides” for the carbohydrate-origin subnetwork

[b] The adjustments for remaining macronutrients according to a substitution model were reported for every analysis (eg, for a substitution of CHO for PRO: fat was adjusted; for a substitution of MUFA for CHO: PRO, SFA, PUFA (and TFA) were adjusted, for a substitution of AP for CHO: PP and fat were adjusted, etc)

[c] The estimates for men and women were pooled

[d] The estimates are risk ratios; Only the confidence interval for the highest quintile was specified in the study; the confidence intervals for the 2nd – 4th quintile were transferred on this basis.

[e] The estimates TFA from PHFO, TFA from PHVO, rTFA (men) and rTFA (women) were pooled

[f] The estimates for different age categories were pooled

Supplemental table 4: Reasons for exclusion of specific comparisons from the network meta-analysis

Author, year	Cohort, country	↓ Nutrient[a]	↑ Replacement[a]	Unit[b]	Hazard ratio (95% CI)	Reason for exclusion
Chen, 2020 (5)	Rotterdam, Netherlands	FAT	AP	5%	1.16 (1.02, 1.33)	No matching network, the protein-origin subnetwork was analyzed using fatty acids as comparison and not total fat
		FAT	PP	5%	1.04 (0.85, 1.27)	No matching network, the protein-origin subnetwork was analyzed using fatty acids as comparison and not total fat
Guasch Ferre, 2015 (11)	PREDIMED, Spain	CHO	TFA	5%	45 (0.16, 12995.85)	Very high inconsistency of the effect estimate
Hernandez-Alonso, 2016 (13)	PREDIMED, Spain	FAT	AP	5%	1.42 (1.05, 1.91)	No matching network, the protein-origin subnetwork was analyzed using fatty acids as comparison and not total fat
		FAT	PP	5%	1.72 (0.74, 3.99)	No matching network, the protein-origin subnetwork was analyzed using fatty acids as comparison and not total fat
Laguna, 2021 (19)	PREDIMED, Spain	Liquid sugars	Total solid sugar	5%	0.77 (0.57, 1.04)	No matching network, no suitable node for the carbohydrate-origin subnetwork
Li, 2022 (21)	NHANES, USA	Low-quality CHO	UFA	5%	0.78 (0.65, 0.95)	No matching network, the carbohydrate-origin subnetwork was analyzed using PUFA and MUFA as opposed to UFA
		SFA	Low-quality CHO	5%	0.83 (0.60, 1.15)	Comparison was reported twice, overlap[c]

Zhao, 2023 (34)	NIH-AARP, USA	Low-quality CHO	High quality CHO	5%	0.99 (0.98, 0.99)	Very high inconsistency of the effect estimate
		Low-quality CHO	UFA	5%	0.98 (0.97, 0.99)	No matching network, the carbohydrate-origin subnetwork was analyzed using PUFA and MUFA as opposed to UFA
		Low-quality CHO	SFA	5%	1.06 (1.05, 1.07)	Comparison was reported twice in this study, overlap[c]
Zhuang 2019b (37)	CHNS, China	MUFA	SFA	5%	2.56 (1.53, 4.26)	Overlap[c] with study by Mao et al. (22)

AP animal protein; CHNS Chinese Health and Nutrition Survey; CHO carbohydrates; CI confidence interval; HR hazard ratio; MUFA monounsaturated fatty acids; NHANES National Health and Nutrition Examination Survey; NIH-AARP National Institutes of Health - American Association of Retired Persons Diet and Health Study; PP plant protein; PREDIMED PREvención con DIeta MEDiterránea; PUFA polyunsaturated fatty acid; SFA saturated fatty acid; TFA trans- fatty acids; UFA unsaturated fatty acids;

[a] Mono-/Disaccharides, refined CHO, sugar, liquid sugars, low-quality CHO were used to form the node “low-quality CHO / Mono-/ Disaccharides” for the carbohydrate-origin subnetwork; other CHO, complex CHO, starch, high-quality CHO were used to form the node “high-quality CHO / Polysaccharides” for the carbohydrate-origin subnetwork

[b] This evaluation was conducted after harmonizing all substitutions to a 5% energy level, according to Greenland and Longnecker method (38) (if estimates were presented per quantiles/unit of intake/exchange), or extrapolation (eg, from 1% to 5%)

[c] If there was an overlap, we chose the most conservative estimate

Supplemental table 5: League table for the overall macronutrient network (5% isocaloric energy substitution) for the outcome all-cause mortality

**FAT**	0.97 [0.95, 1.00]	0.99 [0.96, 1.02]
0.97 [0.96, 1.00]	**CHO**	1.01 [0.99, 1.03]
0.98 [0.96, 1.01]	1.01 [0.99, 1.03]	**PRO**

CHO carbohydrates; FAT fat; HR hazard ratio; PRO protein
The HRs are established by the replacement of the lower nutrient with the one above, eg, the direct and network HR for ↑ FAT (increase) vs. ↓ CHO (decrease) are displayed in the first row second column (HR: 0.97, 95% CI: 0.95, 0.99) and second row first column, respectively.
The upper triangle (marked blue) displays the HRs and 95% CI from direct comparison; the lower triangle (marked yellow) displays the HRs and 95% CI established from network meta-analysis.
For the upper triangle, HRs < 1 favor the row-defining treatment. For the lower triangle, HRs < 1 favor the column-defining treatment
The heterogeneity standard deviation (τ) was estimated at 0.03.

Supplemental table 6: GRADE assessment of all-cause mortality for the overall macronutrient network (5% isocaloric energy substitution);
Number of participants (n= 1 572 571) and number of mortality events (n= 239 450)

		Direct evidence			Indirect evidence		Network Meta-Analysis	
**Comparison**	**N studies**	**Proportion direct evidence**	**HR (95% CI)**	**Certainty of evidence**	**HR (95% CI)**	**Certainty of evidence**	**HR (95% CI)**	**Certainty of evidence**
**↑ FAT**								
**↓ CHO**	14	93	0.97 [0.95, 1.00]	⊕⊕⊕◯[1]	0.98 [0.91, 1.06]	⊕⊕◯◯	0.97 [0.96, 1.00]	⊕⊕⊕◯
**↓ PRO**	9	68	0.99 [0.96, 1.02]	⊕⊕◯◯[1,2]	0.97 [0.93, 1.01]	⊕⊕◯◯	0.98 [0.96, 1.01]	⊕⊕◯◯
**↑ CHO**								
**↓ PRO**	19	94	1.01 [0.99, 1.03]	⊕⊕◯◯[1,2]	1.04 [0.96, 1.12]	⊕⊕◯◯	1.01 [0.99, 1.03]	⊕⊕◯◯

⊕⊕⊕⊕ High; ⊕⊕⊕◯ Moderate; ⊕⊕◯◯ Low; ⊕◯◯◯ Very low; ↑ increase; ↓ decrease
95% CI 95% confidence interval; CHO carbohydrates; FAT fat, GRADE Grading of Recommendations Assessment, Development and Evaluations; HR hazard ratio; PRO protein; RoB risk of bias;
[1] downgraded by 1 level for RoB: less than 2/3 of the studies (and their contributing weight) were rated with a low RoB, and less than 2/3 of the studies were rated with a high RoB, OR more than 2/3 of the studies (and their contributing weight) were rated with a high RoB, but the effect estimate in the subgroup analysis, excluding studies with a high RoB, was robust.
[2] downgraded by 1 level for inconsistency: The point estimates differ substantially between primary studies, and the corresponding 95% CI overlap only minimally or not at all. We found no clinical or methodological explanation for this inconsistency

Supplemental table 7: League table for the fatty acids expanded network (5% isocaloric energy substitution) for the outcome all-cause mortality

**PUFA**	0.92 [0.86, 0.99]	0.86 [0.80, 0.92]	0.64 [0.56, 0.73]	0.90 [0.84, 0.96]	0.99 [0.86, 1.14]
0.94 [0.88, 1.01]	**MUFA**	0.91 [0.86, 0.97]	0.74 [0.65, 0.84]	0.94 [0.88, 1.01]	0.95 [0.82, 1.09]
0.86 [0.81, 0.91]	0.91 [0.86, 0.97]	**SFA**	0.85 [0.75, 0.97]	1.06 [1.00, 1.13]	1.01 [0.91, 1.13]
0.75 [0.67, 0.84]	0.80 [0.72, 0.89]	0.87 [0.78, 0.97]	**TFA**	1.18 [1.05, 1.32]	1.04 [0.60, 1.82]
0.90 [0.84, 0.95]	0.95 [0.90, 1.01]	1.04 [0.99, 1.11]	1.20 [1.08, 1.33]	**CHO**	0.99 [0.86, 1.13]
0.91 [0.82, 1.01]	0.96 [0.87, 1.07]	1.06 [0.96, 1.16]	1.21 [1.05, 1.39]	1.01 [0.92, 1.12]	**PRO**

CHO carbohydrates; HR hazard ratio; MUFA monounsaturated fatty acids; PRO protein; PUFA polyunsaturated fatty acids; SFA saturated fatty acids; TFA trans-fatty acids;
The HRs are established by the replacement of the lower nutrient with the one above, eg, the direct and network HR for ↑ PUFA (increase) vs. ↓ MUFA (decrease) are displayed in the first row second column and second row first column, respectively.
The upper triangle (marked blue) displays the HRs and 95% CI from direct comparison; the lower triangle (marked yellow) displays the HRs and 95% CI established from network meta-analysis.
For the upper triangle, HRs < 1 favor the row-defining treatment. For the lower triangle, HRs < 1 favor the column-defining treatment
The heterogeneity standard deviation (τ) was estimated at 0.09.

Supplemental table 8: League table for the MUFA-origin network (5% isocaloric energy substitution) for the outcome all-cause mortality

**Plant MUFA**	0.81 [0.76, 0.85]	0.85 [0.80, 0.90]	0.78 [0.66, 0.92]	0.90 [0.85, 0.95]
0.81 [0.76, 0.85]	**Animal MUFA**	1.05 [0.98, 1.12]	1.01 [0.85, 1.20]	1.12 [1.05, 1.19]
0.85 [0.80, 0.90]	1.05 [0.99, 1.12]	**SFA**	0.92 [0.77, 1.11]	1.06 [1.00, 1.13]
0.79 [0.67, 0.94]	0.99 [0.84, 1.16]	0.94 [0.79, 1.11]	**TFA**	1.12 [0.94, 1.32]
0.90 [0.85, 0.95]	1.12 [1.05, 1.18]	1.06 [1.00, 1.13]	1.13 [0.96, 1.33]	**CHO**

Animal MUFA monounsaturated fatty acids of animal origin; CHO carbohydrates; HR hazard ratio; Plant MUFA monounsaturated fatty acids of plant origin; SFA saturated fatty acids; TFA trans-fatty acids;

The HRs are established by the replacement of the lower nutrient with the one above, eg, the direct and network HR for ↑ plant MUFA (increase) vs. ↓ animal MUFA (decrease) are displayed in the first row second column and second row first column, respectively.

The upper triangle (marked blue) displays HRs and 95% CI from direct comparison; the lower triangle (marked yellow) displays the HRs and 95% CI established from network meta-analysis.

For the upper triangle, HRs < 1 favor the row-defining treatment. For the lower triangle, HRs < 1 favor the column-defining treatment

The heterogeneity standard deviation (τ) was estimated at 0.04.

Supplemental table 9: League table for the PUFA-origin network (5% isocaloric energy substitution) for the outcome all-cause mortality

**n-3 PUFA**	0.92 [0.74, 1.13]	0.81 [0.57, 1.15]	0.70 [0.58, 0.86]	0.83 [0.43, 1.58]	0.82 [0.66, 1.01]	0.83 [0.59, 1.17]
0.84 [0.70, 1.01]	**n-6 PUFA**	0.96 [0.76, 1.21]	0.86 [0.77, 0.97]	0.98 [0.54, 1.78]	0.85 [0.77, 0.94]	0.99 [0.81, 1.22]
0.75 [0.58, 0.97]	0.90 [0.73, 1.10]	**MUFA**	0.98 [0.78, 1.24]	1.02 [0.56, 1.86]	0.96 [0.76, 1.21]	1.03 [0.82, 1.30]
0.69 [0.57, 0.83]	0.82 [0.74, 0.92]	0.91 [0.74, 1.12]	**SFA**	1.04 [0.58, 1.88]	1.06 [0.92, 1.22]	1.05 [0.85, 1.29]
0.77 [0.42, 1.41]	0.92 [0.51, 1.64]	1.02 [0.56, 1.86]	1.12 [0.62, 2.00]	**TFA**	0.94 [0.52, 1.69]	1.01 [0.56, 1.82]
0.72 [0.59, 0.86]	0.85 [0.77, 0.94]	0.95 [0.77, 1.17]	1.04 [0.92, 1.17]	0.93 [0.52, 1.66]	**CHO**	1.08 [0.87, 1.32]
0.78 [0.61, 0.98]	0.93 [0.77, 1.11]	1.03 [0.82, 1.30]	1.13 [0.94, 1.35]	1.01 [0.56, 1.82]	1.09 [0.91, 1.30]	**PRO**

CHO carbohydrates; HR hazard ratio; MUFA monounsaturated fatty acids; n-3 PUFA n-3 polyunsaturated fatty acids; n-6 PUFA n-6 polyunsaturated fatty acids; PRO protein; SFA saturated fatty acids; TFA trans-fatty acids;
The HRs are established by the replacement of the lower nutrient with the one above, eg, the direct and network HR for ↑ n-3 PUFA (increase) vs. ↓ n-6 PUFA (decrease) are displayed in the first row second column and second row first column, respectively.
The upper triangle (marked blue) displays the HR's and 95% CI from direct comparison; the lower triangle (marked yellow) displays the HRs and 95% CI established from network meta-analysis.
For the upper triangle, HRs < 1 favor the row-defining treatment. For the lower triangle, HRs < 1 favor the column-defining treatment
The heterogeneity standard deviation (τ) was estimated at 0.10.

Supplemental table 10: League table for the fat-origin subnetwork (5% isocaloric energy substitution) for the outcome all-cause mortality

**PF**	0.98 [0.96, 1.00]	0.95 [0.93, 0.98]	1.03 [0.99, 1.07]
0.98 [0.96, 1.00]	**AF**	0.97 [0.95, 0.99]	1.04 [1.00, 1.08]
0.95 [0.93, 0.98]	0.97 [0.95, 1.00]	**CHO**	1.09 [1.05, 1.13]
1.03 [0.99, 1.07]	1.05 [1.01, 1.09]	1.08 [1.04, 1.12]	**PRO**

AF animal fat; CHO carbohydrates; HR hazard ratio; PF plant fat; PRO protein;
The HRs are established by the replacement of the lower nutrient with the one above, eg, the direct and network HR for ↑ PF (increase) vs. ↓ AF (decrease) are displayed in the first row second column and second row first column, respectively.
The upper triangle (marked blue) displays the HRs and 95% CI from direct comparison; the lower triangle (marked yellow) displays the HR and 95% CI established from network meta-analysis.
For the upper triangle, HRs < 1 favor the row-defining treatment. For the lower triangle, HRs < 1 favor the column-defining treatment
The heterogeneity standard deviation (τ) was estimated at 0.01.

Supplemental table 11: GRADE assessment of all-cause mortality for the fat-origin subnetwork (5% isocaloric energy substitution); Number of participants (n= 258 291) and number of mortality events (n= 17 471)

		Direct evidence			Indirect evidence		Network Meta-Analysis	
Comparison	N studies	Proportion direct evidence	HR (95% CI)	Certainty of evidence	HR (95% CI)	Certainty of evidence	HR (95% CI)	Certainty of evidence
↑ PF								
↓ AF	3	100	0.98 [0.96, 1.00]	⊕⊕◯◯[1]	NA	NA	0.98 [0.96, 1.00]	⊕⊕◯◯
↓ CHO	2	96	0.95 [0.93, 0.98]	⊕⊕◯◯[1]	0.88 [0.78, 0.99]	⊕⊕◯◯	0.95 [0.93, 0.98]	⊕⊕◯◯
↓ PRO	1	89	1.03 [0.99, 1.07]	⊕⊕◯◯[1]	1.02 [0.91, 1.14]	⊕⊕◯◯	1.03 [0.99, 1.07]	⊕◯◯◯[2]
↑ AF								
↓ CHO	2	96	0.97 [0.95, 0.99]	⊕⊕◯◯[1]	1.05 [0.92, 1.19]	⊕⊕◯◯	0.97 [0.95, 1.00]	⊕⊕◯◯
↓ PRO	1	92	1.04 [1.00, 1.08]	⊕⊕◯◯[1]	1.14 [1.00, 1.30]	⊕⊕◯◯	1.05 [1.01, 1.09]	⊕⊕◯◯

⊕⊕⊕⊕ High; ⊕⊕⊕◯ Moderate; ⊕⊕◯◯ Low; ⊕◯◯◯ Very low; ↑ increase; ↓ decrease

95% CI 95% confidence interval; AF animal fat; CHO carbohydrates; GRADE Grading of Recommendations Assessment, Development and Evaluations; HR hazard ratio; NA not applicable (the proportion of evidence was 100% for the direct estimate); PF plant fat; PRO protein; RoB risk of bias;

[1] downgraded by 2 levels for RoB: More than 2/3 of the studies (and their contributing weight) were rated with a high RoB. No subgroup analysis for RoB could be conducted to test to robustness of the effect estimates.

[2] downgraded by 1 level for imprecision: The 95% CI includes a RR/HR of 1 and the 95% CI is not narrow (maximal width of 0.05).

Supplemental table 12: League table for the protein-origin subnetwork (5% isocaloric energy substitution) for the outcome all-cause mortality

**PP**	0.87 [0.83, 0.91]	0.98 [0.88, 1.10]	1.02 [0.91, 1.15]	0.90 [0.84, 0.97]	0.93 [0.53, 1.64]	0.88 [0.85, 0.92]
0.87 [0.84, 0.91]	**AP**	1.01 [0.92, 1.10]	1.04 [0.95, 1.14]	0.99 [0.93, 1.05]	0.95 [0.55, 1.66]	1.00 [0.97, 1.03]
0.86 [0.80, 0.94]	0.99 [0.92, 1.07]	**PUFA**	1.05 [0.95, 1.17]	1.04 [0.95, 1.14]	0.93 [0.53, 1.63]	1.02 [0.94, 1.11]
0.92 [0.84, 1.00]	1.05 [0.96, 1.14]	1.06 [0.96, 1.17]	**MUFA**	0.98 [0.89, 1.08]	0.99 [0.56, 1.73]	0.95 [0.87, 1.04]
0.86 [0.82, 0.91]	0.99 [0.94, 1.04]	1.00 [0.92, 1.09]	0.95 [0.86, 1.03]	**SFA**	1.00 [0.57, 1.74]	0.97 [0.89, 1.05]
0.87 [0.50, 1.51]	0.99 [0.57, 1.73]	1.01 [0.58, 1.76]	0.95 [0.54, 1.66]	1.01 [0.58, 1.75]	**TFA**	0.97 [0.56, 1.69]
0.88 [0.84, 0.91]	1.00 [0.97, 1.03]	1.01 [0.94, 1.10]	0.96 [0.88, 1.04]	1.01 [0.96, 1.07]	1.01 [0.58, 1.75]	**CHO**

AP animal protein; CHO carbohydrates; HR hazard ratio; MUFA monounsaturated fatty acids; PP plant protein; PUFA polyunsaturated fatty acids; SFA saturated fatty acids; TFA trans-fatty acids;
The HRs are established by the replacement of the lower nutrient with the one above, eg, the direct and network HR for ↑ PP (increase) vs. ↓ AP (decrease) are displayed in the first row second column and second row first column, respectively.
The upper triangle (marked blue) displays the HRs and 95% CI from direct comparison; the lower triangle (marked yellow) displays the HRs and 95% CI established from network meta-analysis.
For the upper triangle, HRs < 1 favor the row-defining treatment. For the lower triangle, HRs < 1 favor the column-defining treatment
The heterogeneity standard deviation (τ) was estimated at 0.05.

Supplemental table 13: League table for the carbohydrate-origin subnetwork (5% isocaloric energy substitution) for the outcome all-cause mortality

**High-quality CHO**	0.98 [0.93, 1.03]	0.99 [0.91, 1.07]	1.04 [0.96, 1.13]	0.98 [0.93, 1.04]	0.93 [0.54, 1.60]	0.99 [0.94, 1.04]
0.97 [0.92, 1.02]	**Low-quality CHO**	1.01 [0.93, 1.09]	1.07 [0.99, 1.17]	1.00 [0.94, 1.06]	0.97 [0.56, 1.66]	1.02 [0.97, 1.08]
0.97 [0.90, 1.05]	1.01 [0.93, 1.08]	**PUFA**	1.03 [0.94, 1.14]	1.03 [0.94, 1.12]	0.86 [0.50, 1.48]	0.99 [0.91, 1.08]
1.02 [0.95, 1.11]	1.06 [0.98, 1.14]	1.05 [0.96, 1.15]	**MUFA**	0.98 [0.89, 1.07]	0.92 [0.53, 1.58]	0.97 [0.89, 1.06]
0.96 [0.91, 1.02]	1.00 [0.94, 1.05]	0.99 [0.91, 1.07]	0.94 [0.87, 1.02]	**SFA**	0.92 [0.53, 1.58]	1.02 [0.96, 1.09]
0.91 [0.53, 1.55]	0.93 [0.55, 1.60]	0.93 [0.54, 1.60]	0.89 [0.52, 1.52]	0.94 [0.55, 1.61]	**TFA**	1.11 [0.65, 1.92]
0.99 [0.94, 1.04]	1.02 [0.97, 1.08]	1.02 [0.94, 1.10]	0.97 [0.89, 1.05]	1.03 [0.97, 1.09]	1.09 [0.64, 1.88]	**PRO**

CHO carbohydrates; HR hazard ratio; MUFA monounsaturated fatty acids; PRO protein; PUFA polyunsaturated fatty acids; SFA saturated fatty acids; TFA trans-fatty acids;
The HRs are established by the replacement of the lower nutrient with the one above, eg, the direct and network HR for ↑ high-quality CHO (increase) vs. ↓ low-quality CHO (decrease) are displayed in the first row second column and second row first column, respectively.
The upper triangle (marked blue) displays the HRs and 95% CI from direct comparison; the lower triangle (marked yellow) displays the HRs and 95% CI established from network meta-analysis.
For the upper triangle, HRs < 1 favor the row-defining treatment. For the lower triangle, HRs < 1 favor the column-defining treatment
The heterogeneity standard deviation (τ) was estimated at 0.05.

Supplemental table 14: GRADE assessment of all-cause mortality for the carbohydrate-origin subnetwork (5% isocaloric energy substitution);
Number of participants (n= 633 358) and number of mortality events (n= 185 350)

		Direct evidence			Indirect evidence		Network Meta-Analysis	
Comparison	N studies	Proportion direct evidence	HR (95% CI)	Certainty of evidence	HR (95% CI)	Certainty of evidence	HR (95% CI)	Certainty of evidence
↑ High-quality CHO								
↓ Low-quality CHO	5	94	0.98 [0.93, 1.03]	⊕⊕◯◯[2]	0.85 [0.69, 1.03]	⊕⊕◯◯	0.97 [0.92, 1.02]	⊕◯◯◯[4]
↓ PUFA	3	89	0.99 [0.91, 1.07]	⊕◯◯◯[2,3]	0.86 [0.69, 1.08]	⊕◯◯◯	0.97 [0.90, 1.05]	⊕◯◯◯[4]
↓ MUFA	3	88	1.04 [0.96, 1.13]	⊕⊕◯◯[2]	0.89 [0.72, 1.12]	⊕⊕◯◯	1.02 [0.95, 1.11]	⊕◯◯◯[4]
↓ SFA	5	93	0.98 [0.93, 1.04]	⊕⊕◯◯[1,3]	0.77 [0.63, 0.95]	⊕⊕◯◯	0.96 [0.91, 1.02]	⊕◯◯◯[4]
↓ TFA	2	99	0.93 [0.54, 1.60]	⊕⊕◯◯[2]	0.03 [0.00, 13.97]	⊕⊕◯◯	0.91 [0.53, 1.55]	⊕◯◯◯[4]
↓ PRO	4	98	0.99 [0.94, 1.04]	⊕◯◯◯[2,3]	0.96 [0.71, 1.30]	⊕⊕◯◯	0.99 [0.94, 1.04]	⊕◯◯◯[4]
↑ Low-quality CHO								
↓ PUFA	3	90	1.01 [0.93, 1.09]	⊕◯◯◯[2,3]	1.00 [0.78, 1.27]	⊕◯◯◯	1.01 [0.93, 1.08]	⊕◯◯◯[4]
↓ MUFA	3	90	1.07 [0.99, 1.17]	⊕⊕◯◯[2]	0.92 [0.72, 1.17]	⊕⊕◯◯	1.06 [0.98, 1.14]	⊕◯◯◯[4]
↓ SFA	5	97	1.00 [0.94, 1.06]	⊕⊕◯◯[1,3]	0.93 [0.68, 1.28]	⊕⊕◯◯	1.00 [0.94, 1.05]	⊕◯◯◯[4]
↓ TFA	2	99	0.97 [0.56, 1.66]	⊕⊕◯◯[2]	0.01 [0.00, 5.61]	⊕⊕◯◯	0.93 [0.55, 1.60]	⊕◯◯◯[4]
↓ PRO	4	89	1.02 [0.97, 1.08]	⊕⊕◯◯[1,3]	1.04 [0.88, 1.22]	⊕◯◯◯	1.02 [0.97, 1.08]	⊕◯◯◯[4]

⊕⊕⊕⊕ High; ⊕⊕⊕◯ Moderate; ⊕⊕◯◯ Low; ⊕◯◯◯ Very low; ↑ increase; ↓ decrease

95% CI 95% confidence interval; CHO carbohydrates; GRADE Grading of Recommendations Assessment, Development and Evaluations; HR hazard ratio; MUFA monounsaturated fatty acids; PRO protein, PUFA polyunsaturated fatty acids; RoB risk of bias; SFA saturated fatty acids; TFA trans-fatty acids

[1] downgraded by 1 level for RoB: less than 2/3 of the studies (and their contributing weight) were rated with a low RoB, and less than 2/3 of the studies were rated with a high RoB, OR more than 2/3 of the studies (and their contributing weight) were rated with a high RoB, but the effect estimate in the subgroup analysis, excluding studies with a high RoB, was robust.

[2] downgraded by 2 levels for RoB: More than 2/3 of the studies (and their contributing weight) were rated with a high RoB. No subgroup analysis for RoB could be conducted to test to robustness of the effect estimates.
[3] downgraded by 1 level for inconsistency: The point estimates differ substantially between primary studies, and the corresponding 95% CI overlap only minimally or not at all. We found no clinical or methodological explanation for this inconsistency.
[4] downgraded by 1 level for imprecision: The 95% CI includes a RR/HR of 1 and the 95% CI is not narrow (maximal width of 0.05).

Supplemental table 15: SIDE splitting approach comparing direct and indirect evidence for the overall macronutrient network (5% isocaloric energy substitution) for the outcome all-cause mortality

	Direct	Indirect	Ratio of HRs	p-value
**↑ FAT vs. ↓ CHO**	0.97 [0.95, 1.00]	0.98 [0.91, 1.06]	0.99 [0.92, 1.08]	0.89
**↑ FAT vs. ↓ PRO**	0.99 [0.96, 1.02]	0.97 [0.93, 1.01]	1.02 [0.97, 1.07]	0.49
**↑ CHO vs. ↓ PRO**	1.01 [0.99, 1.03]	1.04 [0.96, 1.12]	0.97 [0.89, 1.06]	0.49

CHO carbohydrates; FAT fat; HR hazard ratio; PRO protein; ↑ increase; ↓ decrease
Estimates are hazard ratios with corresponding 95% confidence interval

Supplemental table 16: SIDE splitting approach comparing direct and indirect evidence for the fatty acids expanded network (5% isocaloric energy substitution) for the outcome all-cause mortality

	Direct	Indirect	Ratio of HRs	p-value
**↑ PUFA vs. ↓ MUFA**	0.92 [0.86, 0.99]	1.11 [0.92, 1.35]	0.83 [0.67, 1.01]	0.06
**↑ PUFA vs. ↓ SFA**	0.86 [0.80, 0.92]	0.84 [0.69,1.03]	1.02 [0.83, 1.27]	0.83
**↑ PUFA vs. ↓ TFA**	0.64 [0.56, 0.73]	1.07 [0.88, 1.30]	0.60 [0.47, 0.75]	<0.001
**↑ PUFA vs. ↓ CHO**	0.90 [0.84, 0.96]	0.88 [0.74, 1.05]	1.02 [0.85, 1.24]	0.81
**↑ PUFA vs. ↓ PRO**	0.99 [0.86, 1.14]	0.82 [0.70, 0.95]	1.22 [0.99, 1.50]	0.06
**↑ MUFA vs. ↓ SFA**	0.91 [0.86, 0.97]	0.90 [0.75; 1.08]	1.01 [0.84, 1.23]	0.88
**↑ MUFA vs. ↓ TFA**	0.74 [0.65, 0.84]	0.95 [0.78, 1.16]	0.78 [0.62, 0.99]	0.04
**↑ MUFA vs. ↓ CHO**	0.94 [0.88, 1.01]	1.01 [0.86, 1.19]	0.93 [0.78, 1.11]	0.42
**↑ MUFA vs. ↓ PRO**	0.95 [0.82, 1.09]	0.98 [0.85; 1.14]	0.96 [0.78, 1.18]	0.69
**↑ SFA vs. ↓ TFA**	0.85 [0.75, 0.97]	0.92 [0.76, 1.12]	0.92 [0.73, 1.17]	0.51
**↑ SFA vs. ↓ CHO**	1.06 [1.00, 1.13]	0.95 [0.82; 1.11]	1.11 [0.94, 1.31]	0.22
**↑ SFA vs. ↓ PRO**	1.01 [0.91, 1.13]	1.23 [1.01, 1.51]	0.82 [0.65, 1.03]	0.09
**↑ TFA vs. ↓ CHO**	1.18 [1.05, 1.32]	1.30 [0.98, 1.72]	0.91 [0.67, 1.23]	0.54
**↑ TFA vs. ↓ PRO**	1.04 [0.60, 1.82]	1.22 [1.06; 1.41]	0.85 [0.48, 1.52]	0.59

CHO carbohydrates; HR hazard ratio; MUFA monounsaturated fatty acids; PRO protein; PUFA polyunsaturated fatty acids; SFA saturated fatty acids; TFA trans-fatty acids; ↑ increase; ↓ decrease
Estimates are hazard ratios with corresponding 95% confidence interval

Supplemental table 17: SIDE splitting approach comparing direct and indirect evidence for the MUFA-origin network (5% isocaloric energy substitution) for the outcome all-cause mortality

	Direct	Indirect	Ratio of HRs	p-value
**↑ Plant MUFA vs. ↓ Animal MUFA**	0.81 [0.76, 0.85]	NA	NA	NA
**↑ Plant MUFA vs. ↓ SFA**	0.85 [0.80, 0.90]	0.88 [0.64, 1.22]	0.96 [0.69, 1.33]	0.80
**↑ Plant MUFA vs. ↓ TFA**	0.78 [0.66, 0.92]	1.25 [0.57, 2.74]	0.62 [0.28, 1.39]	0.25
**↑ Plant MUFA vs. ↓ CHO**	0.90 [0.85, 0.95]	1.11 [0.53, 2.34]	0.81 [0.38, 1.70]	0.57
**↑ Animal MUFA vs. ↓ SFA**	1.05 [0.98, 1.12]	1.20 [0.86, 1.68]	0.87 [0.62, 1.23]	0.44
**↑ Animal MUFA vs. ↓ TFA**	1.01 [0.85, 1.20]	0.75 [0.42, 1.34]	1.35 [0.74, 2.47]	0.33
**↑ Animal MUFA vs. ↓ CHO**	1.12 [1.05, 1.19]	1.07 [0.55, 2.12]	1.04 [0.53, 2.05]	0.91

animal MUFA monounsaturated fatty acids of animal origin; CHO carbohydrates; HR hazard ratio; MUFA monounsaturated fatty acids; NA not applicable (the proportion of evidence was 100% for the direct estimate); plant MUFA monounsaturated fatty acids of plant origin; SFA saturated fatty acids; TFA trans-fatty acids; ↑ increase; ↓ decrease
Estimates are hazard ratios with corresponding 95% confidence interval

Supplemental table 18: SIDE splitting approach comparing direct and indirect evidence for the PUFA-origin network (5% isocaloric energy substitution) for the outcome all-cause mortality

	Direct	Indirect	Ratio of HRs	p-value
**↑ n-3 PUFA vs. ↓ n-6 PUFA**	0.92 [0.74, 1.13]	0.60 [0.40, 0.90]	1.53 [0.97, 2.43]	0.07
**↑ n-3 PUFA vs. ↓ MUFA**	0.81 [0.57, 1.15]	0.70 [0.49, 1.01]	1.15 [0.69, 1.92]	0.58
**↑ n-3 PUFA vs. ↓ SFA**	0.70 [0.58, 0.86]	0.59 [0.35, 0.99]	1.20 [0.69, 2.11]	0.52
**↑ n-3 PUFA vs. ↓ TFA**	0.83 [0.43, 1.58]	0.51 [0.10, 2.51]	1.62 [0.29, 9.11]	0.58
**↑ n-3 PUFA vs. ↓ CHO**	0.82 [0.66, 1.01]	0.38 [0.24, 0.59]	2.17 [1.31, 3.58]	0.002
**↑ n-3 PUFA vs. ↓ PRO**	0.83 [0.59, 1.17]	0.73 [0.53, 1.01]	1.14 [0.71, 1.82]	0.58
**↑ n-6 PUFA vs. ↓ MUFA**	0.96 [0.76, 1.21]	0.70 [0.45, 1.08]	1.38 [0.84, 2.26]	0.20
**↑ n-6 PUFA vs. ↓ SFA**	0.86 [0.77, 0.97]	0.55 [0.39, 0.78]	1.56 [1.09, 2.23]	0.02
**↑ n-6 PUFA vs. ↓ TFA**	0.98 [0.54, 1.78]	0.12 [0.005, 2.95]	8.38 [0.32, 222.36]	0.20
**↑ n-6 PUFA vs. ↓ CHO**	0.85 [0.77, 0.94]	0.89 [0.55, 1.42]	0.96 [0.59, 1.55]	0.86
**↑ n-6 PUFA vs. ↓ PRO**	0.99 [0.81, 1.22]	0.76 [0.54, 1.08]	1.30 [0.87, 1.95]	0.20

CHO carbohydrates; HR hazard ratio; MUFA monounsaturated fatty acids; n-3 PUFA n-3 polyunsaturated fatty acids; n-6 PUFA n-6 polyunsaturated fatty acids; PRO protein; PUFA polyunsaturated fatty acids; SFA saturated fatty acids; TFA trans-fatty acids; ↑ increase; ↓ decrease
Estimates are hazard ratios with corresponding 95% confidence interval

Supplemental table 19: SIDE splitting approach comparing direct and indirect evidence for the fat-origin subnetwork (5% isocaloric energy substitution) for the outcome all-cause mortality

	Direct	Indirect	Ratio of HRs	p-value
**↑ PF vs. ↓ AF**	0.98 [0.96, 1.00]	NA	NA	NA
**↑ PF vs. ↓ CHO**	0.95 [0.93, 0.98]	0.88 [0.78, 0.99]	1.08 [0.96, 1.22]	0.19
**↑ PF vs. ↓ PRO**	1.03 [0.99, 1.07]	1.02 [0.91, 1.14]	1.01 [0.89, 1.14]	0.85
**↑ AF vs. ↓ CHO**	0.97 [0.95, 0.99]	1.05 [0.92, 1.19]	0.92 [0.81, 1.05]	0.23
**↑ AF vs. ↓ PRO**	1.04 [1.00, 1.08]	1.14 [1.00, 1.30]	0.91 [0.80, 1.05]	0.19

AF animal fat; CHO carbohydrates; HR hazard ratio; NA not applicable (the proportion of evidence was 100% for the direct estimate); PF plant fat; PRO protein; ↑ increase; ↓ decrease
Estimates are hazard ratios with corresponding 95% confidence interval

Supplemental table 20: SIDE splitting approach comparing direct and indirect evidence for the protein-origin subnetwork (5% isocaloric energy substitution) for the outcome all-cause mortality

	Direct	Indirect	Ratio of HRs	p-value
**↑ PP vs. ↓ AP**	0.87 [0.83, 0.91]	0.88 [0.81, 0.97]	0.99 [0.89, 1.09]	0.82
**↑ PP vs. ↓ PUFA**	0.98 [0.88, 1.10]	0.76 [0.68, 0.85]	1.30 [1.10, 1.53]	0.002
**↑ PP vs. ↓ MUFA**	1.02 [0.91, 1.15]	0.80 [0.71, 0.91]	1.27 [1.07, 1.51]	0.01
**↑ PP vs. ↓ SFA**	0.90 [0.84, 0.97]	0.81 [0.75, 0.89]	1.11 [0.99, 1.24]	0.08
**↑ PP vs. ↓ TFA**	0.93 [0.53, 1.64]	0.11 [0.01, 2.31]	8.34 [0.38, 181.11]	0.18
**↑ PP vs. ↓ CHO**	0.88 [0.85, 0.92]	0.82 [0.73, 0.93]	1.07 [0.94, 1.23]	0.29
**↑ AP vs. ↓ PUFA**	1.01 [0.92, 1.10]	0.92 [0.79, 1.08]	1.09 [0.91, 1.31]	0.34
**↑ AP vs. ↓ MUFA**	1.04 [0.95, 1.14]	1.06 [0.88, 1.28]	0.98 [0.80, 1.21]	0.86
**↑ AP vs. ↓ SFA**	0.99 [0.93, 1.05]	0.99 [0.90, 1.09]	1.00 [0.89, 1.12]	0.94
**↑ AP vs. ↓ TFA**	0.95 [0.55, 1.66]	87.09 [0.31, 24507.53]	0.01 [0.00, 3.16]	0.12
**↑ AP vs. ↓ CHO**	1.00 [0.97, 1.03]	1.01 [0.88, 1.17]	0.99 [0.85, 1.15]	0.88

95% CI 95% confidence interval; AP animal protein; CHO carbohydrates; HR hazard ratio; MUFA monounsaturated fatty acids; n-3 PUFA n-3 polyunsaturated fatty acids; n-6 PUFA n-6 polyunsaturated fatty acids; PP plant protein; PRO protein; PUFA polyunsaturated fatty acids; SFA saturated fatty acids; TFA trans-fatty acids; ↑ increase; ↓ decrease
Estimates are hazard ratios with corresponding 95% confidence interval

Supplemental table 21: SIDE splitting approach comparing direct and indirect evidence for the carbohydrate-origin subnetwork (5% isocaloric energy substitution) for the outcome all-cause mortality

	Direct	Indirect	Ratio of HRs	p-value
**↑ High-quality CHO vs. ↓ low-quality CHO**	0.98 [0.93, 1.03]	0.85 [0.69, 1.03]	1.16 [0.94, 1.42]	0.17
**↑ High-quality CHO vs. ↓ PUFA**	0.99 [0.91, 1.07]	0.86 [0.69, 1.08]	1.14 [0.91, 1.44]	0.26
**↑ High-quality CHO vs. ↓ MUFA**	1.04 [0.96, 1.13]	0.89 [0.72, 1.12]	1.17 [0.92, 1.48]	0.20
**↑ High-quality CHO vs. ↓ SFA**	0.98 [0.93, 1.04]	0.77 [0.63, 0.95]	1.28 [1.03, 1.58]	0.03
**↑ High-quality CHO vs. ↓ TFA**	0.93 [0.54, 1.60]	0.03 [0.00, 13.97]	28.50 [0.07, 12491.81]	0.28
**↑ High-quality CHO vs. ↓ PRO**	0.99 [0.94, 1.04]	0.96 [0.71, 1.30]	1.03 [0.76, 1.41]	0.84
**↑ Low-quality CHO vs. ↓ PUFA**	1.01 [0.93, 1.09]	1.00 [0.78, 1.27]	1.01 [0.78, 1.30]	0.95
**↑ Low-quality CHO vs. ↓ MUFA**	1.07 [0.99, 1.17]	0.92 [0.72, 1.17]	1.17 [0.90, 1.52]	0.24
**↑ Low-quality CHO vs. ↓ SFA**	1.00 [0.94, 1.06]	0.93 [0.68, 1.28]	1.07 [0.77, 1.48]	0.68
**↑ Low-quality CHO vs. ↓ TFA**	0.97 [0.56, 1.66]	0.01 [0.00, 5.61]	97.65 [0.17, 56693.01]	0.16
**↑ Low-quality CHO vs. ↓ PRO**	1.02 [0.97, 1.08]	1.04 [0.88, 1.22]	0.98 [0.83, 1.17]	0.85

CHO carbohydrates; HR hazard ratio; MUFA monounsaturated fatty acids; PRO protein; PUFA polyunsaturated fatty acids; SFA saturated fatty acids; TFA trans-fatty acids; ↑ increase; ↓ decrease
Estimates are hazard ratios with corresponding 95% confidence interval

Supplemental table 22: Results of the design by treatment test (global test for statistical inconsistency in the network meta-analysis for the outcome all-cause mortality)

	**p-value**
**Overall macronutrient network**	p = 0.28
**Fatty acids expanded network**	p = 0.005
**MUFA-origin network**	p = 0.06
**PUFA-origin network**	p < 0.001
**Fat-origin subnetwork**	p = 0.20
**Protein-origin subnetwork**	p < 0.001
**Carbohydrate-origin subnetwork**	p < 0.001

Supplemental table 23: P-scores for the overall macronutrient network (5% isocaloric energy substitution) for the outcome all-cause mortality

	P-score
**FAT**	0.95
**PRO**	0.45
**CHO**	0.10

CHO carbohydrates; PRO protein

Supplemental table 24: P-scores for the fatty acids expanded network (5% isocaloric energy substitution) for the outcome all-cause mortality

	P-score
**PUFA**	0.99
**MUFA**	0.75
**PRO**	0.55
**CHO**	0.48
**SFA**	0.24
**TFA**	0.00

CHO carbohydrates; MUFA monounsaturated fatty acids; PRO protein; PUFA polyunsaturated fatty acids; SFA saturated fatty acids; TFA trans-fatty acids;

Supplemental table 25: P-scores for the MUFA-origin network (5% isocaloric energy substitution) for the outcome all-cause mortality

	P-score
**Plant MUFA**	1.00
**CHO**	0.72
**SFA**	0.44
**TFA**	0.18
**Animal MUFA**	0.16

Animal MUFA monounsaturated fatty acids of animal origin; CHO carbohydrates; Plant MUFA monounsaturated fatty acids of plant origin; SFA saturated fatty acids; TFA trans-fatty acids;

Supplemental table 26: P-scores for the PUFA-origin network (5% isocaloric energy substitution) for the outcome all-cause mortality

	P-score
**n-3 PUFA**	0.96
**n-6 PUFA**	0.72
**PRO**	0.51
**TFA**	0.47
**MUFA**	0.42
**CHO**	0.27
**SFA**	0.15

CHO carbohydrates; MUFA monounsaturated fatty acids; n-3 PUFA n-3 polyunsaturated fatty acids; n-6 PUFA n-6 polyunsaturated fatty acids; PRO protein; SFA saturated fatty acids; TFA trans-fatty acids;

Supplemental table 27: P-scores for the fat-origin subnetwork (5% isocaloric energy substitution) for the outcome all-cause mortality

	P-score
**PRO**	0.97
**PF**	0.68
**AF**	0.34
**CHO**	0.00

AF animal fat; CHO carbohydrates; PF plant fat; PRO protein;

Supplemental table 28: P-scores for the protein-origin subnetwork (5% isocaloric energy substitution) for the outcome all-cause mortality

	P-score
**PP**	0.94
**MUFA**	0.68
**TFA**	0.46
**CHO**	0.43
**AP**	0.40
**PUFA**	0.31
**SFA**	0.29

AP animal protein; CHO carbohydrates; MUFA monounsaturated fatty acids; PP plant protein; PUFA polyunsaturated fatty acids; SFA saturated fatty acids; TFA trans-fatty acids;

Supplemental table 29: P-scores for the carbohydrate-origin subnetwork (5% isocaloric energy substitution) for the outcome all-cause mortality

	P-score
**MUFA**	0.81
**High-quality CHO**	0.68
**PRO**	0.58
**PUFA**	0.41
**TFA**	0.38
**Low-quality CHO**	0.33
**SFA**	0.30

CHO carbohydrates; MUFA monounsaturated fatty acids; PRO protein; PUFA polyunsaturated fatty acids; SFA saturated fatty acids; TFA trans-fatty acids;

Supplemental table 30: Subgroup analysis for the overall macronutrient network, including American cohort studies (5% isocaloric energy substitution);
Number of participants (n= 771 011) and number of mortality events (n= 174 566)

**FAT**	0.98 [0.95, 1.01]	1.02 [0.97, 1.06]
0.98 [0.96, 1.01]	**CHO**	1.02 [0.99, 1.04]
1.00 [0.97, 1.03]	1.02 [1.00, 1.04]	**PRO**

CHO carbohydrates; CI confidence interval; FAT fat; HR hazard ratio; PRO protein
The HRs are established by the replacement of the lower nutrient with the one above, eg, the direct and network HR for ↑ FAT (increase) vs. ↓ CHO (decrease) are displayed in the first row second column (HR: 0.97, 95% CI: 0.95, 0.99) and second row first column, respectively.
The upper triangle (marked blue) displays the HRs and 95% CI from direct comparison; the lower triangle (marked yellow) displays the HRs and 95% CI established from network meta-analysis.
For the upper triangle, HRs < 1 favor the row-defining treatment. For the lower triangle, HRs < 1 favor the column-defining treatment
The heterogeneity standard deviation (τ) was estimated at 0.03.

Supplemental table 31: Subgroup analysis for the fatty acids expanded network, including American cohort studies (5% isocaloric energy substitution);
Number of participants (n= 593 658) and number of mortality events (n= 182 925)

**PUFA**	0.88 [0.79, 0.98]	0.78 [0.70, 0.86]	0.62 [0.53, 0.72]	0.83 [0.75, 0.92]	NA
0.89 [0.81, 0.98]	**MUFA**	0.89 [0.80, 0.98]	0.70 [0.60, 0.81]	0.95 [0.86, 1.06]	NA
0.79 [0.72, 0.87]	0.89 [0.81, 0.98]	**SFA**	0.82 [0.71, 0.95]	1.09 [0.98, 1.21]	1.13 [0.94, 1.37]
0.64 [0.56, 0.74]	0.72 [0.63, 0.83]	0.81 [0.70, 0.93]	**TFA**	1.37 [1.18, 1.59]	NA
0.85 [0.78, 0.94]	0.96 [0.87, 1.05]	1.08 [0.98, 1.18]	1.33 [1.16, 1.53]	**CHO**	NA
0.90 [0.73, 1.11]	1.01 [0.82, 1.25]	1.13 [0.94, 1.37]	1.40 [1.11, 1.77]	1.05 [0.85, 1.30]	**PRO**

CHO carbohydrates; CI confidence interval; HR hazard ratio; MUFA monounsaturated fatty acids; NA not applicable; PRO protein; PUFA polyunsaturated fatty acids; SFA saturated fatty acids; TFA trans-fatty acids;
The HRs are established by the replacement of the lower nutrient with the one above, eg, the direct and network HR for ↑ PUFA (increase) vs. ↓ MUFA (decrease) are displayed in the first row second column and second row first column, respectively.
The upper triangle (marked blue) displays the HRs and 95% CI from direct comparison; the lower triangle (marked yellow) displays the HRs and 95% CI established from network meta-analysis.
For the upper triangle, HRs < 1 favor the row-defining treatment. For the lower triangle, HRs < 1 favor the column-defining treatment
The heterogeneity standard deviation (τ) was estimated at 0.10.

Supplemental table 32: Subgroup analysis for the protein-origin subnetwork, including American cohort studies (5% isocaloric energy substitution);
Number of participants (n= 684 084) and number of mortality events (n= 191 082)

**PP**	0.84 [0.81, 0.88]	0.83 [0.78, 0.88]	0.86 [0.83, 0.90]
0.87 [0.84, 0.90]	**AP**	0.94 [0.89, 0.99]	0.99 [0.96, 1.02]
0.82 [0.79, 0.86]	0.95 [0.91, 0.99]	**SFA**	NA
0.86 [0.83, 0.89]	0.99 [0.97, 1.02]	1.05 [1.00, 1.10]	**CHO**

AP animal protein; CHO carbohydrates; CI confidence interval; HR hazard ratio; NA not applicable; PP plant protein; SFA saturated fatty acids;
The HRs are established by the replacement of the lower nutrient with the one above, eg, the direct and network HR for ↑ PP (increase) vs. ↓ AP (decrease) are displayed in the first row second column and second row first column, respectively.
The upper triangle (marked blue) displays the HRs and 95% CI from direct comparison; the lower triangle (marked yellow) displays the HRs and 95% CI established from network meta-analysis.
For the upper triangle, HRs < 1 favor the row-defining treatment. For the lower triangle, HRs < 1 favor the column-defining treatment
The heterogeneity standard deviation (τ) was estimated at 0.03.

Supplemental table 33: Subgroup analysis for the overall macronutrient network, including studies with a single dietary assessment (5% isocaloric energy substitution);
Number of participants (n= 1 174 221) and number of mortality events (n= 185 772)

**FAT**	0.99 [0.96, 1.01]	0.97 [0.94, 1.01]
0.98 [0.96, 1.01]	**CHO**	1.00 [0.97, 1.02]
0.98 [0.95, 1.01]	1.00 [0.97, 1.02]	**PRO**

CHO carbohydrates; CI confidence interval; FAT fat; HR hazard ratio; PRO protein
The HRs are established by the replacement of the lower nutrient with the one above, eg, the direct and network HR for ↑ FAT (increase) vs. ↓ CHO (decrease) are displayed in the first row second column (HR: 0.97, 95% CI: 0.95, 0.99) and second row first column, respectively.
The upper triangle (marked blue) displays the HRs and 95% CI from direct comparison; the lower triangle (marked yellow) displays the HRs and 95% CI established from network meta-analysis.
For the upper triangle, HRs < 1 favor the row-defining treatment. For the lower triangle, HRs < 1 favor the column-defining treatment
The heterogeneity standard deviation (τ) was estimated at 0.03.

Supplemental table 34: Subgroup analysis for the protein-origin subnetwork, including studies with a single dietary assessment (5% isocaloric energy substitution);
Number of participants (n= 675 408) and number of mortality events (n= 175 283)

**PP**	0.87 [0.82, 0.92]	0.96 [0.81, 1.14]	1.16 [0.96, 1.41]	0.89 [0.82, 0.97]	0.88 [0.83, 0.93]
0.86 [0.82, 0.91]	**AP**	0.99 [0.88, 1.12]	1.20 [1.03, 1.40]	1.01 [0.94, 1.09]	1.03 [0.98, 1.07]
0.81 [0.72, 0.90]	0.94 [0.84, 1.04]	**PUFA**	1.21 [1.04, 1.41]	1.17 [1.03, 1.34]	1.12 [1.01, 1.25]
0.98 [0.85, 1.12]	1.13 [0.99, 1.30]	1.21 [1.04, 1.41]	**MUFA**	0.97 [0.82, 1.14]	0.93 [0.80, 1.07]
0.86 [0.80, 0.91]	0.99 [0.94, 1.06]	1.06 [0.95, 1.19]	0.88 [0.76, 1.01]	**SFA**	0.96 [0.85, 1.08]
0.88 [0.83, 0.93]	1.02 [0.98, 1.06]	1.09 [0.98, 1.21]	0.90 [0.78, 1.03]	1.02 [0.96, 1.09]	**CHO**

AP animal protein; CHO carbohydrates; CI confidence interval; HR hazard ratio; MUFA monounsaturated fatty acids; PP plant protein; PUFA polyunsaturated fatty acids; SFA saturated fatty acids; TFA trans-fatty acids;
The HRs are established by the replacement of the lower nutrient with the one above, eg, the direct and network HR for ↑ PP (increase) vs. ↓ AP (decrease) are displayed in the first row second column and second row first column, respectively.
The upper triangle (marked blue) displays the HRs and 95% CI from direct comparison; the lower triangle (marked yellow) displays the HRs and 95% CI established from network meta-analysis.
For the upper triangle, HRs < 1 favor the row-defining treatment. For the lower triangle, HRs < 1 favor the column-defining treatment
The heterogeneity standard deviation (τ) was estimated at 0.05.

Supplemental table 35: Sensitivity analysis for the overall macronutrient network, excluding studies with a high risk of bias (5% isocaloric energy substitution);
Number of participants (n= 1 161 535) and number of mortality events (n= 209 817)

**FAT**	0.97 [0.94, 1.00]	0.96 [0.91, 1.02]
0.97 [0.94, 0.99]	**CHO**	1.00 [0.97, 1.02]
0.97 [0.94, 1.00]	1.00 [0.98, 1.03]	**PRO**

CHO carbohydrates; CI confidence interval; FAT fat; HR hazard ratio; PRO protein
The HRs are established by the replacement of the lower nutrient with the one above, eg, the direct and network HR for ↑ FAT (increase) vs. ↓ CHO (decrease) are displayed in the first row second column (HR: 0.97, 95% CI: 0.95, 0.99) and second row first column, respectively.
The upper triangle (marked blue) displays the HRs and 95% CI from direct comparison; the lower triangle (marked yellow) displays the HRs and 95% CI established from network meta-analysis.
For the upper triangle, HRs < 1 favor the row-defining treatment. For the lower triangle, HRs < 1 favor the column-defining treatment
The heterogeneity standard deviation (τ) was estimated at 0.03.

Supplemental table 36: Sensitivity analysis for the fatty acids expanded network, excluding studies with a high risk of bias (5% isocaloric energy substitution);
Number of participants (n= 623 244) and number of mortality events (n= 187 630)

**PUFA**	0.84 [0.76, 0.92]	0.76 [0.69, 0.83]	0.62 [0.55, 0.71]	0.83 [0.76, 0.91]	NA
0.87 [0.79, 0.95]	**MUFA**	0.88 [0.80, 0.95]	0.73 [0.64, 0.83]	0.93 [0.86, 1.01]	NA
0.76 [0.69, 0.83]	0.88 [0.81, 0.95]	**SFA**	0.85 [0.74, 0.97]	1.10 [1.02, 1.20]	1.07 [0.90, 1.28]
0.65 [0.57, 0.74]	0.75 [0.66, 0.85]	0.85 [0.75, 0.96]	**TFA**	1.30 [1.14, 1.48]	NA
0.83 [0.76, 0.91]	0.95 [0.88, 1.03]	1.09 [1.00, 1.18]	1.28 [1.13, 1.44]	**CHO**	NA
0.82 [0.67, 1.00]	0.94 [0.77, 1.14]	1.07 [0.90, 1.28]	1.26 [1.01, 1.56]	0.99 [0.81, 1.20]	**PRO**

CHO carbohydrates; CI confidence interval; HR hazard ratio; MUFA monounsaturated fatty acids; NA not applicable; PRO protein; PUFA polyunsaturated fatty acids; SFA saturated fatty acids; TFA trans-fatty acids;
The HRs are established by the replacement of the lower nutrient with the one above, eg, the direct and network HR for ↑ PUFA (increase) vs. ↓ MUFA (decrease) are displayed in the first row second column and second row first column, respectively.
The upper triangle (marked blue) displays the HRs and 95% CI from direct comparison; the lower triangle (marked yellow) displays the HRs and 95% CI established from network meta-analysis.
For the upper triangle, HRs < 1 favor the row-defining treatment. For the lower triangle, HRs < 1 favor the column-defining treatment
The heterogeneity standard deviation (τ) was estimated at 0.09.

Supplemental table 37: Sensitivity analysis for the protein-origin subnetwork, excluding studies with a high risk of bias (5% isocaloric energy substitution);
Number of participants (n= 782 644) and number of mortality events (n= 207 369)

**PP**	0.85 [0.81, 0.89]	0.83 [0.78, 0.89]	0.84 [0.81, 0.88]
0.85 [0.82, 0.89]	**AP**	0.94 [0.89, 1.00]	1.00 [0.98, 1.03]
0.82 [0.78, 0.86]	0.96 [0.91, 1.01]	**SFA**	NA
0.85 [0.82, 0.89]	1.00 [0.97, 1.03]	1.04 [0.99, 1.10]	**CHO**

AP animal protein; CHO carbohydrates; CI confidence interval; HR hazard ratio; NA not applicable; PP plant protein; SFA saturated fatty acids;
The HRs are established by the replacement of the lower nutrient with the one above, eg, the direct and network HR for ↑ PP (increase) vs. ↓ AP (decrease) are displayed in the first row second column and second row first column, respectively.
The upper triangle (marked blue) displays the HRs and 95% CI from direct comparison; the lower triangle (marked yellow) displays the HRs and 95% CI established from network meta-analysis.
For the upper triangle, HRs < 1 favor the row-defining treatment. For the lower triangle, HRs < 1 favor the column-defining treatment
The heterogeneity standard deviation (τ) was estimated at 0.03.

Supplemental table 38: Sensitivity analysis for the overall macronutrient network, excluding studies with high relative residual effects (5% isocaloric energy substitution);
Number of participants (n= 1 520 063) and number of mortality events (n= 213 593)

**FAT**	0.97 [0.95, 0.99]	0.99 [0.96, 1.02]
0.97 [0.95, 0.99]	**CHO**	1.01 [0.99, 1.03]
0.98 [0.96, 1.00]	1.01 [0.99, 1.03]	**PRO**

CHO carbohydrates; CI confidence interval; FAT fat; HR hazard ratio; PRO protein
The HRs are established by the replacement of the lower nutrient with the one above, eg, the direct and network HR for ↑ FAT (increase) vs. ↓ CHO (decrease) are displayed in the first row second column (HR: 0.97, 95% CI: 0.95, 0.99) and second row first column, respectively.
The upper triangle (marked blue) displays the HRs and 95% CI from direct comparison; the lower triangle (marked yellow) displays the HRs and 95% CI established from network meta-analysis.
For the upper triangle, HRs < 1 favor the row-defining treatment. For the lower triangle, HRs < 1 favor the column-defining treatment
The heterogeneity standard deviation (τ) was estimated at 0.03.

Supplemental table 39: Sensitivity analysis for the fatty acids expanded network, excluding studies with high relative residual effects (5% isocaloric energy substitution);
Number of participants (n= 1 027 288) and number of mortality events (n= 242 504)

**PUFA**	0.91 [0.84, 0.99]	0.87 [0.80, 0.94]	0.54 [0.45, 0.65]	0.89 [0.83, 0.97]	0.99 [0.85, 1.16]
0.94 [0.87, 1.01]	**MUFA**	0.92 [0.85, 0.99]	0.70 [0.58, 0.84]	0.94 [0.87, 1.01]	0.94 [0.81, 1.10]
0.86 [0.80, 0.93]	0.92 [0.86, 0.98]	**SFA**	0.79 [0.66, 0.95]	1.05 [0.98, 1.13]	1.01 [0.90, 1.14]
0.74 [0.64, 0.86]	0.79 [0.68, 0.91]	0.86 [0.74, 0.99]	**TFA**	1.15 [0.99, 1.33]	1.04 [0.59, 1.84]
0.89 [0.83, 0.96]	0.95 [0.88, 1.02]	1.03 [0.97, 1.10]	1.21 [1.05, 1.39]	**CHO**	0.99 [0.85, 1.14]
0.91 [0.81, 1.02]	0.97 [0.86, 1.08]	1.05 [0.95, 1.17]	1.23 [1.04, 1.46]	1.02 [0.91, 1.14]	**PRO**

CHO carbohydrates; CI confidence interval; HR hazard ratio; MUFA monounsaturated fatty acids; PRO protein; PUFA polyunsaturated fatty acids; SFA saturated fatty acids; TFA trans-fatty acids;
The HRs are established by the replacement of the lower nutrient with the one above, eg, the direct and network HR for ↑ PUFA (increase) vs. ↓ MUFA (decrease) are displayed in the first row second column and second row first column, respectively.
The upper triangle (marked blue) displays the HRs and 95% CI from direct comparison; the lower triangle (marked yellow) displays the HRs and 95% CI established from network meta-analysis.
For the upper triangle, HRs < 1 favor the row-defining treatment. For the lower triangle, HRs < 1 favor the column-defining treatment
The heterogeneity standard deviation (τ) was estimated at 0.10.

Supplemental table 40: Sensitivity analysis for the protein origin network, excluding studies with high relative residual effects (5% isocaloric energy substitution);
Number of participants (n= 1 021 954) and number of mortality events (n= 224 522)

**PP**	0.87 [0.83, 0.91]	0.98 [0.88, 1.10]	1.02 [0.91, 1.15]	0.90 [0.84, 0.97]	0.93 [0.53, 1.64]	0.88 [0.85, 0.92]
0.87 [0.84, 0.91]	**AP**	1.01 [0.92, 1.10]	1.04 [0.95, 1.14]	0.99 [0.93, 1.05]	0.95 [0.55, 1.66]	1.01 [0.97, 1.04]
0.86 [0.80, 0.94]	0.99 [0.92, 1.07]	**PUFA**	1.05 [0.95, 1.17]	1.04 [0.95, 1.14]	0.93 [0.53, 1.63]	1.02 [0.94, 1.11]
0.91 [0.84, 1.00]	1.05 [0.97, 1.14]	1.06 [0.96, 1.17]	**MUFA**	0.98 [0.89, 1.08]	0.99 [0.56, 1.73]	0.95 [0.87, 1.04]
0.86 [0.82, 0.91]	0.99 [0.94, 1.04]	1.00 [0.92, 1.09]	0.94 [0.86, 1.03]	**SFA**	1.00 [0.57, 1.74]	0.97 [0.89, 1.05]
0.87 [0.50, 1.51]	1.00 [0.57, 1.73]	1.01 [0.58, 1.76]	0.95 [0.54, 1.66]	1.01 [0.58, 1.75]	**TFA**	0.97 [0.56, 1.69]
0.88 [0.84, 0.91]	1.01 [0.98, 1.04]	1.02 [0.94, 1.10]	0.96 [0.88, 1.04]	1.02 [0.96, 1.07]	1.01 [0.58, 1.76]	**CHO**

AP animal protein; CHO carbohydrates; CI confidence interval; HR hazard ratio; MUFA monounsaturated fatty acids; PP plant protein; PUFA polyunsaturated fatty acids; SFA saturated fatty acids; TFA trans-fatty acids;
The HRs are established by the replacement of the lower nutrient with the one above, eg, the direct and network HR for ↑ PP (increase) vs. ↓ AP (decrease) are displayed in the first row second column and second row first column, respectively.
The upper triangle (marked blue) displays the HRs and 95% CI from direct comparison; the lower triangle (marked yellow) displays the HRs and 95% CI established from network meta-analysis.
For the upper triangle, HRs < 1 favor the row-defining treatment. For the lower triangle, HRs < 1 favor the column-defining treatment
The heterogeneity standard deviation (τ) was estimated at 0.05.

Supplemental appendix 1: Search terms

Database(s): **Ovid MEDLINE(R) ALL** 1946 to February 09, 2024
Search Strategy:

#	Searches	Hits
1	exp dietary carbohydrates/	102,664
2	((carbohydrate* or glucose or fructose or sucrose or starch or glycemic index) adj9 (intake* or diet* or consum* or nutrition* or food* or eat or eating or meal*)).ti,ab,kf.	89,308
3	1 or 2	171,988
4	exp dietary proteins/	107,970
5	exp plant proteins, dietary/	7,496
6	exp animal proteins, dietary/	66,041
7	(protein* adj9 (intake* or diet* or consum* or nutrition* or food* or eat or eating or meal*)).ti,ab,kf.	96,324
8	or/4-7	176,382
9	exp dietary fats/	99,164
10	exp dietary fats, unsaturated/	45,862
11	(((saturated or trans or monounsaturated or polyunsaturated or animal or plant) AND (fat or fats)) adj9 (intake* or diet* or consum* or nutrition* or food* or eat or eating or meal*)).ti,ab,kf.	27,324
12	(((linoleic acid or omega-6 or omega-3) adj9 (intake* or diet* or consum* or nutrition* or food* or eat or eating or meal*))).ti,ab,kf.	9,674
13	or/9-12	119,354
14	(macronutrient* adj9 (intake* or diet* or consum* or nutrition* or food* or eat or eating or meal*)).ti,ab,kf.	7,956
15	3 or 8 or 13 or 14	418,987
16	(substitut* or replac* or exchang*).mp.	1,405,623
17	(expense or increment*).mp.	167,232
18	(nutrient density or nutrient densities).mp.	1,171
19	proportional hazards models/	90,867
20	(cox adj3 (model* or regression)).mp.	173,953
21	or/16-20	1,774,505
22	15 and 21	33,925
23	cohort studies/ or follow-up studies/ or longitudinal studies/ or prospective studies/	1,652,366
24	(prospective or cohort* or observational or longitudinal or follow-up or cases or (case* and control*) or population-based).ti,ab,kf.	4,942,129
25	23 or 24	5,428,059
26	**22 and 25**	**5,112**

Database(s): **Embase (via Website)** 1946 to February 09, 2024
Search Strategy:

#	Searches	Hits
1	‘carbohydrate intake’/exp	35,932
2	((carbohydrate* OR glucose OR fructose or sucrose OR starch OR ‘glycemic index’) NEAR/9 (intake* OR diet* or consum* OR nutrition* or food* OR eat OR eating OR meal*)):ti,ab,kw	119,275
3	#1 OR #2	137,317
4	‘protein intake’/de	50,203
5	(protein* NEAR/9 (intake* OR diet* OR consum* OR nutrition* OR food* OR eat OR eating OR meal*)):ti,ab,kw	117,504
6	#4 OR #5	137,649
7	‘fat intake’/exp	59,229
8	‘edible oil’/exp	85,686
9	((saturated OR trans OR monounsaturated OR polyunsaturated OR animal or plant) NEAR/3 (fat OR fats) NEAR/9 (intake* OR diet* or consum* OR nutrition* OR food* OR eat OR eating OR meal*)):ti,ab,kw	14,969
10	((‘linoleic acid’ OR “omega 6’ OR "omega 3’) NEAR/9 (intake* OR diet* OR consum* OR nutrition* OR food* OR eat OR eating OR meal*)):ti,ab,kw	10,368
11	#7 OR #8 OR #9 OR #10	153,735
12	(macronutrient* NEAR/9 (intake* OR diet* OR consum* OR nutrition* OR food* OR eat OR eating OR meal*)):ti,ab,kw	10,513
13	#3 OR #6 OR #11 OR #12	382,605
14	substitut* OR replac* or exchang*	1,775,385
15	expense OR increment*	223,634
16	‘nutrient density’ OR ‘nutrient densities’	1,367
17	‘proportional hazards models’/de	105,437
18	cox NEAR/3 (model* OR regression)	291,061
19	#14 OR #15 OR #16 OR #17 OR #18	2,280,544
20	#13 AND #19	30,523
21	‘cohort analysis’/de OR ‘follow up’/de OR ‘longitudinal study’/de OR ‘prospective study’/de	3,644,104
22	prospective:ti,ab,kw OR cohort*:ti,ab,kw OR observational:ti,ab,kw OR longitudinal:ti,ab,kw OR ‘follow up’:ti,ab,kw OR cases:ti,ab,kw OR (case*:ti,ab,kw AND control*:ti,ab,kw) OR ‘population-based’:ti,ab,kw	7,025,813
23	#21 OR #22	8,045,638
24	**#20 AND #23**	**6,296**

Database(s): **Scopus (via Website)** 1946 to February 13, 2024
Search Strategy:

#	Searches	Hits
1	TITLE-ABS-KEY((carbohydrate* OR glucose OR fructose or sucrose OR starch OR “glycemic index“) W/9 (intake* OR diet* or consum* OR nutrition* or food* OR eat OR eating OR meal*))	160,132
2	TITLE-ABS-KEY(protein* W/9 (intake* OR diet* OR consum* OR nutrition* OR food* OR eat OR eating OR meal*))	195,512
3	TITLE-ABS-KEY((saturated OR trans OR monounsaturated OR polyunsaturated OR animal or plant) W/3 (fat OR fats) W/9 (intake* OR diet* or consum* OR nutrition* OR food* OR eat OR eating OR meal*))	19,531
4	TITLE-ABS-KEY((“linoleic acid” OR “omega 6” OR “omega 3”) NEAR/9 (intake* OR diet* OR consum* OR nutrition* OR food* OR eat OR eating OR meal*))	1,728
5	#1 OR #2 OR #3 OR #4	336,755
6	TITLE-ABS-KEY(substitut* OR replac* or exchang*)	3,963,135
7	TITLE-ABS-KEY(expense OR increment*)	565,157
8	TITLE-ABS-KEY(“nutrient density” OR “nutrient densities”)	1,859
9	TITLE-ABS-KEY(cox W/3 (model* OR regression))	190,267
10	#6 OR #7 OR #8 OR #9	4,674,072
11	TITLE-ABS-KEY(prospective OR cohort* OR observational OR longitudinal OR “follow up” OR cases OR (case* AND control*) OR population-based)	14,640,491
12	#5 AND #10 AND #11	**5,517**
	((TITLE-ABS-KEY((carbohydrate OR glucose OR fructose OR sucrose OR starch OR "glycemic index") W/9 (intake* OR diet* OR consum* OR nutrition* OR food* OR eat OR eating OR meal*))) OR (TITLE-ABS-KEY((protein* W/9 (intake* OR diet* OR consum* OR nutrition* OR food* OR eat OR eating OR meal*)))) OR (TITLE-ABS-KEY(((saturated OR trans OR monounsaturated OR polyunsaturated OR animal or plant) W/3 (fat OR fats) W/9 (intake* OR diet* or consum* OR nutrition* OR food* OR eat OR eating OR meal*)))) OR (TITLE-ABS-KEY(("linoleic acid" OR "omega 6" OR "omega 3") NEAR/9 (intake* OR diet* OR consum* OR nutrition* OR food* OR eat OR eating OR meal*))) AND ((TITLE-ABS-KEY(substitut* OR replac* or exchang*)) OR (TITLE-ABS-KEY(expense or increment*)) OR (TITLE-ABS-KEY("nutrient density" OR "nutrient densities")) OR (TITLE-ABS-KEY(cox W/3 (model* OR regression)))) AND (TITLE-ABS-KEY((prospective OR cohort* OR observational OR longitudinal OR "follow up" OR cases OR (case* AND control*) OR population-based)))*	

Supplemental appendix 2: Detailed description and decision criteria for each domain in ROBINS-E assessment

<table>
<tr><th>Domain</th><th>Explanation</th><th>Judgements</th></tr>
<tr><td>Risk of bias due to confounding</td><td>Is there potential for confounding of the effect of exposure in this study?
Did the authors use a multivariable-adjusted analysis method that controlled at least for age, sex, education/SES, smoking, alcohol consumption, physical activity?
Were confounding factors that were controlled for measured validly and reliably by the variables available in this study?
Did the authors avoid adjusting for post-exposure variables?
Notes: Confounding is expected in all observational studies; thus, no study was assigned low risk of bias. Time-varying confounding was expected to be unlikely and is not expected to cause risk of bias in the present study.</td><td>Low risk of bias:
No bias is expected due to confounding, including time-varying confounding.

Some concerns:
Confounding is expected for age, sex, smoking, alcohol consumption, education/SES, physical activity, and the authors performed a multivariable-adjusted analysis to control for these confounding factors. The variables adjusted for are valid and reliable measures of the confounding factors.
or
SES is the only important covariate not included as confounding factor in the multivariable-adjusted analysis, but SES is not expected to vary substantially within the cohort (eg, NHS, HPFS).
or
The authors statistically investigated whether the confounding factors have an effect on the risk estimate and excluded the confounder from the multivariable model if there was no effect on the overall effect estimate.

High risk of bias:[a]
At least one known important confounding factor was not measured or appropriately controlled for.
or
The authors adjusted for post-exposure variables that are affected by exposure (eg, sodium intake and risk of stroke [adjustment for blood pressure during follow-up = intermediate biological variable on the causal pathway] → over adjustment).

Very high risk of bias:[a]
No adjustment was made for any covariate.
or
The authors controlled for post-exposure variables, and the use of negative controls, or other considerations, suggest serious uncontrolled confounding.</td></tr>
</table>

**Risk of bias arising from measurement of exposure assessment**	• Does the measured exposure well-characterize the exposure metric specified to be of interest in this study?<br>• Was the exposure likely to be measured with error, or misclassified?<br><br>*Notes:* Differential misclassification is not expected to occur in prospective cohort studies, since diet is reported before the occurrence of the outcome (39).<br>Some type of non-differential misclassification cannot be excluded (any dietary assessment method involves measurement error), thus no study was assigned low risk of bias.	Low risk of bias:<br>The exposure status is well characterised by the measurement and no measurement error is expected in its assessment.<br>*and*<br>The exposure was measured at multiple times, and is stable or changes only slightly over time.<br><br>Some concerns:<br>The exposure status is well characterised by the measurement, and was measured using an established or validated tool (eg, a validated FFQ/DHQ, **multiple** 24h recalls).<br>*and* (1) *or* (2)<br><br>(1) The exposure was measured at multiple times, and it is stable or changes only slightly over time.<br>(2) The exposure was measured by a single measurement assessing longer periods of time (ie, validated FFQ/DHQ), and is therefore assumed to be stable over time.<br><br>High risk of bias:<br>The exposure status is not well characterized by the measurement (eg, assumed from an indirect measurement or important sources of dietary intake are not considered).<br>*or*<br>The exposure was measured using a not validated tool.<br>*or*<br>The exposure was measured with a single measurement, which is unlikely to characterize the exposure over a longer period of time (eg, single 24h recall) and therefore cannot be assumed to be representative.<br>*or*<br>The exposure cannot be assumed to be stable over time.<br><br>Very high risk of bias:<br>Differential measurement error is expected (measurement error depends on the outcome).

**Risk of bias in selection of participants into the study**	• Was selection of participants into the study (or into the analysis) based on participant characteristics observed after the start of the exposure window being studied?<br>• Do start of follow-up and start of exposure coincide for most participants?<br>• Were methods used that are likely to correct for the presence of selection biases?<br><br>Notes: In observational studies, it is unlikely that post-exposure variables influenced selection of participants into the study. Exclusion of participants may be mostly based on missing data, which will be considered in the domain referring to missings (see below).	Low risk of bias:<br>All participants who would have been eligible for the target study were included in the study.<br>*and*<br>The start of exposure and follow-up coincide.<br><br>Some concerns:<br>The selection into the study may have been related to exposure and outcome but, the authors used appropriate methods to correct for the selection bias.<br>*or*<br>The start of exposure and follow-up do not coincide, but the association of exposure is constant over time.<br><br>High risk of bias:<br>The selection into the study was related to exposure and outcome and this could not be corrected for in the analyses.<br>*or*<br>The start of exposure and follow-up do not coincide and the effect of exposure is not constant over time and this could not be corrected for in the analyses.<br><br>Very high risk of bias:<br>The selection into the study was related to exposure and outcome and a sensitivity analysis is available that demonstrates substantial impact.<br>*or*<br>The start of exposure and follow-up do not coincide and the effect of exposure is not constant over time and a sensitivity analysis is available that demonstrates substantial impact.

**Risk of bias due to post-exposure interventions**	• Were there post-exposure interventions that were influenced by prior exposure during the follow-up period?<br><br>*Notes:* In prospective observational studies, post-exposure interventions are unlikely. We don't expect any issues in this domain for our analysis.	Low risk of bias:<br>There were (probably) no interventions administered to alleviate the effect of exposures.<br><br>Some concerns:<br>Post-exposure interventions were identified and the analysis corrected for the effect of these interventions.<br><br>High risk of bias:<br>Post-exposure interventions were identified and the analysis did not correct for the effect of these interventions
**Bias due to missing data**	• Were there missing outcome data?<br>• Were participants excluded due to missing data on exposure status?<br>• Were participants excluded due to missing data on other variables needed for analysis?<br>• Did the authors perform a complete case analysis?<br>• Was an appropriate method used to correct for bias due to missing data (eg, appropriate imputation)?<br><br>*Notes:* Missing data on exposure variables and other variables are expected to be missing at random and not related to exposure or outcome that have been assessed during follow-up.	Low risk of bias:<br>There was little loss-to-follow-up (<20%) and data on exposure and other variables were reasonably complete (<10% missing data) and was unlikely to introduce bias.<br>*or*<br>The analysis addressed missing data and is likely to have removed any risk of bias.<br><br>Some concerns:<br>There is a proportion (>10%) of missing data in the original cohort or a high proportion (>20%) of loss-to-follow-up, and the analysis is unlikely to have removed the risk of bias arising from the missing data (eg, using logistic regression).<br>*or*<br>There is a significant proportion (>20%) of missing data but the authors addressed this issue by appropriate methods (ie, imputation of data).<br><br>High risk of bias:<br>There are high proportions (>50%) of missing data and the analysis is unlikely to have removed the risk of bias arising from the missing data.<br>*or*<br>The nature of the missing data means that the risk of bias cannot be removed through appropriate analysis.<br><br>Very high risk of bias:<br>There are very high proportions (>50%) of missing data; and missing data were addressed inappropriately in the analysis.

**Risk of bias due to measurement of the outcome**	• Were the methods of outcome assessment comparable across exposure groups?<br>• Could the outcome measure have been influenced by knowledge of the exposure status?<br>• Were any systematic error in measurement of the outcome related to exposure status?<br><br>*Notes:* In prospective observational studies, it is not expected that outcome assessors were aware of exposure status of the participants.	Low risk of bias:<br>The methods of outcome assessment were comparable across all exposure groups.<br>*and*<br>The outcome measure was unlikely to be influenced by knowledge of the exposure status of study participants.<br>*and*<br>Any error in measuring the outcome is unrelated to exposure status (ie, objective measures or self-reported outcomes that are mostly (≥90%) confirmed by a second source, eg, medical records, record linkage and death certificates).<br><br>Some concerns:<br>The methods of the outcome assessment were comparable across exposure groups, and any error in measuring the outcome may be minimally related to exposure status.<br>*or*<br>The measurement of the outcome is not reliable (ie, confirmed records are available for <90% of all participants and the authors did not perform an additional analysis separating confirmed and probable cases).<br><br>High risk of bias:<br>The methods of outcome assessment were not comparable across exposure groups, and errors in measuring the outcome were related to exposure status.<br>*or*<br>The outcome measure was subjective (ie, self-report of CVD, type 2 diabetes, etc by study participants or next of kin, without confirmation by a second source), and errors in measuring the outcome were related to exposure status.

<table>
<tr><td>Risk of bias due to selection of the reported result</td><td>Was the result reported in accordance with an available, pre-determined analysis plan?
Is the reported effect estimate likely to be selected from multiple exposure measurements?
Is the reported effect estimate likely to be selected from multiple analyses of exposure-outcome relationship?
Is the reported effect estimate likely to be selected from different subgroups?
Notes: In observational studies, it is unusual to publish an a priori analysis plan or protocol. Therefore, if the authors present a clear description of the conducted analyses (ie, methods section), and it appears to be consistent with the reported results; and the reported results correspond to all intended outcomes, analyses and sub cohorts (eg, postmenopausal women), low risk of bias can be adequate.
However, if there are any inconsistencies/or no information between intended analyses and reported results, eg,:
a substitution analysis was conducted in line with the methods section, but reported results were incomplete (eg, authors state that a substitution analysis for fat vs. carbohydrates was conducted, but estimates are not reported);
results of substitution analyses reported, but methodological approach not described;
there are inconsistencies between the adjustments described in the methods section and the adjustments for the corresponding reported estimates in the results section;
There is reason for some concerns in this domain.
Multiple outcome measurements for the definition of CVD, mortality, type 2 diabetes, etc are not expected.</td><td><u>Low risk of bias:</u>
The results are reported according to an a-priori analysis plan or protocol. There is a clear description of all analysis, the analyses are consistent, and all reported results correspond to all intended outcomes, analyses and sub-cohorts.

<u>Some concerns:</u>
The results are reported according to an a-priori analysis plan or protocol, and there is indication of selection of the reported analysis among multiple analyses; or there is indication of selection of the cohort or subgroups for analysis and reporting on basis of the results (eg, estimates not shown for all analyses).
or
There is no a-priori analysis plan or protocol and there appear to be no issues with the exposure, multiple analyses (eg, effect estimates were similar when different multiple analyses were used), or the selection or definition of subgroups, but there are inconsistencies/or no information between intended and reported analyses.

<u>High risk of bias:</u>
There is a high risk of selective reporting from multiple exposure measurements, outcomes measurements, or multiple analyses of data.
or
The cohort or subgroup is selected from a larger study for analysis and appears to be reported based on the results. (up to 2)

<u>Very high risk of bias:</u>
There is a high risk of selective reporting from multiple exposure measurements, or outcomes measurements, or multiple analyses of data or the cohort or subgroup is selected from a larger study for analysis and appears to be reported based on the results. (more than 2)</td></tr>
<tr><td rowspan="3">Overall judgement</td><td>Low risk of bias</td><td>The study is judged to be at low risk of bias for all domains.</td></tr>
<tr><td>Some concerns</td><td>The study is judged to be at low risk of bias or some concerns for all domains.</td></tr>
<tr><td>High risk of bias</td><td>The study is judged to be at high risk of bias in at least one domain, but no domains are at very high risk of bias.</td></tr>
</table>

	Very high risk of bias	The study is judged to be at very high risk of bias in at least one domain.

CVD cardiovascular disease; DHQ diet history questionnaire; FFQ food frequency questionnaire; HPFS Health Professional Follow up Study; NHS Nurses' Health Study; SES socioeconomic status

[a]Triage: A (very) high risk of bias in the first domain leads to a triage. The further domains will not be considered for evaluation, as the overall judgement will already be at high risk of bias. (40)

Supplemental appendix 3: Handling of substitution analyses reported per kcal/d or g/d

If a study expressed the isocaloric replacement of dietary (macro)nutrients as kcal/d or g/d we calculated the 5% exchange accordingly. The substitution for g/d was transformed into kcal/d by multiplying with specific caloric values for each nutrient (for carbohydrates $f_c = 4kcal$, fat $f_f = 9kcal$, and protein $f_p = 4kcal$), which was then divided by the amount of total energy (mean, kcal/d) in order to establish the value as percentage. Afterwards the percent exchange was calculated to 5%.

For example for Das et al (6) we multiplied the mean of each quintile by the factor 4 (exchange of carbohydrates with protein), and divided each value by 2103 kcal/d, which was the total amount of energy consumed per day. We proceeded by estimating the linear estimate using Greenland and Longnecker method (38).

Quintile	Protein intake (g/d)	Protein intake (kcal/d)	Protein intake (% of total energy)
Quintile 1	72.0	288.2	13.7
Quintile 2	86.4	345.6	16.4
Quintile 3	100.4	401.6	19.1
Quintile 4	115.7	462.8	22.0
Quintile 5	132.7	530.7	25.2

g/d grams/day; kcal/d kilocalories/day
Quintiles of protein intake reported by Das et al. (6). Intakes were calculated as kcal/d and % of total energy intake by multiplying by 4 and dividing by 2103kcal/d, which was the mean of total amount of energy consumed per day.

Supplemental appendix 4: Handling of multiple publications on the same cohort:

If more than one publication reported different treatment comparisons from the same cohort and the comparison did not share any common nodes, we treated them as separate cohort studies. If more than one publication reported different treatment comparisons from the same cohort and the comparison did share a common node, we treated them as a single cohort study for the analyses, but we adjusted the respective weight accordingly. If a study reported a direct comparison, while within another the indirect[a] comparison was available, we included the reported comparison. In order to do so, we did not use the calculation of the indirect[a] comparison and inflated the variance of the arm by the factor $\frac{number\ of\ comparisons}{number\ of\ arms-1}$. This arises as consequence of the factor being $f = \frac{k}{2}$, where k is the number of arms, when a study reports all relevant comparisons. Since there are $n = \frac{k(k-1)}{2}$ comparisons in a fully connected study, this generalises to $f = \frac{n}{k-1}$. In a second approach both studies are merged to a multi-arm study, by applying the factor $f = \frac{number\ of\ joined\ arms}{number\ of\ reported\ arms}$. For example, if a 3-arm study is merged with a 7-arm study we adjust its variances by $\frac{7}{3}$. Since multi-arm studies are adjusted by the netmeta package by a factor $= \frac{k}{2}$, we override this by dividing $\frac{k}{2}$ and afterwards applying the correct factor $f = \frac{k\prime}{k}$, where k' is the number of the merged multi-arm study. The difference of these approaches is, the calculation of the indirect comparisons is done by the random effects model applied in the final NMA, while in our approach a fixed effect model is used in order to merge two cohort studies of the same cohort to a larger multi-arm study.

[a] Note that the term "indirect" in this case, is not the same as the computation of indirect estimates for the NMA. Here, "indirect" refers to a comparison that was available **within** a study. For example, Das et al. (6) reported estimates for the substitution of PRO vs CHO and PRO vs FAT and based on this, the "not reported" effect estimate for the substitution of FAT vs CHO was estimated. The analysis is specified in supplemental appendix 5.

Supplemental appendix 5: Handling of missing or inconsistent risk estimates and variances:

Estimates:

Due to secondary nature of substitution analyses in nutritional epidemiological publications, not every estimate was always provided for all possible comparisons. However, “not reported” estimates are estimated based on the “reported” relative effects within a study. Ideally, a multi-arm study reports consistent effect estimates, where k-1 comparisons (k = number of arms) are needed to derive all other possible effect estimates for a given network. These comparisons form a “spanning tree”. In this case excess effects can be dismissed in order to build the design matrix. We proceeded by calculating arm-based study specific effects. For every study, nuisance parameter ie, taking an arbitrary arm as 0, were used. Note that these arm effects are later converted back to contrast-based effects for inclusion into the NMA and thus their values are not relevant to the analysis and are not reported or interpreted. Afterwards the arm-based study specific were merged if required and all relative effects were than included into netmeta for the final NMA.

Whenever more than k-1 effects were reported by a study we assessed the consistency by comparing the reported effects with those calculated by the spanning tree. In order to do so, the spanning tree with the maximum precision was selected and residuals between reported and calculated effect were compared. If the residual of the observed effect was higher than 30% and the associated Q value ($\Sigma residual_i^n weights_i^n$), with $weights_i^n$ being the inverse of the variances of residuals, resulted in a p-value < 0.90 we recorded the cohort study as study with “high residual effect”.

Variances:

Every arm specific effect requires a corresponding variance (ie, there are k arms and k variances).

If a study reports k or more variances for the comparisons and they are consistent, we select the variances corresponding to the k-1 arms identified by the spanning tree and add the additional relative variance, which forms a triangle with the spanning tree. After which arm-based variances are established by the design matrix.

In case of inconsistency, the corresponding arm-based variance will be negative and must be dismissed. We proceed as if we only had k-1 variances reported and impute the missing value as the maximum variance between the variances of the other two comparisons consisting the respective triangle loop.

In case a study did not report higher-tier contrasts (eg, fat vs carbohydrates, fat vs protein), but did specify lower-tier origin-specific contrasts (eg, SFA vs carbohydrates, MUFA vs carbohydrates, etc), we approximated higher-tier contrast by pooling lower tier contrasts. This way we combined networks 2-7 or 3-7 with network 1 or 2 (original nodes → higher-tier node, ie, SFA, MUFA, PUFA, TFA → fat). We merged arm-based study-specific effects and variances as a fixed effect meta-analysis and we then converted effects to contrast based study effects which were used in the NMA.

Supplemental appendix 6: Detailed description and decision criteria for each domain in the GRADE assessment

Estimate	Domain	Judgement / explanation
In order to establish the certainty of evidence for each comparison every direct, indirect and network estimate for all comparisons in a network must be evaluated. The guidance is adapted according to Brignardello-Petersen et al. and Izcovich et al. (41, 42).<br><br>Due to the Risk of Bias evaluation with the ROBINS-E tool the GRADEing starts with a "high" certainty of evidence. Each downgrade leads to a decrease in certainty per one level. After downgrading by three levels, the certainty of the evidence is "very low" and it cannot be graded any lower (43).<br><br>Threshold choice: Minimally contextualized approach using the null effect (Hazard ratio = 1) as threshold (44) due to the importance of the outcome all-cause mortality on a population level.<br><br>The assessment of the certainty of evidence was conducted at network level and incorporated always the main nutrients of the network (ie, network 1: CHO vs. PRO). No comparison was graded twice (eg, CHO vs. PRO was graded for Network 1 only).		
**Direct estimate**	**Risk of bias**	<u>Don't downgrade:</u><br>More than 2/3 of the studies (and their contributing weight) are rated with low RoB.<br>*and*<br>No cohort study is rated with high RoB.<br><br><u>Downgrade by 1 level:</u><br>Less than 2/3 of the studies are rated with low RoB.<br>*and*<br>Less than 2/3 of the studies are rated with (very) high RoB, or more than 2/3 of the studies (and their contributing weight) are rated with a high RoB, **but** the subgroup analysis, excluding studies with a (very) high risk of bias, is robust.<br><br><u>Downgrade by 2 levels:</u><br>More than 2/3 of the studies (and their contributing weight) are rated with a high RoB.<br>*and*<br>The estimate of the subgroup analysis, excluding studies with a (very) high risk of bias, differs from the main analysis, or there is no subgroup analysis.<br><br><u>Downgrade by 3 levels:</u><br>More than 1/3 of the studies (and their contributing weight) are rated with a very high RoB.<br>*and*<br>The estimate of the subgroup analysis, excluding studies with a (very) high risk of bias, differs from the main analysis, or there is no subgroup analysis.

	**Inconsistency**	Don't downgrade:<br>The point estimates indicate a similar direction of effect and the corresponding 95% CI overlap to a high degree.<br>*or*<br>The point estimates show some heterogeneity, but this can be explained by differences between the studies.<br><br>Downgrade by 1 level<br>The point estimates differ distinctly between studies.<br>*and*<br>The corresponding 95% CI overlap only minimally or not at all.<br>*and*<br>The statistical test for heterogeneity shows a low p-value and the I^2 value is large.
	**Indirectness**	Don't downgrade:<br>The intervention/exposure of the included studies as well as the population studied represents the research question of interest and directly measures outcomes of interest.<br><br>Downgrade by 1 level:<br>The population of included studies differs markedly (in biology and/or physiology) from the population of interest and this could have a substantial impact on the magnitude effect.<br>*or*<br>The outcome measures are only available as surrogate parameters instead of the outcome intended.<br>*or*<br>The duration, intensity or modality of the intervention/exposure differs to an extend that the magnitude of effect could be influenced.
	**Publication bias**	Don't downgrade:<br>The funnel plot shows no asymmetry.<br><br>Downgrade by 1 level:<br>The funnel plot shows a substantial asymmetry.
	**Overall GRADEing of the direct estimate**	The certainty of evidence for the direct estimate can be rated as "high", "moderate", "low" or "very low".
**Indirect estimate [a]**	**Starting point**	The most dominant first order loop for the indirect estimate is formed by two direct estimates / has only one additional node. For example the indirect evidence of A vs C is established by A vs B and B vs C (2 arms, additional node = B).<br><br>If there are more than 1 first order loops the one with the higher number of studies and the lesser inverse variance is chosen.

		The lower rating of the two comparisons forming the most dominant first order loop is the starting point for the evaluation of the indirect estimate.
	**Intransitivity**	Don't downgrade:<br>Differences in the direct comparisons that form the indirect estimate are assumed to be differences relating only to the exposures of interest per each arm. There are probably no effect modifiers which lead to reasonable questioning of the credibility of the indirect estimate.<br><br>Downgrade by 1 level:<br>Effect modifiers vary substantially between the two arms that form the indirect estimate and there is a strong assumption that this has an impact on the credibility of the indirect estimate.
	**Overall GRADEing of the direct estimate**	The certainty of evidence for the indirect estimate can be rated as "high", "moderate", "low" or "very low", depending on the starting point.
**Network estimate**	**Starting point**	The higher rating of the direct and indirect estimate is the starting point for the assessment of the network estimate.
	**Incoherence**	Don't downgrade:<br>The direct and indirect estimates as well as their corresponding 95% CI are coherent. The p-value for the comparison of the indirect and direct evidence is not significant.<br><br>Downgrade by 1 level:<br>The direct and indirect estimates differ beyond chance and this difference cannot be explained. The p-value for the comparison of the indirect and direct evidence is significant.
	**Imprecision**	Don't downgrade:<br>The 95% CI doesn't cross the threshold (RR/HR of 1) and the ratio of the upper to the lower bound of the 95% CI is < 3.<br>*or*<br>The 95% CI crosses the threshold (RR/HR of 1) however, the 95% CI is narrow and as a result strongly indicates a null effect.<br><br>Downgrade by 1 level:<br>The 95% CI includes a RR/HR of 1 and the 95% CI is not narrow enough (maximal width of 0.05) to justify a null effect.<br><br>Downgrade by 2 levels:<br>The 95% CI includes a RR/HR of 1 and the ratio of the upper to the lower bound of the 95% CI is >3.<br>*or*<br>There is a large effect (HR/RR <0.5 or >2), the 95% CI doesn't cross the threshold of RR/HR = 1, but the ratio of the upper to the lower bound of the 95% CI is >3.

	**OPTION TO UPGRADE**	**Prerequisite for the option to upgrade:**<br>There was no downgrading for inconsistency and there was no downgrading for more than 1 levels for RoB. The direct and indirect estimates are coherent and there was no downgrading for imprecision.
	**Dose-response**	Option to upgrade by 1 level:<br>The prerequisites are met and the analyses show a consistent does-response relationship across and within all studies (>50% of studies must report a consistent dose response effect within the study).
	**Large effect**	Option to upgrade by 1 level:<br>The prerequisites are met and there is a large effect (HR/RR <0.5 or >2) with a narrow 95% CI.<br><br>Option to upgrade by 2 levels:<br>The prerequisites are met and there is a large effect (HR/RR <0.2 or >5) with a narrow 95% CI.
**Overall rating:**		The overall certainty of evidence for each comparison can be rated as “high”, “moderate”, “low” or “very low”.

95% CI 95% confidence interval; CHO carbohydrates; GRADE grading of recommendations, assessment, development, and evaluations; HR hazard ratio; PRO protein; RR risk ratio; RoB Risk of bias; ROBINS-E tool Risk Of Bias In Non-randomized Studies - of Exposures tool

[a] If the certainty of evidence of the direct estimate is “high” and the direct evidence contributes as much as the indirect evidence there is no need to grade the indirect estimate.

Supplemental appendix 7: List of excluded studies

Reason for exclusion	References
No exposure relevant substitution analysis (n = 675)	(45-294)(295-536)(537-719)
Wrong study design (n = 10)	(720-729)
**Reason for exclusion from the present review [a]**	**References**
Wrong outcome (all, n = 189):	(730-918)
• Cancer (n = 24)	(740, 752, 758, 762, 766, 776, 777, 797, 799, 804, 811, 817, 829, 844, 847, 854, 855, 861, 873, 878, 889, 890, 892, 918)
• Cardiovascular disease (n = 51)	(749, 750, 755, 760, 763, 765, 772, 773, 775, 779, 781, 783, 784, 787-790, 796, 798, 801, 802, 807, 813, 827, 830-835, 839, 845, 846, 850, 857, 866, 869, 872, 876, 877, 882, 887, 888, 894, 897, 903, 904, 908, 909, 912, 913)
• Type 2 diabetes (n = 34)	(731, 741, 748, 759, 760, 769, 770, 803, 808-810, 815, 820-822, 836, 838, 841, 848, 849, 851-853, 865, 870, 884-886, 891, 895, 896, 900, 901, 911)
• Secondary Prevention (n = 20)	(743-745, 756, 761, 764, 778, 786, 792, 795, 823, 826, 837, 856, 859, 863, 864, 873, 879, 910)
• Age related outcomes (n = 23)	(737, 747, 754, 767, 771, 782, 785, 791, 793, 816, 824, 842, 858, 868, 880, 881, 892, 898, 899, 905-907, 917)
• Adiposity (n = 16)	(730, 734-736, 738, 746, 780, 800, 812, 814, 825, 828, 843, 867, 874, 914)
• Other diseases (n = 26)	(732, 733, 739, 742, 751, 753, 757, 768, 774, 794, 805, 806, 818, 819, 840, 843, 860, 862, 869, 871, 875, 883, 893, 902, 915, 916)

[a] References were excluded from the present review if no information on all-cause mortality was available. Some publications reported multiple outcomes.

Supplemental appendix 8: Analytical procedure for UK Biobank study

We were not able to use the published effect estimates from the substitution model by Ho et al. (14), since only the replacement of different nutrients as curves conditional on the current macronutrient intake were presented. Due to access to the raw UK Biobank data (Approved project 75001), we replicated the analysis as linear substitution model expanded for all relevant comparisons. We included participants with at least one complete 24h recall and plausible energy intake (males: >800 kcal/d and < 4200 kcal/d and females: >600 kcal/d and < 3500 kcal/d). An energy partition model was used to evaluate the substitution of different nutrients. In this model the difference between the coefficients for energy intake from substituted nutrients were calculated. The analysis was conducted at 5% energy substitution. Adjustments were conducted in concordance with the original paper by Ho et al. (14) (ie, age, sex, total energy intake, BMI, height, smoking status, daily alcohol and fiber intake, deprivation index, ethnicity, total physical activity, systolic blood pressure, baseline diabetes, and mental health disorders). Data from 208 294 participants with an average follow up of 13.2 years were analyzed (deaths from all-cause mortality was n= 12 611).

Supplemental appendix 9: Deviations from protocol

The systematic review protocol (including all pre-specific inclusion criteria) was registered in the International Prospective Register of Systematic Reviews (PROSPERO; registration number CRD42023450706).

Pre-specified	Conducted	Reason for deviation
The carbohydrate-origin network was planned to contain the nodes:<br>Glucose, fructose, sucrose, starch, fat, protein	The carbohydrate-origin network was analysed as follows:<br>High quality carbohydrates / Polysaccharides, low quality carbohydrates / Mono-/Disaccharides, SFA, MUFA, PUFA, TFA, protein	Due to the limited data availability we were not able to analyze the pre-specified network. However, given the importance of this network, instead we provided an alternative network by classifying carbohydrates as ”high quality” (including: complex, starch) to form the node “high quality carbohydrates / polysaccharides” and by classifying carbohydrates as “low quality” (including: sugar, mono-/disaccharides) to form the node “low quality carbohydrates / mono-/disaccharides”.

MUFA monounsaturated fatty acids; PUFA polyunsaturated fatty acids; SFA saturated fatty acids; TFA trans-fatty acids;

Supplemental References

Additional References

1. Reynolds AN, Hodson L, De Souza R, Tran Diep Pham H, Vlietstra L, Mann J. Saturated fat and trans-fat intakes and their replacement with other macronutrients: a systematic review and meta-analysis of prospective observational studies. Geneva: World Health Organisation; 2022.

38. Greenland S, Longnecker MP. Methods for trend estimation from summarized dose-response data, with applications to meta-analysis. Am J Epidemiol. 1992;135(11):1301-9.

39. Freedman LS, Schatzkin A, Midthune D, Kipnis V. Dealing with dietary measurement error in nutritional cohort studies. J Natl Cancer Inst. 2011;103(14):1086-92.

40. Sterne J, Reeves B, Savovic J, Higgins J. Assessing risk of bias in non-randomized studies of interventions: introduction to the ROBINS-S tool. ROBINS-I London Workshop October 2023. www.riskofbias.info: University of Bristol; 2023. p. https://drive.google.com/file/d/1FyRqs88FTp42b6fQsT-5CeDif07Mn776/view.

41. Brignardello-Petersen R, Bonner A, Alexander PE, Siemieniuk RA, Furukawa TA, Rochwerg B, et al. Advances in the GRADE approach to rate the certainty in estimates from a network meta-analysis. J Clin Epidemiol. 2018;93:36-44.

42. Izcovich A, Chu DK, Mustafa RA, Guyatt G, Brignardello-Petersen R. A guide and pragmatic considerations for applying GRADE to network meta-analysis. BMJ. 2023;381:e074495.

43. Schünemann HJ, Cuello C, Akl EA, Mustafa RA, Meerpohl JJ, Thayer K, et al. GRADE guidelines: 18. How ROBINS-I and other tools to assess risk of bias in nonrandomized studies should be used to rate the certainty of a body of evidence. J Clin Epidemiol. 2019;111:105-14.

Included Studies

2. Argos M, Melkonian S, Parvez F, Rakibuz-Zaman M, Ahmed A, Chen Y, Ahsan H. A population-based prospective study of energy-providing nutrients in relation to all-cause cancer mortality and cancers of digestive organs mortality. Int J Cancer. 2013;133(10):2422-8.

3. Bajracharya R, Katzke V, Mukama T, Kaaks R. Effect of Iso-Caloric Substitution of Animal Protein for Other Macro Nutrients on Risk of Overall, Cardiovascular and Cancer Mortality: Prospective Evaluation in EPIC-Heidelberg Cohort and Systematic Review. Nutrients. 2023;15(3).

4. Budhathoki S, Sawada N, Iwasaki M, Yamaji T, Goto A, Kotemori A, et al. Association of Animal and Plant Protein Intake With All-Cause and Cause-Specific Mortality in a Japanese Cohort. JAMA Intern Med. 2019;179(11):1509-18.

5. Chen Z, Glisic M, Song M, Aliahmad HA, Zhang X, Moumdjian AC, et al. Dietary protein intake and all-cause and cause-specific mortality: results from the Rotterdam Study and a meta-analysis of prospective cohort studies. Eur J Epidemiol. 2020;35(5):411-29.

6. Das A, Cumming R, Naganathan V, Blyth F, Couteur DGL, Handelsman DJ, et al. Associations between dietary intake of total protein and sources of protein (plant vs. animal)

and risk of all-cause and cause-specific mortality in older Australian men: The Concord Health and Ageing in Men Project. J Hum Nutr Diet. 2022;35(5):845-60.

7. Dehghan M, Mente A, Zhang X, Swaminathan S, Li W, Mohan V, et al. Associations of fats and carbohydrate intake with cardiovascular disease and mortality in 18 countries from five continents (PURE): a prospective cohort study. Lancet. 2017;390(10107):2050-62.

8. Dominguez LJ, Bes-Rastrollo M, Basterra-Gortari FJ, Gea A, Barbagallo M, Martínez-González MA. Should we recommend reductions in saturated fat intake or in red/processed meat consumption? The SUN prospective cohort study. Clin Nutr. 2018;37(4):1389-98.

9. Fontana L, Sieri S, Ricceri F, Agnoli C, Pala V, Masala G, et al. Dietary intake of animal and plant proteins and risk of all cause and cause-specific mortality: The Epic-Italy cohort. Nutrition and Healthy Aging. 2021;6:257-68.

10. Fridén M, Olsson E, Lind L, Rosqvist F, Risérus U. Substitution analyses of foods with varying fat quality and the associations with all-cause mortality and impact of the FADS-1 genotype in elderly men. Eur J Nutr. 2023.

11. Guasch-Ferré M, Babio N, Martínez-González MA, Corella D, Ros E, Martín-Peláez S, et al. Dietary fat intake and risk of cardiovascular disease and all-cause mortality in a population at high risk of cardiovascular disease. Am J Clin Nutr. 2015;102(6):1563-73.

12. Guasch-Ferré M, Zong G, Willett WC, Zock PL, Wanders AJ, Hu FB, Sun Q. Associations of Monounsaturated Fatty Acids From Plant and Animal Sources With Total and Cause-Specific Mortality in Two US Prospective Cohort Studies. Circ Res. 2019;124(8):1266-75.

13. Hernández-Alonso P, Salas-Salvadó J, Ruiz-Canela M, Corella D, Estruch R, Fitó M, et al. High dietary protein intake is associated with an increased body weight and total death risk. Clin Nutr. 2016;35(2):496-506.

14. Ho FK, Gray SR, Welsh P, Petermann-Rocha F, Foster H, Waddell H, et al. Associations of fat and carbohydrate intake with cardiovascular disease and mortality: prospective cohort study of UK Biobank participants. BMJ. 2020;368:m688.

15. Huang J, Liao LM, Weinstein SJ, Sinha R, Graubard BI, Albanes D. Association Between Plant and Animal Protein Intake and Overall and Cause-Specific Mortality. JAMA Intern Med. 2020;180(9):1173-84.

16. Kelemen LE, Kushi LH, Jacobs DR, Jr., Cerhan JR. Associations of Dietary Protein with Disease and Mortality in a Prospective Study of Postmenopausal Women. Am J Epidemiol. 2005;161(3):239-49.

17. Kwon Y-J, Hyun DS, Koh SB, Lee JW. Differential relationship between dietary fat and cholesterol on total mortality in Korean population cohorts. J Intern Med. 2021;290(4):866-77.

18. Laake I, Pedersen JI, Selmer R, Kirkhus B, Lindman AS, Tverdal A, Veierød MB. A prospective study of intake of trans-fatty acids from ruminant fat, partially hydrogenated vegetable oils, and marine oils and mortality from CVD. Br J Nutr. 2012;108(4):743-54.

19. Laguna JC, Alegret M, Cofán M, Sánchez-Tainta A, Díaz-López A, Martínez-González MA, et al. Simple sugar intake and cancer incidence, cancer mortality and all-cause mortality: A cohort study from the PREDIMED trial. Clin Nutr. 2021;40(10):5269-77.

20. Levine ME, Suarez JA, Brandhorst S, Balasubramanian P, Cheng CW, Madia F, et al. Low protein intake is associated with a major reduction in IGF-1, cancer, and overall mortality in the 65 and younger but not older population. Cell Metab. 2014;19(3):407-17.

21. Li L, Shan Z, Wan Z, Li R, Geng T, Lu Q, et al. Associations of lower-carbohydrate and lower-fat diets with mortality among people with prediabetes. Am J Clin Nutr. 2022;116(1):206-15.

22. Mao L, Zhang Y, Wang W, Zhuang P, Wu F, Jiao J. Plant-sourced and animal-sourced monounsaturated fatty acid intakes in relation to mortality: a prospective nationwide cohort study. Eur J Nutr. 2020;59(5):1989-98.

23. Meroño T, Zamora-Ros R, Hidalgo-Liberona N, Rabassa M, Bandinelli S, Ferrucci L, et al. Animal Protein Intake Is Inversely Associated With Mortality in Older Adults: The InCHIANTI Study. J Gerontol a Biol Sci Med Sci. 2022;77(9):1866-72.

24. Nagata C, Nakamura K, Wada K, Oba S, Tsuji M, Tamai Y, Kawachi T. Total fat intake is associated with decreased mortality in Japanese men but not in women. J Nutr. 2012;142(9):1713-9.

25. Ricci C, Baumgartner J, Zec M, Kruger HS, Smuts CM. Type of dietary fat intakes in relation to all-cause and cause-specific mortality in US adults: an iso-energetic substitution analysis from the American National Health and Nutrition Examination Survey linked to the US mortality registry. Br J Nutr. 2018;119(4):456-63.

26. Shan Z, Haslam DE, Rehm CD, Song M, Hu FB, Zhang FF, Bhupathiraju SN. Abstract P510: Association of Animal and Plant Protein Intake With Mortality Among US Adults: A Prospective Cohort Study. Circulation. 2020;141(Suppl_1):AP510-AP.

27. Song M, Fung TT, Hu FB, Willett WC, Longo VD, Chan AT, Giovannucci EL. Association of Animal and Plant Protein Intake With All-Cause and Cause-Specific Mortality. JAMA Intern Med. 2016;176(10):1453-63.

28. Sun Y, Liu B, Snetselaar LG, Wallace RB, Shadyab AH, Kroenke CH, et al. Association of Major Dietary Protein Sources With All-Cause and Cause-Specific Mortality: Prospective Cohort Study. J Am Heart Assoc. 2021;10(5):e015553.

29. Virtanen HEK, Voutilainen S, Koskinen TT, Mursu J, Kokko P, Ylilauri MPT, et al. Dietary proteins and protein sources and risk of death: the Kuopio Ischaemic Heart Disease Risk Factor Study. Am J Clin Nutr. 2019;109(5):1462-71.

30. Wakai K, Naito M, Date C, Iso H, Tamakoshi A. Dietary intakes of fat and total mortality among Japanese populations with a low fat intake: the Japan Collaborative Cohort (JACC) Study. Nutr Metab (Lond). 2014;11(1):12.

31. Wang DD, Li Y, Chiuve SE, Stampfer MJ, Manson JE, Rimm EB, et al. Association of Specific Dietary Fats With Total and Cause-Specific Mortality. JAMA Intern Med. 2016;176(8):1134-45.

32. Wu F, Mao L, Zhuang P, Chen X, Jiao J, Zhang Y. Plant-sourced cooking oil consumption is associated with lower total mortality in a longitudinal nationwide cohort study. Clin Nutr. 2020;39(12):3703-10.

33. Zeng X, Li X, Zhang Z, Li H, Wang Y, Zhu Y, et al. A prospective study of carbohydrate intake and risk of all-cause and specific-cause mortality. Eur J Nutr. 2022;61(6):3149-60.

34. Zhao Y, Li Y, Wang W, Song Z, Zhuang Z, Li D, et al. Low-carbohydrate diets, low-fat diets, and mortality in middle-aged and older people: A prospective cohort study. J Intern Med. 2023;294(2):203-15.

35. Zhou C, Yang S, Zhang Y, Wu Q, Ye Z, Liu M, et al. Relations of Variety and Quantity of Dietary Proteins Intake from Different Sources with Mortality Risk: A Nationwide Population-Based Cohort. J Nutr Health Aging. 2022;26(12):1078-86.

36. Zhuang P, Zhang Y, He W, Chen X, Chen J, He L, et al. Dietary Fats in Relation to Total and Cause-Specific Mortality in a Prospective Cohort of 521 120 Individuals With 16 Years of Follow-Up. Circ Res. 2019a;124(5):757-68.

37. Zhuang P, Cheng L, Wang J, Zhang Y, Jiao J. Saturated Fatty Acid Intake Is Associated with Total Mortality in a Nationwide Cohort Study. J Nutr. 2019b;149(1):68-77.

Excluded References

44. Zeng L, Brignardello-Petersen R, Hultcrantz M, Siemieniuk RAC, Santesso N, Traversy G, et al. GRADE guidelines 32: GRADE offers guidance on choosing targets of GRADE certainty of evidence ratings. J Clin Epidemiol. 2021;137:163-75.

45. Acosta S, Johansson A, Drake I. Diet and Lifestyle Factors and Risk of Atherosclerotic Cardiovascular Disease—A Prospective Cohort Study. Nutrients. 2021;13(11):3822.

46. Adebamowo SN, AlEssa HB, Spiegelman D, Rexrode KM, Willett WC. Abstract P317: Dietary Carbohydrate Quantity and Quality Measures and Risk of Stroke. Circulation. 2016;133(suppl_1):AP317-AP.

47. Aglago EK, Huybrechts I, Murphy N, Casagrande C, Nicolas G, Pischon T, et al. Consumption of Fish and Long-chain n-3 Polyunsaturated Fatty Acids Is Associated With Reduced Risk of Colorectal Cancer in a Large European Cohort. CGH. 2020;18(3):654-66.e6.

48. Akam EY, Murff HJ, Xiang Y-B, Khankari NK, Cai H, Shu XO, et al. Abstract 3011: Dietary fat, fatty acids, and ovarian cancer risk: Preliminary findings from the Shanghai Women’s Health Study. Cancer Res. 2017;77(13_Supplement):3011-.

49. Akter S, Mizoue T, Nanri A, Goto A, Noda M, Sawada N, et al. Low carbohydrate diet and all cause and cause-specific mortality. Clin Nutr. 2021;40(4):2016-24.

50. AlEssa H, Cohen R, Adebamowo SN, Malik VS, Rimm EB, Manson JE, et al. Abstract MP23: Carbohydrate Quality and Quantity and Risk of Coronary Heart Disease Among US Women. Circulation. 2016;133(suppl_1):AMP23-AMP.

51. AlEssa H, Bhupathiraju S, Malik V, Wedick N, Campos H, Rosner B, et al. Abstract 20: Carbohydrate Quality, Measured Using Multiple Carbohydrate Quality Metrics, is Negatively Associated with Risk of Type 2 Diabetes in US Women. Circulation. 2015;131(suppl_1):A20-A.

52. AlEssa HB, Bhupathiraju SN, Malik VS, Wedick NM, Campos H, Rosner B, et al. Carbohydrate quality and quantity and risk of type 2 diabetes in US women12. The American Journal of Clinical Nutrition. 2015;102(6):1543-53.

53. AlEssa HB, Cohen R, Malik VS, Adebamowo SN, Rimm EB, Manson JE, et al. Carbohydrate quality and quantity and risk of coronary heart disease among US women and men. The American Journal of Clinical Nutrition. 2018;107(2):257-67.

54. Allen NE, Key TJ, Appleby PN, Travis RC, Roddam AW, Tjønneland A, et al. Animal foods, protein, calcium and prostate cancer risk: the European Prospective Investigation into Cancer and Nutrition. Br J Cancer. 2008;98(9):1574-81.

55. Allen NE, Roddam AW, Sieri S, Boeing H, Jakobsen MU, Overvad K, et al. A prospective analysis of the association between macronutrient intake and renal cell carcinoma in the European Prospective Investigation into Cancer and Nutrition. Int J Cancer. 2009;125(4):982-7.

56. Allen NE, Appleby PN, Key TJ, Bueno-de-Mesquita HB, Ros MM, Kiemeney LALM, et al. Macronutrient intake and risk of urothelial cell carcinoma in the European prospective investigation into cancer and nutrition. Int J Cancer. 2013;132(3):635-44.

57. Alperovich M, Neuman MI, Willett WC, Curhan GC. Fatty acid intake and the risk of community-acquired pneumonia in U.S. women. Nutrition. 2007;23(3):196-202.

58. Amiano P, Machon M, Molina E, Chirlaque M, Quiros J, Moreno C, et al., editors. INTAKE OF LONG-CHAIN OMEGA-3 FATTY ACIDS AND RISK OF CORONARY HEART DISEASE IN THE SPANISH EPIC COHORT STUDY. Ann Nutr Metab; 2013: KARGER ALLSCHWILERSTRASSE 10, CH-4009 BASEL, SWITZERLAND.

59. Ananthakrishnan AN, Khalili H, Higuchi LM, de Silva PS, Fuchs C, Richter JM, Chan AT. Mo1347 Intake of Dietary Fat and Risk of Crohn's Disease and Ulcerative Colitis Among Women. Gastroenterology. 2013;5(144):S-643.

60. Anderson JJ, Gray SR, Welsh P, Mackay DF, Celis-Morales CA, Lyall DM, et al. The associations of sugar-sweetened, artificially sweetened and naturally sweet juices with all-cause mortality in 198,285 UK Biobank participants: a prospective cohort study. BMC Medicine. 2020;18(1):97.

61. InterAct Consortium I. Investigation of gene–diet interactions in the incretin system and risk of type 2 diabetes: the EPIC-InterAct study. Diabetologia. 2016;59(12):2613-21.

62. Ardisson Korat AV, Li Y, Sacks F, Rosner B, Willett WC, Hu FB, Sun Q. Dairy fat intake and risk of type 2 diabetes in 3 cohorts of US men and women. The American Journal of Clinical Nutrition. 2019;110(5):1192-200.

63. Arem HE, Mayne ST, Sampson J, Risch H, Stolzenberg-Solomon RZ. Abstract 4809: Dietary fat intake and risk of pancreatic cancer in the Prostate, Lung, Colorectal and Ovarian (PLCO) Cancer Screening Trial. Cancer Res. 2013;73(8_Supplement):4809-.

64. Arem H, Mayne ST, Sampson J, Risch H, Stolzenberg-Solomon RZ. Dietary fat intake and risk of pancreatic cancer in the Prostate, Lung, Colorectal and Ovarian Cancer Screening Trial. Ann Epidemiol. 2013;23(9):571-5.

65. Arthur AE, Goss AM, Demark-Wahnefried W, Mondul AM, Fontaine KR, Chen YT, et al. Higher carbohydrate intake is associated with increased risk of all-cause and disease-specific mortality in head and neck cancer patients: results from a prospective cohort study. Int J Cancer. 2018;143(5):1105-13.

66. Ax EH, Cederholm T, Grundmark B, Bill-Axelson A, Becker W, Holmberg L, et al. Dietary Patterns and prostate cancer risk: a population based cohort study in elderly Swedish men. The FASEB Journal. 2013;27(S1):847.8-.8.

67. Ax E, Garmo H, Grundmark B, Bill-Axelson A, Holmberg L, Becker W, et al. Dietary Patterns and Prostate Cancer Risk: Report from the Population Based ULSAM Cohort Study of Swedish Men. Nutr Cancer. 2014;66(1):77-87.

68. Bahadoran Z, Mirmiran P, Tohidi M, Azizi F. Longitudinal Associations of High-Fructose Diet with Cardiovascular Events and Potential Risk Factors: Tehran Lipid and Glucose Study. Nutrients. 2017;9(8):872.

69. Bamia C, Siakavellas S, Bamias G, Trichopoulou A, editors. Dietary intake and gastric cancer risk in the Greek EPIC cohort study. Ann Nutr Metab; 2011: KARGER ALLSCHWILERSTRASSE 10, CH-4009 BASEL, SWITZERLAND.

70. Bandera EV, Freudenheim JL, Marshall JR, Zielezny M, Priore RL, Brasure J, et al. Diet and alcohol consumption and lung cancer risk in the New York State Cohort (United States). CCC. 1997;8:828-40.

71. P J R Banim, Luben R, Sharp SJ, Wareham NJ, Khaw KT, Hart AR. The role of dietary cholesterol and transfatty acids in the aetiology of gallstones: a uk prospective cohort study (EPIC-Norfolk) using 7-day food diaries. Gut. 2011;60(Suppl 1):A225.

72. Banim PR, Luben R, Khaw K-T, Hart A. Sa1382 Dietary Saturated Fatty Acids Are Positively Associated With the Development of Pancreatic Cancer -Data From a UK Prospective Study (EPIC) Using 7-Day Food Diaries. Gastroenterology. 2014;146(5, Supplement 1):S-279.

73. Banim P, Luben R, Khaw K-T, Wareham N, Hart A. PTH-101 Dietary Saturated Fatty Acids Are Positively Associated With The Development Of Pancreatic Cancer – Data From A Uk Prospective Study (epic) Using 7-day Food Diaries. Gut. 2014;63(Suppl 1):A255-A.

74. Banim PR, Luben R, Sharp SJ, Wareham N, Khaw K-T, Hart A. The Role of Dietary Cholesterol and Transfatty Acids in the Aetiology of Gallstones. A UK Prospective Cohort Study (EPIC-Norfolk) Using 7-Day Food Diaries. Gastroenterology. 2011;140(5, Supplement 1):S-447.

75. Bao W, Li S, Chavarro JE, Tobias DK, Zhu Y, Hu FB, Zhang C. Low Carbohydrate–Diet Scores and Long-term Risk of Type 2 Diabetes Among Women With a History of Gestational Diabetes Mellitus: A Prospective Cohort Study. Diabetes Care. 2015;39(1):43-9.

76. Barbour MF, Ashraf F, Roberts MB, Allison M, Martin L, Johnson K, et al. Abstract 11363: Association of Dietary Protein, Animal and Vegetable Protein With the Incidence of Heart Failure Among Postmenopausal Women. Circulation. 2016;134(suppl_1):A11363-A.

77. Barrett-Connor E, Friedlander NJ. Dietary fat, calories, and the risk of breast cancer in postmenopausal women: a prospective population-based study. J Am Coll Nutr. 1993;12(4):390-9.

78. Bassett JK, Hodge AM, English DR, MacInnis RJ, Giles GG. Plasma phospholipids fatty acids, dietary fatty acids, and breast cancer risk. CCC. 2016;27(6):759-73.

79. Bassett JK, Severi G, Hodge AM, MacInnis RJ, Gibson RA, Hopper JL, et al. Plasma phospholipid fatty acids, dietary fatty acids and prostate cancer risk. Int J Cancer. 2013;133(8):1882-91.

80. Beasley JM, LaCroix AZ, Larson JC, Huang Y, Neuhouser ML, Tinker LF, et al. Biomarker-calibrated protein intake and bone health in the Women's Health Initiative clinical

trials and observational study123. The American Journal of Clinical Nutrition. 2014;99(4):934-40.

81. Beasley JM, Newcomb PA, Trentham-Dietz A, Hampton JM, Bersch AJ, Passarelli MN, et al. Post-diagnosis dietary factors and survival after invasive breast cancer. Breast Cancer Res Treat. 2011;128(1):229-36.

82. Belin RJ, Greenland P, Martin L, Oberman A, Tinker L, Robinson J, et al. Fish Intake and the Risk of Incident Heart Failure. Circulation: Heart Failure. 2011;4(4):404-13.

83. Belle FN, Kampman E, McTiernan A, Bernstein L, Baumgartner K, Baumgartner R, et al. Dietary Fiber, Carbohydrates, Glycemic Index, and Glycemic Load in Relation to Breast Cancer Prognosis in the HEAL Cohort. CEBP. 2011;20(5):890-9.

84. Benetou V, Orfanos P, Zylis D, Sieri S, Contiero P, Tumino R, et al. Diet and hip fractures among elderly Europeans in the EPIC cohort. Eur J Clin Nutr. 2011;65(1):132-9.

85. Benito-Garcia E, Feskanich D, Hu FB, Mandl LA, Karlson EW. Protein, iron, and meat consumption and risk for rheumatoid arthritis: a prospective cohort study. Arthritis Res Ther. 2007;9(1):R16.

86. Bernstein AM, Pan A, Rexrode KM, Stampfer M, Hu FB, Mozaffarian D, Willett WC. Dietary Protein Sources and the Risk of Stroke in Men and Women. Stroke. 2012;43(3):637-44.

87. Bertrand KA, Giovannucci E, Zhang SM, Birmann BM. Abstract A73: Body mass index, diet, and risk of non-Hodgkin lymphoma in the Nurses' Health Study and Health Professionals Follow-Up Study. Cancer Prev Res. 2014;4(10_Supplement):A73-A.

88. Bertrand KA, Giovannucci E, Rosner BA, Zhang SM, Laden F, Birmann BM. Dietary fat intake and risk of non-Hodgkin lymphoma in 2 large prospective cohorts. The American Journal of Clinical Nutrition. 2017;106(2):650-6.

89. Beulens JW, de Bruijne LM, Stolk RP, Peeters PH, Bots ML, Grobbee DE, van der Schouw YT. High dietary glycemic load and glycemic index increase risk of cardiovascular disease among middle-aged women: a population-based follow-up study. J Am Coll Cardiol. 2007;50(1):14-21.

90. Beydoun MA, Kaufman JS, Sloane PD, Heiss G, Ibrahim J. n-3 Fatty acids, hypertension and risk of cognitive decline among older adults in the Atherosclerosis Risk in Communities (ARIC) study. Public Health Nutr. 2008;11(1):17-29.

91. Blank MM, Wentzensen N, Murphy MA, Hollenbeck A, Park Y. Dietary fat intake and risk of ovarian cancer in the NIH-AARP Diet and Health Study. Br J Cancer. 2012;106(3):596-602.

92. Boden-Albala B, Elkind MSV, White H, Szumski A, Paik MC, Sacco RL. Dietary Total Fat Intake and Ischemic Stroke Risk: The Northern Manhattan Study. Neuroepidemiology. 2009;32(4):296-301.

93. Boeke CE, Eliassen AH, Chen WY, Cho E, Holmes MD, Rosner B, et al. Abstract 144: Dietary fat and cholesterol intake in relation to fatal breast cancer. Cancer Res. 2013;73(8_Supplement):144-.

94. Boniface DR, Tefft ME. Dietary fats and 16-year coronary heart disease mortality in a cohort of men and women in Great Britain. Eur J Clin Nutr. 2002;56(8):786-92.

95. Bork CS, Jakobsen M, Lundbye-Christensen S, Schmidt EB, Overvad K. Dietary intake of alpha-linolenic acid and risk of myocardial infarction: A Danish cohort study. Eur Heart J. 2015;36(Suppl. 1):724-5.

96. Bork CS, Calder PC, Jakobsen MU, Lasota AN, Lundbye-Christensen S, Overvad K, et al. Intake of α-linolenic acid is not consistently associated with a lower risk of peripheral artery disease: results from a Danish cohort study. Br J Nutr. 2019;122(1):86-92.

97. Bork CS, Venø SK, Lundbye-Christensen S, Jakobsen MU, Tjønneland A, Schmidt EB, Overvad K. Dietary Intake of α-Linolenic Acid Is Not Appreciably Associated with Risk of Ischemic Stroke among Middle-Aged Danish Men and Women. J Nutr. 2018;148(6):952-8.

98. Bork C, Venø SK, Lasota AN, Lundbye-Christensen S, Overvad K, Schmidt EB. Alpha-Linolenic Acid Intake And The Risk Of Atherosclerotic Cardiovascular Disease. Atherosclerosis. 2019;287:e83.

99. Bork CS, Lundbye-Christensen S, Venø SK, Lasota AN, Tjønneland A, Schmidt EB, Overvad K. Intake of marine and plant-derived n-3 fatty acids and development of atherosclerotic cardiovascular disease in the Danish Diet, Cancer and Health cohort. Eur J Nutr. 2023;62(3):1389-401.

100. Borugian MJ, Sheps SB, Kim-Sing C, Van Patten C, Potter JD, Dunn B, et al. Insulin, macronutrient intake, and physical activity: are potential indicators of insulin resistance associated with mortality from breast cancer? CEBP. 2004;13(7):1163-72.

101. Bostick RM, Potter JD, Kushi LH, Sellers TA, Steinmetz KA, McKenzie DR, et al. Sugar, meat, and fat intake, and non-dietary risk factors for colon cancer incidence in Iowa women (United States). CCC. 1994;5(1):38-52.

102. Bradbury KE, Murphy N, Key TJ. Diet and colorectal cancer in UK Biobank: a prospective study. Int J Epidemiol. 2019;49(1):246-58.

103. Brasky TM, Rodabough RJ, Liu J, Kurta ML, Wise LA, Orchard TS, et al. Long-chain ω-3 fatty acid intake and endometrial cancer risk in the Women's Health Initiative234. The American Journal of Clinical Nutrition. 2015;101(4):824-34.

104. Brasky TM, Sponholtz TR, Palmer JR, Rosenberg L, Ruiz-Narváez EA, Wise LA. Associations of Dietary Long-Chain ω-3 Polyunsaturated Fatty Acids and Fish Consumption With Endometrial Cancer Risk in the Black Women's Health Study. American Journal of Epidemiology. 2016;183(3):199-209.

105. Brennan SF, Cantwell MM, Dwek MV, Leathem AJ, Lentjes M, Perkins KA, et al. Dietary patterns, fat and breast cancer recurrence in a UK breast cancer population. Proc Nutr Soc. 2012;71(OCE2):E103.

106. Burger K, Beulens J, Boer J, Spijkerman A, van der A D, editors. Dietary glycemic load and glycemic index and risk of coronary heart disease and stroke. Ann Nutr Metab; 2011: KARGER ALLSCHWILERSTRASSE 10, CH-4009 BASEL, SWITZERLAND.

107. Buyken AE, Flood V, Empson M, Rochtchina E, Barclay AW, Brand-Miller J, Mitchell P. Carbohydrate nutrition and inflammatory disease mortality in older adults123. The American Journal of Clinical Nutrition. 2010;92(3):634-43.

108. Byrne C, Rockett H, Holmes M. Dietary fat, fat subtypes, and breast cancer risk: lack of an association among postmenopausal women with no history of benign breast disease. CEBP. 2002;11(3):261-5.

109. Cai H, Sobue T, Kitamura T, Ishihara J, Sawada N, Iwasaki M, et al. Association between meat and saturated fatty acid intake and lung cancer risk: The Japan Public Health Center-based prospective study. Int J Cancer. 2020;147(11):3019-28.

110. Cai H, Sobue T, Kitamura T, Ishihara J, Nanri A, Mizoue T, et al. Low-carbohydrate diet and risk of cancer incidence: The Japan Public Health Center-based prospective study. Cancer Sci. 2022;113(2):744-55.

111. Calvert* J, Shu X, Cai H, Xiang Y-B, Li H, Miller N, et al. PD12-05 ANIMAL AND NON-ANIMAL PROTEIN INTAKES AND RISK OF INCIDENT KIDNEY STONES: A REPORT FROM THE SHANGHAI MEN'S AND WOMEN'S HEALTH STUDIES. Urol J. 2019;201(Supplement 4):e222-e.

112. Carballo-Casla A, García-Esquinas E, Banegas JR, Rodríguez-Artalejo F, Ortolá R. Fish consumption, omega-3 fatty acid intake, and risk of pain: the Seniors-ENRICA-1 cohort. Clin Nutr. 2022;41(11):2587-95.

113. Castor LL, Bradlee ML, Singer MR, Moore LL. Animal and Plant Proteins Impact Risk of Type 2 Diabetes Mellitus in The Framingham Study. The FASEB Journal. 2017;31(S1):652.21-.21.

114. Castro-Quezada I, Sánchez-Villegas A, Díaz-González V, Ruíz-López MD, Artacho R, Álvarez-Pérez J, et al. Relationship between dietary glycemic index, dietary glycemic load and major cardiovascular events in the PREDIMED study. Eur Geriatr Med. 2013;4:S128-S9.

115. Castro-Quezada I, Sánchez-Villegas A, Estruch R, Salas-Salvadó J, Corella D, Schröder H, et al. A High Dietary Glycemic Index Increases Total Mortality in a Mediterranean Population at High Cardiovascular Risk. PLOS ONE. 2014;9(9):e107968.

116. Castro-Quezada I, Sánchez-Villegas A, Martínez-González MÁ, Salas-Salvadó J, Corella D, Estruch R, et al. Glycemic index, glycemic load and invasive breast cancer incidence in postmenopausal women: The PREDIMED study. Eur J Cancer Prev. 2016;25(6).

117. Castro-Quezada I, Sanchez-Villegas A, Ruiz-Lopez M, Artacho R, Álvarez Pérez J, Estruch R, et al. Glycemic index and glycemic load and risk of mortality: the predimed study. Ann Nutr Metab. 2013.

118. Chan JM, Pietinen P, Virtanen M, Malila N, Tangrea J, Albanes D, Virtamo J. Diet and prostate cancer risk in a cohort of smokers, with a specific focus on calcium and phosphorus (Finland). CCC. 2000;11:859-67.

119. Chan R, Leung J, Woo J. High Protein Intake Is Associated with Lower Risk of All-Cause Mortality in Community-Dwelling Chinese Older Men and Women. J Nutr Health Aging. 2019;23(10):987-96.

120. Chang ET, Lee VS, Canchola AJ, Clarke CA, Purdie DM, Reynolds P, et al. Diet and risk of ovarian cancer in the California Teachers Study cohort. Am J Epidemiol. 2007;165(7):802-13.

121. Chen M, Li Y, Sun Q, Pan A, Manson JE, Rexrode KM, et al. Dairy fat and risk of cardiovascular disease in 3 cohorts of US adults. The American Journal of Clinical Nutrition. 2016;104(5):1209-17.

122. Chen M, Sun Q, Pan A, Li Y, Manson JE, Rexrode KM, et al. Abstract P282: Dairy Fat and Risk of Cardiovascular Disease in 3 Cohorts of US Adults. Circulation. 2016;133(suppl_1):AP282-AP.

123. Chen T, Song L, Zhong X, Zhu Q, Huo J, Chen J, et al. Dietary polyunsaturated fatty acids intake, air pollution, and the risk of lung cancer: A prospective study in UK biobank. Sci Total Environ. 2023;882:163552.

124. Chiu C-J, Milton RC, Klein R, Gensler G, Taylor A. Dietary carbohydrate and the progression of age-related macular degeneration: a prospective study from the Age-Related Eye Disease Study23. The American Journal of Clinical Nutrition. 2007;86(4):1210-8.

125. Chiuve SE, Rimm EB, Sandhu RK, Rexrode KM, Manson JE, Willett WC, Albert CM. Abstract 9238: Fatty Acids and Sudden Cardiac Death. Circulation. 2011;124(suppl_21):A9238-A.

126. Chyou PH, Nomura AM, Stemmermann GN, Kato I. Lung cancer: a prospective study of smoking, occupation, and nutrient intake. Arch Environ Health. 1993;48(2):69-72.

127. Coleman HG, Kitahara CM, Murray LJ, Dodd KW, Black A, Stolzenberg-Solomon RZ, Cantwell MM. Dietary Carbohydrate Intake, Glycemic Index, and Glycemic Load and Endometrial Cancer Risk: A Prospective Cohort Study. American Journal of Epidemiology. 2013;179(1):75-84.

128. Courand P-Y, Lesiuk C, Milon H, Defforges A, Fouque D, Harbaoui B, Lantelme P. Association Between Protein Intake and Mortality in Hypertensive Patients Without Chronic Kidney Disease in the OLD-HTA Cohort. Hypertension. 2016;67(6):1142-9.

129. Cross AJ, Leitzmann MF, Subar AF, Thompson FE, Hollenbeck AR, Schatzkin A. A Prospective Study of Meat and Fat Intake in Relation to Small Intestinal Cancer. Cancer Res. 2008;68(22):9274-9.

130. Crowe FL, Key TJ, Appleby PN, Travis RC, Overvad K, Jakobsen MU, et al. Dietary fat intake and risk of prostate cancer in the European Prospective Investigation into Cancer and Nutrition. Am J Clin Nutr. 2008;87(5):1405-13.

131. Cui X, Rosner B, Willett WC, Hankinson SE. Dietary Fat, Fiber, and Carbohydrate Intake in Relation to Risk of Endometrial Cancer. CEBP. 2011;20(5):978-89.

132. Cui Z, Wu M, Liu K, Wang Y, Kang T, Meng S, Meng H. Associations between Conventional and Emerging Indicators of Dietary Carbohydrate Quality and New-Onset Type 2 Diabetes Mellitus in Chinese Adults. Nutrients. 2023;15(3):647.

133. Curhan GC, Willett WC, Knight EL, Stampfer MJ. Dietary factors and the risk of incident kidney stones in younger women: Nurses' Health Study II. Arch Intern Med. 2004;164(8):885-91.

134. Dal Maso L, Zucchetto A, Talamini R, Serraino D, Stocco CF, Vercelli M, et al. Effect of obesity and other lifestyle factors on mortality in women with breast cancer. Int J Cancer. 2008;123(9):2188-94.

135. Daniel CR, McCullough ML, Patel RC, Jacobs EJ, Flanders WD, Thun MJ, Calle EE. Dietary Intake of ω-6 and ω-3 Fatty Acids and Risk of Colorectal Cancer in a Prospective Cohort of U.S. Men and Women. CEBP. 2009;18(2):516-25.

136. Daniel CR, Sinha R, Park Y, Graubard BI, Hollenbeck AR, Morton LM, Cross AJ. Meat Intake Is Not Associated with Risk of Non-Hodgkin Lymphoma in a Large Prospective Cohort of U.S. Men and Women. J Nutr. 2012;142(6):1074-80.

137. Dargent-Molina P, Sabia S, Touvier M, Kesse E, Bréart G, Clavel-Chapelon F, Boutron-Ruault MC. Proteins, Dietary Acid Load, and Calcium and Risk of Postmenopausal Fractures in the E3N French Women Prospective Study*. JBMR. 2009;23(12):1915-22.

138. Goede Jd, Soedamah-Muthu SS, Trichia E, Geleijnse JM, Kromhout D. Abstract P061: Dietary Intake of Saturated Fat by Food Source and Incident Coronary Heart Disease: the Zutphen Elderly Study. Circulation. 2015;131(suppl_1):AP061-AP.

139. de Goede J, Verschuren WMM, Boer JMA, Kromhout D, Geleijnse JM. Alpha-Linolenic Acid Intake and 10-Year Incidence of Coronary Heart Disease and Stroke in 20,000 Middle-Aged Men and Women in The Netherlands. PLOS ONE. 2011;6(3):e17967.

140. de Goede J, Geleijnse JM, Boer JMA, Kromhout D, Verschuren WMM. Marine (n-3) Fatty Acids, Fish Consumption, and the 10-Year Risk of Fatal and Nonfatal Coronary Heart Disease in a Large Population of Dutch Adults with Low Fish Intake. J Nutr. 2010;140(5):1023-8.

141. de Goede J, Verschuren WMM, Boer JMA, Kromhout D, Geleijnse JM. Gender-Specific Associations of Marine n-3 Fatty Acids and Fish Consumption with 10-Year Incidence of Stroke. PLOS ONE. 2012;7(4):e33866.

142. de Koning L, Fung TT, Liao X, Chiuve SE, Rimm EB, Willett WC, et al. Low-carbohydrate diet scores and risk of type 2 diabetes in men. Am J Clin Nutr. 2011;93(4):844-50.

143. De Koning L, Fung TT, Rimm EB, Willett WC, Hu FB. Low-carbohydrate diets and the risk of type 2 diabetes among men. Diabetes. 2010.

144. de la Cruz-Ares S, Gutiérrez-Mariscal FM, Alcalá-Díaz JF, Quintana-Navarro GM, Podadera-Herreros A, Cardelo MP, et al. Quality and Quantity of Protein Intake Influence Incidence of Type 2 Diabetes Mellitus in Coronary Heart Disease Patients: From the CORDIOPREV Study. Nutrients. 2021;13(4):1217.

145. Otto MCdO, Wu JHY, Baylin A, Vaidya D, Rich SS, Tsai MY, et al. Circulating and Dietary Omega‐3 and Omega‐6 Polyunsaturated Fatty Acids and Incidence of CVD in the Multi‐Ethnic Study of Atherosclerosis. JAHA. 2013;2(6):e000506.

146. de Oliveira Otto MC, Mozaffarian D, Kromhout D, Bertoni AG, Sibley CT, Jacobs DR, Nettleton JA. Dietary intake of saturated fat by food source and incident cardiovascular disease: the Multi-Ethnic Study of Atherosclerosis. The American Journal of Clinical Nutrition. 2012;96(2):397-404.

147. Debras C, Chazelas E, Srour B, Julia C, Kesse-Guyot E, Zelek L, et al. Glycaemic index, glycaemic load and cancer risk: results from the prospective NutriNet-Santé cohort. Int J Epidemiol. 2022;51(1):250-64.

148. Debras C, Chazelas E, Srour B, Kesse-Guyot E, Julia C, Zelek L, et al. Total and added sugar intakes, sugar types, and cancer risk: results from the prospective NutriNet-Santé cohort. The American Journal of Clinical Nutrition. 2020;112(5):1267-79.

149. DeChristopher LR, Tucker KL. Excess free fructose, high-fructose corn syrup and adult asthma: the Framingham Offspring Cohort. Br J Nutr. 2018;119(10):1157-67.

150. Deschasaux M, Pouchieu C, His M, Hercberg S, Latino-Martel P, Touvier M. Dietary Total and Insoluble Fiber Intakes Are Inversely Associated with Prostate Cancer Risk1, 2, 3. J Nutr. 2014;144(4):504-10.

151. Devore EE, Grodstein F, van Rooij FJA, Hofman A, Rosner B, Stampfer MJ, et al. Dietary intake of fish and omega-3 fatty acids in relation to long-term dementia risk. The American Journal of Clinical Nutrition. 2009;90(1):170-6.

152. DiGiuseppe D, Wallin A, Bottai M, Askling J, Wolk A. Long-term intake of dietary long-chain n-3 polyunsaturated fatty acids and risk of rheumatoid arthritis: a prospective cohort study of women. Ann Rheum Dis. 2014;73(11):1949.

153. Dianatinasab M, Wesselius A, Salehi-Abargouei A, Yu EYW, Fararouei M, Brinkman M, et al. Dietary fats and their sources in association with the risk of bladder cancer: A pooled analysis of 11 prospective cohort studies. Int J Cancer. 2022;151(1):44-55.

154. Dijkstra SC, Brouwer IA, van Rooij FJA, Hofman A, Witteman JCM, Geleijnse JM. Intake of very long chain n-3 fatty acids from fish and the incidence of heart failure: the Rotterdam Study. Eur J Heart Fail. 2009;11(10):922-8.

155. Djoussé L, Gaziano JM, Buring JE, Lee IM. Dietary omega-3 fatty acids and fish consumption and risk of type 2 diabetes. The American Journal of Clinical Nutrition. 2011;93(1):143-50.

156. Dong C, Chan SSM, Jantchou P, Racine A, Oldenburg B, Weiderpass E, et al. Meat Intake Is Associated with a Higher Risk of Ulcerative Colitis in a Large European Prospective Cohort Studyø. J Crohns Colitis. 2022;16(8):1187-96.

157. dos Santos ALT, Weiss T, Duarte CK, Cantori GA, de Azevedo MJ, Gross JL, Zelmanovitz T, editors. Association between Nutritional Recommendations for Cardiovascular Disease and a Lower Incidence of Cardiac Events in Patients with Type 2 Diabetes. Diabetes; 2010: AMER DIABETES ASSOC 1701 N BEAUREGARD ST, ALEXANDRIA, VA 22311-1717 USA.

158. dos Santos ALT, Weiss T, Duarte CK, Gross JL, de Azevedo MJ, Zelmanovitz T. Dietary fat composition and cardiac events in patients with type 2 diabetes. Atherosclerosis. 2014;236(1):31-8.

159. Dow C, Mangin M, Balkau B, Affret A, Boutron-Ruault M-C, Clavel-Chapelon F, et al., editors. Fatty acid consumption and incident type 2 diabetes: evidence from the E3N cohort study. Diabetologia; 2016: SPRINGER 233 SPRING ST, NEW YORK, NY 10013 USA.

160. Dow C, Mangin M, Balkau B, Affret A, Boutron-Ruault M-C, Clavel-Chapelon F, et al. Fatty acid consumption and incident type 2 diabetes: an 18-year follow-up in the female E3N (Etude Epidémiologique auprès des femmes de la Mutuelle Générale de l'Education Nationale) prospective cohort study. Br J Nutr. 2016;116(10):1807-15.

161. Duarte-Salles T, Fedirko V, Trichopoulou A, Bamia C, Romieu I, Jenab M, Group obotE. Abstract 4830: Dietary intake of dairy foods, calcium, fat, protein and risk of hepatocellular carcinoma in the European Prospective Investigation into Cancer and Nutrition (EPIC) study. Cancer Res. 2013;73(8_Supplement):4830-.

162. Dwyer JT, Madans JH, Turnbull B, Cornoni-Huntley J, Dresser C, Everett DF, Perrone RD. Diet, indicators of kidney disease, and later mortality among older persons in the NHANES I Epidemiologic Follow-Up Study. American journal of public health. 1994;84(8):1299-303.

163. Eaton CB, Barbour MF, Ashraf F, Roberts M, Martin LW, Allison MA, et al. Abstract P170: High Intake of Dietary Protein is Associated With Increased Risk of Heart Failure With Preserved Ejection Fraction. Circulation. 2017;135(suppl_1):AP170-AP.

164. Elstgeest LEM, Schaap LA, Heymans MW, Hengeveld LM, Naumann E, Houston DK, et al. Sex-and race-specific associations of protein intake with change in muscle mass and physical function in older adults: the Health, Aging, and Body Composition (Health ABC) Study. The American Journal of Clinical Nutrition. 2020;112(1):84-95.

165. Engelhart MJ, Geerlings MI, Ruitenberg A, van Swieten JC, Hofman A, Witteman JCM, Breteler MMB. Diet and risk of dementia: Does fat matter? Neurology. 2002;59(12):1915-21.

166. Epstein MM, Kasperzyk JL, Mucci LA, Giovannucci E, Price A, Wolk A, et al. Dietary Fatty Acid Intake and Prostate Cancer Survival in Örebro County, Sweden. American Journal of Epidemiology. 2012;176(3):240-52.

167. Ericson U, Hellstrand S, Brunkwall L, Schulz C-A, Sonestedt E, Wallström P, et al. Food sources of fat may clarify the inconsistent role of dietary fat intake for incidence of type 2 diabetes23. The American Journal of Clinical Nutrition. 2015;101(5):1065-80.

168. Ericson U, Hellstrand S, Brunkwall L, Sonestedt E, Wallstrom P, Gullberg B, et al., editors. Food sources of fat may clarify the earlier inconsistent role of dietary fat intake for incidence of type 2 diabetes. Diabetologia; 2014: SPRINGER 233 SPRING ST, NEW YORK, NY 10013 USA.

169. Esrey KL, Joseph L, Grover SA. Relationship between dietary intake and coronary heart disease mortality: lipid research clinics prevalence follow-up study. J Clin Epidemiol. 1996;49(2):211-6.

170. Estlin AFL, Ahern AL, Griffin SJ, Strelitz J. Modification of cardiovascular disease risk by health behaviour change following type 2 diabetes diagnosis. Diabet Med. 2021;38(10):e14646.

171. Farhadnejad H, Asghari G, Teymoori F, Tahmasebinejad Z, Mirmiran P, Azizi F. Low-carbohydrate diet and cardiovascular diseases in Iranian population: Tehran Lipid and Glucose Study. Nutr Metab Cardiovasc Dis. 2020;30(4):581-8.

172. Farrell SW, DeFina LF, Tintle NL, Leonard D, Cooper KH, Barlow CE, et al. Association of the Omega-3 Index with Incident Prostate Cancer with Updated Meta-Analysis: The Cooper Center Longitudinal Study. Nutrients. 2021;13(2):384.

173. Farvid MS, Cho E, Chen WY, Eliassen AH, Willett WC. Dietary protein sources in early adulthood and breast cancer incidence: prospective cohort study. BMJ : British Medical Journal. 2014;348:g3437.

174. Farvid MS, Eliassen AH, Cho E, Chen WY, Willett WC. Adolescent and Early Adulthood Dietary Carbohydrate Quantity and Quality in Relation to Breast Cancer Risk. CEBP. 2015;24(7):1111-20.

175. Farvid MS, Malekshah AF, Pourshams A, Poustchi H, Sepanlou SG, Sharafkhah M, et al. Dietary Protein Sources and All-Cause and Cause-Specific Mortality: The Golestan Cohort Study in Iran. American Journal of Preventive Medicine. 2017;52(2):237-48.

176. Fitzgerald KC, O'Reilly ÉJ, Falcone GJ, McCullough ML, Park Y, Kolonel LN, Ascherio A. Dietary ω-3 Polyunsaturated Fatty Acid Intake and Risk for Amyotrophic Lateral Sclerosis. JAMA Neurology. 2014;71(9):1102-10.

177. Fitzgerald KC, O' Reilly E, Falcone G, McCullough ML, Le March L, Ascherio A. N-3 and N-6 polyunsaturated fatty acid intake and risk of amyotrophic lateral sclerosis: Pooled results from five cohort studies. Amyotrophic Lateral Sclerosis and Frontotemporal Degeneration. 2013;14:1-63.

178. Flood A, Velie EM, Sinha R, Chaterjee N, Lacey Jr. JV, Schairer C, Schatzkin A. Meat, Fat, and Their Subtypes as Risk Factors for Colorectal Cancer in a Prospective Cohort of Women. American Journal of Epidemiology. 2003;158(1):59-68.

179. Folsom AR, Demissie Z, Harnack L. Glycemic index, glycemic load, and incidence of endometrial cancer: the Iowa women's health study. Nutr Cancer. 2003;46(2):119-24.

180. Forman JP, Choi H, Curhan GC. Fructose and Vitamin C Intake Do Not Influence Risk for Developing Hypertension. JASN. 2009;20(4):863-71.

181. Fradet Y, Meyer F, Bairati I, Shadmani R, Moore L. Dietary fat and prostate cancer progression and survival. Eur Urol. 1999;35(5-6):388-91.

182. Freedman ND, Cross AJ, McGlynn KA, Abnet CC, Park Y, Hollenbeck AR, et al. Association of Meat and Fat Intake With Liver Disease and Hepatocellular Carcinoma in the NIH-AARP Cohort. JNCI. 2010;102(17):1354-65.

183. Fretts AM, King IB, Lemaitre RN, McKnight B, Mozaffarian D, Psaty BM, et al. Plasma phospholipid and dietary α-linolenic acid, mortality, CHD and stroke: the Cardiovascular Health Study. Br J Nutr. 2014;112(7):1206-13.

184. Fretts AM, Mozaffarian D, Siscovick D, Sitlani C, Psaty BM, Rimm EB, et al. Abstract P038: Associations of Plasma Phospholipid and Dietary Alpha Linolenic Acid with Mortality, Coronary Heart Disease and Stroke in Older Adults: The Cardiovascular Health Study. Circulation. 2013;127(suppl_12):AP038-AP.

185. Fretts AM, Mozaffarian D, Siscovick DS, Heckbert SR, McKnight B, King IB, et al. Associations of Plasma Phospholipid and Dietary Alpha Linolenic Acid With Incident Atrial Fibrillation in Older Adults: The Cardiovascular Health Study. JAHA. 2013;2(1):e003814.

186. Friberg E, Wallin A, Wolk A. Sucrose, High-Sugar Foods, and Risk of Endometrial Cancer—a Population-Based Cohort Study. CEBP. 2011;20(9):1831-7.

187. Frost L, Vestergaard P. n-3 Fatty acids consumed from fish and risk of atrial fibrillation or flutter: the Danish Diet, Cancer, and Health Study. Am J Clin Nutr. 2005;81(1):50-4.

188. Fung TT, Meyer HE, Willett WC, Feskanich D. Protein intake and risk of hip fractures in postmenopausal women and men age 50 and older. Osteoporos Int. 2017;28(4):1401-11.

189. Gaeini Z, Bahadoran Z, Mirmiran P, Azizi F. Association of Dietary Fat Pattern and Incidence of Cardiovascular Disease, Hypertension and Chronic Kidney Disease: Tehran Lipid and Glucose Study. IJEM. 2019;20(6):332-43.

190. Gaeini Z, Bahadoran Z, Mirmiran P, Djazayery A. The Association between Dietary Fat Pattern and the Risk of Type 2 Diabetes. Prev Nutr Food Sci. 2019;24(1):1-7.

191. Gago-Dominguez M, Yuan JM, Sun CL, Lee HP, Yu MC. Opposing effects of dietary n-3 and n-6 fatty acids on mammary carcinogenesis: The Singapore Chinese Health Study. Br J Cancer. 2003;89(9):1686-92.

192. Gao J-W, Hao Q-Y, Zhang H-F, Li X-Z, Yuan Z-M, Guo Y, et al. Low-Carbohydrate Diet Score and Coronary Artery Calcium Progression. ATVB. 2021;41(1):491-500.

193. García Gavilan JF, Bulló M, Camacho-Barcia L, Rosique-Esteban N, Hernández-Alonso P, Basora Gallisà J, et al. The relationship between dietary glycaemic, dietary glycemic load and the osteoporotic fracture risk in an elderly Mediterranean population. Obes Facts. 2018;11:309-3010.

194. George SM, Mayne ST, Leitzmann MF, Park Y, Schatzkin A, Flood A, et al. Dietary Glycemic Index, Glycemic Load, and Risk of Cancer: A Prospective Cohort Study. American Journal of Epidemiology. 2009;169(4):462-72.

195. Gerber DA, Stefanick M, Hlatky M, Yang J, Hedlin H, Haring B, Perez M. DIETARY PROTEIN INTAKE AND INCIDENT ATRIAL FIBRILLATION IN POSTMENOPAUSAL WOMEN FROM THE WOMEN's HEALTH INITIATIVE. JACC. 2020;75(11, Supplement 2):5.

196. Ghorbani Z, Hekmatdoost A, Poustchi H, Pourshams A, Malekshah AF, Sharafkhah M, Malekzadeh R. The Association between Dietary Fat Intake and Pancreatic Cancer Risk: A Large Prospective Study. Govaresh. 2017;21(4):221-9.

197. Ghorbani Z, Pourshams A, FAZELTABAR MA, Sharafkhah M, Poustchi H, Hekmatdoost A. Major dietary protein sources in relation to pancreatic cancer: a large prospective study. Arch Iran Med. 2016;19(4):248-56.

198. Gillman MW, Cupples LA, Millen BE, Ellison RC, Wolf PA. Inverse Association of Dietary Fat With Development of Ischemic Stroke in Men. JAMA. 1997;278(24):2145-50.

199. Giovannucci E, Stampfer MJ, Colditz G, Rimm EB, Willett WC. Relationship of diet to risk of colorectal adenoma in men. J Natl Cancer Inst. 1992;84(2):91-8.

200. Glenn AJ, Lo K, Jenkins DJA, Boucher BA, Hanley AJ, Kendall CWC, et al. Relationship Between a Plant-Based Dietary Portfolio and Risk of Cardiovascular Disease: Findings From the Women's Health Initiative Prospective Cohort Study. JAHA. 2021;10(16):e021515.

201. Gnagnarella P, Maisonneuve P, Bellomi M, Rampinelli C, Bertolotti R, Spaggiari L, et al. Nutrient intake and nutrient patterns and risk of lung cancer among heavy smokers: results from the COSMOS screening study with annual low-dose CT. Eur J Epidemiol. 2013;28(6):503-11.

202. Goodwin PJ, Ennis M, Pritchard KI, Koo J, Trudeau ME, Hood N. Diet and breast cancer: evidence that extremes in diet are associated with poor survival. J Clin Oncol. 2003;21(13):2500-7.

203. Gronroos NN, Chamberlain AM, Folsom AR, Soliman EZ, Agarwal SK, Nettleton JA, Alonso A. Fish, Fish-Derived n-3 Fatty Acids, and Risk of Incident Atrial Fibrillation in the Atherosclerosis Risk in Communities (ARIC) Study. PLOS ONE. 2012;7(5):e36686.

204. Guo X, Xu X, Gao J, Wang W, Hou W, Wu X, et al. Twenty-Year Trajectory-Patterns of Percentage Energy From Dietary Fat vs. Carbohydrate Throughout Adult Life and Associations With Cardio-Metabolic Disease and All-Cause Mortality. Front Nutr. 2021;8.

205. Gustafson DR, Bäckman K, Scarmeas N, Stern Y, Manly JJ, Mayeux R, Gu Y. Dietary fatty acids and risk of Alzheimer's disease and related dementias: Observations from the Washington Heights-Hamilton Heights-Inwood Columbia Aging Project (WHICAP). Alzheimer's & Dementia. 2020;16(12):1638-49.

206. Gutierrez-Mariscal FM, Alcalá-Diaz JF, Quintana-Navarro GM, de la Cruz-Ares S, Torres-Peña JD, Cardelo MP, et al. Changes in quantity plant-based protein intake on type 2 diabetes remission in coronary heart disease patients: from the CORDIOPREV study. Eur J Nutr. 2023;62(4):1903-13.

207. Ha K, Sakaki JR, Chun OK. Nutrient Adequacy Is Associated with Reduced Mortality in US Adults. J Nutr. 2021;151(10):3214-22.

208. Haghighatdoost F, Mohammadifard N, Zakeri P, Najafian J, Sadeghi M, Roohafza H, Sarrafzadegan N. Differences in all-cause mortality risk associated with animal and plant dietary protein sources consumption. Scientific Reports. 2023;13(1):3396.

209. Haider S, Grabovac I, Drgac D, Mogg C, Oberndorfer M, Dorner TE. Impact of physical activity, protein intake and social network and their combination on the development of frailty. Eur J Public Health. 2020;30(2):340-6.

210. Halbesma N, Bakker SJL, Jansen DF, Stolk RP, De Zeeuw D, De Jong PE, et al. High Protein Intake Associates with Cardiovascular Events but not with Loss of Renal Function. JASN. 2009;20(8):1797-804.

211. Hall MN, Chavarro JE, Lee I-M, Willett WC, Ma J. A 22-year Prospective Study of Fish, n-3 Fatty Acid Intake, and Colorectal Cancer Risk in Men. CEBP. 2008;17(5):1136-43.

212. Halton TL, Willett WC, Liu S, Manson JE, Albert CM, Rexrode K, Hu FB. Low-carbohydrate-diet score and the risk of coronary heart disease in women. N Engl J Med. 2006;355(19):1991-2002.

213. Hansen-Krone IJ, Enga KF, Njølstad I, Hansen J-B, Braekkan SK. Heart healthy diet and risk of myocardial infarction and venous thromboembolism. The Tromsø Study. 2012;108(09):554-60.

214. Hanyuda A, Wang M, Rosner B, Wiggs JL, Elze T, Pasquale LR, Kang J. Plant-based low carb diet and other risk factors in relation to incident primary open-angle glaucoma characterized by autonomously determined visual field loss patterns. IOVS. 2022;63(7):2268-.

215. Hanyuda A, Rosner BA, Wiggs JL, Willett WC, Tsubota K, Pasquale LR, Kang JH. Low-carbohydrate-diet scores and the risk of primary open-angle glaucoma: data from three US cohorts. Eye. 2020;34(8):1465-75.

216. Harbers MC, Pertiwi K, Soedamah-Muthu SS, Goede Jd, Mölenberg FJ, Wanders AJ, et al. Abstract P215: Plasma and Dietary Linoleic Acid and Diabetes Incidence After Myocardial Infarction. Circulation. 2018;137(suppl_1):AP215-AP.

217. Hartman TJ, McCullough ML, Hodge JM, Gaudet MM, Wang Y, Gapstur SM. Dietary Energy Density, Glycemic Load, Glycemic Index, and Risk for Endometrial Cancer in the CPS-II Nutrition Cohort. CEBP. 2018;27(1):113-5.

218. He D, Sun N, Xiong S, Qiao Y, Ke C, Shen Y. Association between the proportions of carbohydrate and fat intake and hypertension risk: findings from the China Health and Nutrition Survey. J Hypertens. 2021;39(7):1386-92.

219. He F-y, Chen C-g, Lin D-z, Lin X-h, Qi Y-q, Yan L. A greater glycemic load reduction was associated with a lower diabetes risk in pre-diabetic patients who consume a high glycemic load diet. Nutr Res. 2018;53:77-84.

220. He J, Yu S, Fang A, Shen X, Li K. Association between Protein Intake and the Risk of Hypertension among Chinese Men and Women: A Longitudinal Study. Nutrients. 2022;14(6):1276.

221. He K, Merchant A, Rimm EB, Rosner BA, Stampfer MJ, Willett WC, Ascherio A. Dietary fat intake and risk of stroke in male US healthcare professionals: 14 year prospective cohort study. Bmj. 2003;327(7418):777-82.

222. Heath AK, Muller DC, van den Brandt PA, Papadimitriou N, Critselis E, Gunter M, et al. Nutrient-wide association study of 92 foods and nutrients and breast cancer risk. BCR. 2020;22(1):5.

223. Heath A, Muller D, van den Brandt P, Papadimitriou N, Critselis E, Weiderpass E, et al. Nutrient-wide association study of 92 foods and nutrients and breast cancer risk. Proc Nutr Soc. 2020;79(OCE2):E179.

224. Hidaka A, Shimazu T, Sawada N, Yamaji T, Iwasaki M, Sasazuki S, et al. Fish, n–3 PUFA consumption, and pancreatic cancer risk in Japanese: a large, population-based, prospective cohort study12. The American Journal of Clinical Nutrition. 2015;102(6):1490-7.

225. Hisamatsu T, Miura K, Ohkubo T, Yamamoto T, Fujiyoshi A, Miyagawa N, et al. Interaction between dietary marine-derived n-3 fatty acids intake and J-point elevation on the risk of cardiac death: a 24-year follow-up of Japanese men. Heart. 2013;99(14):1024-9.

226. Hisamatsu T, Miura K, Ohkubo T, Yamamoto T, Fujiyoshi A, Miyagawa N, et al. High long-chain n-3 fatty acid intake attenuates the effect of high resting heart rate on cardiovascular mortality risk: A 24-year follow-up of Japanese general population. J Cardiol. 2014;64(3):218-24.

227. Hodge AM, English DR, O'Dea K, Giles GG. Glycemic index and dietary fiber and the risk of type 2 diabetes. Diabetes Care. 2004;27(11):2701-6.

228. Hodge AM, English DR, O'Dea K, Sinclair AJ, Makrides M, Gibson RA, Giles GG. Plasma phospholipid and dietary fatty acids as predictors of type 2 diabetes: interpreting the role of linoleic acid. Am J Clin Nutr. 2007;86(1):189-97.

229. Hodge AM, Williamson EJ, Bassett JK, MacInnis RJ, Giles GG, English DR. Dietary and biomarker estimates of fatty acids and risk of colorectal cancer. Int J Cancer. 2015;137(5):1224-34.

230. Holick CN, Giovannucci EL, Stampfer MJ, Michaud DS. A prospective study of fish, marine fatty acids, and bladder cancer risk among men and women (United States). CCC. 2006;17(9):1163-73.

231. Holmes MD, Stampfer MJ, Colditz GA, Rosner B, Hunter DJ, Willett WC. Dietary factors and the survival of women with breast carcinoma. Cancer. 1999;86(5):826-35.

232. Holmes MD, Wang J, Hankinson SE, Tamimi RM, Chen WY. Protein Intake and Breast Cancer Survival in the Nurses' Health Study. J Clin Oncol. 2017;35(3):325-33.

233. Hopping BN, Erber E, Grandinetti A, Verheus M, Kolonel LN, Maskarinec G. Dietary Fiber, Magnesium, and Glycemic Load Alter Risk of Type 2 Diabetes in a Multiethnic Cohort in Hawaii12. J Nutr. 2010;140(1):68-74.

234. Horikawa C, Yoshimura Y, Kamada C, Tanaka S, Tanaka S, Hanyu O, et al., editors. Is Carbohydrate Intake Associated with the Incidence of Diabetes Complications? Japan Diabetes Complication Study (JDCS). Diabetes; 2016: AMER DIABETES ASSOC 1701 N BEAUREGARD ST, ALEXANDRIA, VA 22311-1717 USA.

235. Horikawa C, Otsuka R, Kato Y, Nishita Y, Tange C, Rogi T, et al. Longitudinal Association between n-3 Long-Chain Polyunsaturated Fatty Acid Intake and Depressive Symptoms: A Population-Based Cohort Study in Japan. Nutrients. 2018;10(11):1655.

236. Horn-Ross PL, Hoggatt KJ, West DW, Krone MR, Stewart SL, Anton-Culver H, et al. Recent diet and breast cancer risk: the California Teachers Study (USA). CCC. 2002;13(5):407-15.

237. Hou W, Han T, Sun X, Chen Y, Xu J, Wang Y, et al. Relationship Between Carbohydrate Intake (Quantity, Quality, and Time Eaten) and Mortality (Total, Cardiovascular, and Diabetes): Assessment of 2003–2014 National Health and Nutrition Examination Survey Participants. Diabetes Care. 2022;45(12):3024-31.

238. Houston DK, Tooze JA, Garcia K, Visser M, Rubin S, Harris TB, et al. Protein Intake and Mobility Limitation in Community-Dwelling Older Adults: the Health ABC Study. J Am Geriatr Soc. 2017;65(8):1705-11.

239. Howarth NC, Murphy SP, Wilkens LR, Henderson BE, Kolonel LN. The association of glycemic load and carbohydrate intake with colorectal cancer risk in the Multiethnic Cohort Study. Am J Clin Nutr. 2008;88(4):1074-82.

240. Hruby A, Guasch-Ferré M, Bhupathiraju SN, Manson JE, Willett WC, McKeown NM, Hu FB. Magnesium Intake, Quality of Carbohydrates, and Risk of Type 2 Diabetes: Results From Three U.S. Cohorts. Diabetes Care. 2017;40(12):1695-702.

241. Hu FB, Bronner L, Willett WC, Stampfer MJ, Rexrode KM, Albert CM, et al. Fish and omega-3 fatty acid intake and risk of coronary heart disease in women. Jama. 2002;287(14):1815-21.

242. HU T, Liu Y, He J, Bazzano L. Abstract P407: Low-Carbohydrate Dietary Pattern and Mortality in US Adults: The Third National Health and Nutritional Examination Survey (NHANES III). Circulation. 2013;127(suppl_12):AP407-AP.

243. Huang H-L, Abe SK, Sawada N, Takachi R, Ishihara J, Iwasaki M, et al. Dietary glycemic index, glycemic load and mortality: Japan Public Health Center-based prospective study. Eur J Nutr. 2021;60(8):4607-20.

244. Hunter DJ, Spiegelman D, Adami HO, Beeson L, van den Brandt PA, Folsom AR, et al. Cohort studies of fat intake and the risk of breast cancer--a pooled analysis. N Engl J Med. 1996;334(6):356-61.

245. Hunter DJ, Spiegelman D, Adami HO, van den Brandt PA, Folsom AR, Goldbohm RA, et al. Non-dietary factors as risk factors for breast cancer, and as effect modifiers of the association of fat intake and risk of breast cancer. CCC. 1997;8(1):49-56.

246. Iff S, Wong G, Webster AC, Flood V, Wang JJ, Mitchell P, Craig JC. Relative Energy Balance, CKD, and Risk of Cardiovascular and All-Cause Mortality. American Journal of Kidney Diseases. 2014;63(3):437-45.

247. Isaksen T, Evensen LH, Johnsen SH, Jacobsen BK, Hindberg K, Brækkan SK, Hansen JB. Dietary intake of marine n-3 polyunsaturated fatty acids and future risk of venous thromboembolism. RPTH. 2019;3(1):59-69.

248. Isaksen T, Evensen LH, Brækkan SK, Hansen J-B. Dietary Intake of Marine Polyunsaturated n-3 Fatty Acids and Risk of Recurrent Venous Thromboembolism. Thromb Haemost. 2019;119(12):2053-63.

249. Iso H, Sato S, Kitamura A, Naito Y, Shimamoto T, Komachi Y. Fat and protein intakes and risk of intraparenchymal hemorrhage among middle-aged Japanese. Am J Epidemiol. 2003;157(1):32-9.

250. Jain M, Miller AB, To T. Premorbid diet and the prognosis of women with breast cancer. J Natl Cancer Inst. 1994;86(18):1390-7.

251. Janket SJ, Manson JE, Sesso H, Buring JE, Liu S. A prospective study of sugar intake and risk of type 2 diabetes in women. Diabetes Care. 2003;26(4):1008-15.

252. Jantchou P, Morois S, Clavel-Chapelon F, Boutron-Ruault M-C, Carbonnel F. Animal Protein Intake and Risk of Inflammatory Bowel Disease: The E3N Prospective Study. ACG. 2010;105(10).

253. Jenkins DJA, Dehghan M, Mente A, Bangdiwala SI, Rangarajan S, Srichaikul K, et al. Glycemic Index, Glycemic Load, and Cardiovascular Disease and Mortality. NEJM. 2021;384(14):1312-22.

254. Ji X-W, Wang J, Shen Q-M, Li Z-Y, Jiang Y-F, Liu D-K, et al. Dietary fat intake and liver cancer incidence: A population-based cohort study in Chinese men. Int J Cancer. 2021;148(12):2982-96.

255. Ji X, Wang J, Li Z, Shen Q, Tuo J, Bi J, et al. Dietary fat intake and liver cancer risk: A prospective cohort study in Chinese women. Cancer Biol Med. 2021;19(3):370-83.

256. Jiao J, Zhuang P, Wang W, Wang J, editors. ASSOCIATION OF DIETARY INTAKE OF POLYUNSATURATED FATTY ACIDS AND MORTALITY: A SINO-AMERICAN NATIONAL JOINT STUDY OF CHNS AND NHANES. Ann Nutr Metab; 2017: KARGER ALLSCHWILERSTRASSE 10, CH-4009 BASEL, SWITZERLAND.

257. Jiao L, Flood A, Subar AF, Hollenbeck AR, Schatzkin A, Stolzenberg-Solomon R. Glycemic Index, Carbohydrates, Glycemic Load, and the Risk of Pancreatic Cancer in a Prospective Cohort Study. CEBP. 2009;18(4):1144-51.

258. Jo U, Park K. Carbohydrate-based diet may increase the risk of cardiovascular disease: A pooled analysis of two prospective cohort studies. Clin Nutr. 2023;42(8):1301-7.

259. Joensen AM, Schmidt EB, Dethlefsen C, Johnsen SP, Overvad K, Rasmussen LH, Tjønneland A. Dietary intake of total marine n-3 polyunsaturated fatty acids, eicosapentaenoic acid, docosahexaenoic acid and docosapentaenoic acid and the risk of acute coronary syndrome – a cohort study. Br J Nutr. 2010;103(4):602-7.

260. Johnston EA, Ibiebele TI, Friedlander ML, Grant PT, van der Pols JC, Webb PM. Association of Protein Intake with Recurrence and Survival Following Primary Treatment of Ovarian Cancer. The American Journal of Clinical Nutrition. 2023;118(1):50-8.

261. Joshipura KJ, Hung H-C, Li TY, Colditz G, Hu FB, Rimm EB, et al. Intakes of fruits, vegetables and carbohydrate and the risk of CVD. Public Health Nutr. 2009;12(1):115-21.

262. Kabat GC, Shikany JM, Beresford SAA, Caan B, Neuhouser ML, Tinker LF, Rohan TE. Dietary carbohydrate, glycemic index, and glycemic load in relation to colorectal cancer risk in the Women’s Health Initiative. CCC. 2008;19(10):1291-8.

263. Kaiser A, Schaefer SM, Behrendt I, Eichner G, Fasshauer M. Association of sugar intake from different sources with incident depression in the prospective cohort of UK Biobank participants. Eur J Nutr. 2023;62(2):727-38.

264. Kaiser A, Schaefer SM, Behrendt I, Eichner G, Fasshauer M. Association of all-cause mortality with sugar intake from different sources in the prospective cohort of UK Biobank participants. Br J Nutr. 2023;130(2):294-303.

265. Kanehara R, Katagiri R, Goto A, Yamaji T, Sawada N, Iwasaki M, et al. Sugar intake and colorectal cancer risk: A prospective Japanese cohort study. Cancer Sci. 2023;114(6):2584-95.

266. Kang M, Park S-Y, Boushey CJ, Wilkens LR, Le Marchand L, Hankin JH, Paik H-Y. Does Incorporating Gender Differences into Quantifying a Food Frequency Questionnaire Influence the Association of Total Energy Intake with All-Cause and Cause-Specific Mortality? Nutrients. 2020;12(10):2914.

267. Kantor ED, Lampe JW, Peters U, Vaughan TL, White E. Long-Chain Omega-3 Polyunsaturated Fatty Acid Intake and Risk of Colorectal Cancer. Nutr Cancer. 2014;66(4):716-27.

268. Kapoor K, Alfaddagh A, Rifai MA, Bhatt DL, Budoff MJ, Nasir K, et al. Association Between Omega-3 Fatty Acid Levels and Risk for Incident Major Bleeding Events and Atrial Fibrillation: MESA. JAHA. 2021;10(11):e021431.

269. Kato A, Okada C, Eshak ES, Iso H, Tamakoshi A, Group tJS. Association between dietary intake of n-3 polyunsaturated fatty acids and risk of colorectal cancer in the Japanese population: The Japan Collaborative Cohort Study. Cancer Med. 2023;12(4):4690-700.

270. Kaushik S, Wang JJ, Wong TY, Flood V, Barclay A, Brand-Miller J, Mitchell P. Glycemic Index, Retinal Vascular Caliber, and Stroke Mortality. Stroke. 2009;40(1):206-12.

271. Key TJ, Appleby PN, Cairns BJ, Luben R, Dahm CC, Akbaraly T, et al. Dietary fat and breast cancer: comparison of results from food diaries and food-frequency questionnaires in the UK Dietary Cohort Consortium. The American Journal of Clinical Nutrition. 2011;94(4):1043-52.

272. Key TJ, Balkwill A, Bradbury KE, Reeves GK, Kuan AS, Simpson RF, et al. Foods, macronutrients and breast cancer risk in postmenopausal women: a large UK cohort. Int J Epidemiol. 2018;48(2):489-500.

273. Khankari NK, Bradshaw PT, Steck SE, He K, Olshan AF, Shen J, et al. Dietary intake of fish, polyunsaturated fatty acids, and survival after breast cancer: A population-based follow-up study on Long Island, New York. Cancer. 2015;121(13):2244-52.

274. Kiage JN, Merrill PD, Robinson CJ, Cao Y, Malik TA, Hundley BC, et al. Intake of trans fat and all-cause mortality in the Reasons for Geographical and Racial Differences in Stroke (REGARDS) cohort. The American Journal of Clinical Nutrition. 2013;97(5):1121-8.

275. Kiage JN, Sampson UKA, Lipworth L, Fazio S, Mensah GA, Yu Q, et al. Intake of polyunsaturated fat in relation to mortality among statin users and non-users in the Southern Community Cohort Study. Nutr Metab Cardiovasc Dis. 2015;25(11):1016-24.

276. Kim DJ, Gallagher RP, Hislop TG, Holowaty EJ, Howe GR, Jain M, et al. Premorbid diet in relation to survival from prostate cancer (Canada). CCC. 2000;11:65-77.

277. Kim H, Lee H, Kwon SH, Jeon JS, Noh H, Han DC, Kim H. Relationship between carbohydrate-to-fat intake ratio and the development of chronic kidney disease: A community-based prospective cohort study. Clin Nutr. 2021;40(10):5346-54.

278. Kim JH, Lee J, Jung S-Y, Kim J. Dietary Factors and Female Breast Cancer Risk: A Prospective Cohort Study. Nutrients. 2017;9(12):1331.

279. Kippler M, Vargas CD, Berglund M, Glynn A, Wolk A, Åkesson A. Abstract P104: Dietary Polychlorinated biphenyls and Long-chain Omega-3 Fish fatty acids Exposures and Risk of Heart Failure. Circulation. 2018;137(suppl_1):AP104-AP.

280. Kiran N, Prizment AE, Lazovich D, Mao Z, Bostick RM. Sucrose Intakes and Incident Colorectal Cancer Risk among Women. JANA. 2022;41(1):57-63.

281. Kiyabu GY, Inoue M, Saito E, Abe SK, Sawada N, Ishihara J, et al. Fish, n − 3 polyunsaturated fatty acids and n − 6 polyunsaturated fatty acids intake and breast cancer risk: The Japan Public Health Center-based prospective study. Int J Cancer. 2015;137(12):2915-26.

282. Knüppel A, Shipley M, Llewellyn C, Brunner E. OP47 Relationships between sugar intake from sweet food and beverages, common mental disorder and depression: prospective findings from the whitehall ii cohort study. JECH. 2017;71(Suppl 1):A24-A.

283. Ananthakrishnan AN, Khalili H, Konijeti GG, Higuchi LM, de-Silva P, Fuchs CS, et al. Long-term intake of dietary fat and risk of ulcerative colitis and Crohn's disease. Gut. 2014;63(5):776-84.

284. Bjørnevik K, Chitnis T, Ascherio A, Munger KL. Polyunsaturated fatty acids and the risk of multiple sclerosis. Mult Scler J. 2015;23(11):53-4.

285. Brouwer IA, Heeringa J, Geleijnse JM, Zock PL, Witteman JC. Intake of very long-chain n-3 fatty acids from fish and incidence of atrial fibrillation. The Rotterdam Study. Am Heart J. 2006;151(4):857-62.

286. Drake I, Sonestedt E, Gullberg B, Ahlgren G, Bjartell A, Wallström P, Wirfält E. Dietary intakes of carbohydrates in relation to prostate cancer risk: a prospective study in the Malmö Diet and Cancer cohort. The American Journal of Clinical Nutrition. 2012;96(6):1409-18.

287. Estrada-deLeon DB, Struijk EA, Caballero FF, Yevenes-Briones H, Banegas JR, Rodriguez-Artalejo F, Lopez-Garcia E. Distribution of daily protein intake across meals and all-cause mortality in community-dwelling older adults. Br J Nutr. 2022:1-7.

288. Ferraro PM, Mandel EI, Curhan GC, Gambaro G, Taylor EN. Diet-Dependent Net Acid Load, Protein Intake, and Risk of Incident Kidney Stones. JASN. 2015;26:79A.

289. Fung T, Willett W, Feskanich D. Protein intake and risk of hip fractures in post menopausal women and men over 50 years. The FASEB Journal. 2016;30(S1):290.5-.5.

290. García-Gavilán JF, Bulló M, Camacho-Barcia L, Rosique-Esteban N, Hernández-Alonso P, Basora J, et al. Higher dietary glycemic index and glycemic load values increase the risk of osteoporotic fracture in the PREvención con DIeta MEDiterránea (PREDIMED)-Reus trial. The American Journal of Clinical Nutrition. 2018;107(6):1035-42.

291. Ghorbani Z, Hekmatdoost A, Poustchi H, Pourshams A, Malekshah AF, Sharafkhah M, Malekzadeh R. The association between dietary carbohydrate intake and pancreatic cancer risk: A large prospective study. Govaresh. 2016;21(3):167-74.

292. He K, Xun P, Brasky TM, Gammon MD, Stevens J, White E. Types of Fish Consumed and Fish Preparation Methods in Relation to Pancreatic Cancer Incidence: The VITAL Cohort Study. American Journal of Epidemiology. 2012;177(2):152-60.

293. Kim YS, Xun P, Iribarren C, Van Horn L, Steffen L, Daviglus ML, et al. Intake of fish and long-chain omega-3 polyunsaturated fatty acids and incidence of metabolic syndrome among American young adults: a 25-year follow-up study. Eur J Nutr. 2016;55(4):1707-16.

294. Banim PJR, Luben R, Sharp SJ, Wareham NJ, Khaw KT, Hart AR. The role of dietary cholesterol and transfatty acids in the aetiology of gallstones: A UK prospective cohort study (epic-norfolk) using 7-day food diaries. Gut. 2011;60:A225.

295. Knuppel A, Shipley MJ, Llewellyn C, Brunner EJ, editors. ROLE OF SUGAR INTAKE IN THE PREVENTION OF DEPRESSION: PROSPECTIVE FINDINGS FROM THE WHITEHALL II COHORT STUDY. Ann Nutr Metab; 2017: KARGER ALLSCHWILERSTRASSE 10, CH-4009 BASEL, SWITZERLAND.

296. Koh W-P, Dan YY, Pan A, Yuan J-M. Abstract 1264: Dietary n-6 fatty acids and risk of hepatocellular carcinoma among Chinese in Singapore: The Singapore Chinese Health Study. Cancer Res. 2014;74(19_Supplement):1264-.

297. Koh W-P, Jin A, editors. FATTY ACID INTAKE IN RELATION TO RISK OF HIP FRACTURE IN THE SINGAPORE CHINESE HEALTH STUDY. Osteoporos Int; 2016: SPRINGER LONDON LTD 236 GRAYS INN RD, 6TH FLOOR, LONDON WC1X 8HL, ENGLAND.

298. Kokts-Porietis RL, Morielli AR, McNeil J, Courneya KS, Cook LS, Friedenreich CM. Prospective Cohort of Pre- and Post-Diagnosis Diet with Survival Outcomes: an Alberta Endometrial Cancer Cohort Study. CEBP. 2023;32(2):242-51.

299. Korat AA, Hu FB, Sun Q. Abstract P234: Dairy Fat Intake and Risk of Type 2 Diabetes in 3 Cohorts of US Adults. Circulation. 2018;137(suppl_1):AP234-AP.

300. Kraja B, Muka T, Ruiter R, Hofman A, Stricker B, Franco O, Kiefte-de Jong J. O-0013 - Dietary Fatty Acids Intake and Colorectal Cancer Risk: The Rotterdam Study. Ann Oncol. 2014;25:ii110.

301. Kraja B, Muka T, Ruiter R, de Keyser CE, Hofman A, Franco OH, et al. Dietary Fiber Intake Modifies the Positive Association between n–3 PUFA Intake and Colorectal Cancer Risk in a Caucasian Population. J Nutr. 2015;145(8):1709-16.

302. Krishnan S, Paton CM, Steffen LM, Cooper JA. Impact of dietary fat composition on prediabetes: a 12-year follow-up study. Public Health Nutr. 2017;20(9):1617-26.

303. Krishnan S, Rosenberg L, Singer M, Hu FB, Djoussé L, Cupples LA, Palmer JR. Glycemic index, glycemic load, and cereal fiber intake and risk of type 2 diabetes in US black women. Arch Intern Med. 2007;167(21):2304-9.

304. Krok-Schoen JL, Brasky TM, Hunt RP, Rohan TE, Baker TA, Li W, et al. Dietary Long-Chain n-3 Fatty Acid Intake and Arthritis Risk in the Women's Health Initiative. J Acad Nutr Diet. 2018;118(11):2057-69.

305. Kubo S, Imano H, Muraki I, Kitamura A, Noda H, Cui R, et al. Total protein intake and subsequent risk of chronic kidney disease: the Circulatory Risk in Communities Study. EHPM. 2023;28:32-.

306. Kurihara A, Okamura T, Sugiyama D, Higashiyama A, Watanabe M, Okuda N, et al. Vegetable Protein Intake was Inversely Associated with Cardiovascular Mortality in a 15-Year Follow-Up Study of the General Japanese Population. J Atheroscler Thromb. 2019;26(2):198-206.

307. Kushi LH, Sellers TA, Potter JD, Nelson CL, Munger RG, Kaye SA, Folsom AR. Dietary fat and postmenopausal breast cancer. J Natl Cancer Inst. 1992;84(14):1092-9.

308. Kwon Y-J, Lee HS, Park J-Y, Lee J-W. Associating Intake Proportion of Carbohydrate, Fat, and Protein with All-Cause Mortality in Korean Adults. Nutrients. 2020;12(10):3208.

309. Kwon Y-J, Park K, Lee J-H. Low-protein diet is inversely related to the incidence of chronic kidney disease in middle-aged and older adults: results from a community-based prospective cohort study. Eur J Nutr. 2022;61(7):3795-807.

310. Laake I, Carlsen MH, Pedersen JI, Weiderpass E, Selmer R, Kirkhus B, et al. Intake of trans fatty acids from partially hydrogenated vegetable and fish oils and ruminant fat in relation to cancer risk. Int J Cancer. 2013;132(6):1389-403.

311. Laaksonen DE, Laukkanen JA, Niskanen L, Nyyssönen K, Rissanen TH, Voutilainen S, et al. Serum linoleic and total polyunsaturated fatty acids in relation to prostate and other cancers: a population-based cohort study. Int J Cancer. 2004;111(3):444-50.

312. Laaksonen DE, Nyyssönen K, Niskanen L, Rissanen TH, Salonen JT. Prediction of cardiovascular mortality in middle-aged men by dietary and serum linoleic and polyunsaturated fatty acids. Arch Intern Med. 2005;165(2):193-9.

313. Lagiou P, Sandin S, Weiderpass E, Lagiou A, Mucci L, Trichopoulos D, Adami HO. Low carbohydrate-high protein diet and mortality in a cohort of Swedish women. J Intern Med. 2007;261(4):366-74.

314. Laguzzi F, Carlsson A, Gigante B, Hellenius M, De F, Leander K, editors. Predictive role of dietary habits in relation to incident CVD and all-cause mortality: results from a Swedish cohort of 60-year-old. Healthy living: the European Congress of Epidemiology; 2015.

315. Lahiri M, Morgan C, Luben RN, Lentjes M, Bunn DK, Lunt M, et al. FRI0067 The relationship between fructose intake, alcohol consumption and serum URIC acid levels and the risk of inflammatory polyarthritis. Results from the european prospective investigation of cancer and the norfolk arthritis register: The EPIC-2-noar study. Ann Rheum Dis. 2013;71(Suppl 3):332-.

316. Lajous M, Boutron-Ruault M-C, Fabre A, Clavel-Chapelon F, Romieu I. Carbohydrate intake, glycemic index, glycemic load, and risk of postmenopausal breast cancer in a prospective study of French women1. The American Journal of Clinical Nutrition. 2008;87(5):1384-91.

317. Lampuré A, Castetbon K, Deglaire A, Schlich P, Péneau S, Hercberg S, Méjean C. Associations between liking for fat, sweet or salt and obesity risk in French adults: a prospective cohort study. IJBNPA. 2016;13(1):74.

318. Lander EM, Wertheim BC, Koch SM, Chen Z, Hsu C-H, Thomson CA. Vegetable protein intake is associated with lower gallbladder disease risk: Findings from the Women's Health Initiative prospective cohort. Prev Med. 2016;88:20-6.

319. Langsetmo L, Shikany JM, Cawthon PM, Cauley JA, Taylor BC, Vo TN, et al. Low protein intake among older men is associated with an increased risk of fracture. JBMR. 2017;31.

320. Langsetmo L, Shikany JM, Cawthon PM, Cauley JA, Taylor BC, Vo TN, et al. The Association Between Protein Intake by Source and Osteoporotic Fracture in Older Men: A Prospective Cohort Study. JBMR. 2016;32(3):592-600.

321. Larsson SC, Bergkvist L, Wolk A. Consumption of sugar and sugar-sweetened foods and the risk of pancreatic cancer in a prospective study. Am J Clin Nutr. 2006;84(5):1171-6.

322. Larsson SC, Bergkvist L, Wolk A. Conjugated linoleic acid intake and breast cancer risk in a prospective cohort of Swedish women12. The American Journal of Clinical Nutrition. 2009;90(3):556-60.

323. Larsson SC, Giovannucci EL, Wolk A. Prospective Study of Glycemic Load, Glycemic Index, and Carbohydrate Intake in Relation to Risk of Biliary Tract Cancer. ACG. 2016;111(6).

324. Larsson SC, Wolk A. Fish, long-chain omega-3 polyunsaturated fatty acid intake and incidence of atrial fibrillation: A pooled analysis of two prospective studies. Clin Nutr. 2017;36(2):537-41.

325. Lasota AN, Grønholdt M-LM, Bork CS, Lundbye-Christensen S, Overvad K, Schmidt EB. Marine n-3 Fatty Acids and the Risk of Peripheral Arterial Disease. JACC. 2018;72(14):1576-84.

326. Lee C-L, Liu W-J, Wang J-S. Effect of low-protein intake on all-cause mortality in subjects with an estimated glomerular filtration rate higher than 60 mL/min/1.73 m2 with or without albuminuria. Int J Clin Pract. 2020;74(7):e13505.

327. Lee C-L, Liu W-J, Wang J-S. Associations of low-carbohydrate and low-fat intakes with all-cause mortality in subjects with prediabetes with and without insulin resistance. Clin Nutr. 2021;40(5):3601-7.

328. Lee J-H, Park H-M, Lee Y-J. Using Dietary Macronutrient Patterns to Predict Sarcopenic Obesity in Older Adults: A Representative Korean Nationwide Population-Based Study. Nutrients. 2021;13(11):4031.

329. Lee KW, Lyu J, Park JK, Jo C, Kim SS. Dietary carbohydrate quality and quantity in relation to the incidence of type 2 diabetes: A prospective cohort study of middle-aged and older Korean adults. Nutrition. 2019;57:245-51.

330. Lelli D, Antonelli Incalzi R, Ferrucci L, Bandinelli S, Pedone C. Association between PUFA intake and serum concentration and mortality in older adults: A cohort study. Clin Nutr. 2020;39(2):510-5.

331. Lelong H, Blacher J, Baudry J, Adriouch S, Galan P, Fezeu L, et al. Individual and Combined Effects of Dietary Factors on Risk of Incident Hypertension. Hypertension. 2017;70(4):712-20.

332. Lentjes MAH, Keogh RH, Luben RN, Mulligan AA, Welch AA, Khaw KT. Total (food and supplement) n-3 PUFA intake is associated with lower Coronary Heart Disease mortality, independently of fish intake. Proc Nutr Soc. 2016;75(OCE1):E42.

333. Lentjes MAH, Keogh RH, Welch AA, Mulligan AA, Luben RN, Wareham NJ, Khaw K-T. Longitudinal associations between marine omega-3 supplement users and coronary heart disease in a UK population-based cohort. BMJ Open. 2017;7(10):e017471.

334. Leosdottir M, Nilsson PM, Nilsson J-Å, Berglund G. Cardiovascular event risk in relation to dietary fat intake in middle-aged individuals: data from The Malmö Diet and Cancer Study. Eur J Cardiovasc Prev Rehabil. 2007;14(5):701-6.

335. LEOSDOTTIR M, NILSSON PM, NILSSON J-Å, MÅNSSON H, BERGLUND G. Dietary fat intake and early mortality patterns – data from The Malmö Diet and Cancer Study. J Intern Med. 2005;258(2):153-65.

336. Levitan EB, Mittleman MA, Wolk A. Dietary glycemic index, dietary glycemic load and mortality among men with established cardiovascular disease. Eur J Clin Nutr. 2009;63(4):552-7.

337. Levitan EB, Mittleman MA, Wolk A. Dietary glycaemic index, dietary glycaemic load and incidence of myocardial infarction in women. Br J Nutr. 2010;103(7):1049-55.

338. Levitan EB, Wolk A, Håkansson N, Mittleman MA. α-Linolenic acid, linoleic acid and heart failure in women. Br J Nutr. 2012;108(7):1300-6.

339. Levitan EB, Mittleman MA, Håkansson N, Wolk A. Dietary glycemic index, dietary glycemic load, and cardiovascular disease in middle-aged and older Swedish men. Am J Clin Nutr. 2007;85(6):1521-6.

340. Levitan EB, Mittleman MA, Wolk A. Dietary glycemic index, dietary glycemic load, and incidence of heart failure events: a prospective study of middle-aged and elderly women. J Am Coll Nutr. 2010;29(1):65-71.

341. Li Q, Liu C, Zhang S, Li R, Zhang Y, He P, et al. Dietary Carbohydrate Intake and New-Onset Hypertension: A Nationwide Cohort Study in China. Hypertension. 2021;78(2):422-30.

342. Li S, Hu FB, Forman JP, Rimm EB. Abstract P064: Dietary Glycemic Index and Glycemic Load and Risk of Coronary Heart Disease in a Prospective Study Among US Male Health Professionals. Circulation. 2012;125(suppl_10):AP064-AP.

343. Li S, Flint A, Pai JK, Forman JP, Hu FB, Willett WC, et al. Low Carbohydrate Diet From Plant or Animal Sources and Mortality Among Myocardial Infarction Survivors. JAHA. 2014;3(5):e001169.

344. Limburg PJ, Liu-Mares W, Vierkant RA, Wang AH, Harnack L, Flood AP, et al. Prospective evaluation of trans-fatty acid intake and colorectal cancer risk in the Iowa Women's Health Study. Int J Cancer. 2008;123(11):2717-9.

345. Lin C-C, Liu C-S, Li C-I, Lin C-H, Lin W-Y, Wang M-C, et al. Dietary Macronutrient Intakes and Mortality among Patients with Type 2 Diabetes. Nutrients. 2020;12(6):1665.

346. Lin J, Zhang SM, Cook NR, Lee IM, Buring JE. Dietary fat and fatty acids and risk of colorectal cancer in women. Am J Epidemiol. 2004;160(10):1011-22.

347. Liss MA, Al-Bayati O, Gelfond J, Goros M, Ullevig S, DiGiovanni J, et al. Higher baseline dietary fat and fatty acid intake is associated with increased risk of incident prostate cancer in the SABOR study. Prostate Cancer Prostatic Dis. 2019;22(2):244-51.

348. Liu L, Volpe SL, Ross JA, Grimm JA, Van Bockstaele EJ, Eisen HJ. Dietary sugar intake and risk of Alzheimer's disease in older women. Nutr Neurosci. 2022;25(11):2302-13.

349. Liu Y, Yang W, VoPham T, Ma Y, Simon TG, Gao X, et al. Plant-Based and Animal-Based Low-Carbohydrate Diets and Risk of Hepatocellular Carcinoma Among US Men and Women. Hepatology. 2021;73(1):175-85.

350. Liu Z. Association of dietary sugar intake with bone loss, non-traumatic fracture risk, and all-cause mortality in Chinese elderly: finding from an 11-year longitudinal study of Mr and Ms OS Hong Kong. The FASEB Journal. 2017;31:lb450-lb.

351. Liu Zm, Tse LA, Chan D, Wong C, Wong SYS. Dietary sugar intake was associated with increased body fatness but decreased cardiovascular mortality in Chinese elderly: an 11-year prospective study of Mr and Ms OS of Hong Kong. Int J Obes. 2018;42(4):808-16.

352. Luchsinger JA, Tang M-X, Shea S, Mayeux R. Caloric intake and the risk of Alzheimer disease. Arch Neurol. 2002;59(8):1258-63.

353. Luu HN, Cai H, Murff HJ, Xiang Y-B, Cai Q, Li H, et al. A prospective study of dietary polyunsaturated fatty acids intake and lung cancer risk. Int J Cancer. 2018;143(9):2225-37.

354. Luu HN, Murff HJ, Li H, Cai Q, Gao Y-T, Gao J, et al. Abstract 5310: A prospective study of dietary polyunsaturated fatty acid intakes and lung cancer risk. Cancer Res. 2017;77(13_Supplement):5310-.

355. Lv D, Wang R, Chen M, Li Y, Cao C. Fish Intake, Dietary Polyunsaturated Fatty Acids, and Lung Cancer: Systematic Review and Dose–Response Meta-Analysis of 1.7 Million Men and Women. Nutr Cancer. 2022;74(6):1976-85.

356. Ma W, Wu JH, Wang Q, Lemaitre RN, Mukamal KJ, Djousse L, et al. Abstract 12: Prospective Associations of Fatty Acids in the De Novo Lipogenesis Pathway and Stearoyl CoA-desaturase-1 Activity with Risk of Type 2 Diabetes: the Cardiovascular Health Study. Circulation. 2014;129(suppl_1):A12-A.

357. Makarem N, Bandera EV, Lin Y, Jacques PF, Hayes RB, Parekh N. Consumption of Sugars, Sugary Foods, and Sugary Beverages in Relation to Adiposity-Related Cancer Risk in the Framingham Offspring Cohort (1991–2013). Cancer Prev Res. 2018;11(6):347-58.

358. Makarem N, Lin Y, Bandera EV, Jacques P, Parekh N. Dietary Carbohydrate Intake, Glycemic Index and Glycemic Load in Relation to Adiposity-Related Cancer Risk: Results from The Framingham Offspring Cohort (1991–2013). The FASEB Journal. 2016;30(S1):417.7-.7.

359. Makarem N, Bandera EV, Hayes RB, Jacques PF, Lin Y, Parekh N. Carbohydrate nutrition and risk of adiposity-related cancers: results from the Framingham Offspring cohort (1991–2013). Br J Nutr. 2017;117(11):1603-14.

360. Malhotra R, Kabagambe EK, Cavanaugh KL, Blot WJ, Ikizler TA, Lipworth L. High Protein Intake in Relation to Incident End-Stage Renal Disease (ESRD) Among Blacks and Whites in the Southern Community Cohort Study (SCCS). JASN. 2015;26:572A.

361. Mandalazi E, Drake I, Wirfält E, Orho-Melander M, Sonestedt E. A High Diet Quality Based on Dietary Recommendations Is Not Associated with Lower Incidence of Type 2 Diabetes in the Malmö Diet and Cancer Cohort. Int J Mol Sci. 2016;17(6):901.

362. Manger MS, Strand E, Ebbing M, Seifert R, Refsum H, Nordrehaug JE, et al. Dietary intake of n–3 long-chain polyunsaturated fatty acids and coronary events in Norwegian patients with coronary artery disease. The American Journal of Clinical Nutrition. 2010;92(1):244-51.

363. Masala G, Assedi M, Bendinelli B, Ermini I, Occhini D, Sieri S, et al. Glycemic Index, Glycemic Load and Mammographic Breast Density: The EPIC Florence Longitudinal Study. PLOS ONE. 2013;8(8):e70943.

364. Matsumoto C, Yoruk A, Wang L, Gaziano JM, Sesso HD. Fish and omega-3 fatty acid consumption and risk of hypertension. J Hypertens. 2019;37(6):1223-9.

365. Mattisson I, Wirfält E, Johansson U, Gullberg B, Olsson H, Berglund G. Intakes of plant foods, fibre and fat and risk of breast cancer – a prospective study in the Malmö Diet and Cancer cohort. Br J Cancer. 2004;90(1):122-7.

366. Mattisson I, Wirfält E, Wallström P, Gullberg B, Olsson H, Berglund G. High fat and alcohol intakes are risk factors of postmenopausal breast cancer: a prospective study from the Malmö diet and cancer cohort. Int J Cancer. 2004;110(4):589-97.

367. Mazidi M, Katsiki N, Mikhailidis DP, Banach M, Panel ILE. P5409Low-carbohydrate diets and all-cause and cause-specific mortality: a population-based cohort study and pooling prospective studies. Eur Heart J. 2018;39(suppl_1).

368. Mazidi M, Mikhailidis DP, Sattar N, Toth PP, Judd S, Blaha MJ, et al. Association of types of dietary fats and all-cause and cause-specific mortality: a prospective cohort study and meta-analysis of prospective studies with 1,148,117 participants. Eur Heart J. 2019;40(Supplement_1).

369. Mazidi M, Katsiki N, Mikhailidis DP, Sattar N, Banach M, Panel obotILE, et al. Lower carbohydrate diets and all-cause and cause-specific mortality: a population-based cohort study and pooling of prospective studies. Eur Heart J. 2019;40(34):2870-9.

370. Mazidi M, Mikhailidis DP, Sattar N, Toth PP, Judd S, Blaha MJ, et al. Association of types of dietary fats and all-cause and cause-specific mortality: A prospective cohort study and meta-analysis of prospective studies with 1,164,029 participants. Clin Nutr. 2020;39(12):3677-86.

371. McEligot AJ, Largent J, Ziogas A, Peel D, Anton-Culver H. Dietary fat, fiber, vegetable, and micronutrients are associated with overall survival in postmenopausal women diagnosed with breast cancer. Nutr Cancer. 2006;55(2):132-40.

372. Meinhold CL, Dodd KW, Jiao L, Flood A, Shikany JM, Genkinger JM, et al. Available Carbohydrates, Glycemic Load, and Pancreatic Cancer: Is There a Link? American Journal of Epidemiology. 2010;171(11):1174-82.

373. Mekary RA, Rimm EB, Giovannucci E, Stampfer MJ, Willett WC, Ludwig DS, Hu FB. Joint association of glycemic load and alcohol intake with type 2 diabetes incidence in women. The American Journal of Clinical Nutrition. 2011;94(6):1525-32.

374. Mendonça NMP, Hengeveld LM, Presse N, Canhão H, Simonsick E, Kritchevsky SB, et al. Protein intake, physical activity and grip strength in European and North American community-dwelling older adults: a pooled analysis of individual participant data from four longitudinal ageing cohorts. Br J Nutr. 2023;129(7):1221-31.

375. Mendonça N, Hengeveld LM, Visser M, Presse N, Canhão H, Simonsick EM, et al. Low protein intake, physical activity, and physical function in European and North American community-dwelling older adults: a pooled analysis of four longitudinal aging cohorts. The American Journal of Clinical Nutrition. 2021;114(1):29-41.

376. Merritt MA, Tzoulaki I, van den Brandt PA, Schouten LJ, Tsilidis KK, Weiderpass E, et al. Nutrient-wide association study of 57 foods/nutrients and epithelial ovarian cancer in the European Prospective Investigation into Cancer and Nutrition study and the Netherlands Cohort Study. The American Journal of Clinical Nutrition. 2016;103(1):161-7.

377. Merritt MA, Riboli E, Weiderpass E, Tsilidis KK, Overvad K, Tjønneland A, et al. Dietary fat intake and risk of epithelial ovarian cancer in the European Prospective Investigation into Cancer and Nutrition. Cancer Epidemiol. 2014;38(5):528-37.

378. Meyer DS, Millen AE, Nie J, Trevisan M, Freudenheim JL. Dietary Fats and All-cause and Breast Cancer–specific Mortality among Women with Breast Cancer: The Western New York Exposures and Breast Cancer Study. CEBP. 2023;32(6):854-6.

379. Meyer F, Bairati I, Shadmani R, Fradet Y, Moore L. Dietary fat and prostate cancer survival. CCC. 1999;10:245-51.

380. Meyer MS, Kasperzyk JL, Andrén O, Wolk A, Håkansson N, Andersson S-O, et al. Abstract 5747: Anti-inflammatory nutrients and prostate cancer survival in the Örebro Prostate Cancer Survivors Cohort. Cancer Res. 2010;70(8_Supplement):5747-.

381. Meyerhardt JA, Sato K, Niedzwiecki D, Ye C, Saltz LB, Mayer RJ, et al. Dietary Glycemic Load and Cancer Recurrence and Survival in Patients with Stage III Colon Cancer: Findings From CALGB 89803. JNCI. 2012;104(22):1702-11.

382. Michaud DS, Fuchs CS, Liu S, Willett WC, Colditz GA, Giovannucci E. Dietary glycemic load, carbohydrate, sugar, and colorectal cancer risk in men and women. CEBP. 2005;14(1):138-47.

383. Michaud DS, Giovannucci E, Willett WC, Colditz GA, Fuchs CS. Dietary meat, dairy products, fat, and cholesterol and pancreatic cancer risk in a prospective study. Am J Epidemiol. 2003;157(12):1115-25.

384. Michaud DS, Liu S, Giovannucci E, Willett WC, Colditz GA, Fuchs CS. Dietary sugar, glycemic load, and pancreatic cancer risk in a prospective study. J Natl Cancer Inst. 2002;94(17):1293-300.

385. Miles FL, Neuhouser ML, Zhang Z-F. Concentrated sugars and incidence of prostate cancer in a prospective cohort. Br J Nutr. 2018;120(6):703-10.

386. Mills PK, Beeson WL, Phillips RL, Fraser GE. Dietary habits and breast cancer incidence among Seventh-day Adventists. Cancer. 1989;64(3):582-90.

387. Mirmiran P, Bahadoran Z, Ghasemi A, Azizi F. Contribution of dietary amino acids composition to incidence of cardiovascular outcomes: A prospective population-based study. Nutr Metab Cardiovasc Dis. 2017;27(7):633-41.

388. Mirmiran P, Esfandyari S, Moghadam SK, Bahadoran Z, Azizi F. Fatty acid quality and quantity of diet and risk of type 2 diabetes in adults: Tehran Lipid and Glucose Study. JDC. 2018;32(7):655-9.

389. Mirmiran P, Houshialsadat Z, Bahadoran Z, Khalili-Moghadam S, Sheikholeslami F, Azizi F. Association of dietary fatty acids and the incidence risk of cardiovascular disease in adults: the Tehran Lipid and Glucose Prospective Study. BMC Public Health. 2020;20(1):1743.

390. Miyagawa N, Miura K, Okuda N, Kadowaki T, Takashima N, Nagasawa SY, et al. Long-chain n-3 polyunsaturated fatty acids intake and cardiovascular disease mortality risk in Japanese: a 24-year follow-up of NIPPON DATA80. Atherosclerosis. 2014;232(2):384-9.

391. Miyagawa N, Miura K, Okuda N, Kadowaki T, Takashima N, Nagasawa S-y, et al. Abstract WP205: Long-chain n-3 Polyunsaturated Fatty Acids Intake and Cardiovascular Disease Mortality Risk in a General Japanese Population: NIPPON DATA80. Stroke. 2013;44(suppl_1):AWP205-AWP.

392. Miyazawa I, Miura K, Miyagawa N, Kondo K, Kadota A, Okuda N, et al. Abstract P221: Relationship of Dietary Carbohydrate and Fiber Intake to Risk of Cardiovascular Disease Mortality in Japanese: NIPPON DATA80. Circulation. 2017;135(suppl_1):AP221-AP.

393. Miyazawa I, Miura K, Miyagawa N, Kondo K, Kadota A, Okuda N, et al. Relationship between carbohydrate and dietary fibre intake and the risk of cardiovascular disease mortality in Japanese: 24-year follow-up of NIPPON DATA80. Eur J Clin Nutr. 2020;74(1):67-76.

394. Mongiovi JM, Freudenheim JL, Moysich KB, McCann SE. Glycemic Index, Glycemic Load, and Risk of Ovarian Cancer in the Prostate, Lung, Colorectal and Ovarian (PLCO) Cohort. J Nutr. 2021;151(6):1597-608.

395. Moore LL, Bradlee ML, Singer MR. Beneficial Effects of Animal and Plant Proteins on Skeletal Muscle Mass and Functional Status. The FASEB Journal. 2017;31(S1):443.4-.4.

396. Morimoto N, Urayama K, Tanaka A, Ai M. Relationship between dietary fibre to carbohydrate ratio and mortality risk in the NHANES. Int J Epidemiol. 2021;50(Supplement_1).

397. Morris MC, Manson JE, Rosner B, Buring JE, Willett WC, Hennekens CH. Fish consumption and cardiovascular disease in the physicians' health study: a prospective study. Am J Epidemiol. 1995;142(2):166-75.

398. Mozaffarian D, Ascherio A, Hu FB, Stampfer MJ, Willett WC, Siscovick DS, Rimm EB. Interplay between different polyunsaturated fatty acids and risk of coronary heart disease in men. Circulation. 2005;111(2):157-64.

399. Muka T, Kraja B, Ruiter R, Keyser CEd, Hofman A, Stricker BH, et al. Dietary polyunsaturated fatty acids intake modifies the positive association between serum total cholesterol and colorectal cancer risk: the Rotterdam Study. JECH. 2016;70(9):881-7.

400. Mursu J, Virtanen JK, Rissanen TH, Tuomainen TP, Nykänen I, Laukkanen JA, et al. Glycemic index, glycemic load, and the risk of acute myocardial infarction in Finnish men: The Kuopio Ischaemic Heart Disease Risk Factor Study. Nutr Metab Cardiovasc Dis. 2011;21(2):144-9.

401. Nagle CM, Purdie DM, Webb PM, Green A, Harvey PW, Bain CJ. Dietary influences on survival after ovarian cancer. Int J Cancer. 2003;106(2):264-9.

402. Nakamura Y, Kiyohara Y, Okuda N, Okamura T, Higashiyama A, Watanabe M, et al. Fatty acid intakes and coronary heart disease mortality in Japan: NIPPON DATA90, 1990-2005. Current Nutrition & Food Science. 2013;9(1):26-32.

403. Nakamura Y, Okuda N, Okamura T, Kadota A, Hayakawa T, Kita Y, et al. Low-carbohydrate diets and cardiovascular and total mortality in Japanese: a 29-year follow-up of NIPPON DATA80. Br J Nutr. 2014;112(6):916-24.

404. Nakano M, Uenishi K, Nakamura Y, Takahashi J, Shiraki M. Distinct dietary risk factors for incident osteoporotic fractures in early and late postmenopausal phase women. JBMM. 2023;41(2):227-38.

405. Narasaki Y, Okuda Y, Moore LW, You AS, Tantisattamo E, Inrig JK, et al. Dietary protein intake, kidney function, and survival in a nationally representative cohort. Am J Clin Nutr. 2021;114(1):303-13.

406. Narasaki Y, You AS, Miyagi T, Miyasato Y, Kimura H, Sy J, et al. Dietary fat intake and mortality across kidney function in a nationally representative cohort. JASN. 2020;31:621.

407. Navarro SL, Neuhouser ML, Cheng TD, Tinker LF, Shikany JM, Snetselaar L, et al. The Interaction between Dietary Fiber and Fat and Risk of Colorectal Cancer in the Women's Health Initiative. Nutrients. 2016;8(12).

408. Neuhouser ML, Barnett MJ, Kristal AR, Ambrosone CB, King I, Thornquist M, Goodman G. (n-6) PUFA increase and dairy foods decrease prostate cancer risk in heavy smokers. J Nutr. 2007;137(7):1821-7.

409. Nguyen S, Li H, Yu D, Cai H, Gao J, Gao Y, et al. Dietary fatty acids and colorectal cancer risk in men: A report from the Shanghai Men's Health Study and a meta-analysis. Int J Cancer. 2021;148(1):77-89.

410. Nielsen TG, Olsen A, Christensen J, Overvad K, Tjønneland A. Dietary carbohydrate intake is not associated with the breast cancer incidence rate ratio in postmenopausal Danish women. J Nutr. 2005;135(1):124-8.

411. Nilsson LM, Winkvist A, Eliasson M, Jansson JH, Hallmans G, Johansson I, et al. Low-carbohydrate, high-protein score and mortality in a northern Swedish population-based cohort. Eur J Clin Nutr. 2012;66(6):694-700.

412. Nilsson L, Winkvist A, Hallmans G, Johansson I, Lindahl B, Lenner P, Van Guelpen B. 3510 POSTER Low-carbohydrate, High-protein Score and Cancer Incidence and Mortality in a Northern Swedish Population. Eur J Cancer. 2011;47:S249.

413. Nilsson LM, Winkvist A, Johansson I, Lindahl B, Hallmans G, Lenner P, Van Guelpen B. Low-carbohydrate, high-protein diet score and risk of incident cancer; a prospective cohort study. Nutr J. 2013;12(1):58.

414. Nimptsch K, Kenfield SA, Stampfer MJ, Franz M, Sampson L, Brand-Miller JC, et al. Abstract 2823: Dietary glycemic index, glycemic load and insulin index, in relation to risk of prostate cancer. Cancer Res. 2010;70(8_Supplement):2823-.

415. Nimptsch K, Kenfield S, Jensen MK, Stampfer MJ, Franz M, Sampson L, et al. Dietary glycemic index, glycemic load, insulin index, fiber and whole-grain intake in relation to risk of prostate cancer. CCC. 2011;22(1):51-61.

416. Nöthlings U, Wilkens LR, Murphy SP, Hankin JH, Henderson BE, Kolonel LN. Meat and fat intake as risk factors for pancreatic cancer: the multiethnic cohort study. J Natl Cancer Inst. 2005;97(19):1458-65.

417. Oba S, Nagata C, Nakamura K, Fujii K, Kawachi T, Takatsuka N, Shimizu H. Dietary glycemic index, glycemic load, and intake of carbohydrate and rice in relation to risk of mortality from stroke and its subtypes in Japanese men and women. Metabolism. 2010;59(11):1574-82.

418. Odutola MK, van Leeuwen MT, Bassett JK, Bruinsma F, Turner J, Seymour JF, et al. Dietary intake of animal-based products and likelihood of follicular lymphoma and survival: A population-based family case-control study. Front Nutr. 2022;9:1048301.

419. Oh S-W, Wood AC, Hwang S-s, Allison M. Racial and Ethnic Differences in the Association of Low-Carbohydrate Diet With Mortality in the Multi-Ethnic Study of Atherosclerosis. JAMA Network Open. 2022;5(10):e2237552-e.

420. Oosterwijk MM, Groothof D, Navis G, Bakker SJL, Laverman GD. High-Normal Protein Intake Is Not Associated With Faster Renal Function Deterioration in Patients With Type 2 Diabetes: A Prospective Analysis in the DIALECT Cohort. Diabetes Care. 2021;45(1):35-41.

421. Otto M, Jacobs DR, Mozaffarian D, Kromhout D, Bertoni AG, Sibley CT, Nettleton JA. Abstract MP004: Dietary Intakes of Saturated Fat and Cardiovascular Disease: Results from the Multi-Ethnic Study of Atherosclerosis. Circulation; 2012.

422. Owen AJ, Magliano DJ, O’Dea K, Barr ELM, Shaw JE. Polyunsaturated fatty acid intake and risk of cardiovascular mortality in a low fish-consuming population: a prospective cohort analysis. Eur J Nutr. 2016;55(4):1605-13.

423. Owen A, Magliano D, O’Dea K, Shaw J. Dietary Polyunsaturated Fat Intake and Risk of Cardiovascular Mortality in the AusDiab Cohort. Heart Lung Circ. 2012;21:S307-S8.

424. Ozawa M, Yoshida D, Hata J, Ohara T, Mukai N, Shibata M, et al. Dietary Protein Intake and Stroke Risk in a General Japanese Population. Stroke. 2017;48(6):1478-86.

425. Paik JK, Park M, Shin JE, Jang S-Y, Shin J-Y. Dietary Protein to Carbohydrate Ratio and Incidence of Metabolic Syndrome in Korean Adults Based on a Long-Term Prospective Community-Based Cohort. Nutrients. 2020;12(11):3274.

426. Palli D, Russo A, Saieva C, Salvini S, Amorosi A, Decarli A. Dietary and familial determinants of 10-year survival among patients with gastric carcinoma. Cancer. 2000;89(6):1205-13.

427. Pan K, Larson JC, Chlebowski RT, Mortimer JE, Manson JE, Van Horn L, et al. Protein intake and breast cancer incidence and mortality. J Clin Oncol. 2020;38(15).

428. Pan K, Larson JC, Prentice RL, Mortimer JE, Neuhouser ML, Manson JE, et al. Protein Intake by Source and Breast Cancer Incidence and Mortality: The Women's Health Initiative. JNCI Cancer Spectrum. 2020;4(6).

429. Papadimitriou N, Bouras E, van den Brandt PA, Muller DC, Papadopoulou A, Heath AK, et al. A Prospective Diet-Wide Association Study for Risk of Colorectal Cancer in EPIC. CGH. 2022;20(4):864-73.e13.

430. Park I, Xun P, Tsinovoi CL, Klemmer P, Liu K, He K. Intakes of long-chain omega-3 polyunsaturated fatty acids and non-fried fish in relation to incidence of chronic kidney disease in young adults: a 25-year follow-up. Eur J Nutr. 2020;59(1):399-407.

431. Park S-Y, Kolonel LN, Henderson BE, Wilkens LR. Dietary Fat and Breast Cancer in Postmenopausal Women According to Ethnicity and Hormone Receptor Status: The Multiethnic Cohort Study. Cancer Prev Res. 2012;5(2):216-28.

432. Park S-Y, Murphy SP, Wilkens LR, Henderson BE, Kolonel LN. Fat and meat intake and prostate cancer risk: The multiethnic cohort study. Int J Cancer. 2007;121(6):1339-45.

433. Park S, Lee S, Kim Y, Lee Y, Kang MW, Kim K, et al. Causal effects of relative fat, protein, and carbohydrate intake on chronic kidney disease: a Mendelian randomization study. The American Journal of Clinical Nutrition. 2021;113(4):1023-31.

434. Patel AV, McCullough ML, Pavluck AL, Jacobs EJ, Thun MJ, Calle EE. Glycemic load, glycemic index, and carbohydrate intake in relation to pancreatic cancer risk in a large US cohort. CCC. 2007;18(3):287-94.

435. Patterson RE, Flatt SW, Newman VA, Natarajan L, Rock CL, Thomson CA, et al. Marine Fatty Acid Intake Is Associated with Breast Cancer Prognosis. J Nutr. 2011;141(2):201-6.

436. Pedersen M, Stripp C, Klarlund M, Olsen SF, Tjønneland AM, Frisch M. Diet and risk of rheumatoid arthritis in a prospective cohort. J Rheumatol. 2005;32(7):1249-52.

437. Pelser C, Mondul A, Hollenbeck A, Park Y, editors. DIETARY FAT AND THE RISK OF PROSTATE CANCER IN THE NIH-AARP DIET AND HEALTH STUDY. AMERICAN JOURNAL OF EPIDEMIOLOGY; 2012: OXFORD UNIV PRESS INC JOURNALS DEPT, 2001 EVANS RD, CARY, NC 27513 USA.

438. Pelser C, Mondul AM, Hollenbeck AR, Park Y. Dietary Fat, Fatty Acids, and Risk of Prostate Cancer in the NIH-AARP Diet and Health Study. CEBP. 2013;22(4):697-707.

439. Perez-Cornago A, Huybrechts I, Appleby PN, Schmidt JA, Crowe FL, Overvad K, et al. Intake of individual fatty acids and risk of prostate cancer in the European prospective investigation into cancer and nutrition. Int J Cancer. 2020;146(1):44-57.

440. Pérez de Arcelus M, Toledo E, Martínez-González MÁ, Sayón-Orea C, Gea A, Moreno-Montañés J. Omega 3:6 ratio intake and incidence of glaucoma: The SUN cohort. Clin Nutr. 2014;33(6):1041-5.

441. Pérez-Heras A, Ros E, Serra-Mir M, Vinyas C, Mestre C, Alegret M, Laguna JC. Intake of simple sugar-S from sweetened beverages is associated with cancer incidence and mortality in the predimed study cohort. Rev esp nutr hum diet. 2016;20:425-6.

442. Pertiwi K, Wanders AJ, Harbers MC, Küpers LK, Soedamah-Muthu SS, de Goede J, et al. Plasma and Dietary Linoleic Acid and 3-Year Risk of Type 2 Diabetes After Myocardial

Infarction: A Prospective Analysis in the Alpha Omega Cohort. Diabetes Care. 2020;43(2):358-65.

443. Petrick JL, Castro-Webb N, Gerlovin H, Bethea TN, Li S, Ruiz-Narváez EA, et al. A Prospective Analysis of Intake of Red and Processed Meat in Relation to Pancreatic Cancer among African American Women. CEBP. 2020;29(9):1775-83.

444. Pickering RT, Yuan M, Singer MR, Castor LL, Moore LL. Abstract MP55: Protein Intake is Associated With Lower Risk of Type 2 Diabetes in the Framingham Offspring Study. Circulation. 2020;141(Suppl_1):AMP55-AMP.

445. Pietinen P, Malila N, Virtanen M, Hartman TJ, Tangrea JA, Albanes D, Virtamo J. Diet and risk of colorectal cancer in a cohort of Finnish men. CCC. 1999;10(5):387-96.

446. Playdon M, Nagle C, Ibiebele T, Protani M, Carter J, Hyde S, et al., editors. THE ASSOCIATION BETWEEN DIET, SUPPLEMENT USE AND SURVIVAL AFTER A DIAGNOSIS OF INVASIVE OVARIAN CANCER. Int J Gynecol Cancer; 2014: LIPPINCOTT WILLIAMS & WILKINS 530 WALNUT ST, PHILADELPHIA, PA 19106-3621 USA.

447. Playdon MC, Nagle CM, Ibiebele TI, Ferrucci LM, Protani MM, Carter J, et al. Pre-diagnosis diet and survival after a diagnosis of ovarian cancer. Br J Cancer. 2017;116(12):1627-37.

448. Podoltsev NA, Wang X, Wang R, Hofmann JN, Liao LM, Zeidan AM, et al. Diet and Risk of Myeloproliferative Neoplasms in Older Individuals from the NIH-AARP Cohort. CEBP. 2020;29(11):2343-50.

449. Prentice RL, Pettinger M, Neuhouser ML, Raftery D, Zheng C, Gowda GAN, et al. Biomarker-Calibrated Macronutrient Intake and Chronic Disease Risk among Postmenopausal Women. J Nutr. 2021;151(8):2330-41.

450. Prentice RL, Huang Y, Kuller LH, Tinker LF, Horn LV, Stefanick ML, et al. Biomarker-calibrated Energy and Protein Consumption and Cardiovascular Disease Risk Among Postmenopausal Women. Epidemiology. 2011;22(2):170-9.

451. Prentice RL, Pettinger M, Zheng C, Neuhouser ML, Raftery D, Gowda GAN, et al. Biomarkers for Components of Dietary Protein and Carbohydrate with Application to Chronic Disease Risk in Postmenopausal Women. J Nutr. 2022;152(4):1107-17.

452. Prentice RL, Shaw PA, Bingham SA, Beresford SAA, Caan B, Neuhouser ML, et al. Biomarker-calibrated Energy and Protein Consumption and Increased Cancer Risk Among Postmenopausal Women. American Journal of Epidemiology. 2009;169(8):977-89.

453. Prineas RJ, Folsom AR, Zhang ZM, Sellers TA, Potter J. Nutrition and other risk factors for renal cell carcinoma in postmenopausal women. Epidemiology. 1997;8(1):31-6.

454. Prinelli F, Adorni F, Leite LC, Musicco M, editors. Macronutrients intake in adulthood and risk of dementia in old age: a 20-years follow-up Italian study. J Alzheimers Dis; 2016: IOS PRESS NIEUWE HEMWEG 6B, 1013 BG AMSTERDAM, NETHERLANDS.

455. Puaschitz N, Norekvaal TM, Dahl L, Assmuss J, Strand E, Pedersen ER, et al. Intake of dietary saturated fat is not associated with increased risk of coronary events in patients with suspected coronary artery disease. Eur J Prev Cardiol. 2013;20(1):S8.

456. Puaschitz NG, Strand E, Norekvål TM, Dierkes J, Dahl L, Svingen GFT, et al. Dietary Intake of Saturated Fat Is Not Associated with Risk of Coronary Events or Mortality in Patients with Established Coronary Artery Disease. J Nutr. 2015;145(2):299-305.

457. Qi H, Xia D, Xu X. Dietary glycemic index, glycemic load, and renal cancer risk: findings from prostate, lung, colorectal, and ovarian cancer trial. Front Nutr. 2023;10.

458. Ramdas WD, Wolfs RCW, Kiefte-de Jong JC, Hofman A, de Jong PTVM, Vingerling JR, Jansonius NM. Nutrient intake and risk of open-angle glaucoma: the Rotterdam Study. Eur J Epidemiol. 2012;27(5):385-93.

459. Ramne S, Alves Dias J, González-Padilla E, Olsson K, Lindahl B, Engström G, et al. Association between added sugar intake and mortality is nonlinear and dependent on sugar source in 2 Swedish population–based prospective cohorts. The American Journal of Clinical Nutrition. 2019;109(2):411-23.

460. Rebello SA, Koh H, Chen C, Naidoo N, Odegaard AO, Koh W-P, et al. Amount, type, and sources of carbohydrates in relation to ischemic heart disease mortality in a Chinese population: a prospective cohort study. The American Journal of Clinical Nutrition. 2014;100(1):53-64.

461. Reiner MF, Stivala S, Limacher A, Bonetti NR, Méan M, Egloff M, et al. Omega-3 fatty acids predict recurrent venous thromboembolism or total mortality in elderly patients with acute venous thromboembolism. JTH. 2017;15(1):47-56.

462. Reynolds R, Rosner B, Seddon JM. Dietary Omega-3 Fatty Acids, Other Fat Intake, Genetic Susceptibility, and Progression to Incident Geographic Atrophy. Ophthalmology. 2013;120(5):1020-8.

463. Rhee JJ, Kim E, Buring JE, Kurth T. Fish Consumption, Omega-3 Fatty Acids, and Risk of Cardiovascular Disease. American Journal of Preventive Medicine. 2017;52(1):10-9.

464. Rice M, Tworoger S, editors. ADOLESCENT AND ADULT DIETARY FAT INTAKE AND OVARIAN CANCER RISK. AMERICAN JOURNAL OF EPIDEMIOLOGY; 2013: OXFORD UNIV PRESS INC JOURNALS DEPT, 2001 EVANS RD, CARY, NC 27513 USA.

465. Rice MS, Poole EM, Willett WC, Tworoger SS. Adult dietary fat intake and ovarian cancer risk. Int J Cancer. 2020;146(10):2756-72.

466. Rissanen T, Voutilainen S, Nyyssönen K, Lakka TA, Salonen JT. Fish Oil–Derived Fatty Acids, Docosahexaenoic Acid and Docosapentaenoic Acid, and the Risk of Acute Coronary Events. Circulation. 2000;102(22):2677-9.

467. Rix TA, Joensen AM, Riahi S, Lundbye-Christensen S, Tjønneland A, Schmidt EB, Overvad K. A U-shaped association between consumption of marine n-3 fatty acids and development of atrial fibrillation/atrial flutter—a Danish cohort study. EP Europace. 2014;16(11):1554-61.

468. Rohan TE, Hiller JE, McMichael AJ. Dietary factors and survival from breast cancer. Nutr Cancer. 1993;20(2):167-77.

469. Romieu I, Ferrari P, Rinaldi S, Slimani N, Jenab M, Olsen A, et al. Dietary glycemic index and glycemic load and breast cancer risk in the European Prospective Investigation into Cancer and Nutrition (EPIC). The American Journal of Clinical Nutrition. 2012;96(2):345-55.

470. Rozanski A, Arnson Y, Gransar H, Hayes SW, Friedman JD, Thomson LEJ, Berman DS. Relation of Intake of Saturated Fat to Atherosclerotic Risk Factors, Health Behaviors, Coronary Atherosclerosis, and All-Cause Mortality Among Patients Who Underwent Coronary Artery Calcium Scanning. The American Journal of Cardiology. 2021;138:40-5.

471. Sahni S, Cupples LA, Mclean RR, Tucker KL, Broe KE, Kiel DP, Hannan MT. Protective effect of high protein and calcium intake on the risk of hip fracture in the framingham offspring cohort*. JBMR. 2010;25(12):2770-6.

472. Sakurai M, Nakamura K, Miura K, Takamura T, Yoshita K, Morikawa Y, et al. Dietary glycemic index and risk of type 2 diabetes mellitus in middle-aged Japanese men. Metabolism. 2012;61(1):47-55.

473. Sala-Vila A, Guasch-Ferré M, Hu FB, Sánchez-Tainta A, Bulló M, Serra-Mir M, et al. Dietary α-Linolenic Acid, Marine ω-3 Fatty Acids, and Mortality in a Population With High Fish Consumption: Findings From the PREvención con DIeta MEDiterránea (PREDIMED) Study. JAHA. 2016;5(1):e002543.

474. Sánchez-Villegas A, Verberne L, De Irala J, Ruíz-Canela M, Toledo E, Serra-Majem L, Martínez-González MA. Dietary Fat Intake and the Risk of Depression: The SUN Project. PLOS ONE. 2011;6(1):e16268.

475. Sanchez-Villegas A, Zazpe I, Santiago S, Perez-Cornago A, Martinez-Gonzalez MA, Lahortiga-Ramos F. Added sugars and sugar-sweetened beverage consumption, dietary carbohydrate index and depression risk in the Seguimiento Universidad de Navarra (SUN) Project. Br J Nutr. 2018;119(2):211-21.

476. Sauvaget C, Nagano J, Hayashi M, Yamada M. Animal protein, animal fat, and cholesterol intakes and risk of cerebral infarction mortality in the adult health study. Stroke. 2004;35(7):1531-7.

477. Sawada N, Inoue M, Iwasaki M, Sasazuki S, Shimazu T, Yamaji T, et al. Consumption of n-3 Fatty Acids and Fish Reduces Risk of Hepatocellular Carcinoma. Gastroenterology. 2012;142(7):1468-75.

478. Schmidt JA, Huybrechts I, Overvad K, Eriksen AK, Tjønneland A, Kaaks R, et al. Protein and amino acid intakes in relation to prostate cancer risk and mortality—A prospective study in the European Prospective Investigation into Cancer and Nutrition. Cancer Med. 2023;12(4):4725-38.

479. Schwartz B, Choi SY, Cook-Wiens G, Kelsey SF, Malhotra P, Handberg EM, et al. DIET, ANGIOGRAPHIC CORONARY DISEASE, AND CARDIOVASCULAR OUTCOMES IN WOMEN. JACC. 2022;79(9, Supplement):1598.

480. Sczaniecka AK, Brasky TM, Lampe JW, Patterson RE, White E. Dietary Intake of Specific Fatty Acids and Breast Cancer Risk Among Postmenopausal Women in the VITAL Cohort. Nutr Cancer. 2012;64(8):1131-42.

481. Seidelmann SB, Claggett B, Cheng S, Henglin M, Shah A, Steffen LM, et al. Dietary carbohydrate intake and mortality: a prospective cohort study and meta-analysis. Lancet Public Health. 2018;3(9):e419-e28.

482. Seino F, Date C, Nakayama T, Yoshiike N, Yokoyama T, Yamaguchi M, Tanaka H. Dietary lipids and incidence of cerebral infarction in a Japanese rural community. J Nutr Sci Vitaminol (Tokyo). 1997;43(1):83-99.

483. Sellem L, Srour B, Guéraud F, Pierre F, Kesse-Guyot E, Fiolet T, et al. Saturated, mono- and polyunsaturated fatty acid intake and cancer risk: results from the French prospective cohort NutriNet-Santé. Eur J Nutr. 2019;58(4):1515-27.

484. Sellers TA, Bazyk AE, Bostick RM, Kushi LH, Olson JE, Anderson KE, et al. Diet and risk of colon cancer in a large prospective study of older women: an analysis stratified on family history (Iowa, United States). CCC. 1998;9(4):357-67.

485. Setiawan VW, Pandol SJ, Porcel J, Wei PC, Wilkens LR, Le Marchand L, et al. Dietary Factors Reduce Risk of Acute Pancreatitis in a Large Multiethnic Cohort. CGH. 2017;15(2):257-65.e3.

486. Sharma S, Vik S, Pakseresht M, Shen L, Kolonel LN. Diet impacts mortality from cancer: results from the multiethnic cohort study. CCC. 2013;24(4):685-93.

487. Shen J, Johnson VM, Sullivan LM, Jacques PF, Magnani JW, Lubitz SA, et al. Dietary factors and incident atrial fibrillation: the Framingham Heart Study. The American Journal of Clinical Nutrition. 2011;93(2):261-6.

488. Shikany JM, Redden DT, Neuhouser ML, Chlebowski RT, Rohan TE, Simon MS, et al. Dietary Glycemic Load, Glycemic Index, and Carbohydrate and Risk of Breast Cancer in the Women's Health Initiative. Nutr Cancer. 2011;63(6):899-907.

489. Shimomura Y, Sobue T, Zha L, Kitamura T, Iwasaki M, Inoue M, et al. Association between meat, fish, and fatty acid intake and incidence of acute myeloid leukemia and myelodysplastic syndrome: the Japan Public Health Center-based Prospective Study. EHPM. 2023;28:19-.

490. Shimomura Y, Sobue T, Zha L, Kitamura T, Iwasaki M, Inoue M, et al. Association between Meat, Fish, and Fatty Acid Intake and Non-Hodgkin Lymphoma Incidence: The Japan Public Health Center–Based Prospective Study. J Nutr. 2022;152(8):1895-906.

491. Shin A, Cho S, Sandin S, Lof M, Oh MY, Weiderpass E. Omega-3 and -6 Fatty Acid Intake and Colorectal Cancer Risk in Swedish Women's Lifestyle and Health Cohort. Cancer Res Treat. 2020;52(3):848-54.

492. Shin D, Lee KW. Dietary carbohydrates interact with AMY1 polymorphisms to influence the incidence of type 2 diabetes in Korean adults. Scientific Reports. 2021;11(1):16788.

493. Shin W-K, Huang D, la Torre KD, Min S, Shin A, Lee J-k, et al. Abstract 4242: High sugar food consumption and breast cancer risk in a prospective cohort study. Cancer Res. 2023;83(7_Supplement):4242-.

494. Shu X, Calvert JK, Cai H, Xiang Y-B, Li H, Zheng W, et al. Plant and Animal Protein Intake and Risk of Incident Kidney Stones: Results from the Shanghai Men's and Women's Health Studies. Urol J. 2019;202(6):1217-23.

495. Shu X, Yu D, Shu X-o, Munro HM, Zheng W, Blot WJ. Investigating the associations of glycemic load and glycemic index with lung cancer risk in the Southern Community Cohort Study. CCC. 2020;31(12):1069-77.

496. Sidahmed E, Freedland SJ, Wu K, Genkinger JM, Smith-Warner SA. Abstract 1738: A pooled analysis of carbohydrate and dietary fiber intake and prostate cancer risk. Cancer Res. 2016;76(14_Supplement):1738-.

497. Sieri S, Chiodini P, Agnoli C, Pala V, Berrino F, Trichopoulou A, et al. Dietary Fat Intake and Development of Specific Breast Cancer Subtypes. JNCI. 2014;106(5).

498. Sieri S, Krogh V, Agnoli C, Ricceri F, Palli D, Masala G, et al. Dietary glycemic index and glycemic load and risk of colorectal cancer: results from the EPIC-Italy study. Int J Cancer. 2015;136(12):2923-31.

499. Sieri S, Krogh V, Ferrari P, Berrino F, Pala V, Thiébaut ACM, et al. Dietary fat and breast cancer risk in the European Prospective Investigation into Cancer and Nutrition. The American Journal of Clinical Nutrition. 2008;88(5):1304-12.

500. Sieri S, Pala V, Brighenti F, Agnoli C, Grioni S, Berrino F, et al. High glycemic diet and breast cancer occurrence in the Italian EPIC cohort. Nutr Metab Cardiovasc Dis. 2013;23(7):628-34.

501. Sieri S, Agnoli C, Pala V, Mattiello A, Panico S, Masala G, et al. [Dietary habits and cancer: the experience of EPIC-Italy]. Epidemiol Prev. 2015;39(5-6):333-8.

502. Sieri S, Krogh V, Berrino F, Evangelista A, Agnoli C, Brighenti F, et al. Dietary Glycemic Load and Index and Risk of Coronary Heart Disease in a Large Italian Cohort: The EPICOR Study. Arch Intern Med. 2010;170(7):640-7.

503. Silvera SA, Jain M, Howe GR, Miller AB, Rohan TE. Glycaemic index, glycaemic load and ovarian cancer risk: a prospective cohort study. Public Health Nutr. 2007;10(10):1076-81.

504. Silvera SA, Rohan TE, Jain M, Terry PD, Howe GR, Miller AB. Glycaemic index, glycaemic load and risk of endometrial cancer: a prospective cohort study. Public Health Nutr. 2005;8(7):912-9.

505. Silvera SA, Rohan TE, Jain M, Terry PD, Howe GR, Miller AB. Glycemic index, glycemic load, and pancreatic cancer risk (Canada). CCC. 2005;16:431-6.

506. Simon MS, Shikany JM, Neuhouser ML, Rohan T, Nirmal K, Cui Y, Abrams J. Glycemic index, glycemic load, and the risk of pancreatic cancer among postmenopausal women in the women's health initiative observational study and clinical trial. CCC. 2010;21(12):2129-36.

507. Sjögren P, Becker W, Warensjö E, Olsson E, Byberg L, Gustafsson I-B, et al. Mediterranean and carbohydrate-restricted diets and mortality among elderly men: a cohort study in Sweden. The American Journal of Clinical Nutrition. 2010;92(4):967-74.

508. Soinio M, Laakso M, Lehto S, Hakala P, Rönnemaa T. Dietary fat predicts coronary heart disease events in subjects with type 2 diabetes. Diabetes Care. 2003;26(3):619-24.

509. Sonestedt E, Gullberg B, Wirfält E, Hedblad B, Orho-Melander M. Association between fat intake, physical activity and mortality depending on genetiv variation in FTO. Diabetologia. 2009;52(Suppl 1):104.

510. Song H, Jeong A, Tran TXM, Lee J, Kim M, Park B. Association between Micronutrient Intake and Breast Cancer Risk According to Body Mass Index in South Korean Adult Women: A Cohort Study. Nutrients. 2022;14(13):2644.

511. Song M, Nishihara R, Wu K, Qian ZR, Kim SA, Sukawa Y, et al. Marine ω-3 Polyunsaturated Fatty Acids and Risk for Colorectal Cancer According to Microsatellite Instability. JNCI. 2015;107(4).

512. Song M, Zhang X, Meyerhardt JA, Giovannucci EL, Ogino S, Fuchs CS, Chan AT. Marine ω-3 polyunsaturated fatty acid intake and survival after colorectal cancer diagnosis. Gut. 2017;66(10):1790-6.

513. Song M, Nishihara R, Cao Y, Chun E, Qian ZR, Mima K, et al. Marine ω-3 Polyunsaturated Fatty Acid Intake and Risk of Colorectal Cancer Characterized by Tumor-Infiltrating T Cells. JAMA Oncology. 2016;2(9):1197-206.

514. Steell L, Sillars A, Welsh P, Iliodromiti S, Wong SC, Pell JP, et al. Associations of dietary protein intake with bone mineral density: An observational study in 70,215 UK Biobank participants. Bone. 2019;120:38-43.

515. Steffen BT, Steffen LM, Zhou X, Ouyang P, Weir NL, Tsai MY. n-3 Fatty Acids Attenuate the Risk of Diabetes Associated With Elevated Serum Nonesterified Fatty Acids: The Multi-Ethnic Study of Atherosclerosis. Diabetes Care. 2015;38(4):575-80.

516. Stern MC, Butler LM, Corral R, Joshi AD, Yuan J-M, Koh W-P, Yu MC. Polyunsaturated Fatty Acids, DNA Repair Single Nucleotide Polymorphisms and Colorectal Cancer in the Singapore Chinese Health Study. J Nutrigenet Nutrigenomics. 2010;2(6):273-9.

517. Stobäus N, Müller MJ, Küpferling S, Schulzke J-D, Norman K. Low Recent Protein Intake Predicts Cancer-Related Fatigue and Increased Mortality in Patients with Advanced Tumor Disease Undergoing Chemotherapy. Nutr Cancer. 2015;67(5):818-24.

518. Stolzenberg-Solomon RZ, Pietinen P, Taylor PR, Virtamo J, Albanes D. Prospective Study of Diet and Pancreatic Cancer in Male Smokers. American Journal of Epidemiology. 2002;155(9):783-92.

519. Strand E, Pedersen ER, Svingen GFT, Schartum-Hansen H, Rebnord EW, Bjørndal B, et al. Dietary intake of n-3 long-chain polyunsaturated fatty acids and risk of myocardial infarction in coronary artery disease patients with or without diabetes mellitus: a prospective cohort study. BMC Medicine. 2013;11(1):216.

520. Strayer L, Jacobs DR, Schairer C, Schatzkin A, Flood A. Dietary carbohydrate, glycemic index, and glycemic load and the risk of colorectal cancer in the BCDDP cohort. CCC. 2007;18(8):853-63.

521. Streppel MT, Ocké MC, Boshuizen HC, Kok FJ, Kromhout D. Long-term fish consumption and n-3 fatty acid intake in relation to (sudden) coronary heart disease death: the Zutphen study. Eur Heart J. 2008;29(16):2024-30.

522. Sugihiro T, Yoneda M, Ohno H, Oki K, Hattori N. Associations of nutrient intakes with obesity and diabetes mellitus in the longitudinal medical surveys of Japanese Americans. J Diabetes Investig. 2019;10(5):1229-36.

523. Sun C, Zhang W-S, Jiang C-Q, Jin Y-L, Deng X-Q, Woo J, et al. Low-Carbohydrate Diets and Mortality in Older Asian People: A 15-Year Follow-Up from a Prospective Cohort Study. Nutrients. 2022;14(7):1406.

524. Sun J-W, Zheng W, Li H-L, Gao J, Yang G, Gao Y-T, et al. Dietary Glycemic Load, Glycemic Index, and Carbohydrate Intake on the Risk of Lung Cancer among Men and Women in Shanghai. Nutr Cancer. 2018;70(4):671-7.

525. Taha HM, Rozek LS, Chen YT, Slade AN, Wolf GT, Arthur A. Risk of mortality varies by type of fat consumed in a longitudinal cohort of head and neck cancer patients. American Society of Clinical Oncology; 2019.

526. Taha HM, Rozek LS, Chen X, Li Z, Zarins KR, Slade AN, et al. Risk of Disease Recurrence and Mortality Varies by Type of Fat Consumed before Cancer Treatment in a Longitudinal Cohort of Head and Neck Squamous Cell Carcinoma Patients. J Nutr. 2022;152(5):1298-305.

527. Tajik B, Tuomainen T-P, Isanejad M, Salonen JT, Virtanen JK. Serum n-6 polyunsaturated fatty acids and risk of atrial fibrillation: the Kuopio Ischaemic Heart Disease Risk Factor Study. Eur J Nutr. 2022;61(4):1981-9.

528. Takeuchi H, Kawashima R. Nutrients and Dementia: Prospective Study. Nutrients. 2023;15(4):842.

529. Tan L-J, Shin S. Effects of oily fish and its fatty acid intake on non-alcoholic fatty liver disease development among South Korean adults. Front Nutr. 2022;9.

530. Tasevska N, Jiao L, Cross AJ, Kipnis V, Subar AF, Hollenbeck A, et al. Sugars in diet and risk of cancer in the NIH-AARP Diet and Health Study. Int J Cancer. 2012;130(1):159-69.

531. Tasevska N, Park Y, Jiao L, Hollenbeck A, Subar AF, Potischman N. Sugars and risk of mortality in the NIH-AARP Diet and Health Study. The American Journal of Clinical Nutrition. 2014;99(5):1077-88.

532. Tasevska N, Pettinger M, Kipnis V, Midthune D, Tinker LF, Potischman N, et al. Associations of Biomarker-Calibrated Intake of Total Sugars With the Risk of Type 2 Diabetes and Cardiovascular Disease in the Women’s Health Initiative Observational Study. American Journal of Epidemiology. 2018;187(10):2126-35.

533. Taylor EN, Stampfer MJ, Curhan GC. Fatty acid intake and incident nephrolithiasis. Am J Kidney Dis. 2005;45(2):267-74.

534. Teng GG, Pan A, Yuan J-M, Koh W-P. Food Sources of Protein and Risk of Incident Gout in the Singapore Chinese Health Study. A&R. 2015;67(7):1933-42.

535. Teng GG, Pan A, Yuan JM, Koh WP. Food sources of protein and risk of incident gout in the Singapore Chinese health study. A&R. 2014;66:S824-S5.

536. Narasaki Y, Okuda Y, Moore LW, You AS, Nakata T, Nguyen DV, et al. Dietary protein intake, kidney function, and mortality in a nationally representative cohort. JASN. 2019;30:967-8.

537. Teymoori F, Farhadnejad H, Jahromi MK, Vafa M, Ahmadirad H, Mirmiran P, Azizi F. Dietary protein score and carbohydrate quality index with the risk of chronic kidney disease: Findings from a prospective cohort study. Front Nutr. 2022;9.

538. Tharrey M, Mariotti F, Mashchak A, Barbillon P, Delattre M, Fraser GE. Patterns of plant and animal protein intake are strongly associated with cardiovascular mortality: the Adventist Health Study-2 cohort. Int J Epidemiol. 2018;47(5):1603-12.

539. Thesing CS, Lok A, Milaneschi Y, Assies J, Bockting CLH, Figueroa CA, et al. Fatty acids and recurrence of major depressive disorder: combined analysis of two Dutch clinical cohorts. Acta Psychiatrica Scandinavica. 2020;141(4):362-73.

540. Thiébaut AC, Clavel-Chapelon F. [Fat consumption and breast cancer: preliminary results from the E3N-Epic cohort]. Bull Cancer. 2001;88(10):954-8.

541. Thiébaut ACM, Chajès V, Gerber M, Boutron-Ruault M-C, Joulin V, Lenoir G, et al. Dietary intakes of ω-6 and ω-3 polyunsaturated fatty acids and the risk of breast cancer. Int J Cancer. 2009;124(4):924-31.

542. Tokede OA, Petrone AB, Hanson NQ, Tsai MY, Weir NA, Glynn RJ, et al. Plasma phospholipid trans fatty acids and risk of heart failure. The American Journal of Clinical Nutrition. 2013;97(4):698-705.

543. Tram L, Bork CS, Venø SK, Lasota AN, Lundbye-Christensen S, Schmidt EB, Overvad K. Intake of marine n-3 polyunsaturated fatty acids and the risk of incident peripheral artery disease. Eur J Clin Nutr. 2021;75(10):1483-90.

544. Trichopoulou A, Psaltopoulou T, Orfanos P, Hsieh CC, Trichopoulos D. Low-carbohydrate–high-protein diet and long-term survival in a general population cohort. Eur J Clin Nutr. 2007;61(5):575-81.

545. Trichopoulou A, Psaltopoulou T, Orfanos P, Trichopoulos D. Diet and physical activity in relation to overall mortality amongst adult diabetics in a general population cohort. J Intern Med. 2006;259(6):583-91.

546. Tu S, Gallagher C, Elliott A, Pitman B, Hendriks J, Lau D, et al. Nutrient Intake and Risk of Atrial Fibrillation in the UK Biobank. Heart Lung Circ. 2022;31:S124.

547. Tu SJ, Gallagher C, Elliott AD, Pitman BM, Hendriks JML, Lau DH, et al. PO-670-02 DIETARY MACRONUTRIENT INTAKE AND INCIDENT ATRIAL FIBRILLATION: AN OBSERVATIONAL STUDY OF 123,151 INDIVIDUALS. Heart Rhythm. 2022;19(5, Supplement):S320-S1.

548. Tucker KL, Hallfrisch J, Qiao N, Muller D, Andres R, Fleg JL. The Combination of High Fruit and Vegetable and Low Saturated Fat Intakes Is More Protective against Mortality in Aging Men than Is Either Alone: The Baltimore Longitudinal Study of Aging1. J Nutr. 2005;135(3):556-61.

549. Van Baak MA, Larsen TM, Jebb SA, Martinez A, Saris WHM, Handjieva-Darlenska T, et al. Dietary Intake of Protein from Different Sources and Weight Regain, Changes in Body Composition and Cardiometabolic Risk Factors after Weight Loss: The DIOGenes Study. Nutrients. 2017;9(12):1326.

550. Van Blarigan EL, Fuchs CS, Niedzwiecki D, Ye X, Zhang S, Song M, et al. Marine ω-3 Polyunsaturated Fatty Acid and Fish Intake after Colon Cancer Diagnosis and Survival: CALGB 89803 (Alliance). CEBP. 2018;27(4):438-45.

551. Varraso R, Kabrhel C, Goldhaber SZ, Rimm EB, Camargo CA, Jr. Prospective Study of Diet and Venous Thromboembolism in US Women and Men. American Journal of Epidemiology. 2011;175(2):114-26.

552. Varraso R, Barr RG, Willett WC, Speizer FE, Camargo CA. Fish intake and risk of chronic obstructive pulmonary disease in 2 large US cohorts. The American Journal of Clinical Nutrition. 2015;101(2):354-61.

553. Velie E, Kulldorff M, Schairer C, Block G, Albanes D, Schatzkin A. Dietary fat, fat subtypes, and breast cancer in postmenopausal women: a prospective cohort study. J Natl Cancer Inst. 2000;92(10):833-9.

554. Venø SK, Bork CS, Jakobsen MU, Lundbye-Christensen S, McLennan PL, Bach FW, et al. Marine n-3 Polyunsaturated Fatty Acids and the Risk of Ischemic Stroke. Stroke. 2019;50(2):274-82.

555. Villegas R, Liu S, Gao Y-T, Yang G, Li H, Zheng W, Shu XO. Prospective Study of Dietary Carbohydrates, Glycemic Index, Glycemic Load, and Incidence of Type 2 Diabetes Mellitus in Middle-aged Chinese Women. Arch Intern Med. 2007;167(21):2310-6.

556. Virtanen JK, Koskinen TT, Virtanen HEK, Mursu J, Tuomainen T-P, Voutilainen S. Abstract MP070: Saturated Fat Intake by Food Source and Risk of Incident Coronary Heart Disease in Men: the Kuopio Ischaemic Heart Disease Risk Factor Study. Circulation. 2017;135.

557. Virtanen JK, Mozaffarian D, Willett WC, Feskanich D. Dietary intake of polyunsaturated fatty acids and risk of hip fracture in men and women. Osteoporos Int. 2012;23(11):2615-24.

558. Virtanen JK, Mursu J, Voutilainen S, Uusitupa M, Tuomainen T-P. Serum Omega-3 Polyunsaturated Fatty Acids and Risk of Incident Type 2 Diabetes in Men: The Kuopio Ischemic Heart Disease Risk Factor Study. Diabetes Care. 2013;37(1):189-96.

559. Vissers LET, Rijksen J, Boer JMA, Verschuren WMM, van der Schouw YT, Sluijs I. Fatty acids from dairy and meat and their association with risk of coronary heart disease. Eur J Nutr. 2019;58(7):2639-47.

560. Vogtmann E, Li HL, Shu XO, Chow WH, Ji BT, Cai H, et al. Dietary glycemic load, glycemic index, and carbohydrates on the risk of primary liver cancer among Chinese women and men. Ann Oncol. 2013;24(1):238-44.

561. Wallström P, Sonestedt E, Hlebowicz J, Ericson U, Drake I, Persson M, et al. Dietary Fiber and Saturated Fat Intake Associations with Cardiovascular Disease Differ by Sex in the Malmö Diet and Cancer Cohort: A Prospective Study. PLOS ONE. 2012;7(2):e31637.

562. Wang B, Tian G, Zhang Q. Vegetable Oil or Animal Fat Oil, Which is More Conducive to Cardiovascular Health Among the Elderly in China? Curr Probl Cardiol. 2023;48(2):101485.

563. Wang F, Baden MY, Rexrode KM, Hu FB. Abstract 9343: Dietary Fat Intake and the Risk of Stroke: Results from Two Prospective Cohort Studies. Circulation. 2021;144(Suppl_1):A9343-A.

564. Wang Y, Fang Y, Witting PK, Charchar FJ, Sobey CG, Drummond GR, Golledge J. Dietary fatty acids and mortality risk from heart disease in US adults: an analysis based on NHANES. Scientific Reports. 2023;13(1):1614.

565. Warfa K, Drake I, Wallström P, Engström G, Sonestedt E. Association between sucrose intake and acute coronary event risk and effect modification by lifestyle factors: Malmö Diet and Cancer Cohort Study. Br J Nutr. 2016;116(9):1611-20.

566. Watanabe S, Yoshihisa A, Kanno Y, Takiguchi M, Yokokawa T, Sato A, et al. Associations With Eicosapentaenoic Acid to Arachidonic Acid Ratio and Mortality in Hospitalized Heart Failure Patients. J Card Fail. 2016;22(12):962-9.

567. Watanabe Y, Katagiri R, Goto A, Shimazu T, Yamaji T, Sawada N, et al. Dietary glycemic index, glycemic load, and endometrial cancer risk: The Japan Public Health Center-based Prospective Study. Cancer Sci. 2021;112(9):3682-90.

568. Watling CZ, Kelly RK, Dunneram Y, Knuppel A, Piernas C, Schmidt JA, et al. Associations of intakes of total protein, protein from dairy sources, and dietary calcium with risks of colorectal, breast, and prostate cancer: a prospective analysis in UK Biobank. Br J Cancer. 2023;129(4):636-47.

569. Watling CZ, Kelly RK, Murphy N, Gunter M, Piernas C, Bradbury KE, et al. Prospective Analysis Reveals Associations between Carbohydrate Intakes, Genetic Predictors of Short-Chain Fatty Acid Synthesis, and Colorectal Cancer Risk. Cancer Res. 2023;83(12):2066-76.

570. Weaver A, Tooze J, Kritchevsky S, Houston D, Cauley J, Bauer D, Tylavsky F, editors. Effect of Dietary Protein Intake on Bone Mineral Density and Fracture Incidence in Older Adults in the Health, Aging, and Body Composition Study. JBMR; 2019: WILEY 111 RIVER ST, HOBOKEN 07030-5774, NJ USA.

571. Weaver AA, Tooze JA, Cauley JA, Bauer DC, Tylavsky FA, Kritchevsky SB, Houston DK. Effect of Dietary Protein Intake on Bone Mineral Density and Fracture Incidence in Older Adults in the Health, Aging, and Body Composition Study. J Gerontol A Biol Sci Med Sci. 2021;76(12):2213-22.

572. Webster J, Greenwood DC, Cade JE. Diet and hip fracture risk in the UK women's cohort study. Proc Nutr Soc. 2022;81(OCE5):E206.

573. Webster J, Greenwood DC, Cade JE. Foods, nutrients and hip fracture risk: A prospective study of middle-aged women. Clin Nutr. 2022;41(12):2825-32.

574. Wei B-Z, Li L, Dong C-W, Tan C-C, Xu W. The Relationship of Omega-3 Fatty Acids with Dementia and Cognitive Decline: Evidence from Prospective Cohort Studies of Supplementation, Dietary Intake, and Blood Markers. The American Journal of Clinical Nutrition. 2023;117(6):1096-109.

575. Wen W, Shu XO, Li H, Yang G, Ji B-T, Cai H, et al. Dietary carbohydrates, fiber, and breast cancer risk in Chinese women. The American Journal of Clinical Nutrition. 2009;89(1):283-9.

576. Wendeu-Foyet G, Chajes V, Huybrechts I, Bard J-M, Debras C, Chazelas E, et al. Abstract P3-12-35: Industrial and ruminant trans fatty acid intakes and cancer risk: Results from the NutriNet-Santé cohort. Cancer Res. 2022;82(4_Supplement):P3-12-35-P3-12-35.

577. Ma W, Wu JHY, Wang Q, Lemaitre RN, Mukamal KJ, Djoussé L, et al. Prospective association of fatty acids in the de novo lipogenesis pathway with risk of type 2 diabetes: the Cardiovascular Health Study. The American Journal of Clinical Nutrition. 2015;101(1):153-63.

578. Wild H, Gasevic D, Woods RL, McNeil J, Britt C, Owen A. The association between plant and animal protein intake and disability free survival in community-dwelling older adults: the results of the ASPREE Longitudinal Study of Older Persons (ALSOP). Proc Nutr Soc. 2023;82(OCE2):E120.

579. Willett WC, Stampfer MJ, Colditz GA, Rosner BA, Speizer FE. Relation of meat, fat, and fiber intake to the risk of colon cancer in a prospective study among women. N Engl J Med. 1990;323(24):1664-72.

580. Willett WC, Stampfer MJ, Manson JE, Colditz GA, Speizer FE, Rosner BA, et al. Intake of trans fatty acids and risk of coronary heart disease among women. Lancet. 1993;341(8845):581-5.

581. Wolk A, Bergström R, Hunter D, Willett W, Ljung H, Holmberg L, et al. A prospective study of association of monounsaturated fat and other types of fat with risk of breast cancer. Arch Intern Med. 1998;158(1):41-5.

582. Wu J, Cho E, Giovannucci EL, Rosner BA, Sastry SM, Willett WC, Schaumberg DA. Dietary Intakes of Eicosapentaenoic Acid and Docosahexaenoic Acid and Risk of Age-Related Macular Degeneration. Ophthalmology. 2017;124(5):634-43.

583. Wu J, Wilson KM, Stampfer MJ, Willett WC, Giovannucci EL. A 24-year prospective study of dietary α-linolenic acid and lethal prostate cancer. Int J Cancer. 2018;142(11):2207-14.

584. Xie J, Wang Z, Wang J, Feng W, Shan T, Jing S, et al. Intakes of omega-3 fatty acids and risks of all-cause and cause-specific mortality in people with diabetes: a cohort study based on NHANES 1999–2014. Acta Diabetologica. 2023;60(3):353-62.

585. Xu X, Yin Y, Niu L, Yang X, Du X, Tian Q. Association between Changes in Protein Intake and Risk of Cognitive Impairment: A Prospective Cohort Study. Nutrients. 2023;15(1):2.

586. Xu X, Shivappa N. Dietary glycemic index, glycemic load and risk of bladder cancer: a prospective study. Eur J Nutr. 2021;60(2):1041-8.

587. Yaemsiri S, Sen S, Tinker L, Rosamond W, Wassertheil-Smoller S, He K, editors. Dietary fat intake and incidence of ischemic stroke in postmenopausal US women: the Womens Health Initiative. Stroke; 2010: LIPPINCOTT WILLIAMS & WILKINS 530 WALNUT ST, PHILADELPHIA, PA 19106-3621 USA.

588. Yaemsiri S, Sen S, Tinker L, Rosamond W, Wassertheil-Smoller S, He K. Trans fat, aspirin, and ischemic stroke in postmenopausal women. Ann Neurol. 2012;72(5):704-15.

589. Yamaoka T, Araki A, Tamura Y, Tanaka S, Fujihara K, Horikawa C, et al. Association between Low Protein Intake and Mortality in Patients with Type 2 Diabetes. Nutrients. 2020;12(6):1629.

590. Yammine SG, Huybrechts I, Biessy C, Dossus L, Panico S, Sánchez MJ, et al. Dietary fatty acids and endometrial cancer risk within the European Prospective Investigation into Cancer and Nutrition. BMC Cancer. 2023;23(1):159.

591. Yammine S, Huybrechts I, Biessy C, Dossus L, Aglago EK, Naudin S, et al. Dietary and Circulating Fatty Acids and Ovarian Cancer Risk in the European Prospective Investigation into Cancer and Nutrition. CEBP. 2020;29(9):1739-49.

592. Yan J, Zheng K, Zhang X, Jiang Y. Fructose Consumption is Associated with a Higher Risk of Dementia and Alzheimer’s Disease: A Prospective Cohort Study. J Prev Alzheimers Dis. 2023;10(2):186-92.

593. Yang B, Glenn AJ, Liu Q, Madsen T, Allison MA, Shikany JM, et al. Added Sugar, Sugar-Sweetened Beverages, and Artificially Sweetened Beverages and Risk of Cardiovascular Disease: Findings from the Women's Health Initiative and a Network Meta-Analysis of Prospective Studies. Nutrients. 2022;14(20):4226.

594. Yang W, Sui J, Ma Y, Simon TG, Petrick JL, Lai M, et al. Dietary Fat Intake and Risk of Hepatocellular Carcinoma in Two Large Prospective Cohort Studies (FS13-07-19). Curr dev nutr. 2019;3:3013097.

595. Yang W, Sui J, Ma Y, Simon TG, Petrick JL, Lai M, et al. High Dietary Intake of Vegetable or Polyunsaturated Fats Is Associated With Reduced Risk of Hepatocellular Carcinoma. CGH. 2020;18(12):2775-83.e11.

596. Yao X, Xu X, Wang S, Xia D. Associations of Dietary Fat Intake With Mortality From All Causes, Cardiovascular Disease, and Cancer: A Prospective Study. Front Nutr. 2021;8.

597. Yates M, Luben R, Cheong E, Igali L, Fitzgerald R, Khaw KT, Hart A. Dietary fat intake in the aetiology of barrett's oesophagus and oesophageal adenocarcinoma: data from a prospective cohort study (EPIC-Norfolk) using 7-day food diary data. Gut. 2011;60(Suppl 1):A179-A.

598. Yates M, Luben R, Cheong E, Igali L, Fitzgerald R, Khaw K-T, Hart A. Dietary Fat Intake in the Aetiology of Barrett's Oesophagus and Oesophageal Adenocarcinoma – Data From a Prospective Cohort Study (EPIC-Norfolk) Using 7-Day Food Diary Data. Gastroenterology. 2011;140(5, Supplement 1):S-80.

599. Yishake D, He TT, Liu ZY, Chen S, Luo Y, Liu XZ, et al. Dietary protein and prognosis of hepatocellular carcinoma: a prospective cohort study. Food Funct. 2021;12(22):11568-76.

600. Yu D, Shu X-O, Li H, Xiang Y-B, Yang G, Gao Y-T, et al. Abstract MP24: High Intakes of Dietary Carbohydrate and Rice were Associated with Increased Risk of Coronary Heart Disease in Chinese Men and Women. Circulation. 2013;127(suppl_12):AMP24-AMP.

601. Yu D, Shu X-O, Li H, Yang G, Ding D, Hong Z, et al. Abstract T P159: Dietary Carbohydrates, Glycemic Index, Glycemic Load, and Risk of Stroke in Chinese Women: a Large Population-based, Prospective Cohort Study. Stroke. 2015;46(suppl_1):ATP159-ATP.

602. Yu D, Zhang X, Shu X-O, Cai H, Li H, Ding D, et al. Dietary glycemic index, glycemic load, and refined carbohydrates are associated with risk of stroke: a prospective cohort study in urban Chinese women. The American journal of clinical nutrition. 2016;104(5):1345-51.

603. Yu YC, Paragomi P, Jin A, Wang R, Schoen RE, Koh WP, et al. Low-Carbohydrate Diet Score and the Risk of Colorectal Cancer: Findings from the Singapore Chinese Health Study. CEBP. 2023;32(6):802-8.

604. Yu Y-C, Paragomi P, Jin A, Wang R, Schoen RE, Koh W-P, et al. Abstract 735: Low-carbohydrate diet score and the risk of colorectal cancer: Findings from a prospective cohort study. Cancer Res. 2022;82(12_Supplement):735-.

605. Yuan C, Joh H-K, Wang Q-L, Zhang Y, Smith-Warner SA, Wang M, et al. Sugar-sweetened beverage and sugar consumption and colorectal cancer incidence and mortality according to anatomic subsite. The American Journal of Clinical Nutrition. 2022;115(6):1481-9.

606. Yuan JM, Ross RK, Gao YT, Yu MC. Fish and shellfish consumption in relation to death from myocardial infarction among men in Shanghai, China. Am J Epidemiol. 2001;154(9):809-16.

607. Yuan M, Pickering R, Singer M, Moore LL. Abstract MP27: Saturated Fat Intake From Dairy Sources Is Associated With Reduced Cardiovascular Risk In Framingham Offspring Study Women. Circulation. 2020;141(Suppl_1):AMP27-AMP.

608. Yuan M, Pickering RT, Singer MR, Bradlee ML, Moore LL. Abstract MP40: Higher Intakes of Animal Protein Helps Maintain Functional Status and Grip Strength in Older Adults in the Framingham Offspring Study. Circulation. 2019;139(Suppl_1):AMP40-AMP.

609. Yuan S, Yu H-j, Liu M-w, Tang B-w, Zhang J, Gasevic D, et al. Fat Intake and Hypertension Among Adults in China: The Modifying Effects of Fruit and Vegetable Intake. American Journal of Preventive Medicine. 2020;58(2):294-301.

610. Zamani SA, McClain KM, Graubard BI, Liao LM, Abnet CC, Cook MB, Petrick JL. Dietary Polyunsaturated Fat Intake in Relation to Head and Neck, Esophageal, and Gastric Cancer Incidence in the National Institutes of Health–AARP Diet and Health Study. American Journal of Epidemiology. 2020;189(10):1096-113.

611. Zamanillo-Campos R, Chaplin A, Romaguera D, Abete I, Salas-Salvadó J, Martín V, et al. Longitudinal association of dietary carbohydrate quality with visceral fat deposition and other adiposity indicators. Clin Nutr. 2022;41(10):2264-74.

612. Zamora-Ros R, Rinaldi S, Tsilidis KK, Weiderpass E, Boutron-Ruault M-C, Rostgaard-Hansen AL, et al. Energy and macronutrient intake and risk of differentiated thyroid carcinoma in the European Prospective Investigation into Cancer and Nutrition study. Int J Cancer. 2016;138(1):65-73.

613. Zhang N, Cheng Y, Luo R, Chang D, Liu T, Wang Z, et al. Low-Carbohydrate-Diet Score and Mortality in Adults With and Without Chronic Kidney Disease: Results From the Third National Health and Nutrition Examination Survey. J Renal Nutr. 2022;32(3):301-11.

614. Zhang S, Li H, Meng G, Zhang Q, Liu L, Wu H, et al. Added sugar intake and its forms and sources in relation to risk of non-alcoholic fatty liver disease: results from the Tianjin Chronic Low-grade Systemic Inflammation and Health cohort study. Br J Nutr. 2023;129(12):2094-101.

615. Zhang S, Yan Y, Meng G, Zhang Q, Liu L, Wu H, et al. Protein foods from animal sources and risk of nonalcoholic fatty liver disease in representative cohorts from North and South China. J Intern Med. 2023;293(3):340-53.

616. Zhang S, Zhuang X, Lin X, Zhong X, Zhou H, Sun X, et al. Low-Carbohydrate Diets and Risk of Incident Atrial Fibrillation: A Prospective Cohort Study. JAHA. 2019;8(9):e011955.

617. Zhang X, Zhang J, Du W, Su C, Ouyang Y, Huang F, et al. Multi-Trajectories of Macronutrient Intake and Their Associations with Obesity among Chinese Adults from 1991 to 2018: A Prospective Study. Nutrients. 2022;14(1):13.

618. Zhang Y, Zhuang P, Mao L, Chen X, Wang J, Cheng L, et al. Current level of fish and omega-3 fatty acid intakes and risk of Type 2 diabetes in China. J Nutr Biochem. 2019;74:108249.

619. Zhang Y, Zhuang P, Wu F, He W, Mao L, Jia W, et al. Cooking oil/fat consumption and deaths from cardiometabolic diseases and other causes: prospective analysis of 521,120 individuals. BMC Medicine. 2021;19(1):92.

620. Zhao L-G, Li H-L, Liu D-K, Fang J, Wang J, Tan Y-T, Xiang Y-B. Dietary glycemic index, glycemic load, and cause-specific mortality: two population-based prospective cohort studies. Eur J Clin Nutr. 2022;76(8):1142-9.

621. Zheng G, Gong T-T, Ma Q-P, Wei Y-F, Du Z-D, Zhao J-Q, et al. The association of macronutrient quality and its interactions with energy intake with survival among patients with ovarian cancer: results from a prospective cohort study. The American Journal of Clinical Nutrition. 2023;117(6):1362-71.

622. Zhong G-C, Li Q-J, Yang P-F, Wang Y-B, Hao F-B, Wang K, et al. Low-carbohydrate diets and the risk of pancreatic cancer: a large prospective cohort study. Carcinogenesis. 2021;42(5):724-32.

623. Zhong VW, Allen NB, Greenland P, Carnethon MR, Ning H, Wilkins JT, et al. Protein foods from animal sources, incident cardiovascular disease and all-cause mortality: a substitution analysis. Int J Epidemiol. 2021;50(1):223-33.

624. Zhou C, Zhang Z, Liu M, Zhang Y, Li H, He P, et al. Dietary carbohydrate intake and new-onset diabetes: A nationwide cohort study in China. Metabolism. 2021;123:154865.

625. Zhu R, Larsen TM, Fogelholm M, Poppitt SD, Vestentoft PS, Silvestre MP, et al. Dose-Dependent Associations of Dietary Glycemic Index, Glycemic Load, and Fiber With 3-Year Weight Loss Maintenance and Glycemic Status in a High-Risk Population: A Secondary Analysis of the Diabetes Prevention Study PREVIEW. Diabetes Care. 2021;44(7):1672-81.

626. Zhu Y, Wang PP. Prediagnostic Consumption of Carbohydrates and Colorectal Cancer Survival: A Cohort Study. Ann Epidemiol. 2020;52:109.

627. Zhuang P, Jiao J, Wang W, Chen J. Association of monounsaturated fatty acids intake with mortality in China and United States. Ann Nutr Metab. 2017;71:765-6.

628. Zhuang P, Mao L, Wu F, Wang J, Jiao J, Zhang Y. Cooking Oil Consumption Is Positively Associated with Risk of Type 2 Diabetes in a Chinese Nationwide Cohort Study. J Nutr. 2020;150(7):1799-807.

629. Zhuang P, Wang W, Wang J, Zhang Y, Jiao J. Polyunsaturated fatty acids intake, omega-6/omega-3 ratio and mortality: Findings from two independent nationwide cohorts. Clin Nutr. 2019;38(2):848-55.

630. Zhuang X, Zhang S, Zhou H, Du Z, Liao X. U-SHAPED RELATIONSHIP BETWEEN CARBOHYDRATE INTAKE PROPORTION AND INCIDENT ATRIAL FIBRILLATION. JACC 2019;73(9, Supplement 2):4.

631. Villegas R, Xiang Y-B, Elasy T, Li H-L, Yang G, Cai H, et al. Fish, shellfish, and long-chain n−3 fatty acid consumption and risk of incident type 2 diabetes in middle-aged Chinese men and women. The American Journal of Clinical Nutrition. 2011;94(2):543-51.

632. Ghamarzad Shishavan N, Masoudi S, Mohamadkhani A, Sepanlou SG, Sharafkhah M, Poustchi H, et al. Dietary intake of fatty acids and risk of pancreatic cancer: Golestan cohort study. Nutr J. 2021;20(1):69.

633. Amiano P, Machón M, Dorronsoro M, Dolores Chirlaque M, Barricarte A, Sánchez MJ, et al. Intake of total omega-3 fatty acids, eicosapentaenoic acid and docosahexaenoic acid and risk of coronary heart disease in the Spanish EPIC cohort study. Nutr Metab Cardiovasc Dis. 2014;24(3):321-7.

634. Ahn S, Kim SH, Chung J, Park Y, Joung H. Associations between polyunsaturated fatty acid intake and risk of dyslipidemia. Ann Nutr Metab. 2023;79.

635. Alessa H, Giovannucci E, Smith S, Willett W, Wu K, Hu F, Malik V. Food Forms and Sources of Fructose and Risk of Type 2 Diabetes Among US Women and Men. Curr dev nutr. 2023;7.

636. Aloraini G, McKeown N, Lichtenstein A, Das SK, Jacques P. Low-Carbohydrate Dietary Patterns Are Associated With Beneficial Long-Term Changes in Systemic Markers of Inflammation in the Framingham Offspring Cohort. Curr dev nutr. 2023;7.

637. Chen H, Leng X, Liu S, Zeng Z, Huang F, Huang R, et al. Association between dietary intake of omega-3 polyunsaturated fatty acids and all-cause and cardiovascular mortality among hypertensive adults: Results from NHANES 1999-2018. Clin Nutr. 2023;42(12).

638. Cho HJ, Shin WK, Song YJ, Lee JK, Kang D, Lee JE. Associations of carbohydrate quantity and quality with all-cause and cause-specific mortality in a prospective cohort study. Ann Nutr Metab. 2023;79.

639. Cui Z, Liu K, Meng H. Associations between Conventional and Emerging Indicators of Dietary Carbohydrate Quality and New-Onset Type 2 Diabetes Mellitus in Chinese Adults. Curr dev nutr. 2022;6.

640. Curtis AR, Livingstone KM, Daly RM, Abbott G, Kiss N. Dietary patterns linked to a lower odds of malnutrition are also associated with all-cause and cancer mortality in adults with cancer: A prospective analysis. Clin Nutr ESPEN. 2023;58.

641. Daftari G, Tehrani AN, Pashayee-khamene F, Karimi S, Ahmadzadeh S, Hekmatdoost A, et al. Dietary protein intake and mortality among survivors of liver cirrhosis: a prospective cohort study. BMC Gastroenterology. 2023;23(1).

642. Doan LN, Hu C, Zhang Z, Shannon J, Bobe G, Takata Y. Dairy product consumption and lung cancer risk: A prospective analysis. Clin Nutr ESPEN. 2023;57.

643. Dos Santos ALT, Weiss T, Duarte CK, Cantori GA, De Azevedo MJ, Gross JL, Zelmanovitz T. Association between nutritional recommendations for cardiovascular disease and a lower incidence of cardiac events in patients with type 2 diabetes. Diabetes. 2010.

644. Glenn A, Wang F, Manson J, Rimm E, Mukamal K, Qi S, et al. Dietary Plant to Animal Protein Ratio and Risk of Cardiovascular Disease in Three Prospective Cohort Studies. Curr dev nutr. 2023;7.

645. Hosseinpour-Niazi S, Bakhshi B, Mirmiran P, Gaeini Z, Hadaegh F, Azizi F. Effect of weight change on the association between overall and source of carbohydrate intake and risk of metabolic syndrome: Tehran lipid and glucose study. Nutr Metab. 2023;20(1).

646. Jin D, Lu Y, Wu W, Jiang F, Li Z, Xu L, et al. Diet-Wide Association, Genetic Susceptibility and Colorectal Cancer Risk: A Prospective Cohort Study. Nutrients. 2023;15(22).

647. Korat AA, Duscova E, Shea K, Jacques P, Wang M, Sebastiani P, et al. Dietary Carbohydrate Intake and Carbohydrate Quality in Relation to Healthy Aging in Women. Curr dev nutr. 2023;7.

648. Kurata H, Meguro S, Abe Y, Sasaki T, Asakura K, Arai Y, Itoh H. Dietary protein intake and all-cause mortality: results from The Kawasaki Aging and Wellbeing Project. BMC geriatrics. 2023;23(1).

649. Morimoto N, Urayama KY, Tanaka A, Ai M. Relationship between dietary fiber to carbohydrate ratio and mortality risk in US adults: NHANES 2007-2018. Nutr Metab Cardiovasc Dis. 2023.

650. Park K, Jo U. Carbohydrate-Based Diet May Increase the Risk of Hypertriglyceridemia and Hypo-HDL Cholesterolemia. Curr dev nutr. 2023;7.

651. Schaefer SM, Kaiser A, Eichner G, Fasshauer M. Association of sugar intake from different sources with incident dementia in the prospective cohort of UK Biobank participants. Nutr J. 2023;22(1).

652. Weir NL, Nomura SO, Guan W, Garg PK, Allison M, Misialek JR, et al. Omega-3 Polyunsaturated Fatty Acids are not associated with Peripheral Artery Disease in a Meta-Analysis from the Multi-Ethnic Study of Atherosclerosis and Atherosclerosis Risk in Communities Study Cohorts. J Nutr. 2024;154(1).

653. Wendeu-Foyet G, Bellicha A, Chajes V, Huybrechts I, Debras C, Srour B, et al. Trans fatty acid intake and risk of type 2 diabetes: findings from the NutriNet-Santé prospective cohort. Ann Nutr Metab. 2023;79.

654. Zhang H, Zhang X, Yang H, Yang H, Zhang T, Chen L, et al. Dietary carbohydrate types, genetic predisposition, and risk of adult-onset asthma: A longitudinal cohort study. Int J Biol Macromol. 2024;261(Pt 1).

655. Zhang Y, Chen H, Lim CCW, Carrillo-Larco RM, Yan LL, Mishra GD, et al. Intake of sugary beverages with chronic conditions and multimorbidity: A prospective cohort study of UK Biobank. Int J Epidemiol. 2023;52(5).

656. Zhu L, Hu Y, Wang M, Rimm E, Hu F, Sun Q. Low-Carbohydrate Diets and Risk of Coronary Heart Disease in U.S. Men and Women. Curr dev nutr. 2023;7.

657. Agarwal P, Ford CN, Leurgans SE, Beck T, Desai P, Dhana K, et al. Dietary Sugar Intake Associated with a Higher Risk of Dementia in Community-Dwelling Older Adults. J Alzheimers Dis. 2023;95:1417-25.

658. McGee DL, Reed DM, Yano K, Kagan A, Tillotson J. Ten-year incidence of coronary heart disease in the Honolulu Heart Program. Relationship to nutrient intake. Am J Epidemiol. 1984;119(5):667-76.

659. Mohan V, Radhika G, Sathya RM, Tamil SR, Ganesan A, Sudha V. Dietary carbohydrates, glycaemic load, food groups and newly detected type 2 diabetes among urban Asian Indian population in Chennai, India (Chennai Urban Rural Epidemiology Study 59). Br J Nutr. 2009;102(10):1498-506.

660. Atkinson C, Whitley E, Ness A, Baker I. Associations between types of dietary fat and fish intake and risk of stroke in the Caerphilly Prospective Study (CaPS). Public Health. 2011;125(6):345-8.

661. Goldbourt U, Yaari S, Medalie JH. Factors predictive of long-term coronary heart disease mortality among 10,059 male Israeli civil servants and municipal employees. A 23-year mortality follow-up in the Israeli Ischemic Heart Disease Study. Cardiology. 1993;82(2-3):100-21.

662. Ascherio A, Rimm EB, Giovannucci EL, Spiegelman D, Stampfer M, Willett WC. Dietary fat and risk of coronary heart disease in men: cohort follow up study in the United States. Bmj. 1996;313(7049):84-90.

663. Pietinen P, Ascherio A, Korhonen P, Hartman AM, Willett WC, Albanes D, Virtamo J. Intake of fatty acids and risk of coronary heart disease in a cohort of Finnish men. The Alpha-Tocopherol, Beta-Carotene Cancer Prevention Study. Am J Epidemiol. 1997;145(10):876-87.

664. Merritt MA, Tzoulaki I, Tworoger SS, De Vivo I, Hankinson SE, Fernandes J, et al. Investigation of dietary factors and endometrial cancer risk using a nutrient-wide association study approach in the EPIC and Nurses' Health Study (NHS) and NHSII. CEBP. 2015;24(2):466-71.

665. Furberg AS, Thune I. Metabolic abnormalities (hypertension, hyperglycemia and overweight), lifestyle (high energy intake and physical inactivity) and endometrial cancer risk in a Norwegian cohort. Int J Cancer. 2003;104(6):669-76.

666. Jain MG, Rohan TE, Howe GR, Miller AB. A cohort study of nutritional factors and endometrial cancer. Eur J Epidemiol. 2000;16(10):899-905.

667. Yeh M, Moysich KB, Jayaprakash V, Rodabaugh KJ, Graham S, Brasure JR, McCann SE. Higher intakes of vegetables and vegetable-related nutrients are associated with lower endometrial cancer risks. J Nutr. 2009;139(2):317-22.

668. Wallström P, Bjartell A, Gullberg B, Olsson H, Wirfält E. A prospective study on dietary fat and incidence of prostate cancer (Malmö, Sweden). CCC. 2007;18(10):1107-21.

669. Wakai K, Tamakoshi K, Date C, Fukui M, Suzuki S, Lin Y, et al. Dietary intakes of fat and fatty acids and risk of breast cancer: a prospective study in Japan. Cancer Sci. 2005;96(9):590-9.

670. Sluijs I, Praagman J, Boer JMA, Verschuren WMM, van der Schouw YT. Fluidity of the dietary fatty acid profile and risk of coronary heart disease and ischemic stroke: Results from the EPIC-Netherlands cohort study. Nutr Metab Cardiovasc Dis. 2017;27(9):799-805.

671. Järvinen R, Knekt P, Hakulinen T, Rissanen H, Heliövaara M. Dietary fat, cholesterol and colorectal cancer in a prospective study. Br J Cancer. 2001;85(3):357-61.

672. Oba S, Shimizu N, Nagata C, Shimizu H, Kametani M, Takeyama N, et al. The relationship between the consumption of meat, fat, and coffee and the risk of colon cancer: a prospective study in Japan. Cancer Lett. 2006;244(2):260-7.

673. Butler LM, Wang R, Koh WP, Stern MC, Yuan JM, Yu MC. Marine n-3 and saturated fatty acids in relation to risk of colorectal cancer in Singapore Chinese: a prospective study. Int J Cancer. 2009;124(3):678-86.

674. Murff HJ, Shu X-O, Li H, Dai Q, Kallianpur A, Yang G, et al. A prospective study of dietary polyunsaturated fatty acids and colorectal cancer risk in Chinese women. CEBP. 2009;18(8):2283-91.

675. van Dam RM, Willett WC, Rimm EB, Stampfer MJ, Hu FB. Dietary fat and meat intake in relation to risk of type 2 diabetes in men. Diabetes Care. 2002;25(3):417-24.

676. Nagata C, Takatsuka N, Shimizu H. Soy and fish oil intake and mortality in a Japanese community. Am J Epidemiol. 2002;156(9):824-31.

677. Folsom AR, Demissie Z. Fish intake, marine omega-3 fatty acids, and mortality in a cohort of postmenopausal women. Am J Epidemiol. 2004;160(10):1005-10.

678. Albert CM, Oh K, Whang W, Manson JE, Chae CU, Stampfer MJ, et al. Dietary alpha-linolenic acid intake and risk of sudden cardiac death and coronary heart disease. Circulation. 2005;112(21):3232-8.

679. Brasky TM, Neuhouser ML, Cohn DE, White E. Associations of long-chain ω-3 fatty acids and fish intake with endometrial cancer risk in the VITamins And Lifestyle cohort. Am J Clin Nutr. 2014;99(3):599-608.

680. Cho E, Spiegelman D, Hunter DJ, Chen WY, Colditz GA, Willett WC. Premenopausal dietary carbohydrate, glycemic index, glycemic load, and fiber in relation to risk of breast cancer. CEBP. 2003;12(11 Pt 1):1153-8.

681. Hsieh LJ, Carter HB, Landis PK, Tucker KL, Metter EJ, Newschaffer CJ, Platz EA. Association of energy intake with prostate cancer in a long-term aging study: Baltimore Longitudinal Study of Aging (United States). Urology. 2003;61(2):297-301.

682. Heinen MM, Verhage BA, Goldbohm RA, van den Brandt PA. Meat and fat intake and pancreatic cancer risk in the Netherlands Cohort Study. Int J Cancer. 2009;125(5):1118-26.

683. Harding AH, Day NE, Khaw KT, Bingham S, Luben R, Welsh A, Wareham NJ. Dietary fat and the risk of clinical type 2 diabetes: the European prospective investigation of Cancer-Norfolk study. Am J Epidemiol. 2004;159(1):73-82.

684. Oomen CM, Ocké MC, Feskens EJ, van Erp-Baart MA, Kok FJ, Kromhout D. Association between trans fatty acid intake and 10-year risk of coronary heart disease in the Zutphen Elderly Study: a prospective population-based study. Lancet. 2001;357(9258):746-51.

685. Hu FB, Stampfer MJ, Manson JE, Ascherio A, Colditz GA, Speizer FE, et al. Dietary saturated fats and their food sources in relation to the risk of coronary heart disease in women. Am J Clin Nutr. 1999;70(6):1001-8.

686. Meinhold CL, Berrington de Gonzalez A, Albanes D, Weinstein SJ, Taylor PR, Virtamo J, Stolzenberg-Solomon RZ. Predictors of fasting serum insulin and glucose and the risk of pancreatic cancer in smokers. CCC. 2009;20(5):681-90.

687. Song Y, Manson JE, Buring JE, Liu S. A prospective study of red meat consumption and type 2 diabetes in middle-aged and elderly women: the women's health study. Diabetes Care. 2004;27(9):2108-15.

688. Alhazmi A, Stojanovski E, McEvoy M, Garg ML. Macronutrient intake and type 2 diabetes risk in middle-aged Australian women. Results from the Australian Longitudinal Study on Women's Health. Public Health Nutr. 2014;17(7):1587-94.

689. Voorrips LE, Brants HA, Kardinaal AF, Hiddink GJ, van den Brandt PA, Goldbohm RA. Intake of conjugated linoleic acid, fat, and other fatty acids in relation to postmenopausal breast cancer: the Netherlands Cohort Study on Diet and Cancer. Am J Clin Nutr. 2002;76(4):873-82.

690. van Dam RM, Visscher AW, Feskens EJ, Verhoef P, Kromhout D. Dietary glycemic index in relation to metabolic risk factors and incidence of coronary heart disease: the Zutphen Elderly Study. Eur J Clin Nutr. 2000;54(9):726-31.

691. Jonas CR, McCullough ML, Teras LR, Walker-Thurmond KA, Thun MJ, Calle EE. Dietary glycemic index, glycemic load, and risk of incident breast cancer in postmenopausal women. CEBP. 2003;12(6):573-7.

692. Higginbotham S, Zhang ZF, Lee IM, Cook NR, Buring JE, Liu S. Dietary glycemic load and breast cancer risk in the Women's Health Study. CEBP. 2004;13(1):65-70.

693. Holmes MD, Liu S, Hankinson SE, Colditz GA, Hunter DJ, Willett WC. Dietary carbohydrates, fiber, and breast cancer risk. Am J Epidemiol. 2004;159(8):732-9.

694. Giles GG, Simpson JA, English DR, Hodge AM, Gertig DM, Macinnis RJ, Hopper JL. Dietary carbohydrate, fibre, glycaemic index, glycaemic load and the risk of postmenopausal breast cancer. Int J Cancer. 2006;118(7):1843-7.

695. Terry PD, Jain M, Miller AB, Howe GR, Rohan TE. Glycemic load, carbohydrate intake, and risk of colorectal cancer in women: a prospective cohort study. J Natl Cancer Inst. 2003;95(12):914-6.

696. McCarl M, Harnack L, Limburg PJ, Anderson KE, Folsom AR. Incidence of colorectal cancer in relation to glycemic index and load in a cohort of women. CEBP. 2006;15(5):892-6.

697. Larsson SC, Giovannucci E, Wolk A. Dietary carbohydrate, glycemic index, and glycemic load in relation to risk of colorectal cancer in women. Am J Epidemiol. 2007;165(3):256-61.

698. Larsson SC, Bergkvist L, Wolk A. Glycemic load, glycemic index and carbohydrate intake in relation to risk of stomach cancer: a prospective study. Int J Cancer. 2006;118(12):3167-9.

699. Larsson SC, Friberg E, Wolk A. Carbohydrate intake, glycemic index and glycemic load in relation to risk of endometrial cancer: A prospective study of Swedish women. Int J Cancer. 2007;120(5):1103-7.

700. Johnson KJ, Anderson KE, Harnack L, Hong CP, Folsom AR. No association between dietary glycemic index or load and pancreatic cancer incidence in postmenopausal women. CEBP. 2005;14(6):1574-5.

701. Tsai CJ, Leitzmann MF, Willett WC, Giovannucci EL. Dietary carbohydrates and glycaemic load and the incidence of symptomatic gall stone disease in men. Gut. 2005;54(6):823-8.

702. Kaushik M, Mozaffarian D, Spiegelman D, Manson JE, Willett WC, Hu FB. Long-chain omega-3 fatty acids, fish intake, and the risk of type 2 diabetes mellitus. Am J Clin Nutr. 2009;90(3):613-20.

703. Devine A, Dick IM, Islam AF, Dhaliwal SS, Prince RL. Protein consumption is an important predictor of lower limb bone mass in elderly women. Am J Clin Nutr. 2005;81(6):1423-8.

704. Hannan MT, Tucker KL, Dawson-Hughes B, Cupples LA, Felson DT, Kiel DP. Effect of dietary protein on bone loss in elderly men and women: the Framingham Osteoporosis Study. J Bone Miner Res. 2000;15(12):2504-12.

705. Langsetmo L, Barr SI, Berger C, Kreiger N, Rahme E, Adachi JD, et al. Associations of Protein Intake and Protein Source with Bone Mineral Density and Fracture Risk: A Population-Based Cohort Study. J Nutr Health Aging. 2015;19(8):861-8.

706. Meng X, Zhu K, Devine A, Kerr DA, Binns CW, Prince RL. A 5-year cohort study of the effects of high protein intake on lean mass and BMC in elderly postmenopausal women. J Bone Miner Res. 2009;24(11):1827-34.

707. Misra D, Berry SD, Broe KE, McLean RR, Cupples LA, Tucker KL, et al. Does dietary protein reduce hip fracture risk in elders? The Framingham Osteoporosis Study. Osteoporos Int. 2011;22(1):345-9.

708. Munger RG, Cerhan JR, Chiu BC. Prospective study of dietary protein intake and risk of hip fracture in postmenopausal women. Am J Clin Nutr. 1999;69(1):147-52.

709. Mussolino ME, Looker AC, Madans JH, Langlois JA, Orwoll ES. Risk factors for hip fracture in white men: the NHANES I Epidemiologic Follow-up Study. J Bone Miner Res. 1998;13(6):918-24.

710. Rapuri PB, Gallagher JC, Haynatzka V. Protein intake: effects on bone mineral density and the rate of bone loss in elderly women. Am J Clin Nutr. 2003;77(6):1517-25.

711. Sahni S, Broe KE, Tucker KL, McLean RR, Kiel DP, Cupples LA, Hannan MT. Association of total protein intake with bone mineral density and bone loss in men and women from the Framingham Offspring Study. Public Health Nutr. 2014;17(11):2570-6.

712. Salmerón J, Manson JE, Stampfer MJ, Colditz GA, Wing AL, Willett WC. Dietary fiber, glycemic load, and risk of non-insulin-dependent diabetes mellitus in women. Jama. 1997;277(6):472-7.

713. Alonso A, Beunza JJ, Bes-Rastrollo M, Pajares RM, Martínez-González MA. Vegetable protein and fiber from cereal are inversely associated with the risk of hypertension in a Spanish cohort. Arch Med Res. 2006;37(6):778-86.

714. Fehily AM, Yarnell JW, Sweetnam PM, Elwood PC. Diet and incident ischaemic heart disease: the Caerphilly Study. Br J Nutr. 1993;69(2):303-14.

715. Mann JI, Appleby PN, Key TJ, Thorogood M. Dietary determinants of ischaemic heart disease in health conscious individuals. Heart. 1997;78(5):450-5.

716. Howe GR, Friedenreich CM, Jain M, Miller AB. A cohort study of fat intake and risk of breast cancer. J Natl Cancer Inst. 1991;83(5):336-40.

717. Tinker LF, Sarto GE, Howard BV, Huang Y, Neuhouser ML, Mossavar-Rahmani Y, et al. Biomarker-calibrated dietary energy and protein intake associations with diabetes risk among postmenopausal women from the Women's Health Initiative. Am J Clin Nutr. 2011;94(6):1600-6.

718. Nagata C, Wada K, Yamakawa M, Konishi K, Goto Y, Koda S, et al. Intake of starch and sugars and total and cause-specific mortality in a Japanese community: the Takayama Study. Br J Nutr. 2019;122(7):820-8.

719. O'Neil A, Shivappa N, Jacka FN, Kotowicz MA, Kibbey K, Hebert JR, Pasco JA. Pro-inflammatory dietary intake as a risk factor for CVD in men: a 5-year longitudinal study. Br J Nutr. 2015;114(12):2074-82.

720. Meisinger C, Rospleszcz S, Wintermeyer E, Lorbeer R, Thorand B, Bamberg F, et al. Isocaloric Substitution of Dietary Carbohydrate Intake with Fat Intake and MRI-Determined Total Volumes of Visceral, Subcutaneous and Hepatic Fat Content in Middle-Aged Adults. Nutrients. 2019;11(5):1151.

721. San-Cristobal R, Navas-Carretero S, Celis-Morales C, Livingstone K, Macready A, Fallaize R, et al. Differences in macronutrient profile across seven European countries (Food4Me study) and isocaloric substitution effects on BMI. Obes Facts. 2016;9:129-30.

722. Zheng M, Allman-Farinelli M, Heitmann BL, Rangan A. Substitution of Sugar-Sweetened Beverages with Other Beverage Alternatives: A Review of Long-Term Health Outcomes. J Acad Nutr Diet. 2015;115(5):767-79.

723. Faerch K, Lau C, Tetens I, Pedersen OB, Jørgensen T, Borch-Johnsen K, Glümer C. A statistical approach based on substitution of macronutrients provides additional information to models analyzing single dietary factors in relation to type 2 diabetes in danish adults: the Inter99 study. J Nutr. 2005;135(5):1177-82.

724. Li N, Petrick JL, Steck SE, Bradshaw PT, McClain KM, Niehoff NM, et al. A pooled analysis of dietary sugar/carbohydrate intake and esophageal and gastric cardia adenocarcinoma incidence and survival in the USA. Int J Epidemiol. 2017;46(6):1836-46.

725. Lubin F, Chetrit A, Modan B, Freedman LS. Dietary intake changes and their association with ovarian cancer risk. J Nutr. 2006;136(9):2362-7.

726. Mozaffarian D, Abdollahi M, Campos H, Houshiarrad A, Willett WC. Consumption of trans fats and estimated effects on coronary heart disease in Iran. Eur J Clin Nutr. 2007;61(8):1004-10.

727. Sekgala MD, Opperman M, Mpahleni B, Mchiza ZJ-R. Association between Macronutrient and Fatty Acid Consumption and Metabolic Syndrome: A South African Taxi Driver Survey. Int J Environ Res Public Health. 2022;19(23):15452.

728. Smith JD, Hou T, Ludwig DS, Rimm EB, Willett W, Hu FB, Mozaffarian D. Changes in intake of protein foods, carbohydrate amount and quality, and long-term weight change: results from 3 prospective cohorts. The American Journal of Clinical Nutrition. 2015;101(6):1216-24.

729. Hu C, Huang R, Li R, Ning N, He Y, Zhang J, et al. Low-Carbohydrate and Low-Fat Diet with Metabolic-Dysfunction-Associated Fatty Liver Disease. Nutrients. 2023;15(22).

730. Agnoli C, Sieri S, Ricceri F, Macciotta A, Masala G, Bendinelli B, et al. Macronutrient composition of the diet and long-term changes in weight and waist circumference in the EPIC–Italy cohort. Nutr Metab Cardiovasc Dis. 2021;31(1):67-75.

731. Ahmadi-Abhari S, Luben RN, Powell N, Bhaniani A, Chowdhury R, Wareham NJ, et al. Dietary intake of carbohydrates and risk of type 2 diabetes: the European Prospective Investigation into Cancer-Norfolk study. Br J Nutr. 2014;111(2):342-52.

732. Altorf-van der Kuil W, Engberink MF, van Rooij FJ, Hofman A, van't Veer P, Witteman JC, Geleijnse JM. Dietary protein and risk of hypertension in a Dutch older population: the Rotterdam study. J Hypertens. 2010;28(12):2394-400.

733. Altorf-van der Kuil W, Engberink MF, Geleijnse JM, Boer JM, Monique Verschuren WM. Sources of dietary protein and risk of hypertension in a general Dutch population. Br J Nutr. 2012;108(10):1897-903.

734. Ankarfeldt MZ, Angquist L, Jakobsen MU, Overvad K, Tjønneland A, Halkjaer J, et al. Interactions of dietary protein and adiposity measures in relation to subsequent changes in body weight and waist circumference. Obesity (Silver Spring). 2014;22(9):2097-103.

735. Ankarfeldt MZ, Larsen SC, Ängquist L, Husemoen LL, Roswall N, Overvad K, et al. Interaction between genetic predisposition to adiposity and dietary protein in relation to subsequent change in body weight and waist circumference. PLOS ONE. 2014;9(10):e110890.

736. Ankarfeldt MZ, Gottliebsen K, Ängquist L, Astrup A, Heitmann BL, Sørensen TIA. Dietary protein and urinary nitrogen in relation to 6-year changes in fat mass and fat-free mass. Int J Obes. 2015;39(1):162-8.

737. Arias-Fernández L, Struijk EA, Rodríguez-Artalejo F, Lopez-Garcia E, Lana A. Habitual dietary fat intake and risk of muscle weakness and lower-extremity functional impairment in older adults: A prospective cohort study. Clin Nutr. 2020;39(12):3663-70.

738. Beulen Y, Martínez-González MA, Van de Rest O, Salas-Salvadó J, Sorlí JV, Gómez-Gracia E, et al. Quality of Dietary Fat Intake and Body Weight and Obesity in a Mediterranean Population: Secondary Analyses within the PREDIMED Trial. Nutrients [Internet]. 2018; 10(12).

739. Bjørnevik K, Chitnis T, Ascherio A, Munger KL. Polyunsaturated fatty acids and the risk of multiple sclerosis. Mult Scler J. 2017;23(14):1830-8.

740. Boeke CE, Eliassen AH, Chen WY, Cho E, Holmes MD, Rosner B, et al. Dietary fat intake in relation to lethal breast cancer in two large prospective cohort studies. Breast Cancer Res Treat. 2014;146(2):383-92.

741. Brostow DP, Odegaard AO, Koh WP, Duval S, Gross MD, Yuan JM, Pereira MA. Omega-3 fatty acids and incident type 2 diabetes: the Singapore Chinese Health Study. Am J Clin Nutr. 2011;94(2):520-6.

742. Buendia JR, Bradlee ML, Singer MR, Moore LL. Diets higher in protein predict lower high blood pressure risk in Framingham Offspring Study adults. Am J Hypertens. 2015;28(3):372-9.

743. Burger KNJ, Beulens JWJ, van der Schouw YT, Sluijs I, Spijkerman AMW, Sluik D, et al. Dietary Fiber, Carbohydrate Quality and Quantity, and Mortality Risk of Individuals with Diabetes Mellitus. PLOS ONE. 2012;7(8):e43127.

744. Campmans-Kuijpers MJE, Sluijs I, Nöthlings U, Freisling H, Overvad K, Weiderpass E, et al. Isocaloric substitution of carbohydrates with protein: the association with weight change and mortality among patients with type 2 diabetes. Cardiovasc Diabetol. 2015;14(1):39.

745. Campmans-Kuijpers MJ, Sluijs I, Nöthlings U, Freisling H, Overvad K, Boeing H, et al. The association of substituting carbohydrates with total fat and different types of fatty acids with mortality and weight change among diabetes patients. Clin Nutr. 2016;35(5):1096-102.

746. Cao Y-J, Wang H-J, Zhang B, Qi S-F, Mi Y-J, Pan X-B, et al. Associations of fat and carbohydrate intake with becoming overweight and obese: an 11-year longitudinal cohort study. Br J Nutr. 2020;124(7):715-28.

747. Chen H, Zhang SM, Hernán MA, Willett WC, Ascherio A. Dietary intakes of fat and risk of Parkinson's disease. Am J Epidemiol. 2003;157(11):1007-14.

748. Chen Z, Franco OH, Lamballais S, Ikram MA, Schoufour JD, Muka T, Voortman T. Associations of specific dietary protein with longitudinal insulin resistance, prediabetes and type 2 diabetes: The Rotterdam Study. Clin Nutr. 2020;39(1):242-9.

749. Chiuve SE, Rimm EB, Sandhu RK, Bernstein AM, Rexrode KM, Manson JE, et al. Dietary fat quality and risk of sudden cardiac death in women. Am J Clin Nutr. 2012;96(3):498-507.

750. Chiuve SE, Hu FB, Albert CM, Rexrode KM, Manson JE, Willett WC, Rimm EB. Abstract P358: Dietary Fat Quality and Risk of CHD in the 28 Year Follow-up of a Large Prospective Study of US Women. Circulation. 2012;125(suppl_10):AP358-AP.

751. Chiuve SE, Sandhu RK, Moorthy MV, Glynn RJ, Albert CM. Dietary Fat Intake Is Differentially Associated with Risk of Paroxysmal Compared with Sustained Atrial Fibrillation in Women. J Nutr. 2015;145(9):2092-101.

752. Cust AE, Slimani N, Kaaks R, van Bakel M, Biessy C, Ferrari P, et al. Dietary Carbohydrates, Glycemic Index, Glycemic Load, and Endometrial Cancer Risk within the European Prospective Investigation into Cancer and Nutrition Cohort. American Journal of Epidemiology. 2007;166(8):912-23.

753. Choi HK, Curhan G. Soft drinks, fructose consumption, and the risk of gout in men: prospective cohort study. BMJ. 2008;336(7639):309-12.

754. de Lau LM, Bornebroek M, Witteman JC, Hofman A, Koudstaal PJ, Breteler MM. Dietary fatty acids and the risk of Parkinson disease: the Rotterdam study. Neurology. 2005;64(12):2040-5.

755. Dennis KK, Wang F, Li Y, Manson JE, Rimm EB, Hu FB, et al. Associations of dietary sugar types with coronary heart disease risk: a prospective cohort study. The American Journal of Clinical Nutrition. 2023;118(5):1000-9.

756. Dierssen-Sotos T, Gómez-Acebo I, Gutiérrez-Ruiz N, Aragonés N, Amiano P, Molina de la Torre AJ, et al. Dietary Constituents: Relationship with Breast Cancer Prognostic (MCC-SPAIN Follow-Up). Int J Environ Res Public Health. 2021;18(1):84.

757. Dinesen PT, Joensen AM, Rix TA, Tjønneland A, Schmidt EB, Lundbye-Christensen S, Overvad K. Effect of Dietary Intake of Saturated Fatty Acids on the Development of Atrial Fibrillation and the Effect of Replacement of Saturated With Monounsaturated and Polyunsaturated Fatty Acids. The American Journal of Cardiology. 2017;120(7):1129-32.

758. Duarte-Salles T, Fedirko V, Stepien M, Aleksandrova K, Bamia C, Lagiou P, et al. Dietary fat, fat subtypes and hepatocellular carcinoma in a large European cohort. Int J Cancer. 2015;137(11):2715-28.

759. Ericson U, Sonestedt E, Gullberg B, Hellstrand S, Hindy G, Wirfält E, Orho-Melander M. High intakes of protein and processed meat associate with increased incidence of type 2 diabetes. Br J Nutr. 2013;109(6):1143-53.

760. Esfandiar Z, Hosseini-Esfahani F, Mirmiran P, Azizi F. The association of dietary macronutrients composition with the incidence of type 2 diabetes, using iso-energetic substitution models: Tehran Lipid and Glucose Study. Prim Care Diabetes. 2021;15(6):1080-5.

761. Esmeijer K, Geleijnse JM, de Fijter JW, Kromhout D, Hoogeveen EK. Dietary protein intake and kidney function decline after myocardial infarction: the Alpha Omega Cohort. Nephrol Dial Transplant. 2020;35(1):106-15.

762. Farvid MS, Cho E, Chen WY, Eliassen AH, Willett WC. Premenopausal dietary fat in relation to pre- and post-menopausal breast cancer. Breast Cancer Res Treat. 2014;145(1):255-65.

763. Farvid MS, Ding M, Pan A, Sun Q, Chiuve SE, Steffen LM, et al. Dietary Linoleic Acid and Risk of Coronary Heart Disease: A Systematic Review and Meta-Analysis of Prospective Cohort Studies. Circulation. 2014;130(18):1568-78.

764. Farvid MS, Spence ND, Rosner BA, Barnett JB, Holmes MD. Associations of low-carbohydrate diets with breast cancer survival. Cancer. 2023;129(17):2694-704.

765. Gaeini Z, Mirmiran P, Bahadoran Z, Aghayan M, Azizi F. The association between dietary fats and the incidence risk of cardiovascular outcomes: Tehran Lipid and Glucose Study. Nutr Metab. 2021;18(1):96.

766. Genkinger JM, Hunter DJ, Spiegelman D, Anderson KE, Beeson WL, Buring JE, et al. A pooled analysis of 12 cohort studies of dietary fat, cholesterol and egg intake and ovarian cancer. CCC. 2006;17(3):273-85.

767. Gkotzamanis V, Panagiotakos D, Yannakoulia M, Kosmidis M, Dardiotis E, Hadjigeorgiou G, et al. Trajectories of healthy aging and their association with the Mediterranean diet: The HELIAD Study. Maturitas. 2022;159:33-9.

768. Guardino ET, Li Y, Nguyen X-M, Wilson PWF, Gaziano JM, Cho K, et al. Dietary ω-3 fatty acids and the incidence of atrial fibrillation in the Million Veteran Program. The American Journal of Clinical Nutrition. 2023;118(2):406-11.

769. Guasch-Ferré M, Becerra-Tomás N, Ruiz-Canela M, Corella D, Schröder H, Estruch R, et al. Total and subtypes of dietary fat intake and risk of type 2 diabetes mellitus in the Prevención con Dieta Mediterránea (PREDIMED) study. Am J Clin Nutr. 2017;105(3):723-35.

770. Ha K, Joung H, Song Y. Inadequate fat or carbohydrate intake was associated with an increased incidence of type 2 diabetes mellitus in Korean adults: A 12-year community-based prospective cohort study. Diabetes Res Clin Pract. 2019;148:254-61.

771. Hantikainen E, Roos E, Bellocco R, D'Antonio A, Grotta A, Adami H-O, et al. Dietary fat intake and risk of Parkinson disease: results from the Swedish National March Cohort. Eur J Epidemiol. 2022;37(6):603-13.

772. Haring B, Gronroos N, Nettleton JA, Wyler von Ballmoos MC, Selvin E, Alonso A. Dietary Protein Intake and Coronary Heart Disease in a Large Community Based Cohort: Results from the Atherosclerosis Risk in Communities (ARIC) Study. PLOS ONE. 2014;9(10):e109552.

773. Haring B, Misialek JR, Rebholz CM, Petruski-Ivleva N, Gottesman RF, Mosley TH, Alonso A. Association of Dietary Protein Consumption With Incident Silent Cerebral Infarcts and Stroke. Stroke. 2015;46(12):3443-50.

774. Haring B, Selvin E, Liang M, Coresh J, Grams ME, Petruski-Ivleva N, et al. Dietary Protein Sources and Risk for Incident Chronic Kidney Disease: Results From the Atherosclerosis Risk in Communities (ARIC) Study. J Ren Nutr. 2017;27(4):233-42.

775. Haugsgjerd TR, Egeland GM, Nygård OK, Igland J, Sulo G, Lysne V, et al. Intake of carbohydrates and SFA and risk of CHD in middle-age adults: the Hordaland Health Study (HUSK). Public Health Nutr. 2022;25(3):634-48.

776. Higginbotham S, Zhang ZF, Lee IM, Cook NR, Giovannucci E, Buring JE, Liu S. Dietary glycemic load and risk of colorectal cancer in the Women's Health Study. J Natl Cancer Inst. 2004;96(3):229-33.

777. Holmes MD, Hunter DJ, Colditz GA, Stampfer MJ, Hankinson SE, Speizer FE, et al. Association of dietary intake of fat and fatty acids with risk of breast cancer. Jama. 1999;281(10):914-20.

778. Horikawa C, Yoshimura Y, Kamada C, Tanaka S, Tanaka S, Matsunaga S, et al. Is the Proportion of Carbohydrate Intake Associated with the Incidence of Diabetes Complications?—An Analysis of the Japan Diabetes Complications Study. Nutrients. 2017;9(2):113.

779. Hosseini-Esfahani F, Koochakpoor G, Tahmasebinejad Z, Khalili D, Mirmiran P, Azizi F. The association of dietary macronutrients composition with the incidence of cardiovascular disease, using iso-energetic substitution models: Tehran lipid and glucose study. Nutr Metab Cardiovasc Dis. 2020;30(12):2186-93.

780. Hosseini-Esfahani F, Koochakpoor G, Mirmiran P, Ebrahimof S, Azizi F. The association of dietary macronutrients with anthropometric changes, using iso-energetic substitution models: Tehran lipid and glucose study. Nutr Metab. 2019;16(1):83.

781. Houston DK, Ding J, Lee JS, Garcia M, Kanaya AM, Tylavsky FA, et al. Dietary fat and cholesterol and risk of cardiovascular disease in older adults: the Health ABC Study. Nutr Metab Cardiovasc Dis. 2011;21(6):430-7.

782. Hruby A, Sahni S, Bolster D, Jacques PF. Protein Intake and Functional Integrity in Aging: The Framingham Heart Study Offspring. J Gerontol A Biol Sci Med Sci. 2018;75(1):123-30.

783. Hu FB, Stampfer MJ, Manson JE, Rimm E, Colditz GA, Rosner BA, et al. Dietary fat intake and the risk of coronary heart disease in women. N Engl J Med. 1997;337(21):1491-9.

784. Hu FB, Stampfer MJ, Manson JE, Rimm E, Colditz GA, Speizer FE, et al. Dietary protein and risk of ischemic heart disease in women. Am J Clin Nutr. 1999;70(2):221-7.

785. Hu T, Rianon NJ, Nettleton JA, Hyder JA, He J, Steffen LM, et al. Protein intake and lumbar bone density: the Multi-Ethnic Study of Atherosclerosis (MESA). Br J Nutr. 2014;112(8):1384-92.

786. Hu Y, Liu G, Yu E, Wang B, Wittenbecher C, Manson JE, et al. Low-Carbohydrate Diet Scores and Mortality Among Adults With Incident Type 2 Diabetes. Diabetes Care. 2023;46(4):874-84.

787. Jakobsen MU, Overvad K, Dyerberg J, Schroll M, Heitmann BL. Dietary Fat and Risk of Coronary Heart Disease: Possible Effect Modification by Gender and Age. American Journal of Epidemiology. 2004;160(2):141-9.

788. Jakobsen MU, Overvad K, Dyerberg J, Heitmann BL. Intake of ruminant trans fatty acids and risk of coronary heart disease. Int J Epidemiol. 2007;37(1):173-82.

789. Jakobsen MU, O'Reilly EJ, Heitmann BL, Pereira MA, Bälter K, Fraser GE, et al. Major types of dietary fat and risk of coronary heart disease: a pooled analysis of 11 cohort studies. Am J Clin Nutr. 2009;89(5):1425-32.

790. Jakobsen MU, Dethlefsen C, Joensen AM, Stegger J, Tjønneland A, Schmidt EB, Overvad K. Intake of carbohydrates compared with intake of saturated fatty acids and risk of myocardial infarction: importance of the glycemic index. Am J Clin Nutr. 2010;91(6):1764-8.

791. Jiang YW, Sheng LT, Pan XF, Feng L, Yuan JM, Pan A, Koh WP. Midlife Dietary Intakes of Monounsaturated Acids, n-6 Polyunsaturated Acids, and Plant-Based Fat Are Inversely Associated with Risk of Cognitive Impairment in Older Singapore Chinese Adults. J Nutr. 2020;150(4):901-9.

792. Jiao J, Liu G, Shin HJ, Hu FB, Rimm EB, Rexrode KM, et al. Dietary fats and mortality among patients with type 2 diabetes: analysis in two population based cohort studies. BMJ. 2019;366:l4009.

793. Kang JH, Pasquale LR, Willett WC, Rosner BA, Egan KM, Faberowski N, Hankinson SE. Dietary fat consumption and primary open-angle glaucoma. Am J Clin Nutr. 2004;79(5):755-64.

794. Katagiri R, Yamaji T, Sawada N, Iwasaki M, Inoue M, Tsugane S. Total, animal, and plant protein intake and pneumonia mortality in the Japan Public Health Center-based Prospective Study. Am J Clin Nutr. 2022;115(3):781-9.

795. Kenfield SA, Richman EL, Sesso HD, Ma J, Stampfer MJ, Chavarro JE. Abstract 3617: Fat intake after prostate cancer diagnosis and risk of lethal prostate cancer and total mortality. Cancer Res. 2013;73(8_Supplement):3617-.

796. Kiage JN, Merrill PD, Judd SE, He K, Lipworth L, Cushman M, et al. Intake of trans fat and incidence of stroke in the REasons for Geographic And Racial Differences in Stroke (REGARDS) cohort. Am J Clin Nutr. 2014;99(5):1071-6.

797. Kim EHJ, Willett WC, Colditz GA, Hankinson SE, Stampfer MJ, Hunter DJ, et al. Dietary Fat and Risk of Postmenopausal Breast Cancer in a 20-year Follow-up. American Journal of Epidemiology. 2006;164(10):990-7.

798. Koh AS, Pan A, Wang R, Odegaard AO, Pereira MA, Yuan JM, Koh WP. The association between dietary omega-3 fatty acids and cardiovascular death: the Singapore Chinese Health Study. Eur J Prev Cardiol. 2015;22(3):364-72.

799. Koh W-P, Dan YY, Goh GB-B, Jin A, Wang R, Yuan J-M. Dietary fatty acids and risk of hepatocellular carcinoma in the Singapore Chinese health study. Liver Int. 2016;36(6):893-901.

800. Koh-Banerjee P, Chu NF, Spiegelman D, Rosner B, Colditz G, Willett W, Rimm E. Prospective study of the association of changes in dietary intake, physical activity, alcohol consumption, and smoking with 9-y gain in waist circumference among 16 587 US men. Am J Clin Nutr. 2003;78(4):719-27.

801. Larsson SC, Virtamo J, Wolk A. Dietary protein intake and risk of stroke in women. Atherosclerosis. 2012;224(1):247-51.

802. Larsson SC, Virtamo J, Wolk A. Dietary fats and dietary cholesterol and risk of stroke in women. Atherosclerosis. 2012;221(1):282-6.

803. Lee H-A, Park H. Substitution of Carbohydrates for Fats and Risk of Type 2 Diabetes among Korean Middle-Aged Adults: Findings from the Korean Genome and Epidemiology Study. Nutrients. 2022;14(3):654.

804. Lee JE, Spiegelman D, Hunter DJ, Albanes D, Bernstein L, van den Brandt PA, et al. Fat, Protein, and Meat Consumption and Renal Cell Cancer Risk: A Pooled Analysis of 13 Prospective Studies. JNCI. 2008;100(23):1695-706.

805. Levitan EB, Wolk A, Mittleman MA. Fish consumption, marine omega-3 fatty acids, and incidence of heart failure: a population-based prospective study of middle-aged and elderly men. Eur Heart J. 2009;30(12):1495-500.

806. Levitan EB, Wolk A, Mittleman MA. Fatty fish, marine omega-3 fatty acids and incidence of heart failure. Eur J Clin Nutr. 2010;64(6):587-94.

807. Li Y, Hruby A, Bernstein AM, Ley SH, Wang DD, Chiuve SE, et al. Saturated Fats Compared With Unsaturated Fats and Sources of Carbohydrates in Relation to Risk of Coronary Heart Disease: A Prospective Cohort Study. JACC. 2015;66(14):1538-48.

808. Li SX, Imamura F, Ye Z, Schulze MB, Zheng J, Ardanaz E, et al. Interaction between genes and macronutrient intake on the risk of developing type 2 diabetes: systematic review and findings from European Prospective Investigation into Cancer (EPIC)-InterAct. Am J Clin Nutr. 2017;106(1):263-75.

809. Li SX, Imamura F, Schulze MB, Zheng J, Ye Z, Agudo A, et al. Interplay between genetic predisposition, macronutrient intake and type 2 diabetes incidence: analysis within EPIC-InterAct across eight European countries. Diabetologia. 2018;61(6):1325-32.

810. Li J, Glenn AJ, Yang Q, Ding D, Zheng L, Bao W, et al. Dietary Protein Sources, Mediating Biomarkers, and Incidence of Type 2 Diabetes: Findings From the Women's Health Initiative and the UK Biobank. Diabetes Care. 2022;45(8):1742-53.

811. Liao LM, Loftfield E, Etemadi A, Graubard BI, Sinha R. Substitution of dietary protein sources in relation to colorectal cancer risk in the NIH-AARP cohort study. CCC. 2019;30(10):1127-35.

812. Lim CGY, Whitton C, Rebello SA, van Dam RM. Diet Quality and Lower Refined Grain Consumption are Associated With Less Weight Gain in a Multi-Ethnic Asian Adult Population. J Nutr. 2021;151(8):2372-82.

813. Lim CGY, Tai ES, van Dam RM. Replacing dietary carbohydrates and refined grains with different alternatives and risk of cardiovascular diseases in a multi-ethnic Asian population. The American Journal of Clinical Nutrition. 2022;115(3):854-63.

814. Liu X, Li Y, Tobias DK, Wang DD, Manson JE, Willett WC, Hu FB. Changes in Types of Dietary Fats Influence Long-term Weight Change in US Women and Men. J Nutr. 2018;148(11):1821-9.

815. Liu S, van der Schouw YT, Soedamah-Muthu SS, Spijkerman AMW, Sluijs I. Intake of dietary saturated fatty acids and risk of type 2 diabetes in the European Prospective Investigation into Cancer and Nutrition-Netherlands cohort: associations by types, sources of fatty acids and substitution by macronutrients. Eur J Nutr. 2019;58(3):1125-36.

816. Liu C, Meng Q, Zu C, Wei Y, Su X, Zhang Y, et al. Dietary low- and high-quality carbohydrate intake and cognitive decline: A prospective cohort study in older adults. Clin Nutr. 2023;42(8):1322-9.

817. Löf M, Sandin S, Lagiou P, Hilakivi-Clarke L, Trichopoulos D, Adami HO, Weiderpass E. Dietary fat and breast cancer risk in the Swedish women's lifestyle and health cohort. Br J Cancer. 2007;97(11):1570-6.

818. Lu B, Driban JB, Xu C, Lapane KL, McAlindon TE, Eaton CB. Dietary Fat Intake and Radiographic Progression of Knee Osteoarthritis: Data From the Osteoarthritis Initiative. Arthritis Care Res (Hoboken). 2017;69(3):368-75.

819. MacDonald CJ, Madkia A-L, Mounier-Vehier C, Severi G, Boutron-Ruault M-C. Associations between saturated fat intake and other dietary macronutrients and incident hypertension in a prospective study of French women. Eur J Nutr. 2023;62(3):1207-15.

820. Malik VS, Li Y, Tobias DK, Pan A, Hu FB. Dietary Protein Intake and Risk of Type 2 Diabetes in US Men and Women. Am J Epidemiol. 2016;183(8):715-28.

821. Merino J, Guasch-Ferré M, Ellervik C, Dashti HS, Sharp SJ, Wu P, et al. Quality of dietary fat and genetic risk of type 2 diabetes: individual participant data meta-analysis. BMJ. 2019;366:l4292.

822. Meyer KA, Kushi LH, Jacobs DR, Jr., Folsom AR. Dietary fat and incidence of type 2 diabetes in older Iowa women. Diabetes Care. 2001;24(9):1528-35.

823. Mölenberg FJM, de Goede J, Wanders AJ, Zock PL, Kromhout D, Geleijnse JM. Dietary fatty acid intake after myocardial infarction: a theoretical substitution analysis of the Alpha Omega Cohort. Am J Clin Nutr. 2017;106(3):895-901.

824. Montiel-Rojas D, Santoro A, Nilsson A, Franceschi C, Capri M, Bazzocchi A, et al. Beneficial Role of Replacing Dietary Saturated Fatty Acids with Polyunsaturated Fatty Acids in the Prevention of Sarcopenia: Findings from the NU-AGE Cohort. Nutrients. 2020;12(10):3079.

825. Moslehi N, Ehsani B, Mirmiran P, Hojjat P, Azizi F. Association of Dietary Proportions of Macronutrients with Visceral Adiposity Index: Non-Substitution and Iso-Energetic Substitution Models in a Prospective Study. Nutrients. 2015;7(10):8859-70.

826. Mozaffarian D, Rimm EB, Herrington DM. Dietary fats, carbohydrate, and progression of coronary atherosclerosis in postmenopausal women. Am J Clin Nutr. 2004;80(5):1175-84.

827. Mozaffarian D, Katan MB, Ascherio A, Stampfer MJ, Willett WC. Trans fatty acids and cardiovascular disease. N Engl J Med. 2006;354(15):1601-13.

828. Nimptsch K, Berg-Beckhoff G, Linseisen J. Effect of dietary fatty acid intake on prospective weight change in the Heidelberg cohort of the European Prospective Investigation into Cancer and Nutrition. Public Health Nutr. 2010;13(10):1636-46.

829. O'Doherty MG, Freedman ND, Hollenbeck AR, Schatzkin A, Murray LJ, Cantwell MM, Abnet CC. Association of dietary fat intakes with risk of esophageal and gastric cancer in the NIH-AARP diet and health study. Int J Cancer. 2012;131(6):1376-87.

830. Oh K, Hu FB, Manson JE, Stampfer MJ, Willett WC. Dietary Fat Intake and Risk of Coronary Heart Disease in Women: 20 Years of Follow-up of the Nurses' Health Study. American Journal of Epidemiology. 2005;161(7):672-9.

831. Praagman J, de Jonge EAL, Kiefte-de Jong JC, Beulens JWJ, Sluijs I, Schoufour JD, et al. Dietary Saturated Fatty Acids and Coronary Heart Disease Risk in a Dutch Middle-Aged and Elderly Population. ATVB. 2016;36(9):2011-8.

832. Praagman J, Beulens JW, Alssema M, Zock PL, Wanders AJ, Sluijs I, van der Schouw YT. The association between dietary saturated fatty acids and ischemic heart disease depends on the type and source of fatty acid in the European Prospective Investigation into Cancer and Nutrition-Netherlands cohort. Am J Clin Nutr. 2016;103(2):356-65.

833. Praagman J, Vissers LET, Mulligan AA, Laursen ASD, Beulens JWJ, van der Schouw YT, et al. Consumption of individual saturated fatty acids and the risk of myocardial infarction in a UK and a Danish cohort. Int J Cardiol. 2019;279:18-26.

834. Preis SR, Stampfer MJ, Spiegelman D, Willett WC, Rimm EB. Lack of association between dietary protein intake and risk of stroke among middle-aged men. Am J Clin Nutr. 2010;91(1):39-45.

835. Preis SR, Stampfer MJ, Spiegelman D, Willett WC, Rimm EB. Dietary protein and risk of ischemic heart disease in middle-aged men. Am J Clin Nutr. 2010;92(5):1265-72.

836. Qian F, Zong G, Li Y, Sun Q. Abstract MP43: Monounsaturated Fatty Acids From Plant or Animal Sources and Risk of Type 2 Diabetes in Three Large Prospective Cohorts of Men and Women. Circulation. 2019;139(Suppl_1):AMP43-AMP.

837. Richman EL, Kenfield SA, Chavarro JE, Stampfer MJ, Giovannucci EL, Willett WC, Chan JM. Fat intake after diagnosis and risk of lethal prostate cancer and all-cause mortality. JAMA Intern Med. 2013;173(14):1318-26.

838. Salmerón J, Hu FB, Manson JE, Stampfer MJ, Colditz GA, Rimm EB, Willett WC. Dietary fat intake and risk of type 2 diabetes in women. Am J Clin Nutr. 2001;73(6):1019-26.

839. Santiago S, Zazpe I, Gea A, NuÃ±ez-CÃ³rdoba JM, Carlos S, Bes-Rastrollo M, MartÃnez-GonzÃ¡lez MA. Fat Quality Index and Risk of Cardiovascular Disease in the Sun Project. J Nutr Health Aging. 2018;22(4):526-33.

840. Schoenaker DAJM, Toeller M, Chaturvedi N, Fuller JH, Soedamah-Muthu SS, the EPCSG. Dietary saturated fat and fibre and risk of cardiovascular disease and all-cause mortality among type 1 diabetic patients: the EURODIAB Prospective Complications Study. Diabetologia. 2012;55(8):2132-41.

841. Schulze MB, Schulz M, Heidemann C, Schienkiewitz A, Hoffmann K, Boeing H. Carbohydrate intake and incidence of type 2 diabetes in the European Prospective Investigation into Cancer and Nutrition (EPIC)-Potsdam Study. Br J Nutr. 2008;99(5):1107-16.

842. Shang X, Hill E, Zhu Z, Liu J, Ge Z, Wang W, He M. Macronutrient Intake and Risk of Dementia in Community-Dwelling Older Adults: A Nine-Year Follow-Up Cohort Study. J Alzheimers Dis. 2022;85(2):791-804.

843. Shang X, Scott D, Hodge A, English DR, Giles GG, Ebeling PR, Sanders KM. Dietary protein from different food sources, incident metabolic syndrome and changes in its components: An 11-year longitudinal study in healthy community-dwelling adults. Clin Nutr. 2017;36(6):1540-8.

844. Shikany JM, Flood AP, Kitahara CM, Hsing AW, Meyer TE, Willcox BJ, et al. Dietary carbohydrate, glycemic index, glycemic load, and risk of prostate cancer in the Prostate, Lung, Colorectal, and Ovarian Cancer Screening Trial (PLCO) cohort. CCC. 2011;22(7):995-1002.

845. Sieri S, Brighenti F, Agnoli C, Grioni S, Masala G, Bendinelli B, et al. Dietary Glycemic Load and Glycemic Index and Risk of Cerebrovascular Disease in the EPICOR Cohort. PLOS ONE. 2013;8(5):e62625.

846. Sieri S, Agnoli C, Grioni S, Weiderpass E, Mattiello A, Sluijs I, et al. Glycemic index, glycemic load, and risk of coronary heart disease: a pan-European cohort study. Am J Clin Nutr. 2020;112(3):631-43.

847. Sieri S, Agnoli C, Pala V, Grioni S, Palli D, Bendinelli B, et al. Dietary Intakes of Animal and Plant Proteins and Risk of Colorectal Cancer: The EPIC-Italy Cohort. Cancers. 2022;14(12):2917.

848. Similä ME, Valsta LM, Kontto JP, Albanes D, Virtamo J. Low-, medium- and high-glycaemic index carbohydrates and risk of type 2 diabetes in men. Br J Nutr. 2011;105(8):1258-64.

849. Similä ME, Kontto JP, Valsta LM, Männistö S, Albanes D, Virtamo J. Carbohydrate substitution for fat or protein and risk of type 2 diabetes in male smokers. Eur J Clin Nutr. 2012;66(6):716-21.

850. Similä ME, Kontto JP, Männistö S, Valsta LM, Virtamo J. Glycaemic index, carbohydrate substitution for fat and risk of CHD in men. Br J Nutr. 2013;110(9):1704-11.

851. Sluijs I, Beulens JW, van der AD, Spijkerman AM, Grobbee DE, van der Schouw YT. Dietary intake of total, animal, and vegetable protein and risk of type 2 diabetes in the European Prospective Investigation into Cancer and Nutrition (EPIC)-NL study. Diabetes Care. 2010;33(1):43-8.

852. Sluijs I, van der Schouw YT, van der AD, Spijkerman AM, Hu FB, Grobbee DE, Beulens JW. Carbohydrate quantity and quality and risk of type 2 diabetes in the European Prospective Investigation into Cancer and Nutrition-Netherlands (EPIC-NL) study. Am J Clin Nutr. 2010;92(4):905-11.

853. Sluik D, Brouwer-Brolsma EM, Berendsen AAM, Mikkilä V, Poppitt SD, Silvestre MP, et al. Protein intake and the incidence of pre-diabetes and diabetes in 4 population-based studies: the PREVIEW project. Am J Clin Nutr. 2019;109(5):1310-8.

854. Smith-Warner SA, Spiegelman D, Adami HO, Beeson WL, van den Brandt PA, Folsom AR, et al. Types of dietary fat and breast cancer: a pooled analysis of cohort studies. Int J Cancer. 2001;92(5):767-74.

855. Smith-Warner SA, Ritz J, Hunter DJ, Albanes D, Beeson WL, van den Brandt PA, et al. Dietary Fat and Risk of Lung Cancer in a Pooled Analysis of Prospective Studies1. CEBP. 2002;11(10):987-92.

856. Song M, Wu K, Meyerhardt JA, Yilmaz O, Wang M, Ogino S, et al. Low-Carbohydrate Diet Score and Macronutrient Intake in Relation to Survival After Colorectal Cancer Diagnosis. JNCI Cancer Spectrum. 2019;2(4).

857. Steur M, Johnson L, Sharp SJ, Imamura F, Sluijs I, Key TJ, et al. Dietary Fatty Acids, Macronutrient Substitutions, Food Sources and Incidence of Coronary Heart Disease: Findings From the EPIC-CVD Case-Cohort Study Across Nine European Countries. J Am Heart Assoc. 2021;10(23):e019814.

858. Struijk EA, Fung TT, Rodríguez-Artalejo F, Bischoff-Ferrari HA, Hu FB, Willett WC, Lopez-Garcia E. Protein intake and risk of frailty among older women in the Nurses' Health Study. J Cachexia Sarcopenia and Muscle. 2022;13(3):1752-61.

859. Tanasescu M, Cho E, Manson JE, Hu FB. Dietary fat and cholesterol and the risk of cardiovascular disease among women with type 2 diabetes. Am J Clin Nutr. 2004;79(6):999-1005.

860. Taylor EN, Curhan GC. Fructose consumption and the risk of kidney stones. Kidney Int. 2008;73(2):207-12.

861. Thiébaut AC, Kipnis V, Chang SC, Subar AF, Thompson FE, Rosenberg PS, et al. Dietary fat and postmenopausal invasive breast cancer in the National Institutes of Health-AARP Diet and Health Study cohort. J Natl Cancer Inst. 2007;99(6):451-62.

862. Tsai CJ, Leitzmann MF, Willett WC, Giovannucci EL. Long-chain saturated fatty acids consumption and risk of gallstone disease among men. Ann Surg. 2008;247(1):95-103.

863. Van Blarigan EL, Kenfield SA, Yang M, Sesso HD, Ma J, Stampfer MJ, et al. Fat intake after prostate cancer diagnosis and mortality in the Physicians' Health Study. CCC. 2015;26(8):1117-26.

864. Van Blarigan EL, Ma C, Ou FS, Bainter TM, Venook AP, Ng K, et al. Dietary fat in relation to all-cause mortality and cancer progression and death among people with metastatic colorectal cancer: Data from CALGB 80405 (Alliance)/SWOG 80405. Int J Cancer. 2023;152(2):123-36.

865. van Nielen M, Feskens EJ, Mensink M, Sluijs I, Molina E, Amiano P, et al. Dietary protein intake and incidence of type 2 diabetes in Europe: the EPIC-InterAct Case-Cohort Study. Diabetes Care. 2014;37(7):1854-62.

866. Venø SK, Schmidt EB, Jakobsen MU, Lundbye-Christensen S, Bach FW, Overvad K. Substitution of Linoleic Acid for Other Macronutrients and the Risk of Ischemic Stroke. Stroke. 2017;48(12):3190-5.

867. Vergnaud A-C, Norat T, Mouw T, Romaguera D, May AM, Bueno-de-Mesquita HB, et al. Macronutrient Composition of the Diet and Prospective Weight Change in Participants of the EPIC-PANACEA Study. PLOS ONE. 2013;8(3):e57300.

868. Verspoor E, Voortman T, van Rooij FJA, Rivadeneira F, Franco OH, Kiefte-de Jong JC, Schoufour JD. Macronutrient intake and frailty: the Rotterdam Study. Eur J Nutr. 2020;59(7):2919-28.

869. Virtanen JK, Mursu J, Tuomainen T-P, Voutilainen S. Dietary Fatty Acids and Risk of Coronary Heart Disease in Men. ATVB. 2014;34(12):2679-87.

870. Virtanen HEK, Koskinen TT, Voutilainen S, Mursu J, Tuomainen TP, Kokko P, Virtanen JK. Intake of different dietary proteins and risk of type 2 diabetes in men: the Kuopio Ischaemic Heart Disease Risk Factor Study. Br J Nutr. 2017;117(6):882-93.

871. Virtanen HEK, Voutilainen S, Koskinen TT, Mursu J, Tuomainen T-P, Virtanen JK. Intake of Different Dietary Proteins and Risk of Heart Failure in Men. Circulation: Heart Failure. 2018;11(6):e004531.

872. Voortman T, Chen Z, Girschik C, Kavousi M, Franco OH, Braun KVE. Associations between macronutrient intake and coronary heart disease (CHD): The Rotterdam Study. Clin Nutr. 2021;40(11):5494-9.

873. Wan Z, Shan Z, Geng T, Lu Q, Li L, Yin J, et al. Associations of Moderate Low-Carbohydrate Diets With Mortality Among Patients With Type 2 Diabetes: A Prospective Cohort Study. J Clin Endocrinol Metab. 2022;107(7):e2702-e9.

874. Wan Y, Tobias DK, Dennis KK, Guasch-Ferré M, Sun Q, Rimm EB, et al. Association between changes in carbohydrate intake and long term weight changes: prospective cohort study. BMJ. 2023;382:e073939.

875. Wang SE, Hodge AM, Dashti SG, Dixon-Suen SC, Mitchell H, Thomas RJ, et al. Diet and risk of gastro-oesophageal reflux disease in the Melbourne Collaborative Cohort Study. Public Health Nutr. 2021;24(15):5034-46.

876. Xu J, Eilat-Adar S, Loria C, Goldbourt U, Howard BV, Fabsitz RR, et al. Dietary fat intake and risk of coronary heart disease: the Strong Heart Study. Am J Clin Nutr. 2006;84(4):894-902.

877. Yamagishi K, Iso H, Yatsuya H, Tanabe N, Date C, Kikuchi S, et al. Dietary intake of saturated fatty acids and mortality from cardiovascular disease in Japanese: the Japan Collaborative Cohort Study for Evaluation of Cancer Risk (JACC) Study. Am J Clin Nutr. 2010;92(4):759-65.

878. Yang JJ, Yu D, Takata Y, Smith-Warner SA, Blot W, White E, et al. Dietary Fat Intake and Lung Cancer Risk: A Pooled Analysis. J Clin Oncol. 2017;35(26):3055-64.

879. Yang T, Yi J, He Y, Zhang J, Li X, Ke S, et al. Associations of Dietary Fats with All-Cause Mortality and Cardiovascular Disease Mortality among Patients with Cardiometabolic Disease. Nutrients. 2022;14(17):3608.

880. Yeh T-S, Yuan C, Ascherio A, Rosner BA, Blacker D, Willett WC. Long-term dietary protein intake and subjective cognitive decline in US men and women. The American Journal of Clinical Nutrition. 2022;115(1):199-210.

881. Yeh T-S, Yuan C, Ascherio A, Rosner BA, Blacker D, Willett WC. Long-term intake of total energy and fat in relation to subjective cognitive decline. Eur J Epidemiol. 2022;37(2):133-46.

882. Yu D, Shu X-O, Li H, Xiang Y-B, Yang G, Gao Y-T, et al. Dietary Carbohydrates, Refined Grains, Glycemic Load, and Risk of Coronary Heart Disease in Chinese Adults. American Journal of Epidemiology. 2013;178(10):1542-9.

883. Zhou C, Wu Q, Ye Z, Liu M, Zhang Z, Zhang Y, et al. Inverse Association Between Variety of Proteins With Appropriate Quantity From Different Food Sources and New-Onset Hypertension. Hypertension. 2022;79(5):1017-27.

884. Zhou C, Liu C, Zhang Z, Liu M, Zhang Y, Li H, et al. Variety and quantity of dietary protein intake from different sources and risk of new-onset diabetes: a Nationwide Cohort Study in China. BMC Medicine. 2022;20(1):6.

885. Zhuang P, Zhang Y, Mao L, Wang L, Wu F, Cheng L, Jiao J. The association between consumption of monounsaturated fats from animal- v. plant-based foods and the risk of type 2 diabetes: a prospective nationwide cohort study. Br J Nutr. 2020;124(1):102-11.

886. Zong G, Liu G, Willett WC, Wanders AJ, Alssema M, Zock PL, et al. Associations Between Linoleic Acid Intake and Incident Type 2 Diabetes Among U.S. Men and Women. Diabetes Care. 2019;42(8):1406-13.

887. Zong G, Li Y, Wanders AJ, Alssema M, Zock PL, Willett WC, et al. Intake of individual saturated fatty acids and risk of coronary heart disease in US men and women: two prospective longitudinal cohort studies. BMJ. 2016;355:i5796.

888. Zong G, Li Y, Sampson L, Dougherty LW, Willett WC, Wanders AJ, et al. Monounsaturated fats from plant and animal sources in relation to risk of coronary heart disease among US men and women. Am J Clin Nutr. 2018;107(3):445-53.

889. Cho E, Spiegelman D, Hunter DJ, Chen WY, Stampfer MJ, Colditz GA, Willett WC. Premenopausal Fat Intake and Risk of Breast Cancer. JNCI. 2003;95(14):1079-85.

890. Leitzmann MF, Stampfer MJ, Michaud DS, Augustsson K, Colditz GC, Willett WC, Giovannucci EL. Dietary intake of n−3 and n−6 fatty acids and the risk of prostate cancer123. The American Journal of Clinical Nutrition. 2004;80(1):204-16.

891. Schulze MB, Liu S, Rimm EB, Manson JE, Willett WC, Hu FB. Glycemic index, glycemic load, and dietary fiber intake and incidence of type 2 diabetes in younger and middle-aged women. Am J Clin Nutr. 2004;80(2):348-56.

892. Rivera-Paredez B, León-Reyes G, Rangel-Marín D, Salmerón J, Velázquez-Cruz R. Associations between Macronutrients Intake and Bone Mineral Density: A Longitudinal Analysis of the Health Workers Cohort Study Participants. J Nutr Health Aging. 2023;27(12):1196-205.

893. Kristal AR, Arnold KB, Neuhouser ML, Goodman P, Platz EA, Albanes D, Thompson IM. Diet, supplement use, and prostate cancer risk: results from the prostate cancer prevention trial. Am J Epidemiol. 2010;172(5):566-77.

894. Iso H, Stampfer MJ, Manson JE, Rexrode K, Hu F, Hennekens CH, et al. Prospective study of fat and protein intake and risk of intraparenchymal hemorrhage in women. Circulation. 2001;103(6):856-63.

895. Kröger J, Zietemann V, Enzenbach C, Weikert C, Jansen EH, Döring F, et al. Erythrocyte membrane phospholipid fatty acids, desaturase activity, and dietary fatty acids in relation to risk of type 2 diabetes in the European Prospective Investigation into Cancer and Nutrition (EPIC)-Potsdam Study. Am J Clin Nutr. 2011;93(1):127-42.

896. Nanri A, Mizoue T, Kurotani K, Goto A, Oba S, Noda M, et al. Low-Carbohydrate Diet and Type 2 Diabetes Risk in Japanese Men and Women: The Japan Public Health Center-Based Prospective Study. PLOS ONE. 2015;10(2):e0118377.

897. Oh K, Hu FB, Cho E, Rexrode KM, Stampfer MJ, Manson JE, et al. Carbohydrate Intake, Glycemic Index, Glycemic Load, and Dietary Fiber in Relation to Risk of Stroke in Women. American Journal of Epidemiology. 2005;161(2):161-9.

898. Ortolá R, Struijk EA, García-Esquinas E, Rodríguez-Artalejo F, Lopez-Garcia E. Changes in Dietary Intake of Animal and Vegetable Protein and Unhealthy Aging. The American Journal of Medicine. 2020;133(2):231-9.e7.

899. Seddon JM, Cote J, Rosner B. Progression of Age-Related Macular Degeneration: Association With Dietary Fat, Transunsaturated Fat, Nuts, and Fish Intake. AMA Arch Ophthalmol. 2003;121(12):1728-37.

900. Shang X, Scott D, Hodge AM, English DR, Giles GG, Ebeling PR, Sanders KM. Dietary protein intake and risk of type 2 diabetes: results from the Melbourne Collaborative

Cohort Study and a meta-analysis of prospective studies. Am J Clin Nutr. 2016;104(5):1352-65.

901. Sluijs I, Beulens JW, van der Schouw YT, van der AD, Buckland G, Kuijsten A, et al. Dietary glycemic index, glycemic load, and digestible carbohydrate intake are not associated with risk of type 2 diabetes in eight European countries. J Nutr. 2013;143(1):93-9.

902. Tsai CJ, Leitzmann MF, Willett WC, Giovannucci EL. Glycemic load, glycemic index, and carbohydrate intake in relation to risk of cholecystectomy in women. Gastroenterology. 2005;129(1):105-12.

903. Liu S, Willett WC, Stampfer MJ, Hu FB, Franz M, Sampson L, et al. A prospective study of dietary glycemic load, carbohydrate intake, and risk of coronary heart disease in US women. Am J Clin Nutr. 2000;71(6):1455-61.

904. Oomen CM, Ocké MC, Feskens EJ, Kok FJ, Kromhout D. alpha-Linolenic acid intake is not beneficially associated with 10-y risk of coronary artery disease incidence: the Zutphen Elderly Study. Am J Clin Nutr. 2001;74(4):457-63.

905. Sabir Z, Hjartåker A, Dierkes J, Rosendahl-Riise H. The Association of Isocaloric Substitution of Dietary Protein in Middle Age with Muscle Mass and Strength in Old Age: The Hordaland Health Study. Curr dev nutr. 2024;8(1):102052.

906. Ardisson Korat AV, Shea MK, Jacques PF, Sebastiani P, Wang M, Eliassen AH, et al. Dietary protein intake in midlife in relation to healthy aging – results from the prospective Nurses' Health Study cohort. The American Journal of Clinical Nutrition. 2024;119(2):271-82.

907. Carballo-Casla A, Sotos-Prieto M, García-Esquinas E, A Struijk E, Caballero FF, Calderón-Larrañaga A, et al. Animal and vegetable protein intake and malnutrition in older adults: a multicohort study. J Nutr Health Aging. 2024;28(1):100002.

908. Ivey KL, Nguyen X-MT, Li R, Furtado J, Cho K, Gaziano JM, et al. Association of dietary fatty acids with the risk of atherosclerotic cardiovascular disease in a prospective cohort of United States veterans. The American Journal of Clinical Nutrition. 2023;118(4):763-72.

909. Kelly RK, Tong TYN, Watling CZ, Reynolds A, Piernas C, Schmidt JA, et al. Associations between types and sources of dietary carbohydrates and cardiovascular disease risk: a prospective cohort study of UK Biobank participants. BMC Medicine. 2023;21(1):34.

910. Liu Z, Huang H, Xie J, Hou L, Xu C. Different dietary carbohydrate component intakes and long-term outcomes in patients with NAFLD: results of longitudinal analysis from the UK Biobank. Nutr J. 2023;22(1):67.

911. Mei Z, Li Y, Dennis K, Hu F, Willett W, Stampfer M, Wang D. P19-031-23 Associations of Types of Dietary Sugar With Risk of Type 2 Diabetes in US Men and Women. Curr dev nutr. 2023;7:101365.

912. Qian S, Fu M, Han L, Sun W, Sun H. Dietary protein sources, genetics, and cardiovascular disease incidence. J Affect Disord. 2024;354:116-25.

913. Zheng J-S, Steur M, Imamura F, Freisling H, Johnson L, van der Schouw YT, et al. Dietary intake of plant- and animal-derived protein and incident cardiovascular diseases: the pan-European EPIC-CVD case–cohort study. The American Journal of Clinical Nutrition. 2024;119(5):1164-74.

914. Forouhi NG, Sharp SJ, Du H, van der AD, Halkjaer J, Schulze MB, et al. Dietary fat intake and subsequent weight change in adults: results from the European Prospective Investigation into Cancer and Nutrition cohorts. Am J Clin Nutr. 2009;90(6):1632-41.

915. Lucas M, Mirzaei F, O'Reilly EJ, Pan A, Willett WC, Kawachi I, et al. Dietary intake of n-3 and n-6 fatty acids and the risk of clinical depression in women: a 10-y prospective follow-up study. Am J Clin Nutr. 2011;93(6):1337-43.

916. Altorf-van der Kuil W, Engberink MF, Ijpma I, Verberne LD, Toeller M, Chaturvedi N, et al. Protein intake in relation to risk of hypertension and microalbuminuria in patients with type 1 diabetes: the EURODIAB Prospective Complications Study. J Hypertens. 2013;31(6):1151-9.

917. Chiu CJ, Hubbard LD, Armstrong J, Rogers G, Jacques PF, Chylack LT, Jr., et al. Dietary glycemic index and carbohydrate in relation to early age-related macular degeneration. Am J Clin Nutr. 2006;83(4):880-6.

918. Thiébaut AC, Jiao L, Silverman DT, Cross AJ, Thompson FE, Subar AF, et al. Dietary fatty acids and pancreatic cancer in the NIH-AARP diet and health study. J Natl Cancer Inst. 2009;101(14):1001-11.
